# Arylboronic Acid-Catalyzed Racemization of Secondary and Tertiary Alcohols

**DOI:** 10.1021/acs.joc.2c01602

**Published:** 2022-09-08

**Authors:** Gregory R. Boyce, Stefania F. Musolino, Jianing Yang, Andrew D. Smith, James E. Taylor

**Affiliations:** † EaStCHEM, School of Chemistry, University of St Andrews, North Haugh, St Andrews, Fife KY16 9ST, U.K.; ‡ Department of Chemistry and Physics, Florida Gulf Coast University, Fort Myers, Florida 33965, United States; § Department of Chemistry, University of Bath, Claverton Down, Bath, Somerset BA2 7AY, U.K.

## Abstract

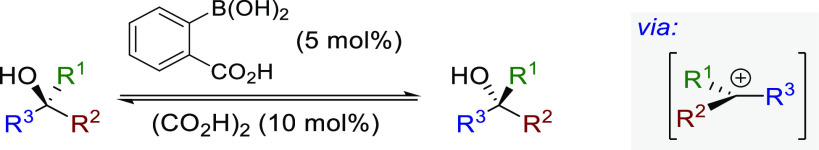

The use of 2-carboxyphenylboronic acid (5 mol %) and oxalic acid (10 mol %) with 2-butanone as a solvent for the racemization of a range of enantiomerically pure secondary and tertiary alcohols is demonstrated. The process is postulated to proceed via reversible Brønsted acid-catalyzed C–O bond cleavage through an achiral carbocation intermediate.

Despite tremendous advances in enantioselective synthesis, the kinetic resolution (KR) of racemic mixtures remains a cornerstone of asymmetric synthesis in academia and industry.^1^ The major limitation of this widely used approach to generate enantiomerically pure compounds is the theoretical maximum 50% yield of a single enantiomer. One strategy to improve efficiency is to racemize the undesired enantiomer to allow recycling of the material. In the most effective case, a dynamic kinetic resolution (DKR) involves the process of combining rapid in situ substrate racemization with a KR, potentially leading to quantitative product yields in enantiomerically pure form.^2^

Owing to the synthetic importance of enantioenriched secondary alcohols, several methods have been developed for their racemization to enable recirculation of the undesired enantiomer in DKR processes.^2^ The most widely used methods in this area rely on reversible removal of the stereogenic carbinol hydrogen atom, either through deprotonation of enolizable protons or, more commonly, through transition-metal promoted hydrogen-transfer through a dehydrogenation–hydrogenation mechanism via an achiral ketone intermediate (Scheme 1a).^3^ A limitation of such processes is that they cannot be applied to *tertiary* alcohols where no carbinol hydrogen exists. This type of racemization requires a conceptually distinct approach where the reversible dehydration of the C–OH bond to form an achiral carbocation intermediate is the most feasible method (Scheme 1b). This approach can be challenging to implement since generating the highly reactive carbocation intermediate can lead to several undesired pathways including alkene formation, rearrangement, and etherification. To date, only a limited number of heterogeneous catalysts, including acidic zeolites,^4^ acidic resins,^5^ and vanadyl sulfate,^6^ have been investigated for the racemization of secondary benzylic alcohols via a cationic intermediate and employed in a DKR. Furthermore, only two examples of acid-promoted racemization of tertiary alcohols have been reported. Bäckvall and co-workers used Dowex 50wX8 resin for the efficient heterogeneous racemization of a small range of acyclic tertiary alcohols where water was used as a solvent (Scheme 1c) to avoid undesired elimination and/or rearrangement processes.^7^ In 2020, Gröger and co-workers reported the only example of the DKR of a tertiary alcohol,^8^ using an oxovanadium-catalyst immobilized on mesoporous silica for the racemization in combination with enzymatic kinetic resolution. Only one substrate was investigated in this protocol, and multiple sequential additions of each catalyst were required over 13 days to achieve high conversion to product with excellent enantioselectivity.

**Scheme 1:**
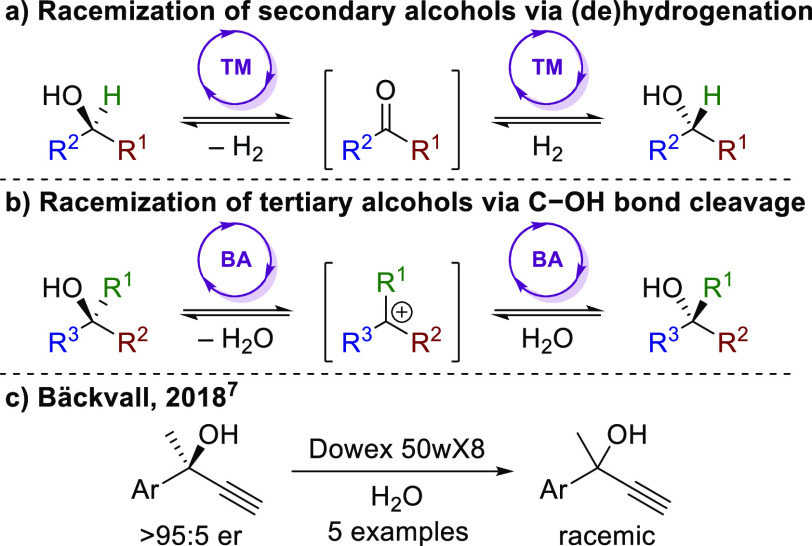
Catalytic Racemization of Alcohols TM = transition metal catalyst; BA = Brønsted acid catalyst.

Building upon this work, the development of a more general homogeneous Brønsted acid-catalyzed dehydrative racemization that could potentially be applied to *both* secondary and tertiary alcohols would represent an advance on existing methods. In this context, an investigation of arylboronic acids as potential catalysts for the racemization of alcohols is described. While the ability of arylboronic acids to promote catalytic dehydration in a variety of S_N_1 type substitution processes has previously been demonstrated, their use as racemization catalysts has not been detailed to date.^9^

Initial studies focused on the racemization of (*R*)-3-phenyl-3-hydroxyoxindole **1** (Table 1),^10^ which was readily obtained as a single enantiomer through isothiourea-catalyzed acylative kinetic resolution.^11^ A range of arylboronic acids **2**–**7** (5 mol %) was screened in combination with oxalic acid (10 mol %) as a cocatalyst, which is known to reversibly condense with arylboronic acids to form the corresponding boronate ester in situ.^12^ Preliminary screening was performed on a small-scale and the enantiomeric excess of the crude material was assessed by analytical HPLC on a chiral stationary phase. Phenylboronic acid **2** gave minimal racemization after 16 h at 40 °C in chloroform (entry 1); however, the more electron-deficient arylboronic acids **3**–**7** provided greater reduction in enantiomeric excess under the same conditions (entries 2–4). The most promising catalysts identified were pentafluorophenylboronic acid **6** and 2-carboxyphenylboronic acid **7**, with the latter generating the racemate of **1** (entries 5 and 6). Control studies indicated that oxalic acid (10 mol %) alone was not capable of promoting racemization (entry 7) and neither was 2-carboxyphenylboronic acid **7** (5 mol %) in isolation (entry 8), demonstrating that the combination of the arylboronic acid and oxalic acid is essential for reactivity. The use of Dowex 50wX8 also did not lead to racemization.^10^ Repeating the successful racemization on a preparative scale revealed competing decomposition of **1** through analysis of the crude ^1^H NMR. Possible side reactions arising from formation of a possible carbocation intermediate include etherification,^12b^ and Friedel–Crafts alkylation processes,^9,13^ which are both precedented under arylboronic acid catalysis. Unfortunately, the side products could not be isolated in sufficient quantities to allow for unambiguous identification. A solvent screen was therefore conducted to find conditions that promoted clean racemization with minimal loss of material. The use of arylboronic acid **7** and oxalic acid in acetonitrile gave complete racemization of **1**, but was accompanied by significant decomposition, with only 25% of *rac*-**1** returned (entry 9). Inspired by the work of Niggemann,^14^ ketone-based solvents capable of stabilizing a cationic intermediate were trialled. The use of cyclopentanone diminished the racemization (entry 10), while acetone gave scalemic **1** in 84:16 er and with a more promising 81% recovery by NMR (entry 11). The use of 2-butanone gave a good balance of reactivity and selectivity, providing **1** in 61:39 er and 82% recovery (entry 12). Increasing the reaction temperature to 60 °C gave complete racemization after only 3 h, with *rac*-**1** recovered in 70% isolated yield (entry 13).

**Table 1:**
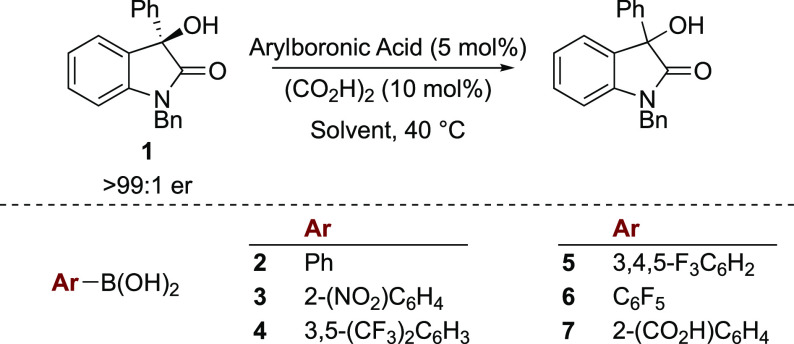
Reaction Optimization

entry	Boronic acid	solvent	yield (%)^a^	er^b^
1	**2**	CHCl_3_	N/D	93:7
2	**3**	CHCl_3_	N/D	82:18
3	**4**	CHCl_3_	N/D	65:35
4	**5**	CHCl_3_	N/D	79:21
5	**6**	CHCl_3_	N/D	54:46
6	**7**	CHCl_3_	N/D	50:50
7	None	CHCl_3_	N/D	>99:1
8^c^	**7**	CHCl_3_	N/D	>99:1
9	**7**	MeCN	25	50:50
10	**7**	Cyclopentanone	99	91:9
11	**7**	Acetone	81	84:16
12	**7**	2-Butanone	82	61:39
13^d^	**7**	2-Butanone	70^e^	50:50

a Determined by ^1^H NMR using relative integrals of product peak and impurities.

b Determined by HPLC analysis on a chiral stationary phase.

c No oxalic acid.

d Reaction at 60 °C, 3 h.

e Isolated yield.

With the optimized conditions for racemization developed, the scope and limitations of this process were assessed by changing the steric and electronic parameters of the heterocyclic tertiary alcohol substrate (Scheme 2). Variation of the *N*-substituent showed that *N*-benzyl, *N*-methyl, and *N*-allyl substituents are all tolerated in this protocol, giving racemic material **1**, **8**, and **9**, respectively, from enantioenriched substrates in good to excellent yield. Similarly, incorporation of a *C*(5)-methyl substituent within oxindole **10** was tolerated. Incorporation of an electron-donating 4-MeOC_6_H_4_ substituent at the *C*(3) position within **11** leads to significant byproduct formation under the standard conditions, likely due to the increased stability of the intermediate carbocation. Two racemic diastereoisomeric products were obtained, consistent with undesired C–C bond formation with the enol tautomer of the 2-butanone solvent.^10,12c^ Switching the solvent to acetone allowed racemic **11** to be isolated in 42% yield, alongside 39% of the ketone obtained from C–C bond formation with the enol of acetone. In contrast, incorporation of a 2-naphthyl group gave effective racemization, forming racemic **12** in 82% yield. Extension to alternative *C*(3)-alkyl substituted alcohols **13**–**15** showed a reduction in enantiomeric ratio from that of the starting materials but slower racemization than that observed with the *C*(3)-aryl-substituted oxindoles. This trend is consistent with the expectedly enhanced cation stabilizing properties of the doubly benzylic carbocation compared to the *C*(3)-alkyl-substituted carbocation.

**Scheme 2:**
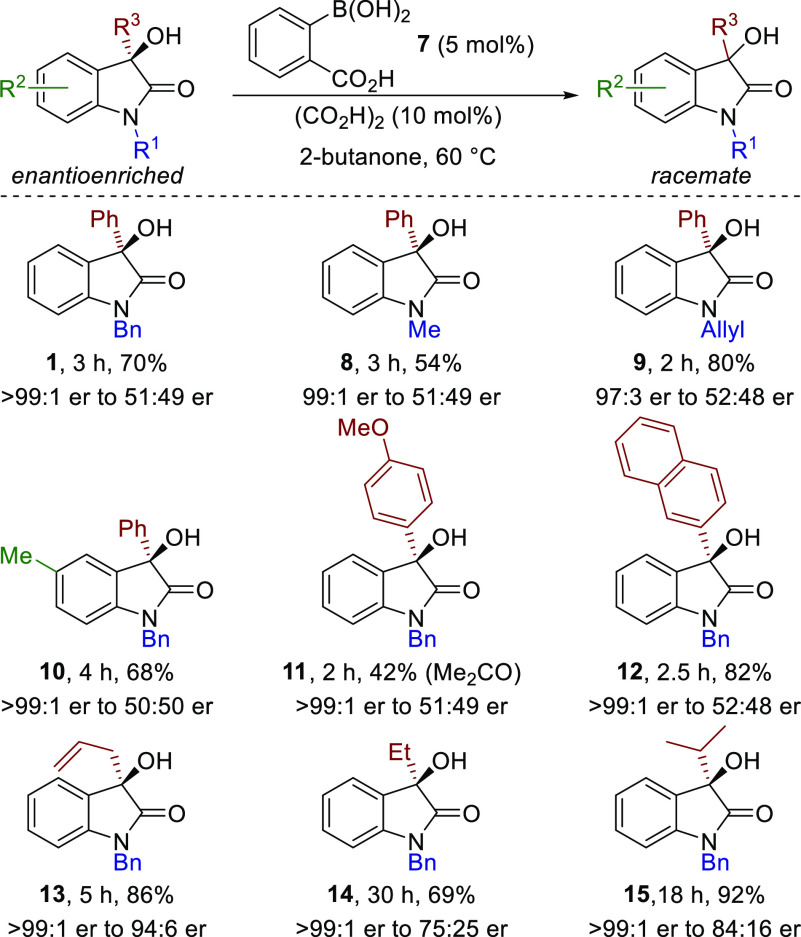
Scope and Limitations of Tertiary Alcohol Racemization Isolated yields after purification by column chromatography. er determined by HPLC analysis on a chiral stationary phase.

Extension of this methodology to the racemization of secondary alcohol substrates was then investigated (Scheme 3). The reaction of enantiomerically pure (*S*)-1-phenylethanol **16** with 2-carboxyphenylboronic acid **7** (5 mol %) and oxalic acid (10 mol %) showed that racemization proceeded at a lower temperature than observed for the tertiary alcohols, with reaction at 40 °C in 2-butanone giving a 75:25 mixture of *rac*-**16** to the corresponding symmetric ether (50:50 dr). Performing the reaction at room temperature improved the selectivity for the racemization, providing a 93:7 mixture of alcohol **16** (55:45 er) to its symmetric ether (50:50 dr), which allowed the alcohol to be recovered in 75% yield. Increasing the steric bulk of the alcohol through introduction of branched alkyl substituents disfavored ether formation but required increasing temperature to achieve racemization likely due to diminished solvation of the carbocation intermediate. For example, *i*-Pr-substituted alcohol **17** was isolated in 71% yield and 54:46 er at 40 °C, while *t*-Bu-substituted alcohol **18** was isolated in 85% yield and 52:48 er after 1.5 h at 60 °C. Varying the electronic characteristics of the aryl substituent at the carbinol was next investigated. The introduction of electron-withdrawing aryl groups disfavored racemization, with a 4-CF_3_C_6_H_4_ substituent on alcohol **19** leading to no racemization even after prolonged heating at 75 °C, while the 4-ClC_6_H_4_ variant **20** provided partial racemization at 60 °C. These results mirror the findings of Bäckvall and co-workers, where electron-deficient benzylic alcohols underwent racemization at a significantly slower rate.^7^ Alcohol **21** bearing a weakly electron-donating 4-MeC_6_H_4_ substituent was readily racemized at 40 °C, as was a 2-naphthyl variant **22**. The racemization of alcohol **23** bearing a strongly electron-donating 4-MeOC_6_H_4_ substituent proceeded even at 0 °C, with higher temperatures leading to multiple side products. Alkynyl alcohol **24** racemized readily at 60 °C, giving a 65:35 mixture of *rac*-**24** (59% yield) to the corresponding symmetric ether (32% yield, 50:50 dr). Decreasing the temperature to 40 °C inhibits the etherification pathway; however, the rate of racemization was slowed. In contrast, allylic alcohol **25** led to extensive formation of the ether side-product even at room temperature, forming *rac*-**25** in only 22% yield. It is noteworthy that the allylic **24** and propargylic **25** alcohols provided no rearranged products via the known boronic acid-catalyzed transposition.^15^ This suggests that the mechanism is likely via Brønsted acid catalysis instead of Lewis acid catalysis.

**Scheme 3:**
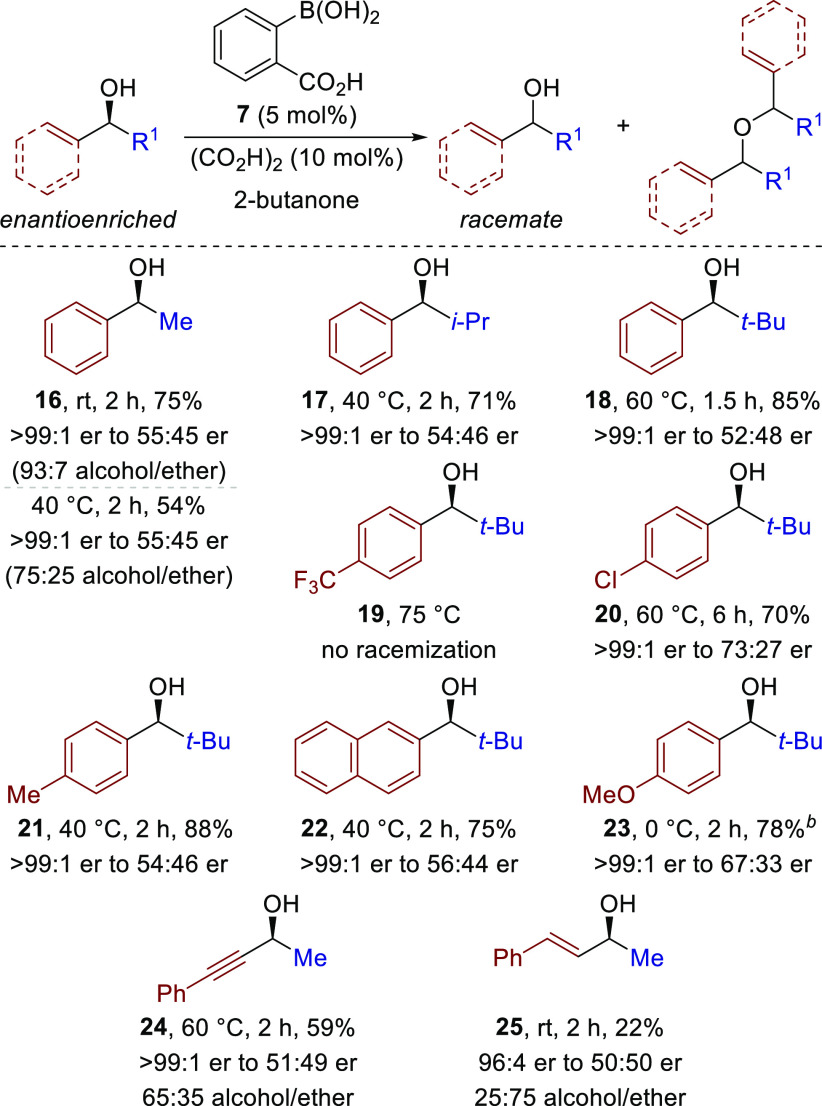
Scope and Limitations of Secondary Alcohol Racemization Isolated yields after purification by column chromatography. er determined by HPLC analysis on a chiral stationary phase. Reaction performed in MeCN.

To further exemplify this protocol, the application to the epimerization of a bioactive secondary alcohol, podophyllotoxin **26**, containing multiple stereocenters and functional group moieties was investigated. Derivatives of podophyllotoxin **26** and its diastereoisomer, *epi*-podophyllotoxin **27**, have been widely investigated due to their potent activity against cancer cells via inhibition of tubulin polymerization, and a number of methods for their synthesis have been developed.^16^ Treatment of commercially available podophyllotoxin **26** to the catalytic protocol in acetonitrile at room temperature resulted in selective epimerization at the benzylic carbinol center to give a 60:40 mixture of podophyllotoxin **26** to *epi*-podophyllotoxin **27** in 81% yield with purification allowing for partial separation (Scheme 4). Given that commercial *epi*-podophyllotoxin **27** is significantly more expensive than podophyllotoxin **26**, this protocol provides a method for its preparation.

**Scheme 4:**
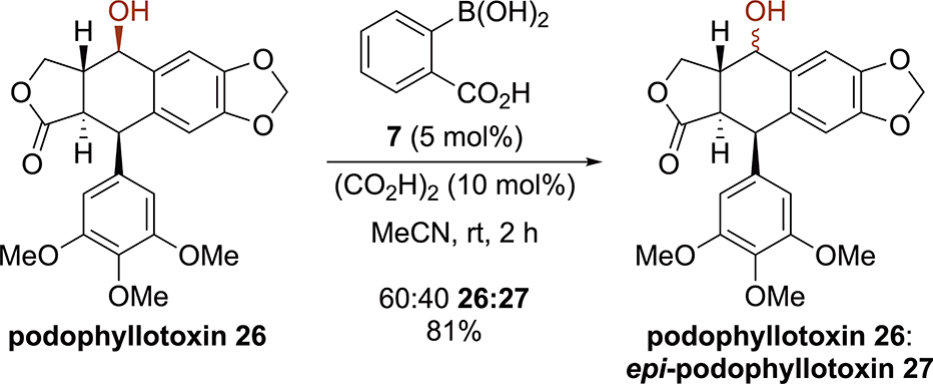
Application to the Epimerization of Podophyllotoxin

Although much controversy over the mode of action of alcohol activation with boronic acids exists, recent work by both Hall^13^ and Taylor^12b^ indicates that a Brønsted acid or H-bonding pathway dominates over the alternative Lewis acid route. To probe whether the combined 2-carboxyphenylboronic acid **7**/oxalic acid system acts as either a Lewis acid or Brønsted acid in the developed racemization process, a control experiment was performed with enantiomerically pure (*R*)-3-hydroxyoxindole **1** under the standard reaction conditions with the addition of catalytic 2,6-di-*tert*-butylpyridine (5 mol %, Scheme 5a). No racemization was observed after 2 h at 60 °C, with inhibition being consistent with a Brønsted acid-catalyzed pathway likely being operational. The symmetric ether of 1-phenylethanol **28** (50:50 dr) was also subjected to the reaction conditions in the presence of water (2 or 10 equiv) to determine the reversibility of the etherification. Conversion by ^1^H NMR demonstrated that the ratio of ether **28** (50:50 dr) to alcohol **16** was equivalent regardless of the amount of water added (Scheme 5b). This further supports the hypothesis that this process proceeds through a Brønsted acid catalyzed S_N_1 process.

**Scheme 5:**
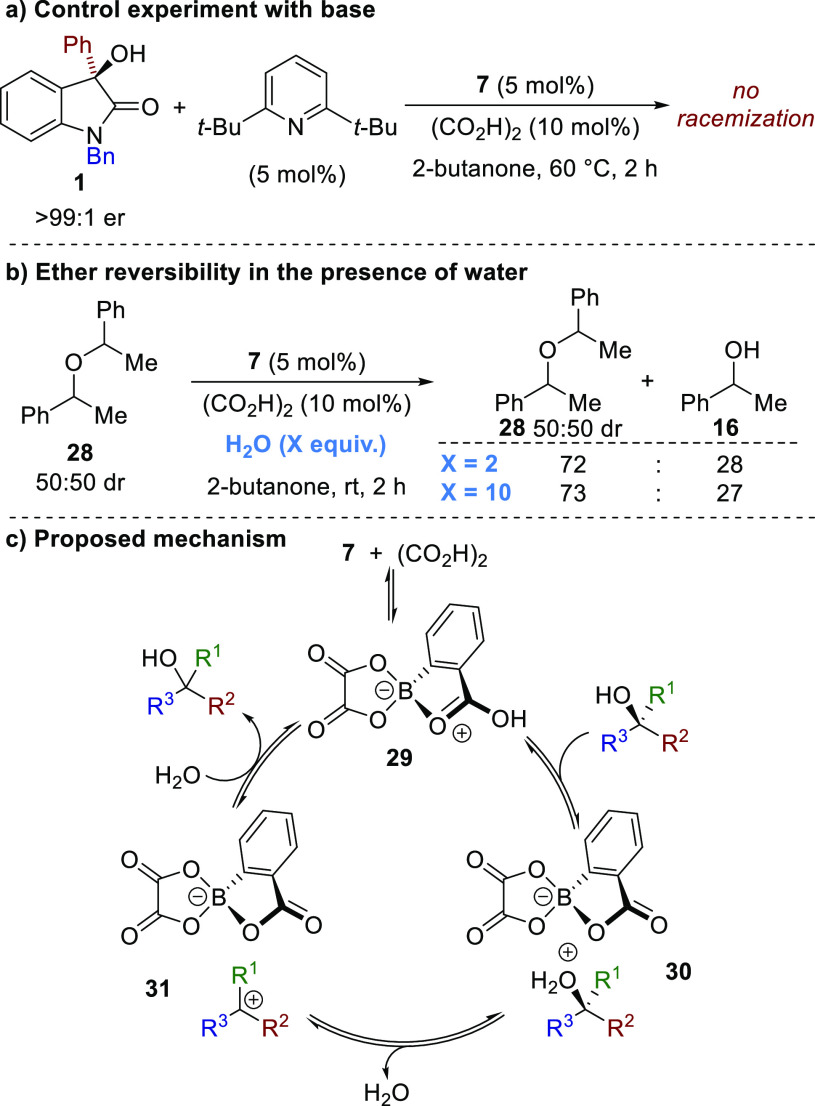
Mechanistic Considerations

A possible mechanism for racemization is outlined in Scheme 5c. In situ condensation between 2-carboxyphenylboronic acid **7** and oxalic acid is assumed to form boronate complex **29** with increased Brønsted acidity compared with either starting material. In this context, both Mattson^17a^ and Maruoka^17b^ have reported that cyclic boronate esters derived from 2-carboxyphenylboronic acid can act as Lewis acid-assisted Brønsted acid catalysts, with the latter providing X-ray crystallographic evidence for formation of spirocyclic boronate species similar to **29**. A ^11^B NMR experiment in acetone-*d*_6_ reacting **7** with oxalic acid (2 equiv) showed one predominant species in solution, consistent with the formation of a tetrahedral sp^3^-hybridized boron compound (δ_B_ = 9.6 ppm).^18^ Direct HRMS analysis of this solution also confirmed the molecular ion of **29** as the major compound present.^10^ However, under the reaction conditions, **29** may exist as part of a dynamic equilibrium with other hydrated forms, and it is therefore difficult to unambiguously define the active catalyst present in solution. Boronate **29**, or a related hydrate, is proposed to behave as an enhanced Brønsted acid that can protonate the enantiopure alcohol, leading to an initial ion pair such as **30**. Reversible C–O bond cleavage is achieved through ionization to generate the corresponding carbocation intermediate **31**, followed by a nonselective hydration event resulting in racemization.

In conclusion, 2-carboxylphenylboronic acid **7** (5 mol %) in combination with oxalic acid (10 mol %) is an efficient catalytic system for the racemization of enantiomerically enriched tertiary 3-hydroxy-3-substituted oxindoles and a range of secondary benzylic alcohols. The process is thought to occur by reversible Brønsted acid-catalyzed C–O bond cleavage to form an achiral carbocation intermediate.

## Experimental Section

### 1 General Information

Reactions involving moisture sensitive reagents were carried out in flame-dried glassware under a nitrogen (N_2_) atmosphere using standard vacuum line techniques and using anhydrous solvents. Anhydrous solvents (CH_2_Cl_2_ and toluene) were obtained from an anhydrous solvent system (purified using an alumina column, Mbraun SPS-800). All other reactions were performed in standard glassware with no precautions to exclude air or moisture. Solvents and commercial reagents were used as supplied without further purification unless otherwise stated. Room temperature (r.t.) refers to 20–25 °C. Temperatures of 0 °C and −78 °C were obtained using ice/water and CO_2_(*s*)/acetone baths, respectively. Reflux conditions were obtained using a DrySyn, oil bath, or sand bath equipped with a contact thermometer. Analytical thin layer chromatography was performed on precoated aluminum plates (Kieselgel 60 F_254_ silica). TLC visualization was carried out with ultraviolet light (254 nm), followed by staining with a 1% aqueous KMnO_4_ solution. Manual column chromatography was performed in glass columns fitted with porosity 3 sintered discs over Kieselgel 60 silica using the solvent system stated. Automated chromatography was performed on a Biotage Isolera Four running Biotage OS578 with a UV–vis detector using the method stated and cartridges filled with Kieselgel 60 silica. Melting points were recorded on an Electrothermal 9100 melting point apparatus and are uncorrected. Optical rotations [α]_*D*_^20^ were measured on a PerkinElmer Model 341 polarimeter operating at the sodium D line with a 100 mm path cell at 20 °C. HPLC analyses were obtained using either a Shimadzu HPLC consisting of a DGU-20A5 degassing unit, LC-20AT liquid chromatography pump, SIL-20AHT autosampler, CMB-20A communications bus module, SPD-M20A diode array detector and a CTO-20A column oven; or a Shimadzu HPLC consisting of a DGU-20A5R degassing unit, LC-20AD liquid chromatography pump, SIL-20AHT autosampler, SPD-20A UV/vis detector and a CTO-20A column oven. Separation was achieved using DAICEL CHIRALCEL OD-H or DAICEL CHIRALPAK AD-H or AS-H columns. All HPLC traces of enantiomerically enriched compounds were compared with authentic racemic spectra. ^1^H, ^13^C, ^19^F nuclear magnetic resonance (NMR) spectra were acquired on either a Bruker Avance 300 (^1^H 300 MHz), Bruker Avance II 400 (^1^H 400 MHz; ^13^C 101 MHz; ^19^F 376 MHz), or a Bruker Avance II 500 (^1^H 500 MHz; ^13^C 126 MHz; ^19^F 476 MHz) spectrometer at ambient temperature in the deuterated solvent stated. All chemical shifts are quoted in parts per million (ppm) and referenced to the residual solvent peak. All coupling constants, *J*, are quoted in Hz. Multiplicities are indicated by s (singlet), d (doublet), t (triplet), q (quartet), and combinations thereof, and m (multiplet). The abbreviation Ar is used to denote aromatic, Ph to denote phenyl, Bn to denote benzyl, br to denote broad, and app to denote apparent. Infrared spectra (ν_max_) were recorded on a Shimadzu IRAffinity-1 Fourier transform IR spectrophotometer fitted with a Specac Quest ATR accessory (diamond puck). Spectra were recorded of either thin films or solids, with characteristic absorption wave numbers (max) reported in cm^–1^. High resolution mass spectrometry (HRMS) data were acquired by electrospray ionization time-of-flight (ESI-TOF) at the University of St Andrews.

### 2 General Procedures

#### General Procedure A: Racemization of Tertiary Oxindoles

The appropriate alcohol (1 equiv), boronic acid (5 mol %), and oxalic acid (10 mol %) were added to a vial. If the reaction was performed on a small-scale, stock solutions (vide infra) of the two catalysts were used and the THF from the stock solution was removed in vacuo prior to the start of the reaction. The reactants were then dissolved in the required solvent (0.25 M) and the mixture was heated at 60 °C. The reaction was stirred for the required time and then filtered through a silica pad and concentrated under reduced pressure. The alcohol was analyzed by chiral HPLC and ^1^H NMR.

#### General Procedure B: Racemization of Secondary Alcohols

The appropriate alcohol (1 equiv), boronic acid (5 mol %), and oxalic acid (10 mol %) were added to a vial. If the reaction was performed on a small-scale, stock solutions (vide infra) of the two catalysts were used and the THF from the stock solution was removed in vacuo prior to the start of the reaction. The reactants were dissolved in the required solvent (0.25 M) and the mixture was heated to the required temperature for the described time. The reaction was diluted with Et_2_O and washed sequentially with 1 M NaOH, brine, then dried with MgSO_4_, filtered, and concentrated in vacuo. The reaction mixture was then filtered through a silica pad and concentrated under reduced pressure. The alcohol was analyzed by chiral HPLC and ^1^H NMR.

#### Preparation of 2-Carboxyphenylboronic Acid Stock Solution (0.015 M)

Boronic acid (5 mg, 0.03 mmol) and THF (1 mL) were added to a 2 mL volumetric flask. Once the mixture was homogeneous (after sonication) THF was added until the total volume of the mixture had reached 2 mL.

#### Preparation of Oxalic Acid Stock Solution (0.11 M)

Oxalic acid (20 mg, 0.22 mmol) and THF (1 mL) were placed in a 2 mL volumetric flask. Once the mixture was homogeneous (after sonication) THF was added until the total volume of the mixture had reached 2 mL.

### 3 Racemization of Enantioenriched Alcohols

#### Racemization of (*R*)-1-Benzyl-3-hydroxy-3-phenylindolin-2-one (**1**)

Following General Procedure A, (*R*)-1-benzyl-3-hydroxy-3-phenylindolin-2-one **1** (>99:1 er, 200 mg, 0.64 mmol), 2-carboxyphenylboronic acid 7 (0.015 M, 2.1 mL, 32 μmol, 5 mol %), and oxalic acid (0.11 M, 570 μL, 64 μmol, 10 mol %) were reacted in 2-butanone (2.6 mL) for 3 h at 60 °C. The reaction was concentrated under reduced pressure and purified by column chromatography (Petrol:EtOAc 3:1) to give *rac*-1-benzyl-3-hydroxy-3-phenylindolin-2-one **1** (140 mg, 0.45 mmol, 70%). Chiral HPLC analysis, Chiralpak AD-H (90:10 hexane:IPA, flow rate 1.25 mL min^–1^, 211 nm, 40 °C) *t*_R_ (*R*): 17.8 min, *t*_R_ (*S*): 21.6 min, 51:49 (*R*:*S*) er.

#### Racemization of (*R*)-3-Hydroxy-1-methyl-3-phenylindolin-2-one (**8**)

Following General Procedure A, (*R*)-3-hydroxy-1-methyl-3-phenylindolin-2-one **8** (>99:1 er, 68 mg, 0.3 mmol), 2-carboxyphenylboronic acid **7** (0.015 M, 1.0 mL, 15 μmol, 5 mol %), and oxalic acid (0.11 M, 270 μL, 30 μmol, 10 mol %) were reacted in 2-butanone (1.2 mL) for 3 h at 60 °C. The reaction was concentrated under reduced pressure and purified by column chromatography (Petrol:EtOAc 1:1) to give *rac*-3-hydroxy-1-methyl-3-phenylindolin-2-one **8** (37 mg, 0.16 mmol, 54%). Chiral HPLC analysis, Chiralpak AD-H (95:5 hexane:IPA, flow rate 1.0 mL min^–1^, 211 nm, 30 °C) *t*_R_ (*R*): 27.4 min, *t*_R_ (*S*): 30.7 min, 51:49 (*R*:*S*) er

#### Racemization of (*R*)-3-Hydroxy-1-allyl-3-phenylindolin-2-one (**9**)

Following General Procedure A, (*R*)-3-hydroxy-1-allyl-3-phenylindolin-2-one **9** (97:3, 80 mg, 0.3 mmol), 2-carboxyphenylboronic acid **7** (0.015 M, 1.0 mL, 15 μmol, 5 mol %), and oxalic acid (0.11 M, 270 μL, 30 μmol, 10 mol %) were reacted in 2-butanone (1.2 mL) for 2 h at 60 °C. The reaction was concentrated under reduced pressure and purified by column chromatography (Petrol:EtOAc 1:3) to give *rac*-3-hydroxy-1-allyl-3-phenylindolin-2-one **9** (64 mg, 0.24 mmol, 80%). Chiral HPLC analysis, Chiralpak OD-H (95:5 hexane:IPA, flow rate 1.0 mL min^–1^, 211 nm, 30 °C) *t*_R_ (*S*): 14.0 min, *t*_R_ (*R*): 16.0.1 min, 52:48 (*R*:*S*) er.

#### Racemization of (*R*)-1-Benzyl-3-hydroxy-5-methyl-3-phenylindolin-2-one (**10**)

Following General Procedure D, (*R*)-1-benzyl-3-hydroxy-5-methyl-3-phenylindolin-2-one **10** (>99:1 er, 99 mg, 0.3 mmol), 2-carboxyphenylboronic acid **7** (0.015 M, 1.0 mL, 15 μmol, 5 mol %), and oxalic acid (0.11 M, 270 μL, 30 μmol, 10 mol %) were reacted in 2-butanone (1.2 mL) for 4 h at 60 °C. The reaction was concentrated under reduced pressure and purified by column chromatography (Petrol:EtOAc 1:5) to give *rac*-1-benzyl-3-hydroxy-5-methyl-3-phenylindolin-2-one **10** (67 mg, 0.20 mmol, 68%). Chiral HPLC analysis, Chiralpak AD-H (90:10 hexane:IPA, flow rate 1.5 mL min^–1^, 211 nm, 40 °C) *t*_R_ (*R*): 12.2 min, *t*_R_ (*S*): 15.6 min, 50:50 (*R*:*S*) er.

#### Attempted Racemization of (*R*)-1-Benzyl-3-hydroxy-3-(4-methoxyphenyl)indolin-2-one (**11**) in 2-Butanone

Following General Procedure A, (*R*)-1-benzyl-3-hydroxy-3-(4-methoxyphenyl)indolin-2-one **11** (>99:1 er, 98 mg, 0.28 mmol), 2-carboxyphenylboronic acid **7** (0.015 M, 0.94 mL, 14 μmol, 5 mol %), and oxalic acid (0.11 M, 255 μL, 28 μmol, 10 mol %) were reacted in 2-butanone (1.1 mL) for 2 h at 60 °C. The reaction was concentrated under reduced pressure and purified by column chromatography (Petrol:EtOAc 1:3) to give two isomers of 1-benzyl-3-(4-methoxyphenyl)-3-(3-oxobutan-2-yl)indolin-2-one **S23** (25 mg, 0.073 mmol, 26%) as a colorless oil and **S24** (25 mg, 0.073 mmol, 26%) as a white solid. 1-Benzyl-3-(4-methoxyphenyl)-3-(3-oxobutan-2-yl)indolin-2-one **S23**: ^1^H NMR (400 MHz, CDCl_3_) δ_H_ 7.74 (1H, dd, *J* 7.6, 0.9), 7.38–7.44 (2H, m), 7.18–7.32 (6H, m), 7.10 (1H, td, *J* 7.6 1.1), 6.79–6.85 (2H, m), 6.75 (1H, d, *J* 7.8), 4.90 (2H, d, *J* 1.7), 4.00 (1H, q, *J* 7.2), 3.77 (3H, s), 2.02 (3H, s), 0.97 (3H, d, *J* 7.2); ^13^C{^1^H} NMR (101 MHz, CDCl_3_) δ_C_ 209.6, 178.1, 158.9, 143.4, 136.0, 130.6, 129.1, 128.9, 128.4, 128.3, 128.0, 127.7, 127.3, 122.6, 114.0, 109.4, 57.6, 55.3, 53.5, 44.2, 31.1, 13.3; IR ν_max_ (film) 2931 (C–H), 2359, 1701 (C=O), 1607 (C=C), 1508, 1352, 1250, 1182 cm^–1^; HRMS (NSI^+^) calculated for C_26_H_25_NO_3_Na^+^ [M + Na]^+^ requires 422.1727, found 422.1713 (−3.3 ppm). 1-Benzyl-3-(4-methoxyphenyl)-3-(3-oxobutan-2-yl)indolin-2-one **S24**: mp 105–108 °C; ^1^H NMR (400 MHz, CDCl_3_) δ_H_ 7.33–7.40 (3H, m), 7.18–7.29 (6H, m), 7.07 (1H, td, *J* 7.6 1.0), 6.81–6.87 (2H, m), 6.74 (1H, d, *J* 7.8), 4.94 (1H, d, *J* 16.1), 4.84 (1H, d, *J* 16.1), 3.86 (1H, q, *J* 7.7), 3.78 (3H, s), 2.06 (3H, s), 1.37 (3H, d, *J* 7.7); ^13^C{^1^H} NMR (101 MHz, CDCl_3_) δ_C_ 208.5, 178.8, 159.1, 143.8, 136.0, 130.3, 130.1, 128.7, 128.7, 128.2, 127.5, 127.2, 125.8, 122.0, 114.1, 109.8, 56.6, 55.4, 54.8, 44.2, 29.0, 12.8; IR ν_max_ (solid) 2926 (C–H), 1697 (C=O), 1607 (C=C), 1508, 1354, 1253, 1180 cm^–1^; HRMS (NSI^+^) calculated for C_26_H_25_NO_3_Na^+^ [M + Na]^+^ requires 422.1727, found 422.1710 (−4.0 ppm).

#### Racemization of (*R*)-1-Benzyl-3-hydroxy-3-(4-methoxyphenyl)indolin-2-one (**11**) in acetone

Following General Procedure D, (*R*)-1-benzyl-3-hydroxy-3-(4-methoxyphenyl)indolin-2-one **11** (>99:1 er, 49 mg, 0.14 mmol), 2-carboxyphenylboronic acid **7** (0.015 M, 0.47 mL, 7 μmol, 5 mol %), and oxalic acid (0.11 M, 178 μL, 14 μmol, 10 mol %) were reacted in acetone (0.6 mL) for 2 h at 40 °C. The reaction was concentrated under reduced pressure and purified by column chromatography (Petrol:EtOAc 1:2) followed by flash column chromatography (CH_2_Cl_2_:EtOAc 95:5) to give *rac*-**11** as a yellow solid (21 mg, 42%), and 1-benzyl-3-(4-methoxyphenyl3-(2-oxopropyl)indolin-2-one **S25** as white solid (21 mg, 39%). 1-Benzyl-3-hydroxy-3-(4-methoxyphenyl)indolin-2-one **11**: Chiral HPLC analysis: Chiralpak IC (80:20 hexane:IPA, flow rate 1.0 mL min^–1^, 211 nm, 30 °C) *t*_R_ (*R*): 15.6 min, *t*_R_ (*S*): 21.0 min, 51:49 (*R*:*S*) er. 1-Benzyl-3-(4-methoxyphenyl)-3-(2-oxopropyl)indolin-2-one **S25**: mp 104–106 °C; ^1^H NMR (400 MHz, CDCl_3_) δ_H_ 7.20–7.35 (8H, m), 7.18 (1H, td, *J* 7.8 1.3), 7.03 (1H, td, *J* 7.7 0.9), 6.80–6.85 (2H, m), 6.73 (1H, d, *J* 7.9), 5.00 (1H, d, *J* 15.9), 4.90 (1H, d, *J* 15.9), 3.77 (3H, s), 3.65(1H, d, *J* 18.0), 3.52 (1H, d, *J* 18.0), 2.06 (3H, s); ^13^C{^1^H} NMR (101 MHz, CDCl_3_) δ_C_ 204.4, 178.8, 159.1, 143.9, 136.2, 131.8, 131.6, 128.8, 128.4, 127.9, 127.5, 127.3, 124.0, 122.4, 114.2, 109.7, 55.4, 52.6, 51.2, 44.3, 30.3; IR ν_max_ (solid) 2912 (C–H), 1705 (C=O), 1606 (C=C), 1508, 1355, 1256, 1180, 1168 cm^–1^; HRMS (NSI^+^) calculated for C_25_H_23_NO_3_Na^+^ [M + Na]^+^ requires 408.1576, found 408.1557 (−4.7 ppm).

#### Racemization of (*R*)-1-Benzyl-3-hydroxy-3-(naphthalen-2-yl)indolin-2-one (**12**)

Following General Procedure A, (*R*)-1-benzyl-3-hydroxy-3-(naphthalen-2-yl)indolin-2-one **12** (>99:1 er, 95 mg, 0.26 mmol), 2-carboxyphenylboronic acid **7** (0.015 M, 0.86 mL, 13 μmol, 5 mol %), and oxalic acid (0.11 M, 234 μL, 26 μmol, 10 mol %) were reacted in 2-butanone (1.0 mL) for 2.5 h at 60 °C. The reaction was concentrated under reduced pressure and purified by column chromatography (Petrol:EtOAc 1:3) to give *rac*-1-benzyl-3-hydroxy-3-(naphthalen-2-yl)indolin-2-one **12** (78 mg, 0.20 mmol, 82%). Chiral HPLC analysis: Chiralpak IA (70:30 hexane:IPA, flow rate 0.5 mL min^–1^, 211 nm, 30 °C) *t*_R_ (*R*): 24.8 min, *t*_R_ (*S*): 31.0 min, 52:48 (*R*:*S*) er.

#### Racemization of (*R*)-3-Allyl-1-benzyl-3-hydroxyindolin-2-one (**13**)

Following General Procedure A, (*R*)-3-allyl-1-benzyl-3-hydroxyindolin-2-one **13** (>99:1 er, 84 mg, 0.30 mmol), 2-carboxyphenylboronic acid **7** (0.015 M, 1.0 mL, 15 μmol, 5 mol %), and oxalic acid (0.11 M, 270 μL, 30 μmol, 10 mol %) were reacted in 2-butanone (1.2 mL) for 5 h at 60 °C. The reaction was concentrated under reduced pressure and purified by column chromatography to provide 1-benzyl-3-ethyl-3-hydroxylindolin-2-one **13** (72 mg, 0.26 mmol, 86%). Chiral HPLC analysis: Chiralpak OD-H (98:2 hexane:IPA, flow rate 1.0 mL min^–1^, 254 nm, 30 °C) *t*_R_ (*R*): 32.1 min, *t*_R_ (*S*): 38.4 min, 94:6 (*R*:*S*) er.

#### Racemization of (*R*)-1-Benzyl-3-ethyl-3-hydroxylindolin-2-one (**14**)

Following General Procedure A, (*R*)-1-benzyl-3-ethyl-3-hydroxylindolin-2-one **14** (>99:1 er, 75 mg, 0.28 mmol), 2-carboxyphenylboronic acid **7** (0.015 M, 0.93 mL, 14 μmol, 5 mol %), and oxalic acid (0.11 M, 255 μL, 28 μmol, 10 mol %) were reacted in 2-butanone (1.1 mL) for 30 h at 60 °C. The reaction was concentrated under reduced pressure and purified by column chromatography (Petrol:EtOAc 1:3) to give *rac*-1-benzyl-3-ethyl-3-hydroxylindolin-2-one **14** (51.4 mg, 0.19 mmol, 69%). Chiral HPLC analysis: Chiralpak IC (80:20 hexane:IPA, flow rate 1.0 mL min^–1^, 211 nm, 30 °C) *t*_R_ (*R*): 7.6 min, *t*_R_ (*S*): 12.5 min, 75:25 (*R*:*S*) er.

#### Racemization of (*R*)-1-Benzyl-hydroxy-3-isopropylindolin-2-one (**15**)

Following General Procedure A, (*R*)-1-benzyl-3-hydroxy-3-isopropyl indolin-2-one **15** (>99:1 er, 60 mg, 0.21 mmol), 2-carboxyphenylboronic acid **7** (0.015 M,166 μL, 10.7 μmol, 5 mol %), and oxalic acid (0.11 M, 19 μL, 21 μmol, 10 mol %) were reacted in 2-butanone (0.85 mL) for 18 h at 60 °C. The reaction was concentrated under reduced pressure and purified by column chromatography 1-benzyl-3-hydroxy-3-isopropyl indolin-2-one **15** (55 mg, 0.19 mmol, 92%). Chiral HPLC analysis: Chiralpak AD-H (98:2 hexane:IPA, flow rate 1.0 mL min^–1^, 211 nm, 30 °C) *t*_R_ (*R*): 23.6 min, *t*_R_ (*S*): 28.5 min, 84:16 (*R*:*S*) er.

#### Racemization of (*S*)-1-Phenylethanol (**16**)

Following General Procedure B, (*S*)-1-phenylethanol **16** (99.5:0.5 er, 366.5 mg, 3.0 mmol), 2-carboxyphenylboronic acid **7** (25 mg, 0.15 mmol, 5 mol %), and oxalic acid (27 mg, 0.3 mmol, 10 mol %) were reacted in 2-butanone (12 mL) for 2 h at room temperature. The reaction was concentrated under reduced pressure and purified by column chromatography (10% EtOAc:Hexane) to give *rac*-1-phenylethanol **16** (274 mg, 2.25 mmol, 75%). Chiral HPLC analysis: Chiralpak OD-H (95:5 hexane:IPA, flow rate 1.0 mL min^–1^, 220 nm, 30 °C) *t*_R_ (*R*): 8.1 min, *t*_R_ (*S*): 9.9 min, 55:45 (*S*:*R*) er.

#### Etherification of *rac*-1-Phenylethanol (**16**)

Following a modified General Procedure D, *rac*-1-phenylethanol **16** (366.5 mg, 3.0 mmol), 2-carboxyphenylboronic acid **7** (25 mg, 0.15 mmol, 5 mol %), and oxalic acid (27 mg, 0.3 mmol, 10 mol %) were reacted in 2-butanone (12 mL) for 4 h at 50 °C. The reaction was concentrated under reduced pressure and purified by column chromatography (2% EtOAc:Hexane) to give (oxybis(ethane-1,1-diyl))dibenzene as a colorless oil **28** (114 mg, 2.25 mmol, 31%) as a 1:1 mixture of diastereomers. Spectroscopic data in accordance with the literature.^19^ (Oxybis(ethane-1,1-diyl))dibenzene (**28**) (1:1 mixture of diastereomers): ^1^H NMR (500 MHz, CDCl_3_) δ_H_ 7.24–7.47 (10H, m), 4.61 (1H, q, *J* 6.4), 4.33 (1H, q, *J* 6.5), 1.55 (3H, d, *J* 6.4), 1.47 (3H, d, *J* 6.5).

#### Racemization of (*S*)-2-Methyl-1-phenylpropanol (**17**)

Following General Procedure B, (*S*)-2-methyl-1-phenylpropanol **17** (>99:1 er, 45 mg, 0.30 mmol), 2-carboxyphenylboronic acid **7** (2.5 mg, 0.015 mmol, 5 mol %), and oxalic acid (2.7 mg, 0.03 mmol, 10 mol %) were reacted in 2-butanone (1.2 mL) for 2 h at room temperature. The reaction was concentrated under reduced pressure and purified by column chromatography (10% EtOAc:Hexane) to give *rac*-2-methyl-1-phenylpropanol **17** (32 mg, 0.21 mmol, 71%). Chiral HPLC analysis: Chiralpak AD-H (99.5:0.5 hexane:IPA, flow rate 1.0 mL min^–1^, 220 nm, 30 °C) *t*_R_ (*R*): 21.6 min, *t*_R_ (*S*): 24.1 min, 55:46 (*S*:*R*) er.

#### Racemization of (*S*)-2,2-Dimethyl-1-phenylpropanol (**18**)

Following General Procedure B, (*S*)-2,2-dimethyl-1-phenylpropanol **18** (>99:1 er, 60 mg, 0.37 mmol), 2-carboxyphenylboronic acid **7** (0.015 M, 1.21 mL, 18 μmol, 5 mol %), and oxalic acid (0.11 M, 329 μL, 37 μmol, 10 mol %) were reacted in 2-butanone (1.5 mL) for 1.5 h at 60 °C. The reaction was concentrated under reduced pressure and purified by column chromatography (10% EtOAc:Hexane) to give *rac*-2,2-dimethyl-1-phenylpropanol **18** (51.0 mg, 0.31 mmol, 85%). Chiral HPLC analysis: Chiralpak OD-H (95:5 hexane:IPA, flow rate 1.0 mL min^–1^, 211 nm, 30 °C) *t*_R_ (*S*): 6.3 min, *t*_R_ (*R*): 8.9 min, 52:48 (*S*:*R*) er.

#### Attempted Racemization of 2,2-Dimethyl-1-(4-(trifluoromethyl)phenyl)propan-1-ol (**19**)

Following General Procedure B, (*S*)-2,2-dimethyl-1-(4-(trifluoromethyl))propan-1-ol **19** (97:3 er, 20 mg, 0.06 mmol), 2-carboxyphenylboronic acid **7** (0.015 M, 0.27 mL, 4.3 μmol, 5 mol %), and oxalic acid (0.11 M, 0.08 mL, 8.6 μmol, 10 mol %) were reacted in 2-butanone (0.35 mL) for 2 h at 75 °C. The reaction was then diluted with ether, washed with 1 M NaOH, brine, dried with MgSO_4_, and filtered. The resulting (*S*)-2,2-dimethyl-1-(4-(trifluoromethyl)phenyl)propan-1-ol **19** was analyzed by HPLC and showed no erosion of enantioenrichment. Chiral HPLC analysis: Chiralpak OJ-H (99:1 hexane:IPA, flow rate 1.0 mL min^–1^, 220 nm, 30 °C) *t*_R_ (*S*): 9.1 min, *t*_R_ (*R*): 10.0 min, 97:3 (*S*:*R*) er.

#### Racemization of 1-(4-Chlorophenyl)-2,2-dimethylpropan-1-ol (**20**)

Following General Procedure B, (*S*)-1-(4-chlorophenyl)-2,2-dimethylpropan-1-ol **20** (98:2 er, 37 mg, 0.19 mmol), 2-carboxyphenylboronic acid **7** (0.015 M, 0.63 mL, 9.5 μmol, 5 mol %), and oxalic acid (0.11 M, 0.17 mL, 19 μmol, 10 mol %) were reacted in 2-butanone (0.75 mL) for 6 h at 60 °C. The reaction was concentrated under reduced pressure and purified by column chromatography (10% EtOAc:Hexane) to give 1-(4-chlorophenyl)-2,2-dimethylpropan-1-ol **20** (26.0 mg, 0.13 mmol, 70%). Chiral HPLC analysis: Chiralpak IC (99.8:0.2 hexane:IPA, flow rate 1.0 mL min^–1^, 211 nm, 30 °C) *t*_R_ (*S*): 7.7 min, *t*_R_ (*R*): 7.2 min, 73:27 (*S*:*R*) er.

#### Racemization of 2,2-Dimethyl-1-(*p*-tolyl)propan-1-ol (**21**)

Following General Procedure B, (*S*)-2,2-dimethyl-1-(*p*-tolyl)propan-1-ol **21** (>99:1 er, 77 mg, 0.43 mmol), 2-carboxyphenylboronic acid **7** (3.88 mg, 22 μmol, 5 mol %), and oxalic acid (3.58 mg, 43 μmol, 10 mol %) were reacted in 2-butanone (1.7 mL) for 2 h at 40 °C. The reaction was concentrated under reduced pressure and purified by column chromatography (10% EtOAc:Hexane) to give *rac*-2,2-dimethyl-1-(*p*-tolyl)propan-1-ol **21** (68.0 mg, 0.38 mmol, 88%). Chiral HPLC analysis: Chiralpak OJ-H (98:2 hexane:IPA, flow rate 1.0 mL min^–1^, 220 nm, 30 °C) *t*_R_ (*S*): 6.8 min, *t*_R_ (*R*): 7.1 min, 54:46 (*S*:*R*) er.

#### Racemization of 2,2-Dimethyl-1-(naphthalen-2-yl)propan-1-ol (**22**)

Following General Procedure B, (*S*)-2,2-dimethyl-1-(naphthalen-2-yl)propan-1-ol **22** (97:3 er, 20 mg, 0.093 mmol), 2-carboxyphenylboronic acid **7** (0.015 M, 0.31 mL, 4.7 μmol, 5 mol %), and oxalic acid (0.11 M, 0.09 mL, 9.3 μmol, 10 mol %) were reacted in 2-butanone (0.4 mL) for 2 h at 40 °C. The reaction was concentrated under reduced pressure and purified by column chromatography (10% EtOAc:Hexane) to give *rac*-2,2-dimethyl-1-(naphthalen-2-yl)propan-1-ol **22** (15.0 mg, 0.070 mmol, 75%). Chiral HPLC analysis: Chiralpak OJ-H (99:1 hexane:IPA, flow rate 1.0 mL min^–1^, 220 nm, 30 °C) *t*_R_ (*S*): 19.4 min, *t*_R_ (*R*): 24.8 min, 56:44 (*S*:*R*) er.

#### Racemization of 1-(4-Methoxyphenyl)-2,2-dimethylpropan-1-ol (**23**)

Following General Procedure B, (*S*)-1-(4-methoxyphenyl)-2,2,-dimethylpropan-1-ol **23** (>99:1 er, 37 mg, 0.19 mmol), 2-carboxyphenylboronic acid **7** (0.015 M, 0.63 mL, 9.5 μmol, 5 mol %), and oxalic acid (0.11 M, 0.17 mL, 19 μmol, 10 mol %) were reacted in MeCN (0.76 mL) for 2 h at 0 °C. The reaction was concentrated under reduced pressure and purified by column chromatography (10% EtOAc:Hexane) to give *rac*-1-(4-methoxyphenyl)-2,2-dimethylpropan-1-ol **23** (29.0 mg, 0.15 mmol, 78%). Chiral HPLC analysis: Chiralpak AD-H (99:1 hexane:IPA, flow rate 1.0 mL min^–1^, 220 nm, 30 °C) *t*_R_ (*R*): 23.6 min, *t*_R_ (*S*): 25.7 min, 67:33 (*S*:*R*) er.

#### Racemization of 4-Phenylbut-3-yn-2-ol (**24**)

Following General Procedure B, (*S*)-4-phenylbut-3-yn-2-ol **24** (>99:1 er, 113.0 mg, 0.77 mmol), 2-carboxyphenylboronic acid **7** (6.4 mg, 0.039 mmol, 5 mol %), and oxalic acid (7 mg, 0.077 mmol, 10 mol %) were reacted in 2-butanone (3 mL) for 2 h at 60 °C. The reaction was concentrated under reduced pressure and purified by column chromatography (10% EtOAc:Hexane) to give *rac*-4-phenylbut-3-yn-2-ol **24** (67 mg, 0.48 mmol, 62%) and (oxybis(but-1-yne-3,1-diyl))dibenzene **S26** (36 mg, 0.25 mmol, 32%). Spectroscopic data in accordance with the literature.^20^ 4-Phenylbut-3-yn-2-ol **24**: Chiral HPLC analysis: Chiralpak OD-H (95:5 hexane:IPA, flow rate 1.0 mL min^–1^, 254 nm, 30 °C) *t*_R_ (*S*): 28.5 min, *t*_R_ (*R*): 11.3 min, 50:50 (*S*:*R*) er. (Oxybis(but-1-yne-3,1-diyl))dibenzene **S26** (1:1 mix of diastereomers): ^1^H NMR (400 MHz, CDCl_3_) δ_H_ 7.41–7.51 (4H, m), 7.25–7.35 (6H, m), 4.86 (1 H, q, *J* 6.6), 4.73 (1H, q, *J* 6.6), 1.60 (3H, d, *J* 2.4), 1.59 (3H, d, *J* 2.4).

#### Racemization of (*E*)-4-Phenylbut-3-en-2-ol (**25**)

Following General Procedure B, (*S*,*E*)-4-phenylbut-3-en-2-ol **25** (95:5 er, 60 mg, 0.40 mmol), 2-carboxyphenylboronic acid **7** (3.4 mg, 20 μmol, 5 mol %), and oxalic acid (3.6 mg, 40 μmol, 10 mol %) were reacted in 2-butanone (1.6 mL) for 2 h at room temperature. The reaction was concentrated under reduced pressure and purified by column chromatography (20% EtOAc:Hexane) to give *rac-*(*E*)-4-phenylbut-3-en-2-ol **25** (13.2 mg, 0.09 mmol, 22%) and ((1*E*,1′*E*)-oxybis(but-1-ene-3,1-diyl))dibenzene **S27** (37 mg, 0.25 mmol, 62%, ∼ 2.6:1 dr). Spectroscopic data in accordance with the literature.^21^ (*E*)-4-Phenylbut-3-en-2-ol **25**: Chiral HPLC analysis: Chiralpak OD-H (95:5 hexane:IPA, flow rate 1.0 mL min^–1^, 220 nm, 30 °C) *t*_R_ (*S*): 25.0 min, *t*_R_ (*R*): 15.1 min, 50:50 (*S*:*R*) er. ((1*E*,1′*E*)-Oxybis(but-1-ene-3,1-diyl))dibenzene **S27** (2.6:1 mixture of diastereomers): ^1^H NMR (400 MHz, CDCl_3_) δ_H_ 7.20–7.45 (5H, m), 6.55 (1H, d, *J* 16.0, minor diastereomer), 6.52 (1H, d, *J* 15.9, major diastereomer), 6.21 (1H, dd, *J* 16.0, 7.0, minor diastereomer), 6.14 (1H, dd, *J* 16.0, 7.8, major diastereomer), 4.13–4.31 (1H, m), 1.37 (3H, m).

#### Epimerization of Podophyllotoxin (**26**) to *epi*-Podophyllotoxin (**27**)

Following General Procedure B, podophyllotoxin **26** (>20:1 dr, 207 mg, 0.5 mmol), 2-carboxyphenylboronic acid **7** (4.1 mg, 0.025 mmol, 5 mol %), and oxalic acid (4.5 mg, 0.05 mmol, 10 mol %) were reacted in 2-butanone (2 mL) for 2 h at room temperature. The reaction was concentrated under reduced pressure and purified by column chromatography (50% EtOAc:Hexane) to give podophyllotoxin **26** (60 mg, 0.15 mmol, 30%) and *epi*-podophyllotoxin **27** (33 mg, 0.08 mmol, 16%) and (76 mg, 0.19 mmol, 37%, 2:1 dr 26:27) for an overall yield of 81% 60:40 dr. Spectroscopic data in accordance with the literature.^22^ Podophyllotoxin **26**: ^1^H NMR (400 MHz, CDCl_3_) δ_H_ 7.11 (1 H, d, *J* 0.8), 6.51 (1H, s), 6.37 (2H, s), 5.98 (2H, dd, *J* 8.2, 1.3), 4.78 (1H, t, *J* 8.7), 4.57–4.66 (2H, m), 4.10 (1H, dd, *J* 9.9, 8.7), 3.81 (3 H, s), 3.76 (6 H, s), 2.69–2.89 (2H, m), 1.98 (1H, dd, *J* 8.3, 0.8). *epi*-Podophyllotoxin **27**: ^1^H NMR (500 MHz, CDCl_3_) δ_H_ 6.87 (1H, s), 6.55 (1H, s), 6.28 (2H, s), 5.98 (2H, dd, *J* 12.4, 1.4), 4.86 (1H, t, *J* 3.9), 4.61 (1H, d, *J* 5.2), 4.31–4.42 (2H, m), 3.80 (3H, s), 3.74 (6H, s), 3.27 (1H, dd, *J* 14.1, 5.2), 2.83 (1H, tdt, *J* 11.0, 7.7, 3.3), 1.82 (1H, d, *J* 4.3).

## Supporting Information Available

Full experimental procedures, characterization data, NMR spectra, and HPLC chromatograms (PDF)

## Notes

Research data supporting this publication can be accessed at DOI: 10.17630/32d1c9e3-d14e-4653-98a1-3a5f13cb408c.
