# Supplementary material for: Arylboronic Acid-Catalyzed Racemization of Secondary and Tertiary Alcohols
Source: J Org Chem. 2022 Sep 8;87(19):13367–74. doi: 10.1021/acs.joc.2c01602 (PMC9552221; doi:10.1021/acs.joc.2c01602)
Supplement: Supplementary file 1 — jo2c01602_si_001.pdf [file jo2c01602_si_001.pdf]

Supporting Information

Arylboronic Acid-Catalyzed Racemization of Secondary and Tertiary Alcohols

Gregory R. Boyce,*,†,‡ Stefania F. Musolino,† Jianing Yang,† Andrew D. Smith,*,† James E. Taylor*,§

†EaStCHEM, School of Chemistry, University of St Andrews, North Haugh, St Andrews, Fife, KY16 9ST, U.K.
‡Department of Chemistry and Physics, Florida Gulf Coast University, Fort Myers, Florida 33965, United States
§Department of Chemistry, University of Bath, Claverton Down, Bath, Somerset, BA2 7AY, U.K.

Email: ads10@st-andrews.ac.uk; jet21@bath.ac.uk; gboyce@fgcu.edu

Table of Contents

1. General Information S1
2. General Procedures S3
3. Synthesis of *N*-Protected Isatin Derivatives S4
4. Racemic Alcohol and Ester Synthesis and Kinetic Resolution S6
5. Racemization Kinetics at Varying Concentrations and Catalyst Loadings S31
6. Mechanistic Studies S31
7. References S36
8. Appendix I: NMR Spectra S37
9. Appendix II: HPLC Spectra S70

1. General Information

Reactions involving moisture sensitive reagents were carried out in flame-dried glassware under a nitrogen (N_2) atmosphere using standard vacuum line techniques and using anhydrous solvents. Anhydrous solvents (CH_2Cl_2 and toluene) were obtained from an anhydrous solvent system (purified using an alumina column, Mbraun SPS-800). All other reactions were performed in standard glassware with no precautions to exclude air or moisture. Solvents and commercial reagents were used as supplied without further purification unless otherwise stated. (2*S*,3*R*)-HyperBTM was prepared from a literature procedure.[1]

Room temperature (r.t.) refers to 20−25 °C. Temperatures of 0 °C and −78 °C were obtained using ice/water and CO_2(s)/acetone baths, respectively. Reflux conditions were obtained using a DrySyn, oil bath, or sand bath equipped with a contact thermometer.

Analytical thin layer chromatography was performed on pre-coated aluminum plates (Kieselgel 60 F_{254} silica). TLC visualization was carried out with ultraviolet light (254 nm), followed by staining with a 1% aqueous $KMnO_4$ solution. Manual column chromatography was performed in glass columns fitted with porosity 3 sintered discs over Kieselgel 60 silica using the solvent system stated. Automated chromatography was performed on a Biotage Isolera Four running Biotage OS578 with a UV/Vis detector using the method stated and cartridges filled with Kieselgel 60 silica.

Melting points were recorded on an Electrothermal 9100 melting point apparatus and are uncorrected.

Optical rotations $[\alpha]_D^{20}$ were measured on a PerkinElmer Model 341 polarimeter operating at the sodium D line with a 100 mm path cell at 20 °C.

HPLC analyses were obtained using either a Shimadzu HPLC consisting of a DGU-20A5 degassing unit, LC-20AT liquid chromatography pump, SIL-20AHT autosampler, CMB-20A communications bus module, SPD-M20A diode array detector and a CTO-20A column oven; or a Shimadzu HPLC consisting of a DGU-20A5R degassing unit, LC-20AD liquid chromatography pump, SIL-20AHT autosampler, SPD-20A UV/Vis detector and a CTO-20A column oven. Separation was achieved using DAICEL CHIRALCEL OD-H or DAICEL CHIRALPAK AD-H or AS-H columns. All HPLC traces of enantiomerically-enriched compounds were compared with authentic racemic spectra.

^{1}H, ^{13}C, ^{19}F nuclear magnetic resonance (NMR) spectra were acquired on either a Bruker Avance 300 (^{1}H 300 MHz), Bruker Avance II 400 (^{1}H 400 MHz; ^{13}C 101 MHz; ^{19}F 376 MHz) or a Bruker Avance II 500 (^{1}H 500 MHz; ^{13}C 126 MHz; ^{19}F 476 MHz) spectrometer at ambient temperature in the deuterated solvent stated. All chemical shifts are quoted in parts per million (ppm) and referenced to the residual solvent peak. All coupling constants, *J*, are quoted in Hz. Multiplicities are indicated by: s (singlet), d (doublet), t (triplet), q (quartet), and combinations thereof, and m (multiplet). The abbreviation Ar is used to denote aromatic, Ph to denote phenyl, Bn to denote benzyl, br to denote broad, and app to denote apparent.

Infrared spectra (ν_{max}) were recorded on a Shimadzu IRAffinity-1 Fourier transform IR spectrophotometer fitted with a Specac Quest ATR accessory (diamond puck). Spectra were recorded of either thin films or solids, with characteristic absorption wave numbers (max) reported in cm^{-1}.

High Resolution Mass spectrometry (HRMS) data were acquired by electrospray ionization time-of-flight (ESI-TOF) at the University of St Andrews.

2. General Procedures

General Procedure A: *Preparation of racemic alcohols*

A solution of *N*-protected isatin or aldehyde (1 equiv) was dissolved in anhydrous THF (0.2 M) under a N_2 atmosphere and cooled to –78 °C. A Grignard reagent (1.2 equiv) was added dropwise, and the solution stirred at –78 °C for 20 mins, then at 0 °C and monitored by TLC until the reaction was complete. The reaction mixture reaction was poured into saturated aqueous NH_4Cl (20 mL) and extracted with EtOAc (3 × 20 mL). The organic layers were combined, dried over $MgSO_4$, filtered, and concentrated *in vacuo*. The product was purified as specified.

General procedure B: *Esterification of alcohols using DMAP and acid anhydrides*

The anhydride (1.1 equiv) and DMAP (10 mol%) were added to a solution of alcohol (1 equiv) in CH_2Cl_2. *i*-Pr_2NEt (1 equiv) was added, and the reaction mixture was stirred at room temperature for 18 h. On completion, the mixture was diluted with EtOAc and washed sequentially with 1 M aqueous HCl (2×), saturated aqueous $NaHCO_3$ (2×) and brine then dried with $MgSO_4$, filtered, and concentrated *in vacuo*. The ester products were purified by flash column chromatography as specified.

General procedure C: *Acylative kinetic resolution of tertiary alcohols using HyperBTM and isobutyric anhydride.*

The (2*S*,3*R*)-HyperBTM (1-10 mol%) was added to a solution of alcohol (1 equiv) in the required solvent. The reaction was cooled to the required temperature and anhydride (0.6-0.7 equiv) and *i*-Pr_2NEt (0.6 equiv) were added. The reaction mixture was stirred for the required time. On completion, the mixture was diluted with EtOAc and washed sequentially with 1 M HCl aqueous (2×), saturated aqueous $NaHCO_3$ (2×) and brine then dried with $MgSO_4$, filtered, and concentrated *in vacuo*. The alcohol and ester were separated by flash column chromatography under the conditions stated and analyzed by chiral HPLC.

General Procedure D: *Racemization of tertiary oxindoles*

The appropriate alcohol (1 equiv.), boronic acid (5 mol%,) and oxalic acid (10 mol%), were added to a vial. If the reaction was performed on a small-scale, stock solutions (*vide infra*) of the two catalysts were used and the THF from the stock solution was removed *in vacuo* prior to the start of the reaction. The reactants were then dissolved in the required solvent (0.25 M) and the mixture was heated at 60 °C. The reaction was stirred for the required time and then filtered through a silica pad and concentrated under reduced pressure. The alcohol was analyzed by chiral HPLC and ^1H NMR.

General Procedure E: *Racemization of secondary alcohols*

The appropriate alcohol (1 equiv.), boronic acid (5 mol%) and oxalic acid (10 mol%) were added to a vial. If the reaction was performed on a small-scale, stock solutions (*vide infra*) of the two catalysts were used and the THF from the stock solution was removed *in vacuo* prior to the start of the reaction. The reactants were dissolved in the required solvent (0.25 M) and the mixture was heated to the required temperature for the described time. The reaction was diluted with Et_2O and washed sequentially with 1 M NaOH, brine, then dried with $MgSO_4$, filtered, and concentrated *in vacuo*. The reaction mixture was then filtered through a silica pad and concentrated under reduced pressure. The alcohol was analyzed by chiral HPLC and ^{1}H NMR.

Preparation of 2-Carboxyphenylboronic Acid Stock Solution (0.015 M)

Boronic acid (5 mg, 0.03 mmol) and THF (1 mL) were added to a 2 mL volumetric flask. Once the mixture was homogeneous (after sonication) THF was added until the total volume of the mixture had reached 2 mL.

Preparation of Oxalic Acid Stock Solution (0.11 M)

Oxalic acid (20 mg, 0.22 mmol) and THF (1 mL) were placed in a 2 mL volumetric flask. Once the mixture was homogeneous (after sonication) THF was added until the total volume of the mixture had reached 2 mL.

3. Synthesis of *N*-Protected Isatin Derivatives

1-Benzylindoline-2,3-dione (S1)

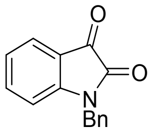

In a flame dried flask, sodium hydride (0.84 g, 0.21 mmol) was added to a solution of isatin (2.94 g, 20 mmol) in anhydrous dimethylformamide (65 mL) and the mixture was stirred for 10 minutes at 0 °C under a nitrogen atmosphere. Benzyl bromide (2.6 mL, 2.2 mmol) was added dropwise over 10 minutes. Once the addition was completed, the solution was stirred for further 30 minutes at room temperature. Water (80 mL) was added, the orange precipitate was collected via filtration, and the residue was washed by water (200 mL). The crude product was dissolved in CH_2Cl_2 (100 mL) and washed with brine (100 mL). The organic layer was dried ($MgSO_4$) and concentrated *in vacuo* to give 1-benzylindoline-2,3-dione **S1** (3.93 g, 16.6 mmol, 83%) as an orange solid with spectroscopic data in accordance with the literature.[2]

m.p. 125−127 °C {Lit.[2] 126-127 °C}; **^{1}H NMR** (400 MHz, $CDCl_3$) δ_H: 7.62 (1H, d, *J* 7.0), 7.48 (1H, td, *J* 15.1 1.4), 7.28−7.39 (5H, m), 7.09 (1H, td, *J* 15.5 0.8), 6.77 (1H, d, *J* 8.3), 4.94 (2H, s).

1-Methylindoline-2,3-dione (S2)

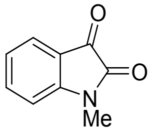

Sodium hydride (60% dispersion in mineral oil, 1.10 g, 27.5 mmol) was added portionwise to a solution of isatin (3.67 g, 25.0 mmol) in anhydrous dimethylformamide (50 mL) at 0 °C under a nitrogen atmosphere, and the reaction stirred for 15 minutes. Methyl iodine (1.71 mL, 27.5 mmol) was added dropwise, and the solution was stirred for 3 h, gradually warming to room temperature. Water (60 mL) was added with caution. The mixture was extracted with CH_2Cl_2 (3 x 50 mL), and the combined organics were washed with H_2O (2 × 50 mL), dried (Na_2SO_4), filtered and concentrated *in vacuo*. The residue was recrystallised from EtOAc/hexane to give 1-methylindoline-2,3-dione **S2** as red solid (2.94 g, 18.2 mmol, 73%) with spectroscopic data in accordance with the literature.[2]

m.p. 121−123 °C {Lit.[3] 124−125 °C}; **^{1}H NMR** (400 MHz, $CDCl_3$) δ_H: 7.57−7.64 (2H, m), 7.13 (1H, td, *J* 7.6, 0.9), 6.90 (1H, dt, *J* 7.9, 0.8), 3.26 (3H, s).

1-Allylindoline-2,3-dione (S3)

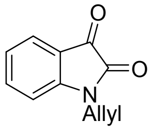

Cesium carbonate (8.96 g, 27.5 mmol) was added to a solution of isatin (3.68 g, 25 mmol) in anhydrous dimethylformamide (50 mL) at room temperature under a nitrogen atmosphere, and the reaction stirred for 30 minutes. Allyl bromide (2.60 mL, 30.0 mmol) was added, and the reaction stirred for 21 hours. EtOAc (100 mL) and water (100 mL) were added, and the layers separated. The aqueous layer was extracted using EtOAc (3 × 50 mL) and the combined organic fractions washed sequentially with water (2 × 100 mL) and brine (2 × 100 mL). The organic layer was dried ($MgSO_4$), filtered and concentrated *in vacuo* to give a red solid, which was dissolved in ethanol (50 mL), and then concentrated *in vacuo*. This process was repeated three times to remove residual dimethylformamide to give 1-allylindoline-2,3-dione **S3** as a red solid (3.98 g, 21.3 mmol, 85%) with spectroscopic data in accordance with the literature.[2]

m.p. 86−89 °C {Lit.[3] 85−87 °C}; **^{1}H NMR** (400 MHz, $CDCl_3$) δ_H: 7.50−7.65 (2H, m), 7.12 (1H, t, *J* 7.5), 6.89 (1H, d, *J* 7.9), 5.85 (1H, ddt, *J* 17.2, 10.5, 5.4), 5.28−5.35 (2H, m), 4.37 (2H, d, *J* 5.3).

1-Benzyl-5-methylindoline-2,3-dione (S4)

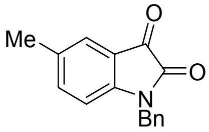

Sodium hydride (60% dispersion in mineral oil, 0.21 g, 5.25 mmol) was added portionwise to a solution of 5-methylisatin (806 mg, 5 mmol) in anhydrous dimethylformamide (25 mL) at 0 °C under a nitrogen atmosphere, and the reaction was stirred for 15 minutes. Benzyl bromide (0.65 mL, 5.5 mmol) was added dropwise at 0 °C and the solution was stirred for 1 h while warming to room temperature. Water (100 mL) was added, and the resulting red precipitate was filtered and washed with water (~200 mL). The precipitate was dissolved in CH_2Cl_2 (100 mL) and washed with brine (100 mL). The organic layer was separated, dried ($MgSO_4$), filtered and concentrated to dryness *in vacuo* to give 1-benzyl-5-methylindoline-2,3-dione **S4** as an orange-red solid (1.00 g, 3.98 mmol, 80%) with spectroscopic data in accordance with the literature.[2]

m.p. 136–140 °C {Lit.[2] 138–140 °C}; **^{1}H NMR** (400 MHz, $CDCl_3$) δ_H: 7.26–7.42 (7H, m), 6.65 (1H, d, *J* 8.1), 4.91 (2H, s), 2.30 (3H, s).

4. Racemic Alcohol and Ester Synthesis and Kinetic Resolution

1-Benzyl-3-hydroxy-3-phenylindolin-2-one (1)

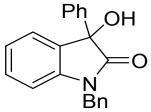

Following **General Procedure A**, 1-benzylindoline-2,3-dione **S1** (3.57 g, 15 mmol) and phenylmagnesium bromide (3.0 M, 6 mL, 18.0 mmol) were reacted in anhydrous THF (125 mL) under N_2 to give the crude product, which was purified by Biotage® Isolera 4 chromatography to give a yellow solid. The product was further purified by recrystallisation by dissolving in a minimal amount of CH_2Cl_2 (~30 mL), layering with hexane (~100 mL), and cooling in a freezer at −28 °C overnight to give 1-benzyl-3-hydroxy-3-phenylindolin-2-one **1** as a white solid (2.76 g, 8.7 mmol, 58%) with spectroscopic data in accordance with the literature.[2]

m.p. 139–142 °C {Lit.[2] 140–142 °C}; **^{1}H NMR** (400 MHz, $CDCl_3$) δ_H: 7.40–7.44 (2H, m), 7.27–7.38 (9H, m), 7.23 (1H, td, *J* 7.8 1.3), 7.05 (1H, td, *J* 7.5 0.7), 6.79 (1H, d, *J* 7.8), 5.06 (1H, d, *J* 15.6), 4.84 (1H, d, *J* 15.6), 3.20 (1H, br s).

1-Benzyl-2-oxo-3-phenylindolin-3-yl isobutyrate (S5): The racemic ester was prepared in a previous report from this group.[2]

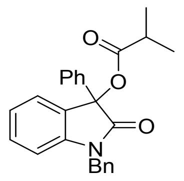

^{1}H NMR (400 MHz, $CDCl_3$) δ_H: 7.27–7.38 (11H, m), 7.23 (1H, td, *J* 6.0 1.3), 7.05 (1H, td, *J* 7.5 1.0), 6.72 (1H, d, *J* 7.9), 5.02 (1H, d, *J* 15.9), 1.23 (3H, d, *J* 7.0), 4.85 (1H, d, *J* 15.9), 2.74 (1H, tt, *J* 7.0), 1.26 (3H, d, *J* 7.0).

Kinetic Resolution of 1-Benzyl-3-hydroxy-3-phenylindolin-2-one (1)

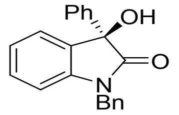

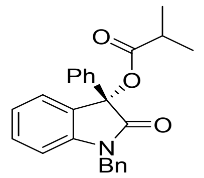

Following **General Procedure C**, 1-benzyl-3-hydroxy-3-phenylindolin-2-one **1** (2.73 g, 8.6 mmol), isobutyric anhydride (1.3 mL, 7.8 mmol), (*2S,3R*)-HyperBTM (45 mg, 0.145 mmol) and *i*-Pr_2NEt (0.84 mL, 5.2 mmol) were reacted in $CHCl_3$ (55 mL) at room temperature for 40 h. The reaction was concentrated under reduced pressure and purified by Biotage® Isolera 4 to give (*S*)-1-benzyl-2-oxo-3-phenylindolin-3-yl isobutyrate **S5** (1.88 g, 4.9 mmol, 57%) and (*R*)-1-benzyl-3-hydroxy-3-phenylindolin-2-one **1** (0.89 g, 2.8 mmol, 33%).

***(R)*-1-Benzyl-3-hydroxy-3-phenylindolin-2-one 1**: $[\alpha]_D^{20}$ +68 (*c* 1.0, $CHCl_3$) {Lit.[9] (99% ee) +55 (*c* 1.0, $CHCl_3$)}; **Chiral HPLC analysis**, Chiralpak AD-H (90:10 hexane:IPA, flow rate 1.25 mLmin^{-1}, 211 nm, 40 °C) t_R (*R*): 16.9 min, t_R (*S*): 21.1 min, >99:1 (*R*:*S*) er. ***(S)*-1-Benzyl-2-oxo-3-phenylindolin-3-yl isobutyrate S5**: $[\alpha]_D^{20}$ +77 (*c* 1.0, $CHCl_3$) {Lit.[9] (90% ee) +107 (*c* 1.0, $CHCl_3$)}; **Chiral HPLC analysis**, Chiralpak AD-H (90:10 hexane:IPA, flow rate 1.25 mLmin^{-1}, 211 nm, 40 °C) t_R (*R*): 10.0 min, t_R (*S*): 20.0 min, 77:23 (*S*:*R*) er.

3-Hydroxy-1-methyl-3-phenylindolin-2-one (8)

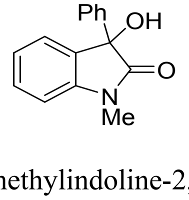

Following **General Procedure A**, 1-methylindoline-2,3-dione **S2** (1.00 g, 6.2 mmol) and phenylmagnesium bromide (3.0 M, 2.5 mL, 7.3 mmol) were reacted in anhydrous THF (60 mL) under N_2. The reaction was concentrated under reduced pressure and purified by column chromatography

(CH_2Cl_2:EtOAc 9:1) to give 1-benzyl-3-hydroxy-3-phenylindolin-2-one **8** as a colorless solid (0.83 g, 3.4 mmol, 55%) with spectroscopic data in accordance with the literature.[3]

m.p. 139–142 °C {Lit.[3] 123–125 °C}; **^{1}H NMR** (400 MHz, $CDCl_3$) δ_H: 7.26–7.42 (7H, m), 7.10 (1H, app td, *J* 7.5, 1.0), 6.92 (1H, app dt, *J* 7.9, 0.7), 3.41 (1H, s), 3.27 (3H, s).

1-Methyl-2-oxo-3-phenylindolin-3-yl isobutyrate (S6): The racemic ester was prepared in a previous report from this group.[3]

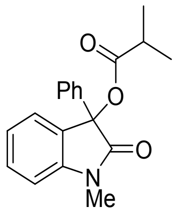

^{1}H NMR (400 MHz, $CDCl_3$) δ_H: 7.31–7.42 (6H, m), 7.20 (1H, ddd, *J* 7.3, 1.4, 0.5), 7.10 (1H, app td, *J* 7.5, 1.0), 6.91 (1H, app dt, *J* 7.8, 0.7), 3.23 (3H, s), 2.70 (1H, sept, *J* 7.0), 1.24 (3H, *J* 7.0), 1.18 (3H, d, *J* 7.0).

Kinetic Resolution of 3-Hydroxy-1-methyl-3-phenylindolin-2-one (8)

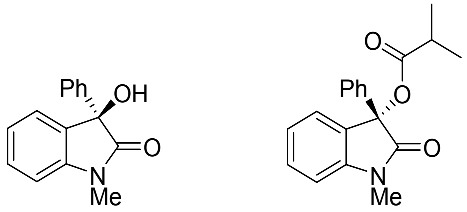

Following **General Procedure C**, 1-methyl-3-hydroxy-3-phenylindolin-2-one **8** (360 mg, 1.5 mmol), isobutyric anhydride (0.23 mL, 1.4 mmol), (*2S,3R*)-HyperBTM (8.0 mg, 0.025 mmol) and *i*-Pr_2NEt (0.16 mL, 1.0 mmol) were reacted in $CHCl_3$ (15 mL) at room temperature for 48 h. The reaction was concentrated under reduced pressure and purified by column chromatography (Petrol:EtOAc 3:1) to give (*S*)-1-methyl-2-oxo-3-phenylindolin-3-yl isobutyrate **S6** (201 mg, 0.65 mmol, 43%) and (*R*)-1-methyl-3-hydroxy-3-phenylindolin-2-one **8** (193 mg, 0.81 mmol, 54%).

***(R)*-1-Methyl-3-hydroxy-3-phenylindolin-2-one 8**: $[\alpha]_D^{20}$ +91 (*c* 1.0, $CHCl_3$) {Lit.[3] (*ent*, 98% ee) –77 (*c* 1.0, $CHCl_3$)}; **Chiral HPLC analysis**: Chiralpak AD-H (95:5 hexane:IPA, flow rate 1.0 mLmin^{-1}, 211 nm, 30 °C) t_R (*R*): 27.9 min, t_R (*S*): 31.0 min, >99:1 (*R*:*S*) er. ***(S)*-1-Methyl-2-oxo-3-phenylindolin-3-yl isobutyrate S6**: $[\alpha]_D^{20}$ +110 (*c* 1.0, $CHCl_3$) {Lit.[3] (*ent*, 88% ee) –149 (*c* 1.0, $CHCl_3$)}; **Chiral HPLC analysis**: Chiralpak AD-H (95:5 hexane:IPA, flow rate 1.0 mLmin^{-1}, 254 nm, 30 °C) t_R (*S*): 8.9 min, t_R (*R*): 12.2 min, 91:9 (*S*:*R*) er.

1-Allyl-3-hydroxy-3-phenylindolin-2-one (9)

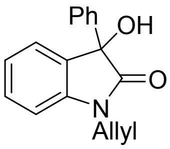

Following **General Procedure A**, 1-allylindoline-2,3-dione **S3** (1.00 g, 5.4 mmol) and phenylmagnesium bromide (3.0 M, 2.2 mL, 6.5 mmol) were reacted in anhydrous THF (60 mL) under N_2. The reaction was concentrated under reduced pressure and purified by column chromatography (CH_2Cl_2:EtOAc 9:1) to give 1-allyl-3-hydroxy-3-phenylindolin-2-one **9** as a white solid (1.24 g, 4.6 mmol, 85%) with spectroscopic data in accordance with the literature.[3]

m.p. 133−135 °C {Lit.[3] 132−134 °C}; **^{1}H NMR** (400 MHz, $CDCl_3$) δ_H: 7.38–7.43 (2H, m), 7.27–7.37 (6H, m), 7.08 (1H, td, *J* 7.5, 1.0), 6.91 (1H, dt, *J* 7.7, 0.8), 5.88 (1 H, ddt, *J* 17.2, 10.4, 5.3), 5.22–5.34 (2H, m), 4.48 (1H, ddt, *J* 16.3, 5.2, 1.7), 4.29 (1H, ddt, *J* 16.4, 5.3, 1.6).

1-Allyl-2-oxo-3-phenylindolin-3-yl isobutyrate (S7): The racemic ester was prepared in a previous report from this group.[3]

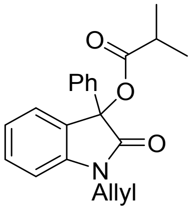

m.p. 82−83 °C {Lit.[3] 80−82 °C}; **^{1}H NMR** (400 MHz, $CDCl_3$) δ_H: 7.31−7.39 (6H, m), 7.20 (1H, ddd, *J* 7.4, 1.5, 0.6), 7.09 (1H, app td, *J* 7.4, 1.0), 6.91 (1H, app dt, *J* 7.9, 0.8), 5.85 (1H, ddt, *J* 17.2, 10.4, 5.2), 5.32 (1H, dq, *J* 17.2, 1.5), 5.23 (1H, dq, *J* 10.4, 1.5), 2.71 (1H, sept, *J* 7.0), 4.34–4.37 (2H, m), 1.23 (3H, d, *J* 7.0), 1.20 (3H, d, *J* 7.0).

Kinetic Resolution of 1-Allyl-3-hydroxy-3-phenylindolin-2-one (9)

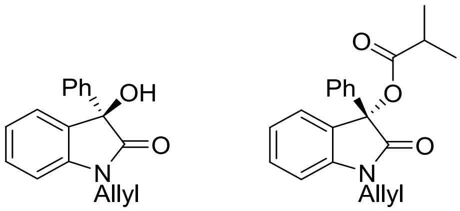

Following **General Procedure C**, 1-allyl-3-hydroxy-3-phenylindolin-2-one **9** (400 mg, 1.5 mmol), isobutyric anhydride (0.23 mL, 1.4 mmol), (*2S,3R*)-HyperBTM (8.0 mg, 0.025 mmol) and *i*-Pr_2NEt (0.16 mL, 1.0 mmol) were reacted in $CHCl_3$ (15 mL) at room temperature for 24 h. The reaction was concentrated under reduced pressure and purified by column chromatography (Petrol:EtOAc 3:1) to give (*S*)-1-allyl-2-

oxo-3-phenylindolin-3-yl isobutyrate **S7** (216 mg, 0.62 mmol, 41%) and (*R*)-1-allyl-3-hydroxy-3-phenylindolin-2-one **9** (92 mg, 0.38 mmol, 25%).

***(R)*-1-Allyl-3-hydroxy-3-phenylindolin-2-one 9**: $[\alpha]_D^{20}$ +68 (*c* 1.0, $CHCl_3$) {Lit.[3] (*ent*, 99% ee) –83 (*c* 1.0, $CHCl_3$)}; **Chiral HPLC analysis**: Chiralpak OD-H (95:5 hexane:IPA, flow rate 1.0 mLmin^{-1}, 211 nm, 30 °C) t_R (*S*): 13.8 min, t_R (*R*): 15.7 min, 97:3 (*R*:*S*) er. ***(S)*-1-Allyl-2-oxo-3-phenylindolin-3-yl isobutyrate S7**: $[\alpha]_D^{20}$ +148 (*c* 1.0, $CHCl_3$) {Lit.[32] (*ent*, 85% ee) –103 (*c* 1.0, $CHCl_3$)}; **Chiral HPLC analysis**: Chiralpak OD-H (99:1 hexane:IPA, flow rate 1.0 mLmin^{-1}, 211 nm, 30 °C) t_R (*R*): 11.1 min, t_R (*S*): 12.9 min, 98:2 (*S*:*R*) er.

1-Benzyl-3-hydroxy-5-methyl-3-phenylindolin-2-one (10)

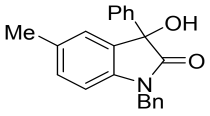

Following **General Procedure A**, 1-benzyl-5-methylindoline-2,3-dione **S4** (1.00 g, 4.0 mmol) and phenylmagnesium bromide (3.0 M, 1.6 mL, 4.8 mmol) were reacted in anhydrous THF (20 mL) under N_2. The reaction was concentrated under reduced pressure and purified by column chromatography (Petrol:EtOAc 3:1) to give 1-benzyl-3-hydroxy-5-methyl-3-phenylindolin-2-one **10** as a white solid (0.69 g, 2.1 mmol, 53%) with spectroscopic data in accordance with the literature.[2]

m.p. 157–159 °C {Lit.[2] 154–156 °C}; **^{1}H NMR** (400 MHz, $CDCl_3$) δ_H: 7.39–7.45 (2H, m), 7.27–7.39 (8H, m), 7.08–7.12 (1H, m), 6.99–7.04 (1H, m), 6.67 (1H, d, *J* 8.0), 5.04 (1H, d, *J* 15.7), 4.85 (1H, d, *J* 15.7), 3.97 (1H, br s), 2.25 (3H, s).

1-Benzyl-2-oxo-3-phenylindolin-3-yl isobutyrate (S8): The racemic ester was prepared in a previous report from this group.[2]

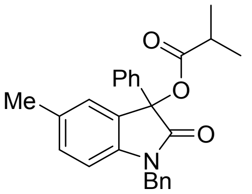

^{1}H NMR (400 MHz, $CDCl_3$) δ_H: 7.23–7.41 (10H, m), 6.99–7.07 (2H, m), 1.24 (3H, d, *J* 7.0), 6.60 (1H, d, *J* 7.9), 4.98 (1H, d, *J* 15.9), 4.84 (1H, d, *J* 15.9), 2.74 (1H, sept, *J* 7.0), 2.27 (3H, s), 1.27 (3H, d, *J* 7.0).

Kinetic Resolution of 1-Benzyl-3-hydroxy-5-methyl-3-phenylindolin-2-one (10)

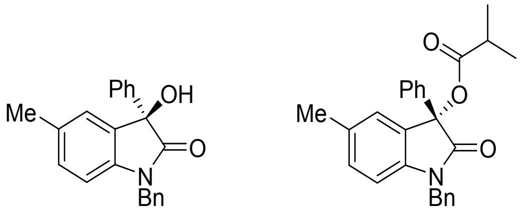

Following **General Procedure C**, 1-benzyl-3-hydroxy-5-methyl-3-phenylindolin-2-one **10** (660 mg, 2.0 mmol), isobutyric anhydride (0.31 mL, 1.9 mmol), (*2S,3R*)-HyperBTM (11.2 mg, 0.035 mmol) and *i*-Pr_2NEt (0.21 mL, 1.3 mmol) were reacted in $CHCl_3$ (15 mL) at room temperature for 48 h. The reaction was concentrated under reduced pressure and purified by column chromatography (Petrol:EtOAc 3:1) to give (*S*)-1-benzyl-5-methyl-2-oxo-3-phenylindolin-3-yl isobutyrate **S8** as a colourless oil (377 mg, 0.94 mmol, 47%) and (*R*)-1-benzyl-3-hydroxy-5-methyl-3-phenylindolin-2-one **10** (132 mg, 0.4 mmol, 20%).

***(R)*-1-Benzyl-3-hydroxy-3-phenylindolin-2-one 10**: $[\alpha]_D^{20}$ –47 (*c* 1.0, $CHCl_3$) {Lit.[2] (*ent*, 82% ee) +24 (*c* 1.0, $CHCl_3$)}; **Chiral HPLC analysis**: Chiralpak AD-H (90:10 hexane:IPA, flow rate 1.5 $mLmin^{-1}$, 211 nm, 40 °C) t_R (*R*): 12.3 min, t_R (*S*): 15.8 min, >99:1 (*R*:*S*) er. ***(S)*-1-Benzyl-2-oxo-3-phenylindolin-3-yl isobutyrate S8**: $[\alpha]_D^{20}$ –89 (*c* 1.0, $CHCl_3$) {Lit.[9] (*ent*, 94% ee) +107 (*c* 1.0, $CHCl_3$)}; **Chiral HPLC analysis**: Chiralpak AD-H (95:5 hexane:IPA, flow rate 1.5 $mLmin^{-1}$, 211 nm, 40 °C) t_R (*S*): 11.2 min, t_R (*R*): 25.0 min, 90:10 (*S*:*R*) er.

1-Benzyl-3-hydroxy-3-(naphthalen-2-yl)indolin-2-one (11)

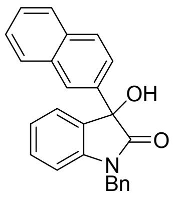

Magnesium turnings (362 mg, 15.0 mmol) were added to anhydrous THF (15 mL) at room temperature under a N_2 atmosphere. 2-Bromonaphthalene (0.21 g, 1.0 mmol) in anhydrous THF (0.5 mL) was added followed by a small iodine crystal. Upon the iodine colour disappearing, the remaining 2-bromonaphthalene solution (1.86 g, 9 mmol, in 4.5 mL anhydrous THF) was added and reacted for a further 3 h.

Following **General Procedure A**, 1-benzylindoline-2,3-dione **S1** (1.18 g, 5.0 mmol) and naphthalen-2-ylmagnesium bromide (prepared above, max. 10 mmol) were reacted in anhydrous THF (25 mL) under N_2. The reaction was concentrated under reduced pressure and purified by column chromatography (Petrol:EtOAc 3:1) to give a yellow solid. The product was further purified by recrystallisation by dissolving in a minimal amount of CH_2Cl_2 (~15 mL), layering with hexane (~100 mL), and cooling in a

freezer at −28 °C overnight to give 1-benzyl-3-hydroxy-3-(naphthalen-2-yl)indolin-2-one **11** as a white solid (1.12 g, 3.1 mmol, 61%) with spectroscopic data in accordance with the literature.[6]

m.p. 188−190 °C; **^{1}H NMR** (400 MHz, $CDCl_3$) δ_H: 7.95 (1H, d, *J* 1.8), 7.77−7.85 (3H, m), 7.46−7.50 (2H, m), 7.41 (1H, dd, *J* 8.6 1.8), 7.28−7.38 (6H, m), 7.25 (1H, td, *J* 8.0 1.2), 7.05 (1H, td, *J* 7.5 0.7), 6.84 (1H, d, *J* 7.6), 5.10 (1H, d, *J* 15.7), 4.88 (1H, d, *J* 15.7), 3.41 (1H, s).

1-Benzyl-2-oxo-3-(naphthalen-2-yl)indolin-3-yl isobutyrate (S9)

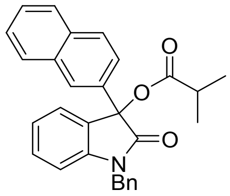

Following **General Procedure B**, 1-benzyl-3-hydroxy-3-(naphthalen-2-yl)indolin-2-one **11** (82 mg, 0.23 mmol), isobutyric anhydride (44 µL, 0.25 mmol), DMAP (2.2 mg, 0.023 mmol, 10 mol%) and *i*-Pr_2NEt (38 µL, 0.23 mmol) were reacted in CH_2Cl_2 (10 mL). The reaction was concentrated under reduced pressure and purified by column chromatography (Petrol:EtOAc 2:1) to give 1-benzyl-2-oxo-3-(naphthalen-2-yl)indolin-3-yl isobutyrate **S9** as a red solid (80 mg, 0.18 mmol, 80%).

m.p. 110−112 °C; **^{1}H NMR** (400 MHz, $CDCl_3$) δ_H: 7.85 (2H, m), 7.77 (1H, m), 7.72 (1H, m), 7.61 (1H, dd, *J* 8.8 2.0), 7.44-7.53 (2H, m), 7.21-7.37 (7H, m), 7.09 (1H, td, *J* 7.6, 0.8), 6.78 (1H, td, *J* 7.7, 0.8), 5.05 (1H, d, *J* 16.4), 4.89 (1H, d, *J* 16.4), 2.81 (1H, sept, *J* 7.1), 1.31 (3H, d, *J* 7.1), 1.27 (3H, d, *J* 7.1); **^{13}C{^{1}H} NMR** (101 MHz, $CDCl_3$) δ_C: 175.2, 174.2, 143.8, 135.8, 134.3, 133.5, 133.0, 130.2, 128.9, 128.8, 128.5, 127.7, 127.7, 127.4, 126.8, 126.5, 125.7, 124.0, 123.9, 123.3, 109.9, 81.1, 44.4, 34.0, 19.0, 18.8; **IR** ν_{max} (solid) 2980 (C–H), 1739, 1724 (C=O), 1614 (C=C), 1487, 1467, 1342, 1140 cm^{-1}; **HRMS** (NSI$^+$) calculated for $C_{29}H_{25}NO_3Na^+$ $[M+Na]^+$ requires 458.1727; found 458.1713 (−3.0 ppm).

Kinetic Resolution of 1-Benzyl-3-hydroxy-3-(naphthalen-2-yl)indolin-2-one (11)

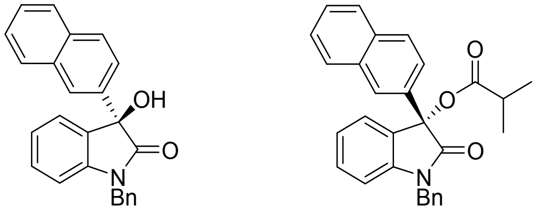

Following **General Procedure C**, 1-benzyl-3-hydroxy-3-(naphthalen-2-yl)indolin-2-one **11** (1.12 g, 3.0 mmol), isobutyric anhydride (0.38 mL, 2.1 mmol), (*2S,3R*)-HyperBTM (10.0 mg, 0.03 mmol) and *i*-Pr_2NEt (0.31 mL, 1.8 mmol) were reacted in $CHCl_3$ (25 mL) at room temperature for 24 h. The reaction was concentrated under reduced pressure and purified by column chromatography (Petrol:EtOAc 3:1) to give

(*S*)-1-benzyl-2-oxo-3-(naphthalen-2-yl)indolin-3-yl isobutyrate **S9** (335 mg, 0.77 mmol, 26%) and (*R*)-1-benzyl-3-hydroxy-3-(naphthalen-2yl)indolin-2-one **11** (313 mg, 0.86 mmol, 28%).

***(R)*-1-Benzyl-3-hydroxy-3-(naphthalen-2-yl)indolin-2-one 11**: $[\alpha]_D^{20}$ +39 (*c* 1.0, $CHCl_3$) {Lit.[6] (74% ee) +32.0 (*c* 1.0, $CHCl_3$)}; **Chiral HPLC analysis**: Chiralpak IA (70:30 hexane:IPA, flow rate 0.5 mLmin^{-1}, 211 nm, 30 °C) t_R (*R*): 24.4 min, t (*S*): 30.3 min, >99:1 (*R*:*S*) er. ***(S)*-1-Benzyl-2-oxo-3-(naphthalen-2-yl)indolin-3-yl isobutyrate S9**:$[\alpha]_D^{20}$ +86 (*c* 1.5, $CHCl_3$); **Chiral HPLC analysis**: Chiralpak IA (70:30 hexane:IPA, flow rate 1.0 mLmin^{-1}, 211 nm, 30 °C) t_R (*S*): 14.2 min, t_R (*R*): 31.2 min, 84:16 (*S*:*R*) er.

1-Benzyl-3-hydroxy-3-(4-methoxyphenyl)indolin-2-one (12)

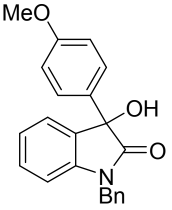

Following **General Procedure A**, 1-benzylindoline-2,3-dione **S1** (1.18 g, 5.0 mmol) and 4-methoxyphenylmagnesium bromide (1.0 M, 6 mL, 6.0 mmol) were reacted in anhydrous THF (25 mL) under N_2. The reaction was concentrated under reduced pressure and purified by column chromatography (Petrol:EtOAc 3:1) to give a yellow solid. The product was further purified by recrystallisation by dissolving in a minimal amount of CH_2Cl_2 (~30 mL), layering with hexane (~100 mL), and cooling in a freezer at −28 °C overnight to give 1-benzyl-3-hydroxy-3-(4-methoxyphenyl)indolin-2-one **12** as a yellow solid. (1.52 g, 4.4 mmol, 88%) with spectroscopic data in accordance with the literature.[4]

m.p. 161−163 °C {Lit.[2] 159–160 °C}; **^{1}H NMR** (400 MHz, $CDCl_3$) δ_H: 7.26–7.39 (8H, m), 7.23 (1H, td, *J* 7.8 1.3), 7.06 (1H, td, *J* 7.5 1.0), 6.85–6.90 (2H, m), 6.78 (1H, d, *J* 7.8), 5.04 (1H, d, *J* 15.5), 4.83 (1H, d, *J* 15.5), 3.78 (3H, s).

1-Benzyl-2-oxo-3-(4-methoxyphenyl)indolin-3-yl isobutyrate (S10)

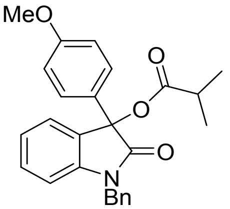

Following **General Procedure B**, 1-benzyl-3-hydroxy-3-(4-methoxyphenyl)indolin-2-on **12** (90 mg, 0.26 mmol), isobutyric anhydride (50 µL, 0.29 mmol), DMAP (3.2 mg, 0.026 mmol, 10 mol%) and *i*-Pr_2NEt (44 µL, 0.26 mmol) were reacted in CH_2Cl_2 (10 mL). The reaction was concentrated under reduced pressure

and purified by column chromatography (Petrol:EtOAc 2:1) to give 1-benzyl-2-oxo-3-(4-methoxyphenyl)indolin-3-yl isobutyrate **S10** as a yellow solid (90.4 mg, 0.22 mmol, 85%).

m.p. 81–84 °C; ^{1}**H NMR** (400 MHz, $CDCl_3$) δ_H: 7.19–7.35 (9H, m), 7.06 (1H, t, *J* 7.4), 6.88 (2H, d, *J* 8.9), 6.70 (1H, d, *J* 8.0), 5.00 (1H, d, *J* 15.9), 4.85 (1H, d, *J* 15.9), 3.80 (3H, s), 2.72 (1H, sept, *J* 7.0), 1.26 (3H, d, *J* 7.0), 1.22 (3H, d, *J* 7.0); ^{13}**C{^{1}H} NMR** (101 MHz, $CDCl_3$) δ_C: 175.3, 174.5, 160.2, 143.7, 135.8, 130.0, 128.9, 128.8, 128.4, 128.0, 127.6, 127.3, 123.9, 123.1, 114.1, 109.8, 80.7, 55.4, 44.3, 33.9, 19.0, 18.9; **IR** ν_{max} (solid) 2972 (C–H), 1716 (C=O), 1608 (C=C) cm^{-1}; **HRMS** (NSI^+) calculated for $C_{26}H_{25}NO_4Na^+$ $[M+Na]^+$ requires 438.1676; found 438.1659 (−3.9 ppm).

Kinetic Resolution of 1-Benzyl-3-hydroxy-3-(4-methoxyphenyl)indolin-2-one (12)

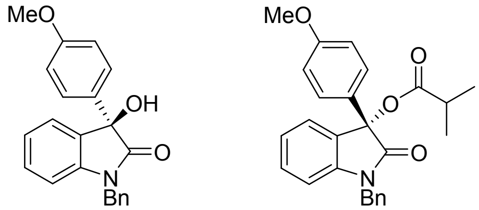

Following **General Procedure C**, 1-benzyl-3-hydroxy-3-(4-methoxyphenyl)indolin-2-one **12** (1.73 g, 5.0 mmol), isobutyric anhydride (0.61 mL, 3.5 mmol), (*2S,3R*)-HyperBTM (15.4 mg, 0.05 mmol) and *i*-Pr_2Net (0.50 mL, 3.0 mmol) were reacted in $CHCl_3$ (30 mL) at room temperature for 72 h. The reaction was concentrated under reduced pressure and purified by column chromatography (Petrol:EtOAc 3:1) to give (*S*)-1-benzyl-2-oxo-3-(4-methoxyphenyl)indolin-3-yl isobutyrate **S10** (579 mg, 1.39 mmol, 28%) and (*R*)-1-benzyl-3-hydroxy-3-(4-methoxyphenyl)indolin-2-one **12** (240 mg, 0.69 mmol, 14%).

***(R)*-1-Benzyl-3-hydroxy-3-(4-methoyphenyl)indolin-2-one 12**: $[\alpha]_D^{20}$ +10 (*c* 0.5, $CHCl_3$) {Lit.[2] (*ent*, 92% ee) −212 (*c* 0.1, $CHCl_3$)}; **Chiral HPLC analysis**: Chiralpak IC (80:20 hexane:IPA, flow rate 1.0 $mLmin^{-1}$, 211 nm, 30 °C) t_R (*R*): 15.9 min, t_R (*S*): 21.3 min, >99:1 (*R*:*S*) er. ***(S)*-1-Benzyl-2-oxo-3-(4-methoxyphenyl)indolin-3-yl isobutyrate S10**: $[\alpha]_D^{20}$ +90 (*c* 1.0, $CHCl_3$); **Chiral HPLC analysis**: Chiralpak IA (80:20 hexane:IPA, flow rate 1.0 $mLmin^{-1}$, 211 nm, 30 °C) t_R (*S*): 15.3 min, t_R (*R*): 19.7 min, 77:23 (*S*:*R*) er.

3-Allyl-1-benzyl-3-hydroxyindolin-2-one (13): The racemic oxindole was prepared in a previous report from this group.[2]

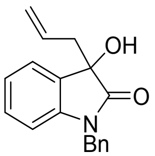

^{1}H NMR (500 MHz, $CDCl_3$) δ_H: 7.41 (2H, dd, *J* 7.4, 1.3), 7.20–7.31 (4H, m), 7.16 (2H, td, *J* 7.8, 1.3), 7.04 (2H, td, *J* 7.6, 1.0), 6.66 (2H, d, *J* 7.9), 5.58 (2H, dddd, *J* 16.6, 10.1, 8.4, 6.2), 5.12 (2H, dq, *J* 17.2, 1.5), 5.05 (2H, dd, *J* 10.2, 1.9), 4.99 (2H, d, *J* 15.7), 4.65 (2H, d, *J* 15.7), 4.32 (1H, s), 2.87 (2H, ddt, *J* 13.2, 6.3, 1.4), 2.77 (2H, dd, *J* 13.3, 8.5).

3-Allyl-1-benzyl-2-oxoindolin-3-yl isobutyrate (S11): The racemic ester was prepared in a previous report from this group.[2]

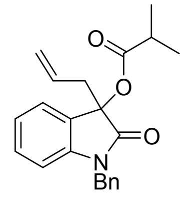

^{1}H NMR (500 MHz, $CDCl_3$) δ_H: 7.29–7.39 (4H, m), 7.22–7.29 (2H, m), 7.13–7.21 (2H, m), 6.99 (1H, td, J 7.5, 1.0), 6.64 (1H, dt, *J* 7.8, 0.8), 5.60 (1H, dddd, J 16.7, 10.2, 8.3, 6.2), 5.06–5.14 (2H, m), 5.04 (1H, d, J 15.9), 4.84 (1H, d, *J* 15.9), 2.88 (1H, ddt, *J* 13.4, 6.2, 1.4), 2.54–2.68 (2H, m), 1.16 (6H, dd, *J* 7.0, 2.5).

Kinetic Resolution of 3-Allyl-1-benzyl-3-hydroxyindolin-2-one (13)

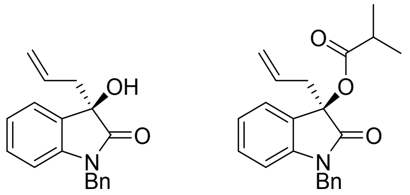

Following **General Procedure C**, 3-allyl-1-benzyl-3-hydroxyindolin-2-one **13** (500 mg, 1.79 mmol), isobutyric anhydride (0.20 mL, 1.25 mmol), (*2S,3R*)-HyperBTM (5.5 mg, 0.018 mmol) and *i*-Pr_2NEt (0.19 mL, 1.1 mmol) were reacted in $CHCl_3$ (7 mL) at room temperature for 24 h. The reaction was concentrated under reduced pressure and purified by column chromatography (30% EtOAc:Hexane) to give (*S*)-3-allyl-1-benzyl-2-oxoindolin-3-yl isobutyrate **S11** as a white solid (164 mg, 1.1 mmol, 61%) and (*R*)-3-allyl-1-benzyl-3-hydroxyindolin-2-one **13** as a colorless oil (164 mg, 0.59 mmol, 33%).

***(R)*- 3-Allyl-1-benzyl-3-hydroxyindolin-2-one 13**: $[\alpha]_D^{20}$ +10.0 (*c* 1.0, $CHCl_3$) {Lit.[2] (94% ee) +13 (*c* 1.0, $CHCl_3$)}; **Chiral HPLC analysis**: Chiralpak AD-H (95:5 hexane:IPA, flow rate 1.0 mLmin^{-1}, 211 nm, 30 °C) t_R (*R*): 23.4 min, t_R (*S*): 28.5 min, >99:1 (*R*:*S*) er. ***(S)*-3-Allyl-1-benzyl-2-oxoindolin-3-yl isobutyrate S11**: $[\alpha]_D^{20}$ +14.0 (*c* 1.0, $CHCl_3$) {Lit.[2] (88% ee) +13 (*c* 1.0, $CHCl_3$)}; **Chiral HPLC analysis**: Chiralpak AD-H, (98:2 hexane:IPA, flow rate 1.0 mLmin^{-1}, 211 nm, 30 °C) t_R (*S*): 14.8 min, t_R (*R*): 16.7 min, 87:13 (*S*:*R*) er.

1-Benzyl-3-ethyl-3-hydroxylindolin-2-one (14)

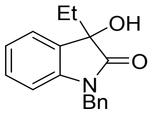

Following **General Procedure A**, 1-benzylindoline-2,3-dione **S1** (1.18 g, 5.0 mmol) and ethylmagnesium bromide (0.9 M, 6 mL, 6.0 mmol) were reacted in anhydrous THF (25 mL) under N_2. The reaction was concentrated under reduced pressure and purified by column chromatography (Petrol:EtOAc 3:1) to give 1-benzyl-3-ethyl-3-hydroxylindolin-2-one **14** as a white solid. (0.65 g, 2.4 mmol, 49%) with spectroscopic data in accordance with the literature.[5]

m.p. 119−122 °C {Lit.[2] 118−119 °C}; **^{1}H NMR** (400 MHz, $CDCl_3$) δ_H: 7.38 (1H, d, *J* 7.3), 7.26−7.35 (5H, m), 7.21 (1H, td, *J* 7.6 1.6), 7.05 (1H, td, *J* 7.2 1.0), 6.73 (1H, d, *J* 8.1), 5.02 (1H, d, *J* 15.0), 4.76 (1H, d, *J* 15.0), 2.63 (1H, s), 1.97−2.14 (2H, m), 0.80 (3H, t, *J* 7.4).

1-Benzyl-3-ethyl-2-oxoindolin-3-yl isobutyrate (S12)

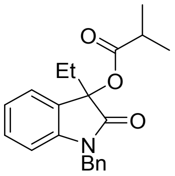

Following **General Procedure B**, 1-benzyl-3-ethyl-3-hydroxylindolin-2-one **14** (90 mg, 0.34 mmol), isobutyric anhydride (65 μL, 0.37 mmol), DMAP (4.1 mg, 0.034 mmol, 10 mol%) and *i*-Pr_2NEt (56 μL, 0.34 mmol) were reacted in CH_2Cl_2 (10 mL). The reaction was concentrated under reduced pressure and purified by column chromatography (Petrol:EtOAc 2:1) to give 1-benzyl-3-ethyl-2-oxoindolin-3-yl isobutyrate **S12** as a dark red oil (92 mg, 0.27 mmol, 79%).

^{1}H NMR (400 MHz, $CDCl_3$) δ_H: 7.29−7.40 (4H, m) 7.23−7.29 (1H, m), 7.15−7.20 (2H, m), 7.00 (1H, td, *J* 7.4 1.0), 6.67 (1H, d, *J* 8.1), 5.05 (1H, d, *J* 15.9), 4.86 (1H, d, *J* 15.9), 2.62 (1H, sept, *J* 7.0), 2.11 (1H, dt, *J* 7.6 6.0), 2.02 (1H, dt, J 7.6 6.0), 1.73 (3H, d, *J* 1.5,), 1.16 (3H, d, *J* 1.5,), 0.88 (3H, t, *J* 7.4); **^{13}C{^{1}H} NMR** (101 MHz, $CDCl_3$) δ_C: 175.2, 175.1, 143.2, 136.0, 129.5, 128.9, 127.9, 127.6, 127.4, 122.7, 122.4, 109.5, 80.1, 44.2, 33.6, 30.3, 19.0, 18.7, 7.0; **IR** ν_{max} (film) 2974 (C-H), 1720 (C=O), 1614, 1465, 1350, 1180, 1152 cm^{-1}; **HRMS** (NSI$^+$) calculated for $C_{21}H_{23}NO_3Na^+$ $[M+Na]^+$ requires 360.1570; found 360.1560 (−2.8 ppm).

Kinetic Resolution of 1-Benzyl-3-ethyl-3-hydroxy-indolin-2-one (14)

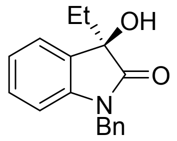
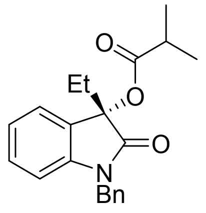

Following **General Procedure C**, 1-benzyl-3-ethyl-3-hydroxylindolin-2-one **14** (651 mg, 2.4 mmol), isobutyric anhydride (0.30 mL, 1.7 mmol), (*2S,3R*)-HyperBTM (8.0 mg, 0.024 mmol) and *i*-Pr_2NEt (0.25 mL, 1.5 mmol) were reacted in $CHCl_3$ (15 mL) at room temperature for 24 h. The reaction was concentrated under reduced pressure and purified by column chromatography (Petrol:EtOAc 3:1) to give (*S*)-1-benzyl-3-ethyl-2-oxolindolin-3-yl isobutyrate **S12** (153 mg, 0.45 mmol, 19%) and (*R*)-1-benzyl-3-ethyl-3-hydroxylindolin-2-one **14** (104 mg, 0.38 mmol, 16%).

***(R)*-1-Benzyl-3-ethyl-3-hydroxylindolin-2-one 14**: $[\alpha]_D^{20}$ +28 (*c* 0.1, $CHCl_3$) {Lit.[7] (ent, 99% ee) −49 (*c* 0.1, $CHCl_3$)}; **Chiral HPLC analysis**: Chiralpak IC (80:20 hexane:IPA, flow rate 1.0 mLmin^{-1}, 211 nm, 30 °C) t_R (*R*): 7.7 min, t_R (*S*): 12.6 min, >99:1 (*R*:*S*) er. ***(S)*-1-Benzyl-3-ethyl-2-oxoindolin-3-yl isobutyrate S12**: $[\alpha]_D^{20}$ −10 (*c* 1.5, $CHCl_3$); **Chiral HPLC analysis**: Chiralpak AD-H (98:2 hexane:IPA, flow rate 1.0 mLmin^{-1}, 211 nm, 30 °C) t_R (*S*): 14.6 min, t_R (*R*): 17.1 min, 71:29 (*S*:*R*) er.

1-Benzyl-3-hydroxy-3-isopropylindolin-2-one (15): The racemic oxindole was prepared in a previous report from this group.[2]

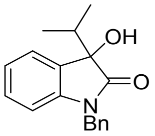

^{1}H NMR (300 MHz, $CDCl_3$) δ_H: 7.11–7.40 (8 H, m), 7.01 (1H, td, *J* 7.6, 1.1), 6.69 (1H, d, *J* 7.8), 4.98 (1H, d, *J* 15.6), 4.67 (1H, d, *J* 15.6), 3.26 (1H, s), 2.31 (1H, hept, *J* 6.8), 1.10 (3H, d, *J* 6.9), 0.75 (3H, d, *J* 6.8).

1-Benzyl-3-isopropyl-2-oxoindolin-3-yl isobutyrate (S13): The racemic ester was prepared in a previous report from this group.[2]

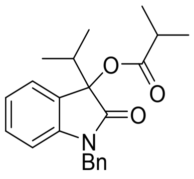

^{1}H NMR (500 MHz, $CDCl_3$) δ_H: 7.39–7.44 (2 H, m), 7.30–7.37 (2H, m), 7.23–7.30 (1H, m), 7.19 (2H, td, *J* 7.6, 1.2), 7.00 (1H, td, *J* 7.5, 1.0), 6.67–6.72 (1H, m), 5.06 (1H, d, *J* 15.9), 4.88 (1H, d, *J* 15.9), 2.65 (1H, hept, *J* 7.0), 2.47 (1H, hept, *J* 6.8), 1.19 (6H, dd, *J* 7.0, 2.4), 1.13 (3H, d, *J* 6.9), 0.83 (3H, d, *J* 6.8).

Kinetic Resolution of 1-Benzyl-3-hydroxy-3-isopropylindolin-2-one (15)

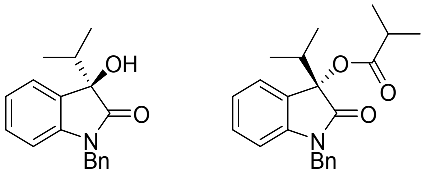

Following **General Procedure C**, 1-benzyl-3-hydroxy-3-isopropylindolin-2-one **15** (300 mg, 1.4 mmol), isobutyric anhydride (0.12 mL, 1.1 mmol), (*2S,3R*)-HyperBTM (5.0 mg, 0.015 mmol) and *i*-Pr_2NEt (0.11 mL, 0.9 mmol) were reacted in $CHCl_3$ (6 mL) at room temperature for 24 h. The reaction was concentrated under reduced pressure and purified by column chromatography (30% EtOAc:Hexane) to give (*S*)-1-benzyl-3-isopropyl-2-oxoindolin-3-yl isobutyrate **S13** as a yellow solid (81 mg, 0.38 mmol, 27%) and (*R*)-1-benzyl-3-hydroxy-3-isopropylindolin-2-one **15** as a colorless oil (122 mg, 0.34 mmol, 24%).

***(R)*-1-Benzyl-3-hydroxy-3-isopropylindolin-2-one 15**: $[\alpha]_D^{20}$ +64.0 (*c* 1.0, $CHCl_3$) {Lit.[2] (70% ee) +59 (*c* 0.1, $CHCl_3$)}; **Chiral HPLC analysis**: Chiralpak AD-H (95:5 hexane:IPA, flow rate 1.0 mLmin^{-1}, 211 nm, 30 °C) t_R (*R*): 23.4 min, t_R (*S*): 28.5 min, >99:1 (*R*:*S*) er. ***(S)*-1-Benzyl-2-oxo-3-isopropylindolin-3-yl isobutyrate S13**: $[\alpha]_D^{20}$ −10.5 (*c* 1.0, $CHCl_3$) {Lit.[2] (93% ee) −30 (*c* 1.0, $CHCl_3$)}; **Chiral HPLC analysis**: Chiralpak AD-H, (98:2 hexane:IPA, flow rate 1.5 mLmin^{-1}, 211 nm, 40 °C) t_R (*S*): 6.8 min, t_R (*R*): 8.1 min, 66:34 (*S*:*R*) er.

2-Methyl-1-phenylpropan-1-ol (17)

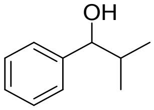

Following **General Procedure A**, isobutyraldehyde (0.6 mL, 6.6 mmol) and phenylmagnesium bromide (3.0 M, 3.3 mL, 9.9 mmol) were reacted in anhydrous THF (21 mL) under N_2. The reaction was concentrated under reduced pressure and purified by column chromatography (10% EtOAc:Hexane) to give 2-methyl-1-phenylpropan-1-ol **17** as a colorless oil (0.67 g, 4.8 mmol, 72%) with spectroscopic data in accordance with the literature.[8]

^{1}H NMR (500 MHz, $CDCl_3$) δ_H: 7.24–7.38 (5H, m), 4.33 (1H, d, *J* 6.9), 2.16 (1H, s), 1.90–2.00 (1H, m, *J* 6.9), 1.01 (3H, d, *J* 6.7), 0.80 (3H, d, *J* 6.9).

2-Methyl-1-phenylpropyl isobutyrate (S14)

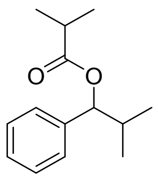

Following **General Procedure B**, 2-methyl-1-phenylpropan-1-ol **17** (45 mg, 0.30 mmol), isobutyric anhydride (55 µL, 0.33 mmol), DMAP (3.6 mg, 0.03 mmol, 10 mol%) and *i*-Pr_2NEt (52 µL, 0.30 mmol) were reacted in CH_2Cl_2 (1 mL). The reaction was concentrated under reduced pressure and purified by column chromatography (5% EtOAc:Hexane) to give 2-methyl-1-phenylpropyl isobutyrate **S14** as a colorless oil (38 mg, 0.17 mmol, 58%) with spectroscopic data in accordance with the literature.[8]

^{1}H NMR (500 MHz, $CDCl_3$) δ_H: 7.22–7.36 (5H, m), 5.48 (1H, d, *J* 7.3), 2.60 (1H, hept, *J* 7.0), 2.04–2.16 (1H, m, *J* 6.8), 1.21 (3H, d, *J* 7.0), 1.17 (3H, d, *J* 7.0), 0.98 (3H, d, *J* 6.7), 0.82 (3H, d, *J* 6.8).

Kinetic Resolution of 2-Methyl-1-phenylpropan-1-ol (17)

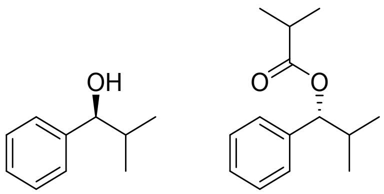

Following **General Procedure C**, 2-methyl-1-phenylpropan-1-ol **17** (400 mg, 1.5 mmol), isobutyric anhydride (0.19 mL, 1.1 mmol), (*2S,3R*)-HyperBTM (5.0 mg, 0.015 mmol) and *i*-Pr_2NEt (0.15 mL, 0.9 mmol) were reacted in $CHCl_3$ (15 mL) at room temperature for 24 h. The reaction was concentrated under reduced pressure and purified by column chromatography (10% EtOAc:Hexane) to give (*S*)-2-methyl-1-phenylpropan-1-ol **17** as a colorless oil (222 mg, 0.66 mmol, 44%) and (*R*)-2-methyl-1-phenylpropyl isobutyrate **S14** as a colorless oil (215 mg, 0.81 mmol, 54%).

***(S)*-2-Methyl-1-phenylpropan-1-ol 17**: $[\alpha]_D^{20}$ –48.4 (*c* 1.0, $CHCl_3$) {Lit.[8] (*ent*, 64% ee) +18 (*c* 1.0, $CHCl_3$)}; **Chiral HPLC analysis**: Chiralpak AD-H (99.5:0.5 hexane:IPA, flow rate 1.0 mLmin^{-1}, 220 nm, 30 °C) t_R (*R*) : 18.5 min, t_R (*S*): 20.2 min, >99:1 (*S*:*R*) er. ***(R)*-2-Methyl-1-phenylpropyl isobutyrate S14**: $[\alpha]_D^{20}$ +72.0 (*c* 1.0, $CHCl_3$) {Lit.[8] (*ent*, 96% ee) –108 (*c* 0.1, $CHCl_3$)}; **Chiral HPLC analysis**: Chiralpak OJ-H (99.5:0.5 hexane:IPA, flow rate 1.0 mLmin^{-1}, 220 nm, 30 °C) t_R (*R*): 4.5 min, t_R (*S*): 5.8 min, 93:7 (*R*:*S*) er.

2,2-Dimethyl-1-phenylpropan-1-ol (18): Commercially available.

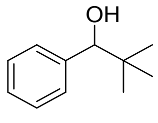

2,2-Dimethyl-1-phenylpropyl isobutyrate (S15): The racemic ester was prepared in a previous report from this group.[8]

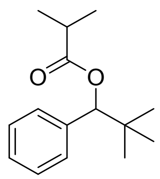

^{1}H NMR (500 MHz, $CDCl_3$) δ_H: 7.22–7.34 (5H, m), 0.93 (9H, s), 5.47 (1H, s), 2.61 (1H, hept, *J* 7.0), 1.21 (3H, d, *J* 7.0), 1.18 (3H, d, *J* 7.0).

Kinetic Resolution of 2,2-Dimethyl-1-phenylpropanol (18)

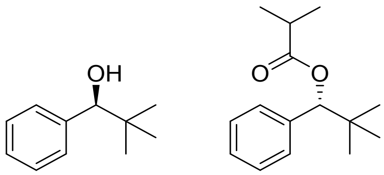

Following **General Procedure C**, 2,2-dimethyl-1-phenylpropanol **18** (490 mg, 3.0 mmol), isobutyric anhydride (0.36 mL, 2.1 mmol), (*2S,3R*)-HyperBTM (10.0 mg, 0.03 mmol) and *i*-Pr_2NEt (0.29 mL, 1.8 mmol) were reacted in $CHCl_3$ (15 mL) at room temperature for 24 h. The reaction was concentrated under reduced pressure and purified by column chromatography (Petrol:EtOAc 3:1) to give (*R*)-2,2-dimethyl-1-phenylpropyl isobutyrate **S15** (125 mg, 0.53 mmol, 18%) and (*S*)-2,2-dimethyl-1-phenylpropanol **18** (143 mg, 0.87 mmol, 29%).

***(S)*-2,2-Dimethyl-1-phenylpropanol 18**: $[\alpha]_D^{20}$ −34 (*c* 0.5, $CHCl_3$) {Lit.[8] (*ent*, 62% ee) +33 (*c* 1.0, $CHCl_3$)}; **Chiral HPLC analysis**: Chiralpak OD-H (95:5 hexane:IPA, flow rate 1.0 mLmin^{-1}, 211 nm, 30 °C) t_R (*S*): 6.4 min, t_R (*R*): 9.7 min, >99:1 (*S*:*R*) er. ***(R)*-2,2-Dimethyl-1-phenylpropyl isobutyrate S15**: $[\alpha]_D^{20}$ +34 (*c* 1.0, $CHCl_3$) {Lit.[8] (*ent*, 97% ee) −62 (*c* 1.0, $CHCl_3$)}; **Chiral HPLC analysis**: Chiralpak AD-H (99.8:0.2 hexane:IPA, flow rate 1.0 mLmin^{-1}, 270 nm, 30 °C) t_R (*R*): 5.0 min, t_R (*S*): 6.5 min, 94:6 (*R*:*S*) er.

2,2-Dimethyl-1-(4-(trifluoromethyl)phenyl)propan-1-ol (19)

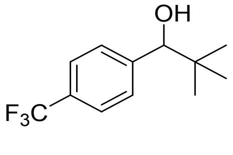

Following **General Procedure A**, 4-trifluoromethylbenzaldehyde (0.5 mL, 3.6 mmol) and *t*-butylmagnesium bromide (2.0 M, 2.8 mL, 5.5 mmol) were reacted in anhydrous THF (12 mL) under N_2. The reaction was concentrated under reduced pressure and purified by column chromatography (10%

EtOAc:Hexane) to give 2,2-dimethyl-1-(4-(trifluoromethyl)phenyl)propan-1-ol **19** as a colorless oil (0.18 g, 0.77 mmol, 21%) with spectroscopic data in accordance with the literature.[12]

^{1}H NMR (500 MHz, $CDCl_3$) δ_H: 7.55 (2H, d, *J* 8.1), 7.39 (2H, d, *J* 8.0), 4.39 (1H, d, *J* 2.5), 2.40 (1H, d, *J* 2.9), 0.90 (9H, s).

2,2-Dimethyl-1-(4-(trifluoromethyl)phenyl)propyl isobutyrate (S16)

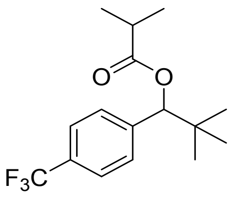

Following **General Procedure B**, 2,2-dimethyl-1-(4-(trifluoromethyl)phenyl)propan-1-ol **19** (25 mg, 0.11 mmol), isobutyric anhydride (18 µL, 0.12 mmol), DMAP (2.0 mg, 0.01 mmol, 10 mol%) and *i*-Pr_2NEt (19 µL, 0.11 mmol) were reacted in CH_2Cl_2 (0.5 mL). The reaction was concentrated under reduced pressure and purified by column chromatography (5% EtOAc:Hexane) to give 2,2-dimethyl-1-(4-(trifluoromethyl)phenyl)propyl isobutyrate **S16** as a colorless oil (16 mg, 0.05 mmol, 50%) with spectroscopic data in accordance with the literature.

^{1}H NMR (500 MHz, $CDCl_3$) δ_H: 7.56 (2H, d, *J* 8.1), 7.38 (2H, d, *J* 8.4), 5.49 (1H, s), 2.63 (1H, hept, *J* 7.0), 1.20 (6H, dd, *J* 10.8, 7.0), 0.94 (9H, s); **^{13}C{^{1}H} NMR** (126 MHz, $CDCl_3$) δ_C: 18.4, 19.0, 19.1, 26.1, 34.4, 35.2, 35.2, 82.0, 124.26 (q, *J* 272.0), 124.77 (q, *J* 3.8), 129.82 (q, *J* 32.4) 142.8, 176.0; **IR** ν_{max} (thin film, cm^{-1}): 2972.3 (C–H), 1735.9 (C=O), 1122.6, 1018.4, 842.9, 765.7 cm^{-1}; **HRMS** (ESI^+) calculated for $C_{16}H_{21}O_2F_3Na^+$ $[M+Na]^+$ requires 325.1391; found 325.1379 (−2.0 ppm).

Kinetic Resolution of 2,2-Dimethyl-1-(4-(trifluoromethyl)phenyl)propan-1-ol (19)

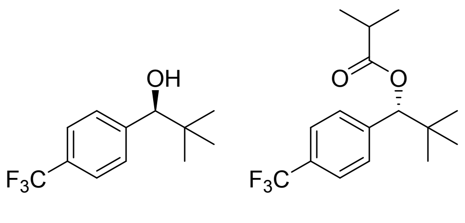

Following **General Procedure C**, 2,2-dimethyl-1-(4-(trifluoromethyl)phenyl)propan-1-ol **19** (150 mg, 0.65 mmol), isobutyric anhydride (0.064 mL, 0.39 mmol), (*2S,3R*)-HyperBTM (2.0 mg, 0.006 mmol) and *i*-Pr_2NEt (0.067 mL, 0.39 mmol) were reacted in $CHCl_3$ (3 mL) at room temperature for 48 h. The reaction was concentrated under reduced pressure and purified by column chromatography (10% EtOAc:Hexane) to give (*S*)-2,2-dimethyl-1-(4-(trifluoromethyl)phenyl)propan-1-ol **19** (39 mg, 0.17 mmol, 26%) and (*R*)-2,2-dimethyl-1-(4-(trifluoromethyl)phenyl)propyl isobutyrate **S16** (74 mg, 0.25 mmol, 38%).

***(S)*-2,2-Dimethyl-1-(4-(trifluoromethyl)phenyl)propan-1-ol 19**: $[\alpha]_D^{20}$ –20.5 (*c* 1.0, $CHCl_3$) {Lit.[12] (76% ee) –11.3 (*c* 0.5, $CHCl_3$)}; **Chiral HPLC analysis**: Chiralpak OJ-H (99:1 hexane:IPA, flow rate 1.0 mLmin^{-1}, 220 nm, 30 °C) t_R (*S*): 9.4 min, t_R (*R*): 10.3 min, 97:3 (*S*:*R*) er. ***(R)*-2,2-Dimethyl-1-(4-(trifluoromethyl)phenyl)propyl isobutyrate S16**: $[\alpha]_D^{20}$ +29.3 (*c* 1.0, $CHCl_3$); **Chiral HPLC analysis**: Chiralpak AD-H (99.8:0.2 hexane:IPA, flow rate 1.0 mLmin^{-1}, 211 nm, 30 °C) t_R (*R*): 5.1 min, t_R (*S*): 5.7 min, 87:13 (*R*:*S*) er.

1-(4-Chlorophenyl)-2,2-dimethylpropan-1-ol (20)

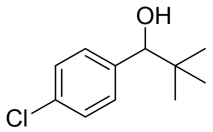

Following **General Procedure A**, 4-chlorobenzaldehyde (0.50 g, 3.6 mmol) and *t*-butylmagnesium bromide (2.0 M, 2.7 mL, 5.3 mmol) were reacted in anhydrous THF (12 mL) under N_2. The reaction was concentrated under reduced pressure and purified by column chromatography (10% EtOAc:Hexane) to give 1-(4-chlorophenyl)-2,2-dimethylpropan-1-ol **20** as a yellow oil (0.34 g, 1.7 mmol, 48%) with spectroscopic data in accordance with the literature.[12]

^{1}H NMR (500 MHz, $CDCl_3$) δ_H: 7.25–7.29 (2H, m), 7.21 (2H, d, *J* 8.5), 4.32 (1H, d, *J* 2.9), 2.18 (1H, d, *J* 3.0), 0.89 (9H, s).

1-(4-Chlorophenyl)-2,2-dimethylpropyl isobutyrate (S17)

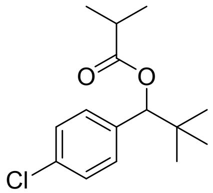

Following **General Procedure B**, 1-(4-chlorophenyl)-2,2-dimethylpropan-1-ol **20** (15 mg, 0.08 mmol), isobutyric anhydride (15 μL, 0.09 mmol), DMAP (2.0 mg, 0.01 mmol, 10 mol%) and *i*-Pr_2NEt (13 μL, 0.08 mmol) were reacted in CH_2Cl_2 (0.3 mL). The reaction was concentrated under reduced pressure and purified by column chromatography (5% EtOAc:Hexane) to give 1-(4-chlorophenyl)-2,2-dimethylpropyl isobutyrate **S17** as a white solid (16 mg, 0.06 mmol, 80%).

mp: 44–46 °C. **^{1}H NMR** (500 MHz, $CDCl_3$) δ_H: 7.26–7.30 (2H, m), 7.17–7.21 (2H, m), 5.42 (1H, s), 2.61 (1H, hept, *J* 7.0), 1.19 (6H, dd, *J* 13.1, 7.0), 0.92 (9H, s); **^{13}C {^{1}H} NMR** (126 MHz, $CDCl_3$) δ_C: 175.9, 137.3, 133.4, 129.1, 128.0, 81.9, 35.2, 34.4, 26.1, 19.1, 19.0; **IR** ν_{max} (thin film, cm^{-1}): 2970.4 (C–H),

1732.1 (C=O), 1091.7, 983.7, 825.5, 767.7 cm^{-1}; **HRMS** (ESI^{+}) calculated for $C_{15}H_{21}O_2ClNa^+$ [M+Na]$^+$ requires 291.1128; found 291.1116 (−2.3 ppm).

Kinetic Resolution of 1-(4-Chlorophenyl)-2,2-dimethylpropan-1-ol (20)

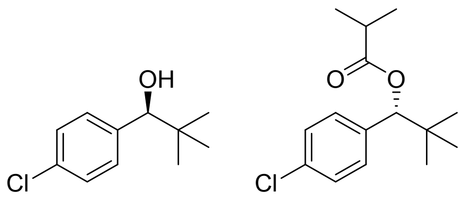

Following **General Procedure C**, 1-(4-chlorophenyl)-2,2-dimethylpropan-1-ol **20** (171 mg, 0.86 mmol), isobutyric anhydride (0.1 mL, 0.6 mmol), (*2S,3R*)-HyperBTM (5.0 mg, 0.015 mmol) and *i*-Pr_2NEt (0.1 mL, 0.5 mmol) were reacted in $CHCl_3$ (5 mL) at room temperature for 48 h. The reaction was concentrated under reduced pressure and purified by column chromatography (10% EtOAc:Hexane) to give (*S*)-1-(4-chlorophenyl)-2,2-dimethylpropan-1-ol **S17** (54 mg, 0.28 mmol, 32%) and (*R*)-1-(4-chlorophenyl)-2,2-dimethylpropyl isobutyrate **20** (90 mg, 0.34 mmol, 39%).

***(S)*-1-(4-Chlorophenyl)-2,2-dimethylpropan-1-ol 20**: $[\alpha]_D^{20}$ –28.4 (*c* 1.0, $CHCl_3$) {Lit.[12] (80% ee) –22.9 (*c* 1.0, $CHCl_3$)}; **Chiral HPLC analysis**: Chiralpak IC (99.8:0.2 hexane:IPA, flow rate 1.0 mLmin^{-1}, 211 nm, 30 °C) t_R (*S*): 7.2 min, t_R (*R*): 7.7 min, 98:2 (*S*:*R*) er. ***(R)*-1-(4-Chlorophenyl)-2,2-dimethylpropyl isobutyrate S17**: $[\alpha]_D^{20}$ +51.5 (*c* 1.0, $CHCl_3$); **Chiral HPLC analysis**: Chiralpak AD-H (99.5:0.5 hexane:IPA, flow rate 1.0 mLmin^{-1}, 211 nm, 30 °C) t_R (*S*): 4.3 min, t_R (*R*): 4.6 min, >99:1 (*S*:*R*) er.

2,2-Dimethyl-1-(*p*-tolyl)propan-1-ol (21)

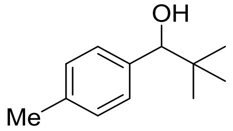

Following **General Procedure A**, *p*-tolylaldehyde (0.6 mL, 5.1 mmol) and *t*-butylmagnesium bromide (2.0 M, 3.8 mL, 7.5 mmol) were reacted in anhydrous THF (17 mL) under N_2. The reaction was concentrated under reduced pressure and purified by column chromatography (10% EtOAc:Hexane) to give 2,2-dimethyl-1-(*p*-tolyl)propan-1-ol **21** as a colorless oil (0.44 g, 2.4 mmol, 48%) with spectroscopic data in accordance with the literature.[10]

^{1}H NMR (500 MHz, $CDCl_3$) δ_H: 7.17–7.22 (2H, m), 7.12 (2H, d, *J* 7.9), 4.37 (1H, d, *J* 2.9), 2.34 (3H, s), 1.77 (1H, d, *J* 2.9), 0.92 (9H, s).

2,2-Dimethyl-1-(*p*-tolyl)propyl isobutyrate (S18)

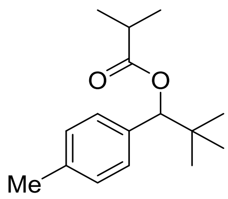

Following **General Procedure B**, 2,2-dimethyl-1-(*p*-tolyl)propan-1-ol **21** (15 mg, 0.08 mmol), isobutyric anhydride (16 µL, 0.09 mmol), DMAP (3.6 mg, 0.03 mmol, 10 mol%) and *i*-Pr_2NEt (13 µL, 0.08 mmol) were reacted in CH_2Cl_2 (0.3 mL). The reaction was concentrated under reduced pressure and purified by column chromatography (5% EtOAc:Hexane) to give 2,2-dimethyl-1-(*p*-tolyl)propyl isobutyrate **S18** as a colorless oil (46 mg, 0.18 mmol, 61%).

^{1}H NMR (500 MHz, $CDCl_3$) δ_H: 7.15 (2H, d, *J* 8.2), 7.10 (2H, d, *J* 8.0), 5.44 (1H, s), 2.60 (1H, hept, *J* 7.0), 2.33 (3H, s), 1.19 (6H, dd, *J* 13.3, 7.0), 0.93 (9H, s); **^{13}C{^{1}H} NMR** (126 MHz, $CDCl_3$) δ_C: 175.9, 137.1, 135.8, 128.4, 127.7, 82.4, 35.2, 34.5, 26.2, 21.2, 19.1, 19.0; **IR** ν_{max} (thin film): 2970.4 (C–H), 1734.0 (C=O), 1066.6, 977.9, 819.6, 763.8 cm^{-1}; **HRMS** (ESI$^+$) calculated for $C_{16}H_{24}O_2Na^+$ [M+Na]$^+$ requires 271.1674; found 271.1662 (−2.3 ppm).

Kinetic Resolution of 2,2-Dimethyl-1-(*p*-tolyl)propan-1-ol (21)

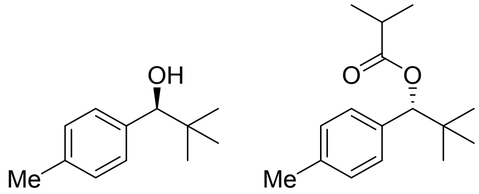

Following **General Procedure C**, 2,2-dimethyl-1-(*p*-tolyl)propan-1-ol **21** (400 mg, 2.2 mmol), isobutyric anhydride (0.26 mL, 1.4 mmol), (*2S,3R*)-HyperBTM (6.8 mg, 0.022 mmol) and *i*-Pr_2NEt (0.22 mL, 1.2 mmol) were reacted in $CHCl_3$ (10 mL) at room temperature for 24 h. The reaction was concentrated under reduced pressure and purified by column chromatography (10% EtOAc:Hexane) to give (*S*)- 2,2-dimethyl-1-(*p*-tolyl)propan-1-ol (112 mg, 0.62 mmol, 28%) **21** and (*R*)-2,2-dimethyl-1-(*p*-tolyl)propyl isobutyrate **S18** (134 mg, 0.53 mmol, 24%).

***(S)*-2,2-Dimethyl-1-(*p*-tolyl)propan-1-ol 21**: $[\alpha]_D^{20}$ −39.0 (*c* 1.0, $CHCl_3$); **Chiral HPLC analysis**: Chiralpak OJ-H (98:2 hexane:IPA, flow rate 1.0 mLmin^{-1}, 220 nm, 30 °C) t_R (*S*): 5.7 min, t_R (*R*): 6.2 min, >99:1 (*S*:*R*) er. ***(R)*-2,2-Dimethyl-1-(*p*-tolyl)propyl isobutyrate S18**: $[\alpha]_D^{20}$ +45.9 (*c* 1.0, $CHCl_3$); **Chiral HPLC analysis**: Chiralpak AD-H (99.5:0.5 hexane:IPA, flow rate 1.0 mLmin^{-1}, 211 nm, 30 °C) t_R (*R*): 4.4 min, t_R (*S*): 5.0 min, 79:21 (*R*:*S*) er.

2,2-Dimethyl-1-(naphthalen-2-yl)propan-1-ol (22)

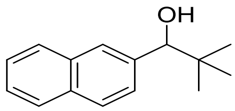

Following **General Procedure A**, 2-naphthaldehyde (0.5 g, 3.2 mmol) and *t*-butylmagnesium bromide (2.0 M, 2.4 mL, 4.8 mmol) were reacted in anhydrous THF (11 mL) under N_2. The reaction was concentrated under reduced pressure and purified by column chromatography (10% EtOAc:Hexane) to give 2,2-dimethyl-1-(naphthalen-2-yl)propan-1-ol **22** as a white solid (0.38 g, 1.8 mmol, 55%) with spectroscopic data in accordance with the literature.[11]

m.p. 42–44 °C; **^{1}H NMR** (500 MHz, $CDCl_3$) δ_H: 7.81–7.87 (2H, m), 7.74–7.81 (2H, m), 7.43–7.51 (3H, m), 4.58 (1H, d, *J* 2.7), 1.96 (1H, d, *J* 2.9), 0.98 (9H, s).

2,2-Dimethyl-1-(naphthalen-2-yl)propyl isobutyrate (S19)

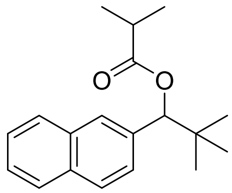

Following **General Procedure B**, 2,2-dimethyl-1-(naphthalen-2-yl)propan-1-ol **22** (25 mg, 0.12 mmol), isobutyric anhydride (21 µL, 0.13 mmol), DMAP (1.5 mg, 0.01 mmol, 10 mol%) and *i*-Pr_2NEt (20 µL, 0.12 mmol) were reacted in CH_2Cl_2 (0.4 mL). The reaction was concentrated under reduced pressure and purified by column chromatography (5% EtOAc:Hexane) to give 2,2-dimethyl-1-(naphthalen-2-yl)propyl isobutyrate **S19** as a white solid (20 mg, 0.07 mmol, 60%) with spectroscopic data in accordance with the literature.[11]

m.p. 38–41 °C; **^{1}H NMR** (500 MHz, $CDCl_3$) δ_H: 7.84–7.89 (2H, m), 7.82 (1H, d, *J* 8.5), 7.76 (1H, s), 7.48 (3H, t, *J* 11.1), 5.70 (1H, s), 2.69 (1H, hept, *J* 7.0), 1.26 (6H, dd, *J* 16.1, 7.0), 1.03 (9H, s).

Kinetic Resolution of 2,2-Dimethyl-1-(naphthalen-2-yl)propan-1-ol 22

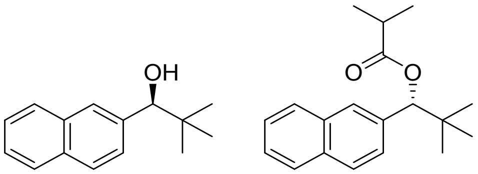

Following **General Procedure C**, 2,2-dimethyl-1-(naphthalen-2-yl)propan-1-ol **22** (345 mg, 1.6 mmol), isobutyric anhydride (0.16 mL, 0.98 mmol), (*2S,3R*)-HyperBTM (5.0 mg, 0.016 mmol) and *i*-Pr_2NEt (0.17 mL, 0.98 mmol) were reacted in $CHCl_3$ (8 mL) at room temperature for 24 h. The reaction was concentrated under reduced pressure and purified by column chromatography (10% EtOAc:Hexane) to give (*S*)-2,2-

dimethyl-1-(naphthalen-2-yl)propan-1-ol **22** (78 mg, 0.37 mmol, 23%) and (*R*)-2,2-dimethyl-1-(naphthalen-2-yl)propyl isobutyrate **S19** (134 mg, 0.46 mmol, 29%).

***(S)*-2,2-Dimethyl-1-(naphthalen-2-yl)propan-1-ol 22**: $[\alpha]_D^{20}$ –26.8 (*c* 1.0, $CHCl_3$); **Chiral HPLC analysis**: Chiralpak OJ-H (98:2 hexane:IPA, flow rate 1.0 mLmin^{-1}, 220 nm, 30 °C) t_R (*S*): 16.5 min, t_R (*R*): 27.7 min, 97:3 (*S*:*R*) er. ***(R)*-2,2-Dimethyl-1-(naphthalen-2-yl)propyl isobutyrate S19**: $[\alpha]_D^{20}$ +51.5 (*c* 1.0, $CHCl_3$); **Chiral HPLC analysis**: Chiralpak AD-H (99.5:0.5 hexane:IPA, flow rate 1.0 mLmin^{-1}, 220 nm, 30 °C) t_R (*S*): 5.7 min, t_R (*R*): 4.3 min, 84:16 (*R*:*S*) er.

1-(4-Methoxyphenyl)-2,2,-dimethylpropan-1-ol (23)

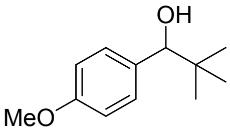

Following **General Procedure A**, pivaldehyde (0.5 mL, 4.6 mmol) and 4-methoxyphenylmagnesium bromide (1.0 M, 6.9 mL, 6.9 mmol) were reacted in anhydrous THF (15 mL) under N_2. The reaction was concentrated under reduced pressure and purified by column chromatography (10% EtOAc:Hexane) to give 1-(4-methoxyphenyl)-2,2,-dimethylpropan-1-ol **23** as a colorless oil (0.70 g, 3.6 mmol, 78%) with spectroscopic data in accordance with the literature.[9]

^{1}H NMR (500 MHz, $CDCl_3$) δ_H: 7.21–7.25 (2H, m), 6.82–6.88 (2H, m), 4.36 (1H, d, *J* 2.8), 3.81 (3H, s), 1.76 (1H, d, *J* 2.8), 0.91 (9H, s).

1-(4-Methoxyphenyl)-2,2-dimethylpropyl isobutyrate S20

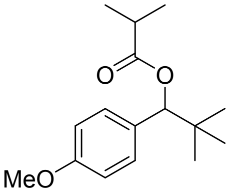

Following **General Procedure B**, 1-(4-methoxyphenyl)-2,2,-dimethylpropan-1-ol **23** (58 mg, 0.30 mmol), isobutyric anhydride (55 μL, 0.33 mmol), DMAP (3.6 mg, 0.03 mmol, 10 mol%) and *i*-Pr_2NEt (52 μL, 0.30 mmol) were reacted in CH_2Cl_2 (1 mL). The reaction was concentrated under reduced pressure and purified by column chromatography (5% EtOAc:Hexane) to give 1-(4-methoxyphenyl)-2,2-dimethylpropyl isobutyrate **S20** as a colorless oil (51 mg, 0.20 mmol, 65%).

^{1}H NMR (500 MHz, $CDCl_3$) δ_H: 7.16–7.22 (2H, m), 6.80–6.87 (2H, m), 5.44 (1H, s), 3.79 (3H, s), 2.60 (1H, hept, *J* 7.0), 1.19 (6H, dd, *J* 13.5, 7.0), 0.92 (9H, s); **^{13}C{^{1}H} NMR** (126 MHz, $CDCl_3$) δ_C: 175.8, 158.9, 130.8, 128.8, 113.0, 82.1, 55.1, 35.2, 34.4, 26.1, 19.0, 18.9; **IR** ν_{max} (thin film): 2970.4 (C–H), 1732.1

(C=O), 1033.8, 977.9, 831.3, 769.6 cm^{-1}; **HRMS** (ESI$^+$) calculated for $C_{16}H_{24}O_3Na^+$ [M+Na]$^+$ requires 287.1623; found 287.1611 (−2.3 ppm).

Kinetic Resolution of 1-(4-Methoxyphenyl)-2,2,-dimethylpropan-1-ol (23)

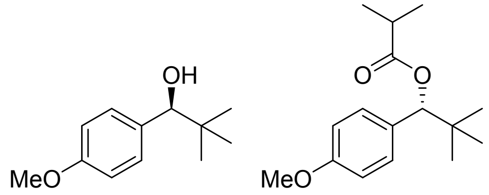

Following **General Procedure C**, 1-(4-methoxyphenyl)-2,2,-dimethylpropan-1-ol **23** (515 mg, 2.7 mmol), isobutyric anhydride (0.22 mL, 1.3 mmol), (*2S,3R*)-HyperBTM (6.8 mg, 0.022 mmol) and *i*-Pr_2NEt (0.23 mL, 1.3 mmol) were reacted in $CHCl_3$ (11 mL) at room temperature for 24 h. The reaction was concentrated under reduced pressure and purified by column chromatography (10% EtOAc:Hexane) to give (*S*)-1-(4-methoxyphenyl)-2,2,-dimethylpropan-1-ol **23** (222 mg, 1.1 mmol, 43%) and (*R*)-1-(4-methoxyphenyl)-2,2-dimethylpropyl isobutyrate **S20** (339 mg, 1.3 mmol, 48%).

***(S)*-1-(4-Methoxyphenyl)-2,2,-dimethylpropan-1-ol 23**: $[\alpha]_D^{20}$ –36.5 (*c* 1.0, $CHCl_3$) {Lit.[9] (92% ee) –33.99 (*c* 0.71, $CHCl_3$)}; **Chiral HPLC analysis**: Chiralpak AD-H (99:1 hexane:IPA, flow rate 1.0 mLmin^{-1}, 220 nm, 30 °C) t_R (*R*): 21.1 min, t_R (*S*): 23.3 min, >99:1 (*S*:*R*) er. ***(R)*-1-(4-Methoxyphenyl)-2,2-dimethylpropyl isobutyrate S20**: $[\alpha]_D^{20}$ +53.5 (*c* 1.0, $CHCl_3$); **Chiral HPLC analysis**: Chiralpak AD-H (99.5:0.5 hexane:IPA, flow rate 1.0 mLmin^{-1}, 220 nm, 30 °C) t_R (*R*): 4.4 min, t_R (*S*): 5.8 min, 94:6 (*R*:*S*) er

4-Phenylbut-3-yn-2-ol 24

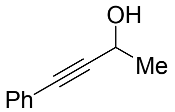

Following a **General Procedure A**, acetaldehyde (0.25 mL, 4.4 mmol) and lithium phenylacetylide (prepared by stirring *n*-BuLi and phenylacetylene, 4.8 mmol) were reacted in anhydrous Et_2O (13 mL) under N_2. The reaction was concentrated under reduced pressure and purified by column chromatography (10% EtOAc:Hexane) to give 4-phenylbut-3-yn-2-ol **24** as a yellow oil (0.57 g, 3.9 mmol, 89%) with spectroscopic data in accordance with the literature.[8]

^{1}H NMR (500 MHz, $CDCl_3$) δ_H: 7.35–7.41 (2H, m), 7.28–7.35 (2H, m), 7.20–7.26 (1H, m), 6.58 (1H, dt, *J* 15.9, 0.7), 6.27 (1H, dd, *J* 15.9, 6.3), 4.45–4.55 (1H, m), 1.38 (3H, d, *J* 6.4).

4-Phenylbut-3-yn-2-yl isobutyrate (S21)

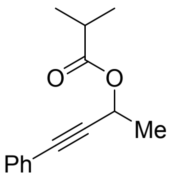

Following **General Procedure B**, 4-phenylbut-3-yn-2-ol **24** (30 mg, 0.21 mmol), isobutyric anhydride (41 μL, 0.23 mmol), DMAP (2.5 mg, 0.02 mmol, 10 mol%) and *i*-Pr_2NEt (36 μL, 0.21 mmol) were reacted in CH_2Cl_2 (0.7 mL). The reaction was concentrated under reduced pressure and purified by column chromatography (5% EtOAc:Hexane) to give 4-phenylbut-3-yn-2-yl isobutyrate **S21** as a colorless oil (34 mg, 0.16 mmol, 77%) with spectroscopic data in accordance with the literature.[8]

^{1}H NMR (500 MHz, $CDCl_3$) δ_H: 7.36–7.41 (2H, m), 7.29–7.35 (2H, m), 7.23–7.27 (1H, m), 6.57–6.64 (1H, m), 6.20 (1H, dd, *J* 16.0, 6.6), 5.53 (1H, pd, *J* 6.5, 1.3), 2.56 (1H, hept, *J* 6.9), 1.41 (3H, d, *J* 6.5), 1.19 (6H, dd, *J* 7.0, 3.1).

Kinetic Resolution of 4-Phenylbut-3-yn-2-ol (24)

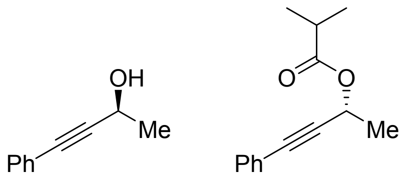

Following **General Procedure C**, 4-phenylbut-3-yn-2-ol **24** (540 mg, 3.69 mmol), isobutyric anhydride (0.37 mL, 2.2 mmol), (*2S,3R*)-HyperBTM (11.4 mg, 0.037 mmol) and *i*-Pr_2NEt (0.39 mL, 2.2 mmol) were reacted in $CHCl_3$ (18 mL) at 0 °C for 24 h. The reaction was concentrated under reduced pressure and purified by column chromatography (10% EtOAc:Hexane) to give (*S*)-4-phenylbut-3-yn-2-ol **24** (137 mg, 0.92 mmol, 25%) and (*R*)-4-phenylbut-3-yn-2-yl isobutyrate **S21** (431 mg, 2.0 mmol, 54%).

***(S)*-4-Phenylbut-3-yn-2-ol 24**: $[\alpha]_D^{20}$ –33.6 (*c* 1.0, $CHCl_3$) {Lit.[8] (*ent*, 90% ee) +25 (*c* 1.0, $CHCl_3$)}; **Chiral HPLC analysis**: Chiralpak OD-H (95:5 hexane:IPA, flow rate 1.0 mLmin^{-1}, 254 nm, 30 °C) t_R (*S*): 28.1 min, t_R (*R*): 10.8 min, >99:1 (*S*:*R*) er. ***(R)*-4-Phenylbut-3-yn-2-yl isobutyrate S21**: $[\alpha]_D^{20}$ +71.9 (*c* 1.0, $CHCl_3$) {Lit.[8] (*ent*, 81% ee) –23 (*c* 1.0, $CHCl_3$)}; **Chiral HPLC analysis**: Chiralpak AS-H (99.8:0.2 hexane:IPA, flow rate 1.0 mLmin^{-1}, 254 nm, 30 °C) t_R (*R*): 4.3 min, t_R (*S*): 4.2 min, 73:27 (*R*:*S*) er.

(*E*)-4-Phenylbut-3-en-2-ol (25)

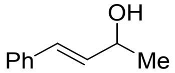

Following **General Procedure A**, *trans*-cinnamaldehyde (0.76 mL, 6.1 mmol) and methylmagnesium bromide (3.0 M, 3.0 mL, 9.1 mmol) were reacted in anhydrous THF (17 mL) under N_2. The reaction was concentrated under reduced pressure and purified by column chromatography (20% EtOAc:Hexane) to give (*E*)-4-phenylbut-3-en-2-ol **25** as a colorless oil (0.72 g, 5.6 mmol, 91%) with spectroscopic data in accordance with the literature.[8]

^{1}H NMR (500 MHz, $CDCl_3$) δ_H: 1.38 (3H, d, *J* 6.4), 4.45–4.55 (1H, m), 6.27 (1H, dd, *J* 15.9, 6.3), 6.58 (1H, dt, *J* 15.9, 0.7), 7.20–7.26 (1H, m), 7.28–7.35 (2H, m), 7.35–7.41 (2H, m).

(*E*)-4-Phenylbut-3-en-2-yl isobutyrate (S22)

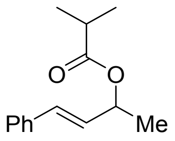

Following **General Procedure B**, (*E*)-4-phenylbut-3-en-2-ol **25** (25 mg, 0.17 mmol), isobutyric anhydride (33 μL, 0.19 mmol), DMAP (4.0 mg, 0.02 mmol, 10 mol%) and *i*-Pr_2NEt (30 μL, 0.17 mmol) were reacted in CH_2Cl_2 (0.6 mL). The reaction was concentrated under reduced pressure and purified by column chromatography (5% EtOAc:Hexane) to give (*E*)-4-phenylbut-3-en-2-yl isobutyrate **S22** as a colorless oil (24 mg, 0.12 mmol, 73%) with spectroscopic data in accordance with the literature.[8]

^{1}H NMR (500 MHz, $CDCl_3$) δ_H: 7.36–7.41 (2H, m), 7.29–7.35 (2H, m), 7.23–7.27 (1H, m), 6.57–6.64 (1H, m), 6.20 (1H, dd, *J* 16.0, 6.6), 5.53 (1H, pd, *J* 6.5, 1.3), 2.56 (1H, hept, *J* 6.9), 1.41 (3H, d, *J* 6.5), 1.19 (6H, dd, *J* 7.0, 3.1).

Kinetic Resolution of (*E*)-4-Phenylbut-3-en-2-ol (25)

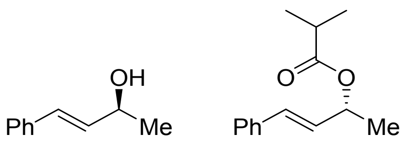

Following **General Procedure C**, (*E*)-4-phenylbut-3-en-2-ol **25** (695 mg, 3.9 mmol), isobutyric anhydride (0.39 mL, 2.4 mmol), (*2S,3R*)-HyperBTM (12.0 mg, 0.039 mmol) and *i*-Pr_2NEt (0.41 mL, 2.4 mmol) were reacted in $CHCl_3$ (20 mL) at 0 °C for 24 h. The reaction was concentrated under reduced pressure and purified by column chromatography (20% EtOAc:Hexane) to give (*S*,*E*)-4-phenylbut-3-en-2-ol (273 mg, 1.6 mmol, 40%) and (*R*,*E*)-4-phenylbut-3-en-2-yl isobutyrate **S22** (410 mg, 1.9 mmol, 48%).

(*S*,*E*)-4-Phenylbut-3-en-2-ol 25: $[\alpha]_D^{20}$ –26.1 (*c* 1.0, $CHCl_3$) {Lit.[8] (*ent*, 69% ee) +20 (*c* 0.1, $CHCl_3$)}; **Chiral HPLC analysis**: Chiralpak OD-H (95:5 hexane:IPA, flow rate 1.0 mLmin^{-1}, 220 nm, 30 °C) t_R (*S*): 25.0 min, t_R (*R*): 15.4 min, 96:4 (*S*:*R*) er. **(*R*,*E*)-4-Phenylbut-3-en-2-yl isobutyrate S22**: $[\alpha]_D^{20}$ +86.8 (*c*

1.0, $CHCl_3$) {Lit.[8] (*ent*, 79% ee) –87 (*c* 1.0, $CHCl_3$)}; **Chiral HPLC analysis**: Chiralpak AD-H (99.8:0.2 hexane:IPA, flow rate 1.0 mLmin^{-1}, 220 nm, 30 °C) t_R (*R*): 6.2 min, t_R (*S*): 6.8 min, 87:13 (*R*:*S*) er.

5. Racemization Kinetics at Varying Concentrations and Temperatures

The effect of catalyst loading and temperature on racemization. The ratio of boronic acid and oxalic acid was kept at 1:2 for each experiment. Samples were removed from the reaction mixture at the prescribed times and analyzed by HPLC.

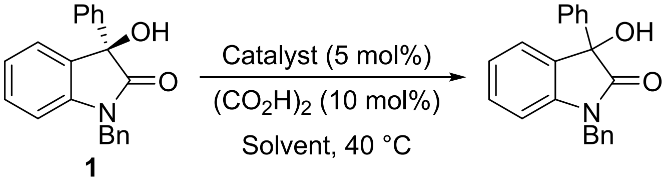

1

>99:1 er

Entry	T (°C)	c (mol%)	Enantiomeric excess (*ee*) (%)				
			0 min	15 min	30 min	60 min	180 min
1	60	5.0	>99	20.3	9.3	1.7	0.8
2	60	3.75	>99	28.9	15.0	5.5	2.0
3	60	2.5	>99	36.4	22.3	12.5	2.6
4	60	1.25	>99	45.3	31.9	19.0	5.3
5	80	1.25	>99	18.7	8.0	2.7	1.4

6. Mechanistic Studies

Reaction Inhibition in the presence of 2,6-Di-*tert*-butylpyridine 27.

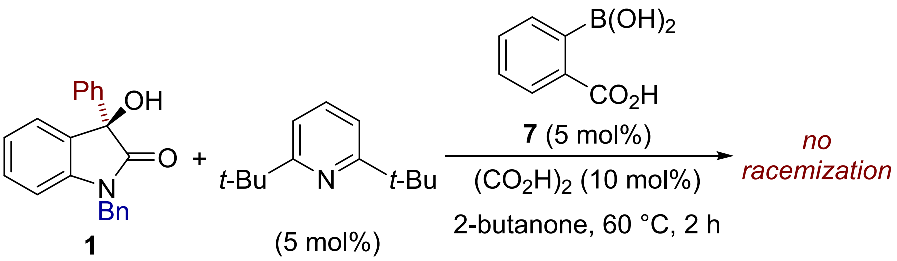

1

>99:1 er

The effect of base was analyzed under the reaction conditions where (*R*)-1-benzyl-3-hydroxy-3-phenylindolin-2-one **1** (>99:1 er, 100 mg, 0.32 mmol), 2-carboxyphenylboronic acid **7** (0.015 M, 1.0 mL, 16 µmol, 5 mol%) and oxalic acid (0.11 M, 285 µL, 32 µmol, 10 mol%), and 2,6-di-*tert*-butylpyridine **27** (3.5 µL, 16 µmol, 5 mol%) were reacted in 2-butanone (1.2 mL) for 3 hours at 60 °C. The reaction was concentrated under reduced pressure and analyzed by chiral HPLC to show no racemization in the presence of base.

The Effect of Water under the Reaction Conditions on Ether Reversibility

The effect of adding water to ether under the boronic acid reaction conditions was analyzed with 2 equiv. and 10 equiv. of the potential nucleophile. (Oxybis(ethane-1,1-diyl))dibenzene **28** (20 mg, 0.06 mmol, 1 equiv.), 2-carboxyphenylboronic acid **7** (0.015 M, 0.2 mL, 3 µmol, 5 mol%) and oxalic acid (0.11 M, 55 µL, 6 µmol, 10 mol%) were reacted in 2-butanone (0.25 mL) for 2 hours at room temperature. The reaction was diluted with ether and washed with 1 M NaOH and concentrated under reduced pressure. Conversion by ^{1}H NMR of the crude reaction mixture demonstrated that the relative ratio of ether:ROH was 72:28 and 73:27 for when 2 equiv. and 10 equiv. of water were added, respectively. This demonstrates that the reaction is not dependent on the amount of nucleophile suggesting an S_N1 process. This implies the reaction is occurring at the proton, not at the boron center.

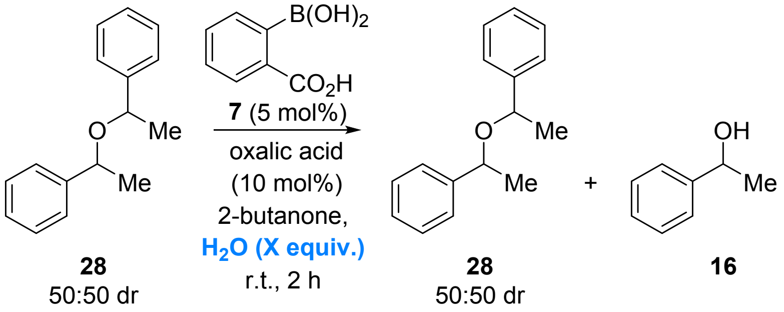

entry	H_2O (equiv.)	ether (**28**)	ROH (**16**)
1	2	72	28
2	10	73	27

NMR Studies of the Boron Complex

2-(4,5-Dioxo-1,3,2-dioxaborolan-2-yl)benzoic acid XX

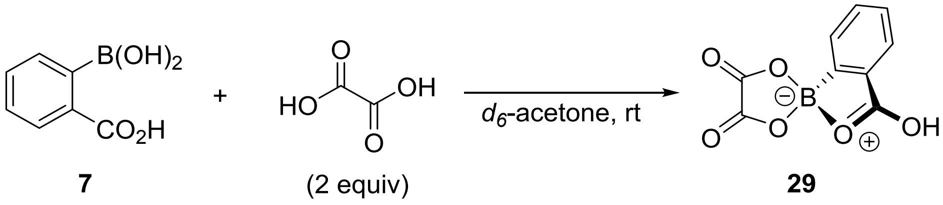

2-Carboxyphenylboronic acid **7** (17.0 mg, 0.102 mmol) and oxalic acid (18.0 mg, 0.200 mmol) were added to an NMR tube followed by d_6-acetone (0.80 mL). The tube was shaken vigorously for *ca.* 5 min until all solids were dissolved. **^{1}H NMR** (400 MHz, d_6-acetone) δ_H: 7.71–7.68 (m, 1H, ArC(6)*H*), 7.57–7.51 (m, 2H, ArC(4)*H*) and ArC(5)*H*)), 7.44–7.40 (m, 1H, ArC(3)*H*)); **^{13}C{^{1}H} NMR** (101 MHz, d_6-acetone, 238 K)[a] δ_C: 174.3 (CO$_2$H), 160.8 (C(O)C(O)), 150.1[b] (ArC(2)), 137.2 (ArC(1)), 133.4 (ArC(5)H), 129.5

(Ar*C*(3)H), 129.4 (Ar*C*(4)H), 124.6 (Ar*C*(6)H); **^{11}B{^{1}H} NMR** (128 MHz, *d_6*-acetone) δ_B: 9.62; **HRMS** (ESI$^-$) *m/z*: [M–H]$^-$ calculated for $C_9H_4BO_6$ 219.0215, found 219.0221 (+2.7 ppm).

[a] An additional peak at 159.8 ppm is observed for the excess, unbound oxalic acid **7**, which was confirmed by spiking the NMR sample.
[b] Signal not observed in spectrum due to quadrupolar boron atom, chemical shift identified by HMBC analysis.

The carboxylic acid carbon peak (δ_C 174.3 ppm) is not clearly visible in the ^{13}C{^{1}H} NMR at room temperature; however, the peak becomes more defined at lower temperatures. This observation may be consistent with a dynamic interaction between the carboxylic acid and the adjacent boron atom.

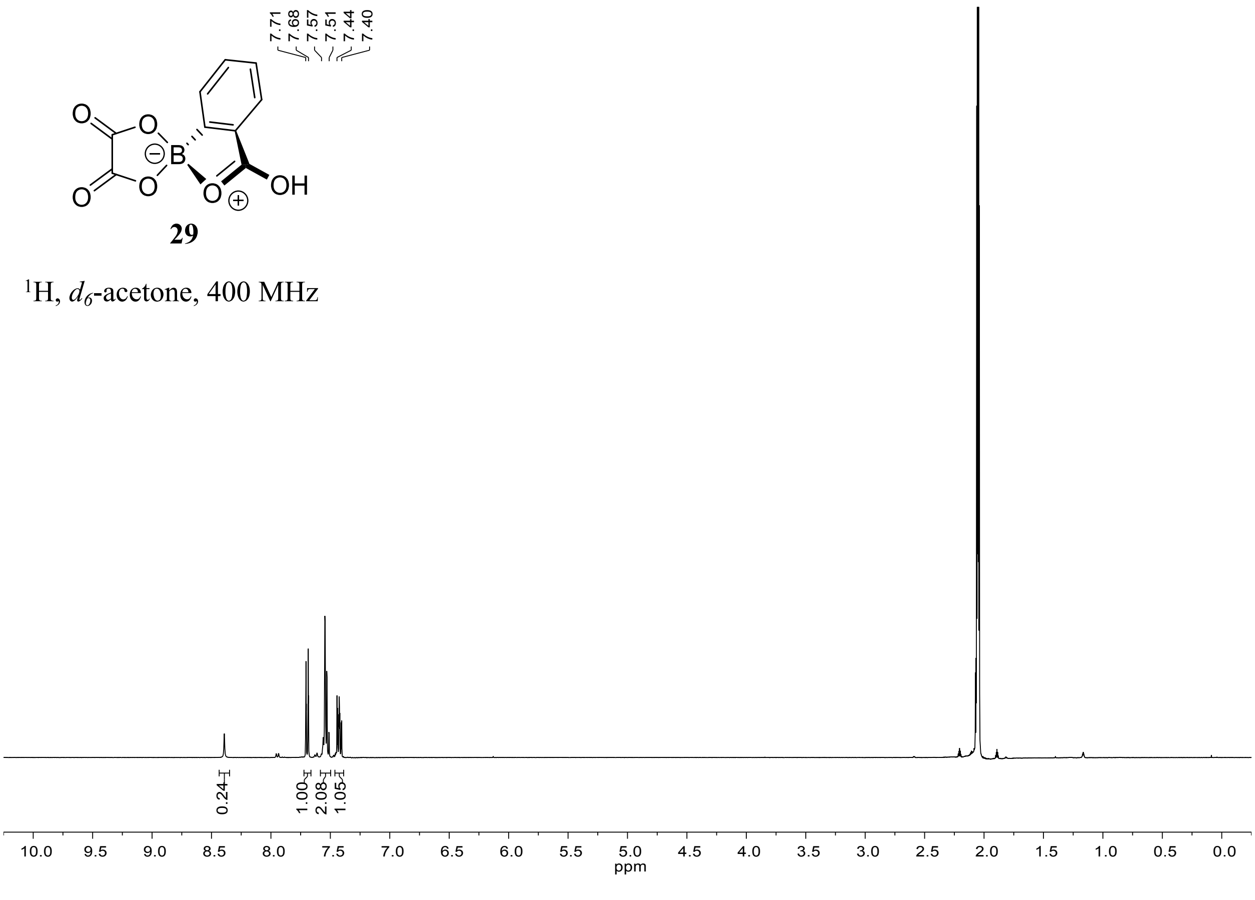

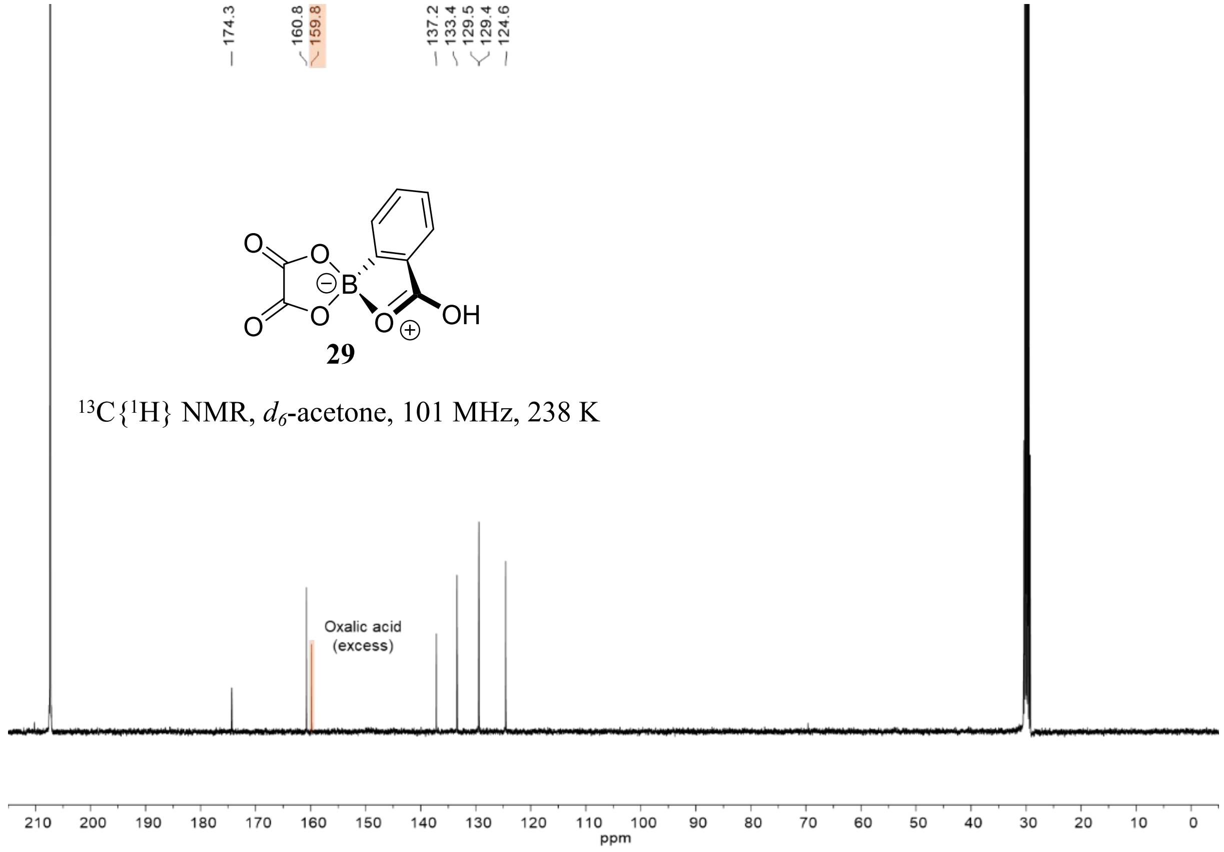

174.3
160.8
159.8
137.2
133.4
129.5
129.4
124.6
29
$^{13}C\{^{1}H\}$ NMR, d_6-acetone, 101 MHz, 238 K
Oxalic acid (excess)
ppm

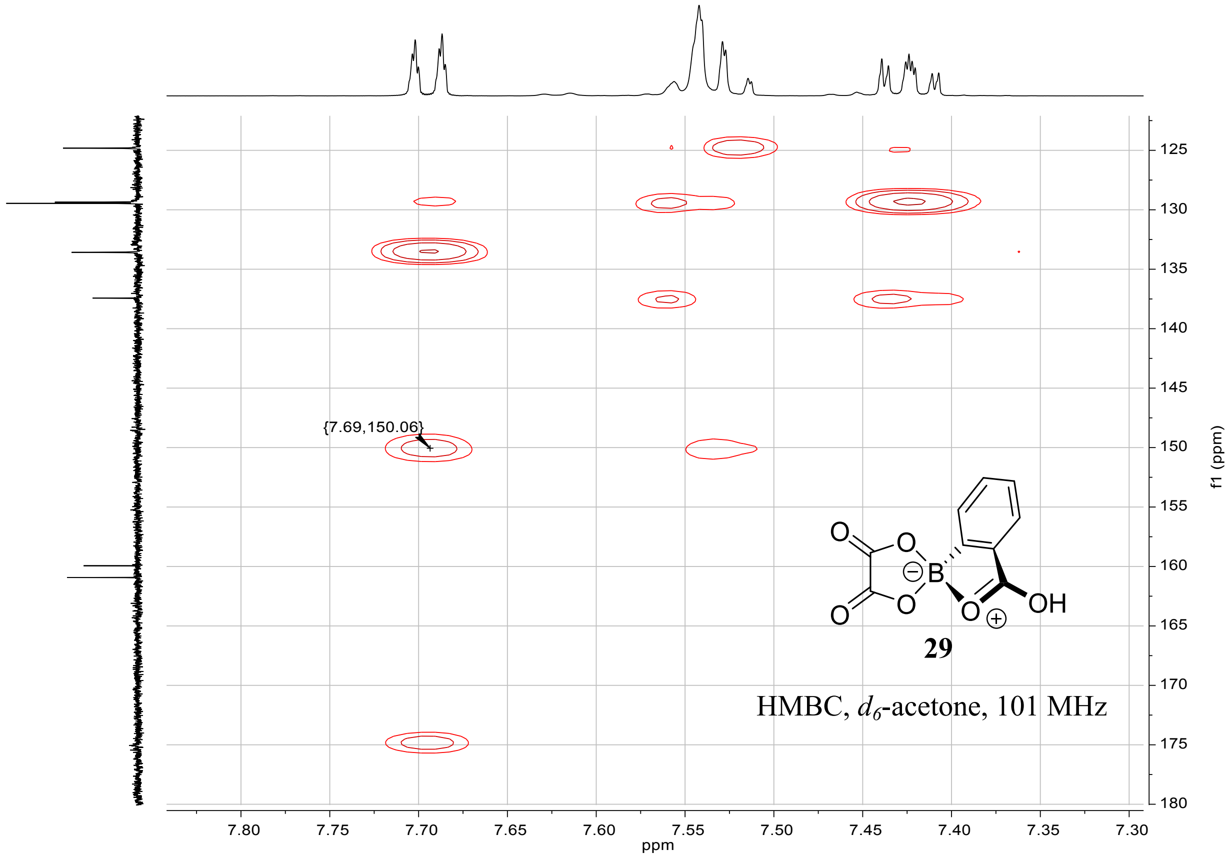

{7.69,150.06}
29
HMBC, d_6-acetone, 101 MHz
f1 (ppm)
ppm

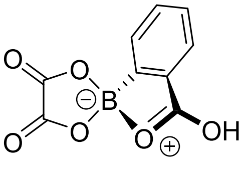

29

$^{11}B\{^{1}H\}$, d_6-acetone, 128 MHz

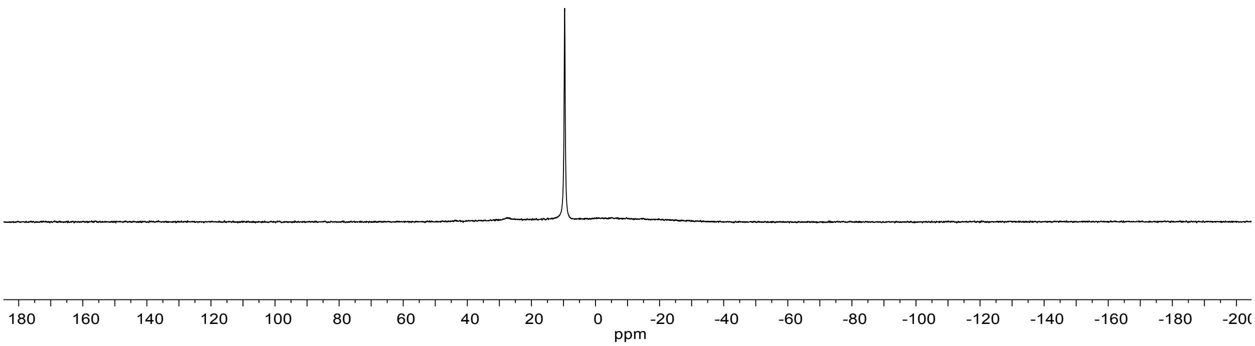

7. References

[1] Morrill, L. C.; Douglas, J.; Lebl, T.; Slawin, A. M. Z.; Fox, D. J.; Smith, A. D. *Chem. Sci.* **2013**, *4*, 4146–4155.

[2] Greenhalgh, M. D.; Smith, S. M.; Walden, D. M.; Taylor, J. E.; Brice, Z.; Robinson, E. R. T.; Fallan, C.; Cordes, D. B.; Slawin, A. M. Z.; Richardson, H. C.; Grove, M. A.; Cheong, P. H.-Y.; Smith, A. D. *Angew. Chem., Int. Ed.* **2018**, *57*, 3200–3206.

[3] Guha, N. R.; Neyyappadath, R. M.; Greenhalgh, M. D.; Chisholm, R.; Smith, S. M.; McEvoy, M. L.; Young, C. M.; Rodríguez-Escrich, C.; Pericas, M. A.; Hähner, G.; Smith, A. D. *Green Chem.* **2018**, *20*, 4537–4546.

[4] Zhang, Y.-Y.; Chen, H.; Jiang, X.; Liang, H.; He, X.; Zhang, Y.; Chen, X.; He, W.; Li, Y.; Qiu, L. *Tetrahedron* **2018**, *74*, 2245−2250.

[5] Gorokhovik, I; Neuville, L.; Zhu, J. *Org. Lett.* **2011**, *13*, 5536–5539.

[6] Li, Q.; Wan, P.; Wang, S.; Zhuang, Y.; Li, L.; Zhou, Y.; He, Y.; Cao, R.; Qiu, L.; Zhou, Z. Appl. Catal., A **2013**, *458*, 201−206.

[7] Lu, S.; Poh, S. B.; Siau, W.-Y.; Zhao, Y. *Angew. Chem., Int. Ed.* **2013**, *52*, 1731−1734.

[8] Neyyappadath, R. M.; Chisholm, R.; Greenhalgh, M. D.; Rodríguez-Escrich, C.; Pericas, M. A.; Hähner, G.; Smith, A. D. *ACS Catal.* **2018**, *8*, 1067.

[9] Wang, L.; Lin, J.; Sun, Q.; Xia, C.; Sun, W. *ACS Catal.* **2021**, *11*, 8033–8041.

[10] Hu, B.; Meng, M.; Fossey, J. S.; Mo, W. M.; Hu, X. Q.; Deng, W. P. *Chem. Commun.* **2011**, *47*, 10632–10634.

[11] Díaz-Valenzuela, M. B.; Phillips, S. D.; France, M. B.; Gunn, M. E.; Clarke, M. L. *Chem; Eur. J.* **2009**, *15*, 1227–1232.

[12] Birman, B. V.; Li X. *Org. Lett.* **2006**, *8*, 1351–1354.

8. Appendix 1: NMR Spectra

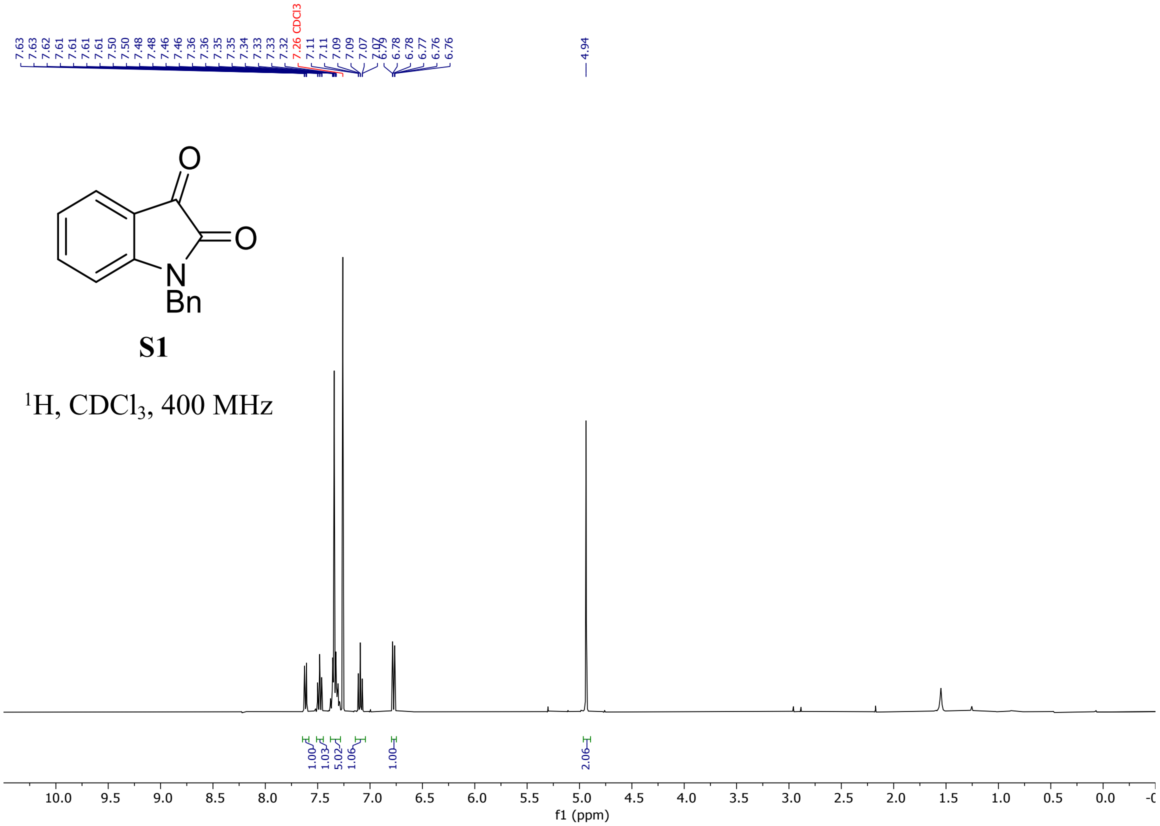

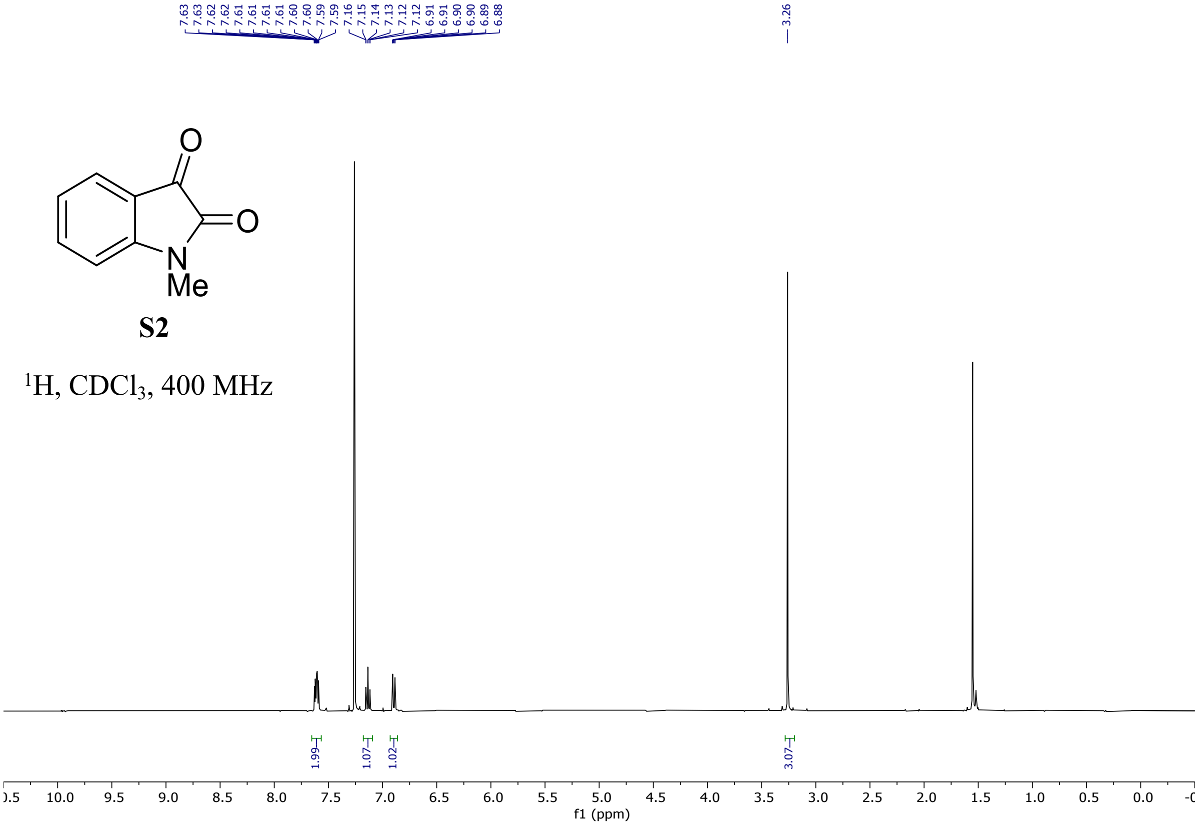

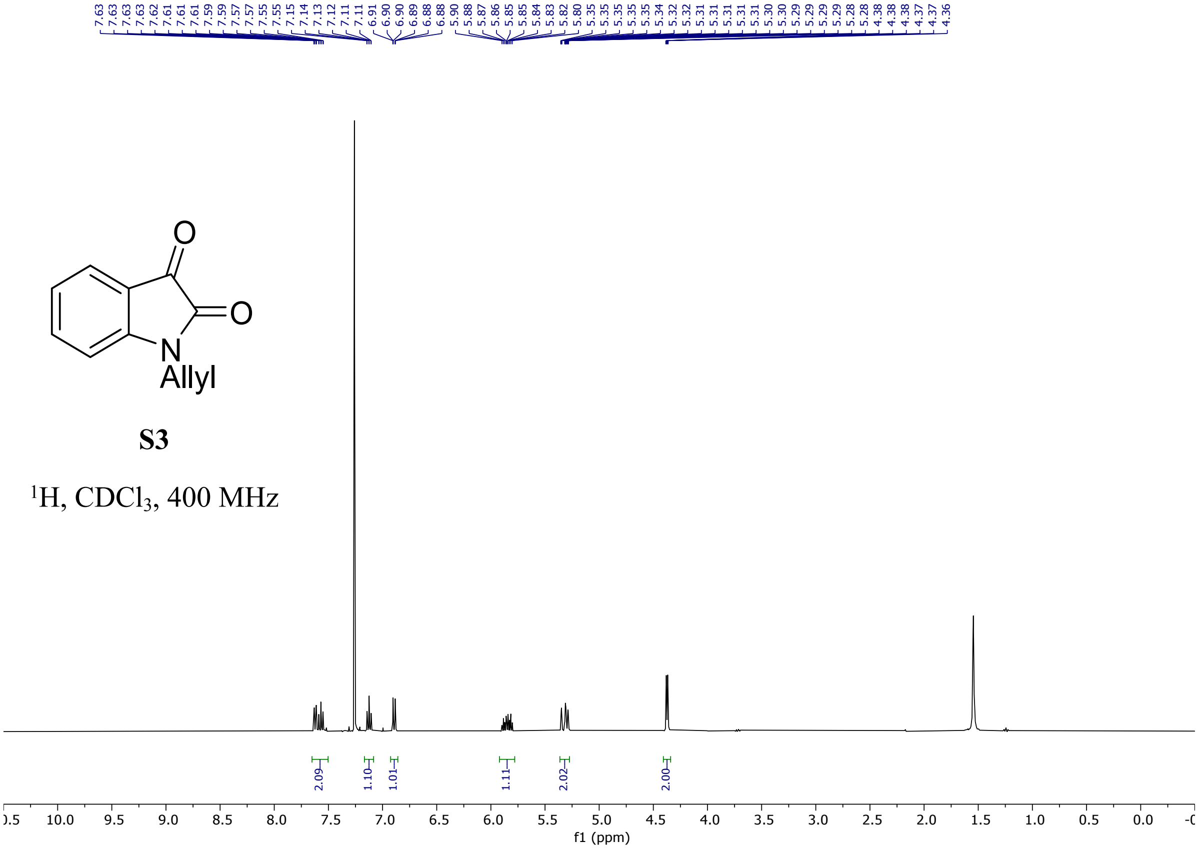

S3

^{1}H, $CDCl_3$, 400 MHz

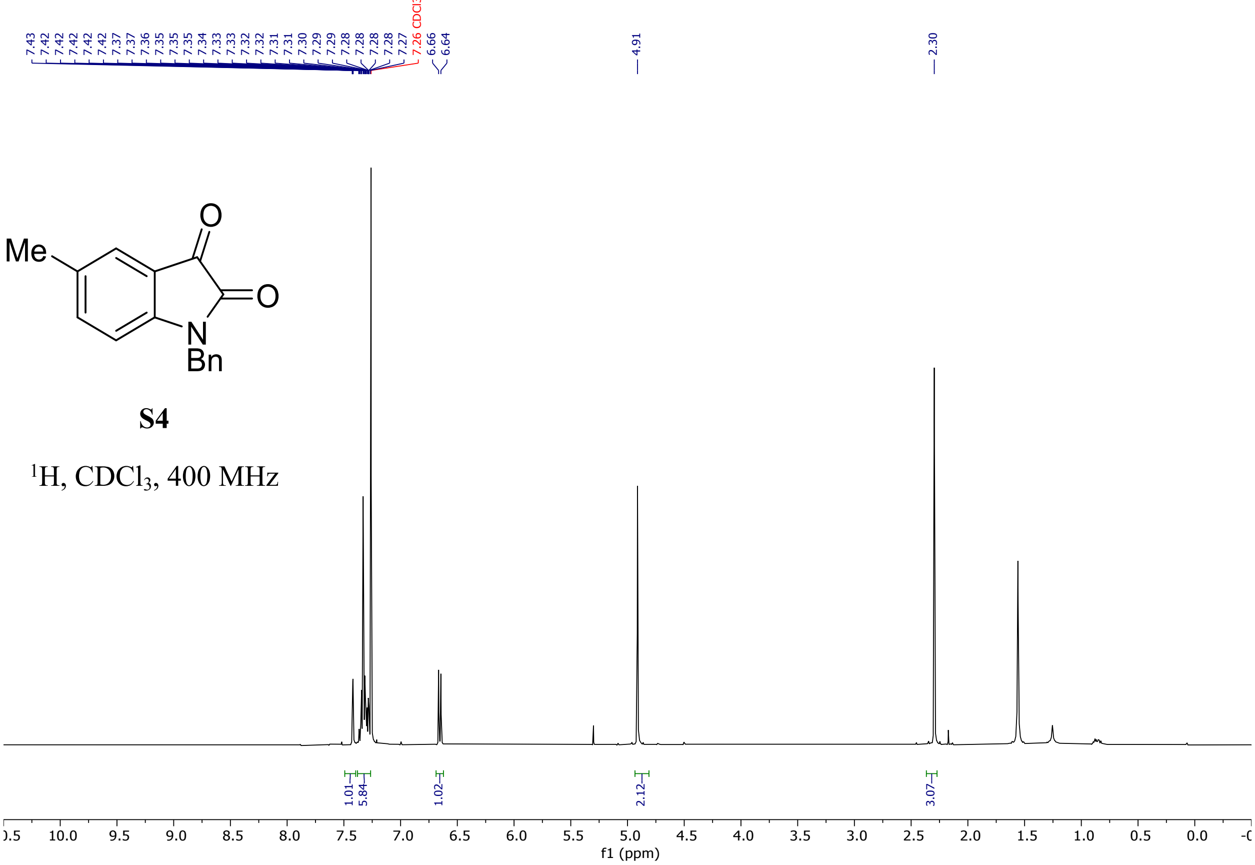

S4

^{1}H, $CDCl_3$, 400 MHz

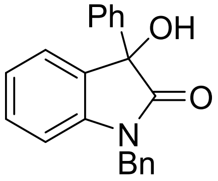

1

^{1}H, $CDCl_3$, 400 MHz

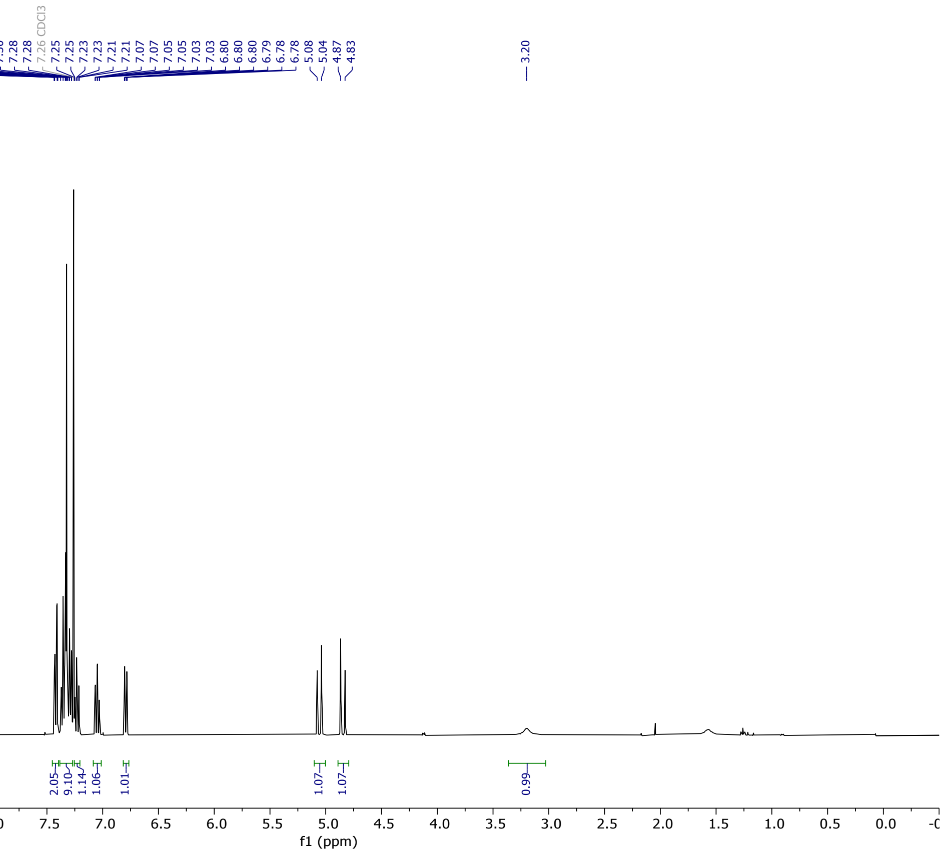

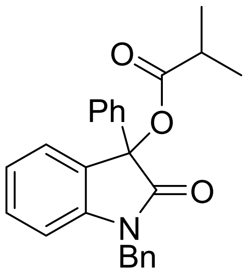

S5

^{1}H, $CDCl_3$, 400 MHz

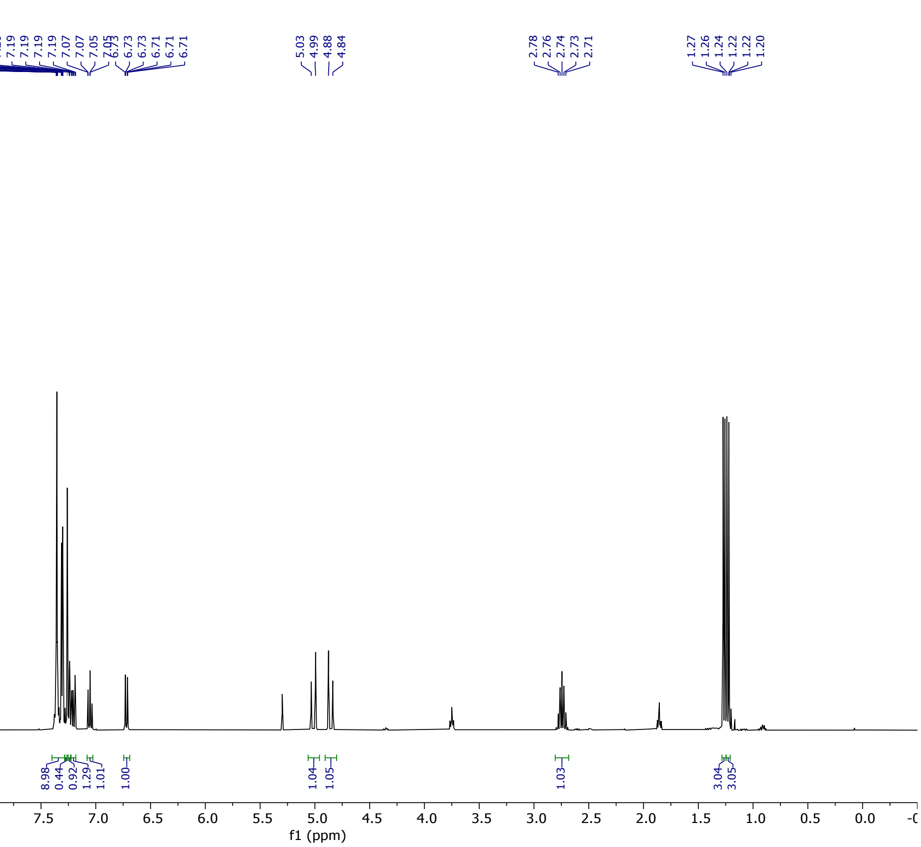

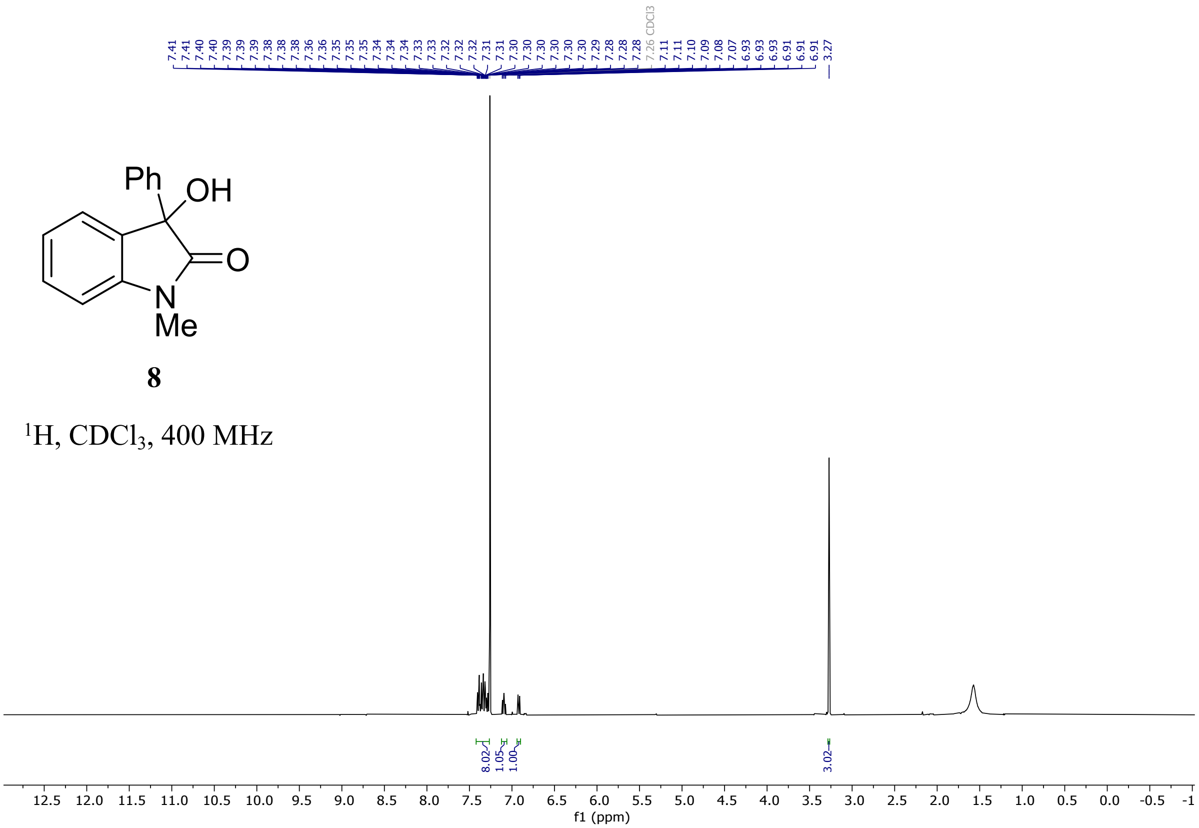

8

^{1}H, $CDCl_3$, 400 MHz

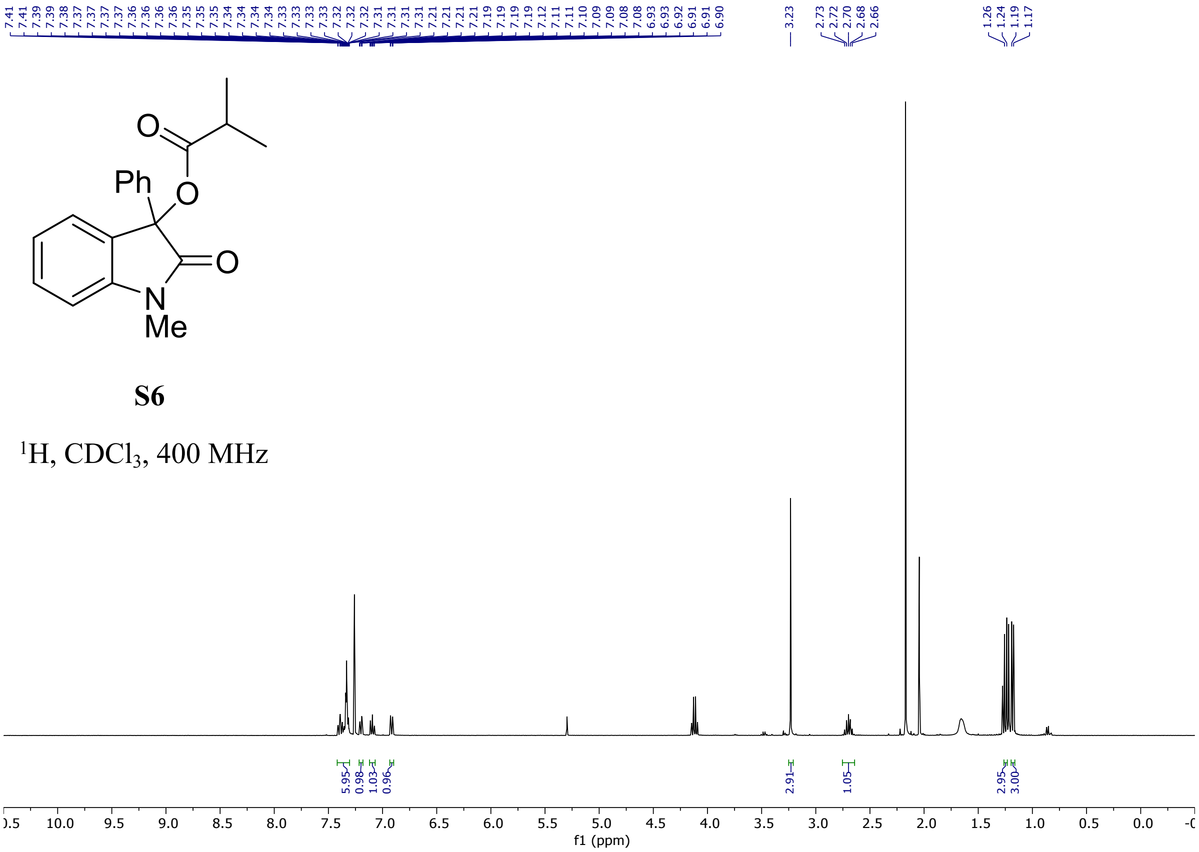

S6

^{1}H, $CDCl_3$, 400 MHz

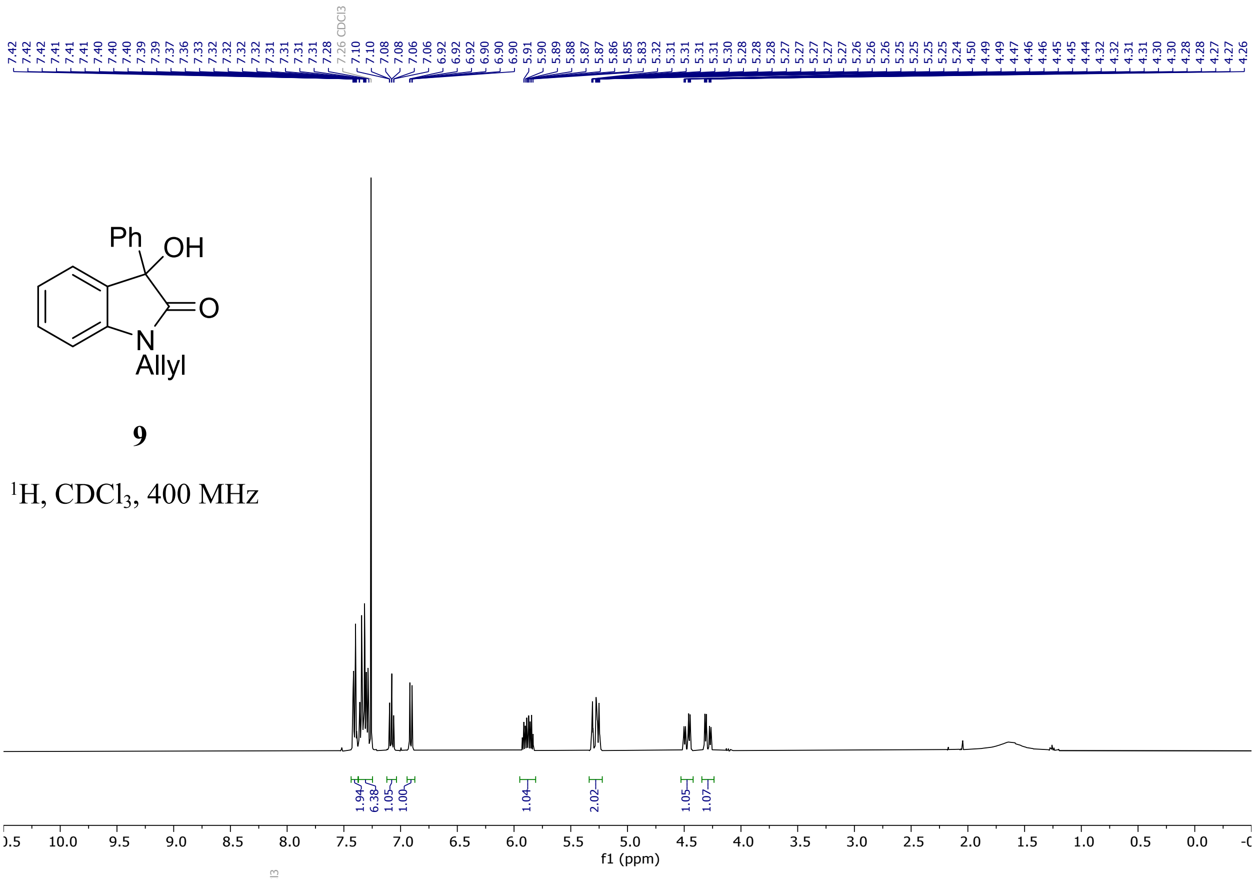

9

^{1}H, $CDCl_3$, 400 MHz

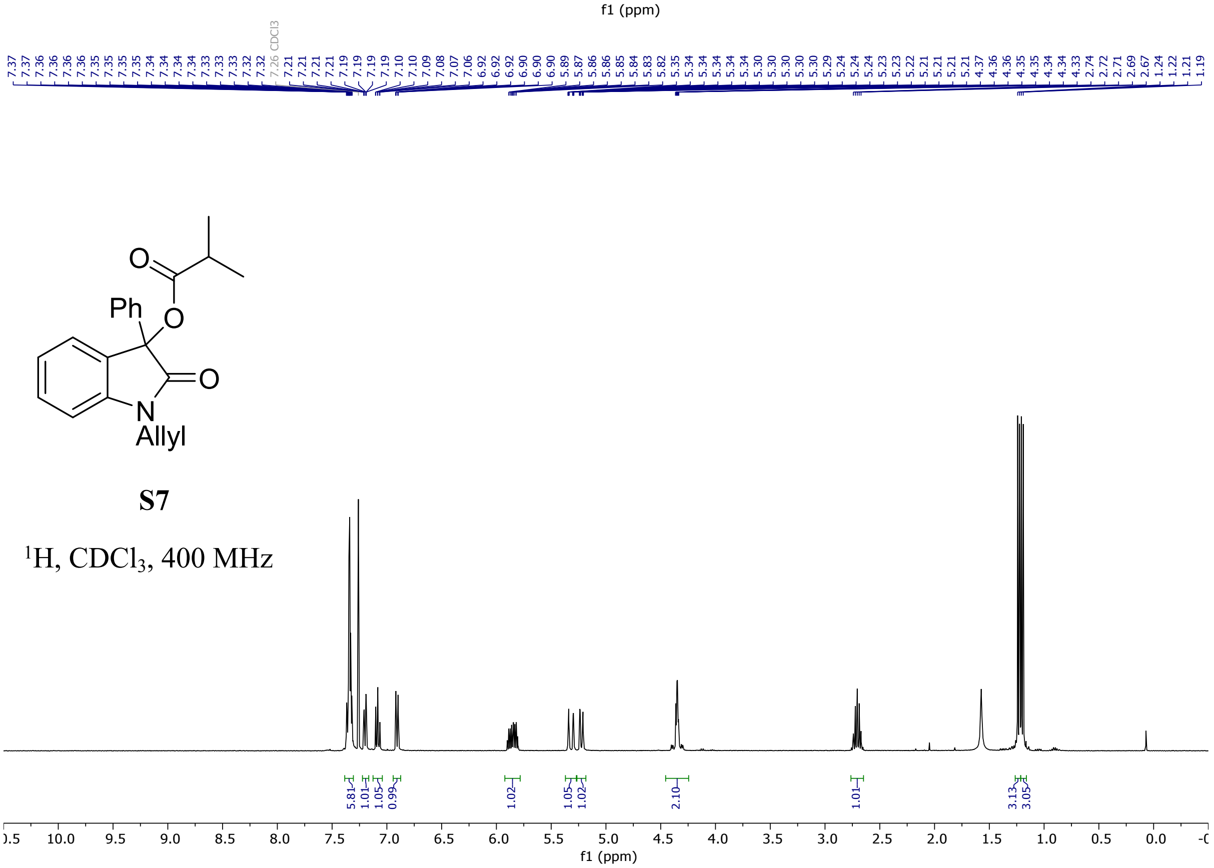

S7

^{1}H, $CDCl_3$, 400 MHz

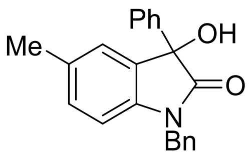

10

^{1}H, $CDCl_3$, 400 MHz

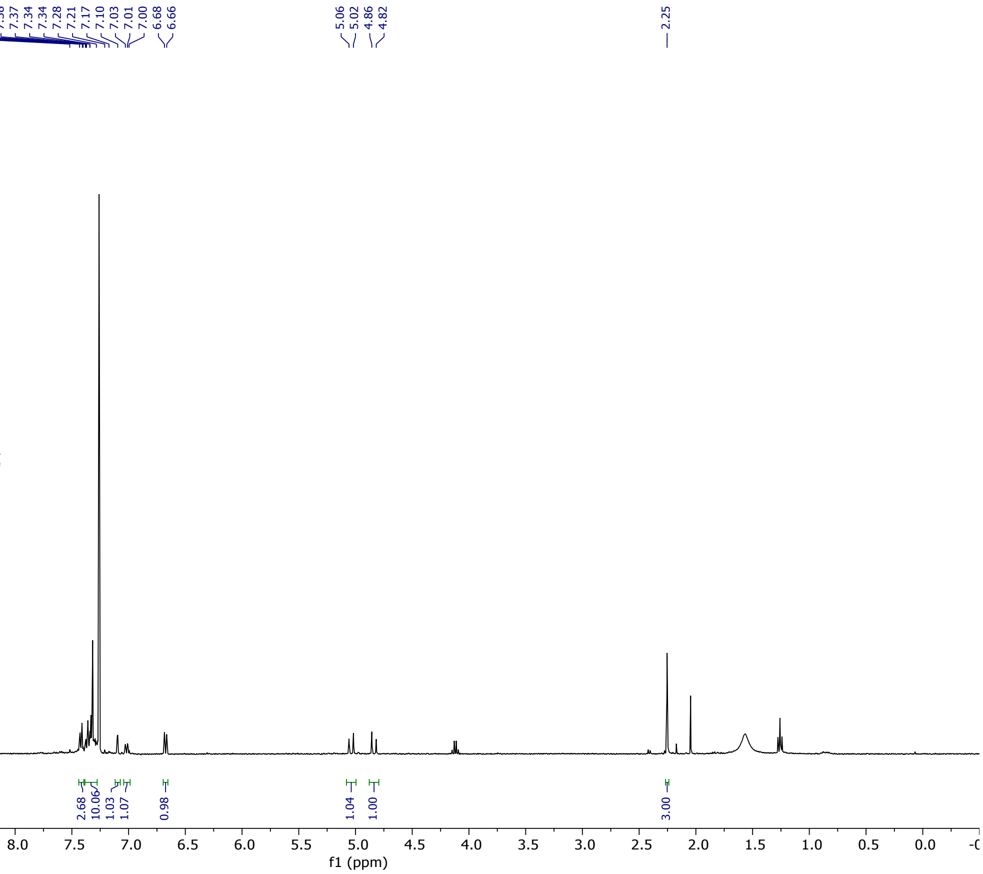

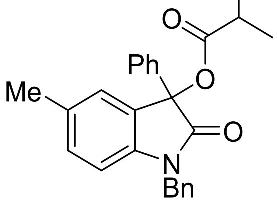

S8

^{1}H, $CDCl_3$, 400 MHz

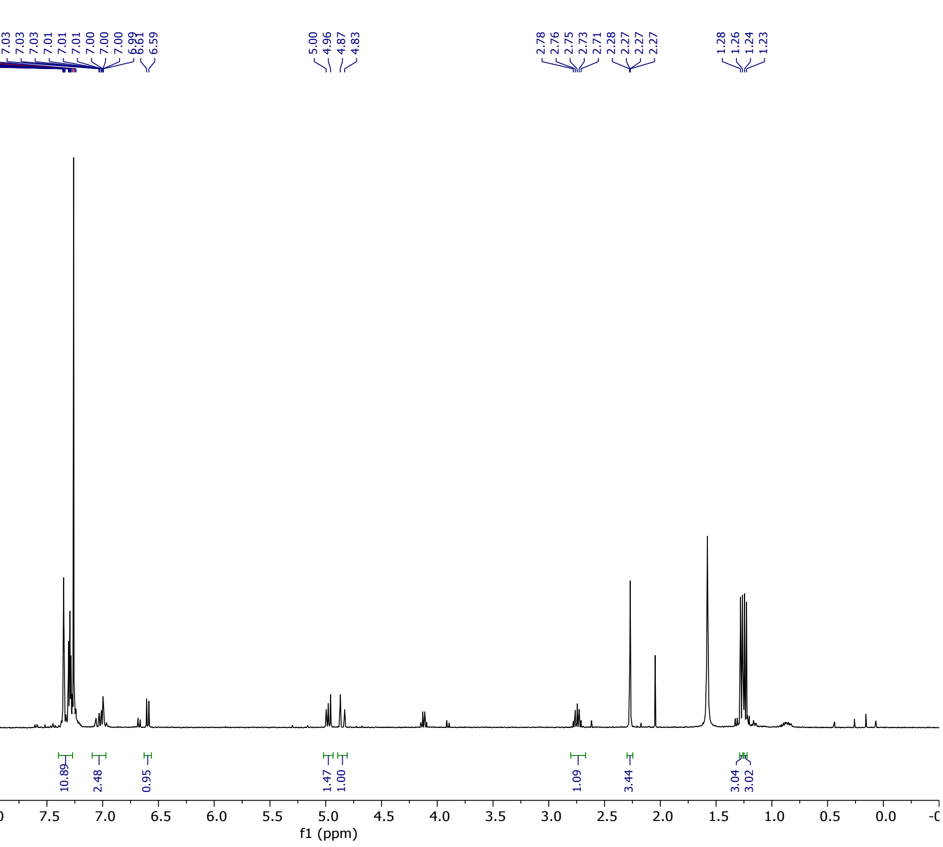

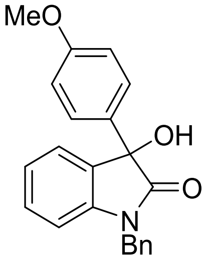

11

^{1}H, $CDCl_3$, 400 MHz

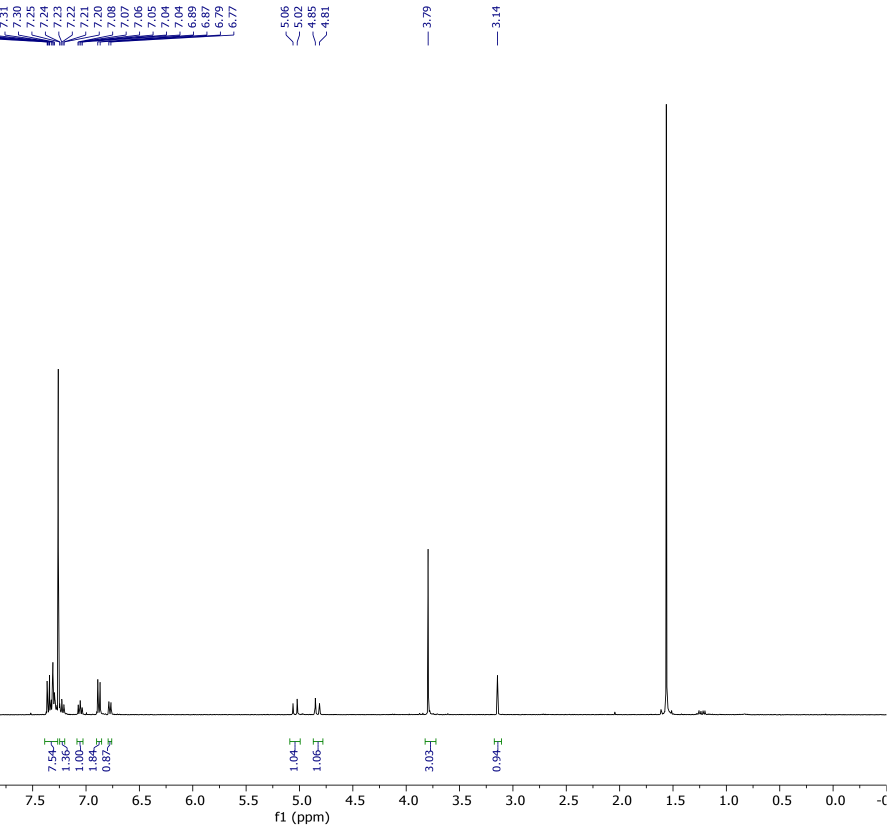

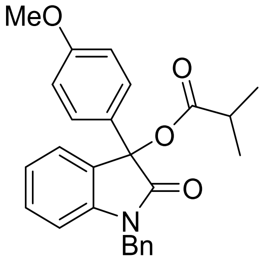

S9

^{1}H, $CDCl_3$, 400 MHz

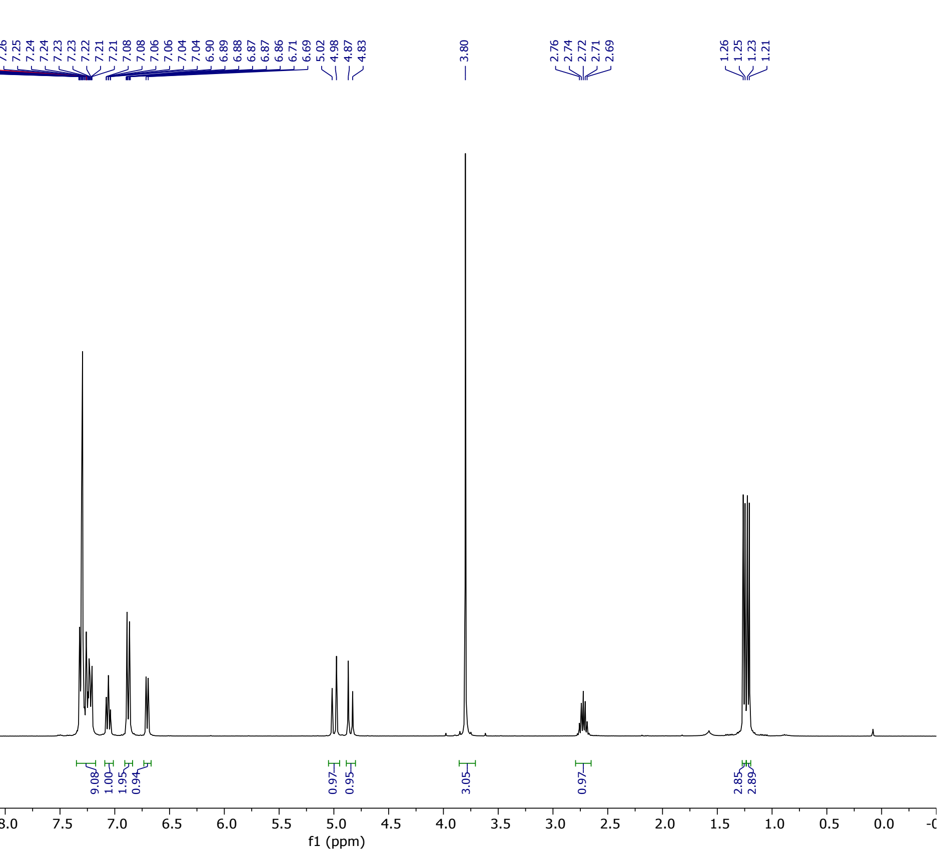

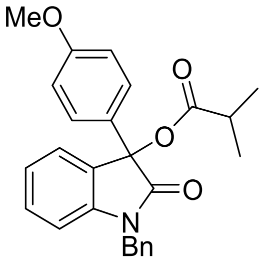

S9

^{13}C, $CDCl_3$, 101 MHz

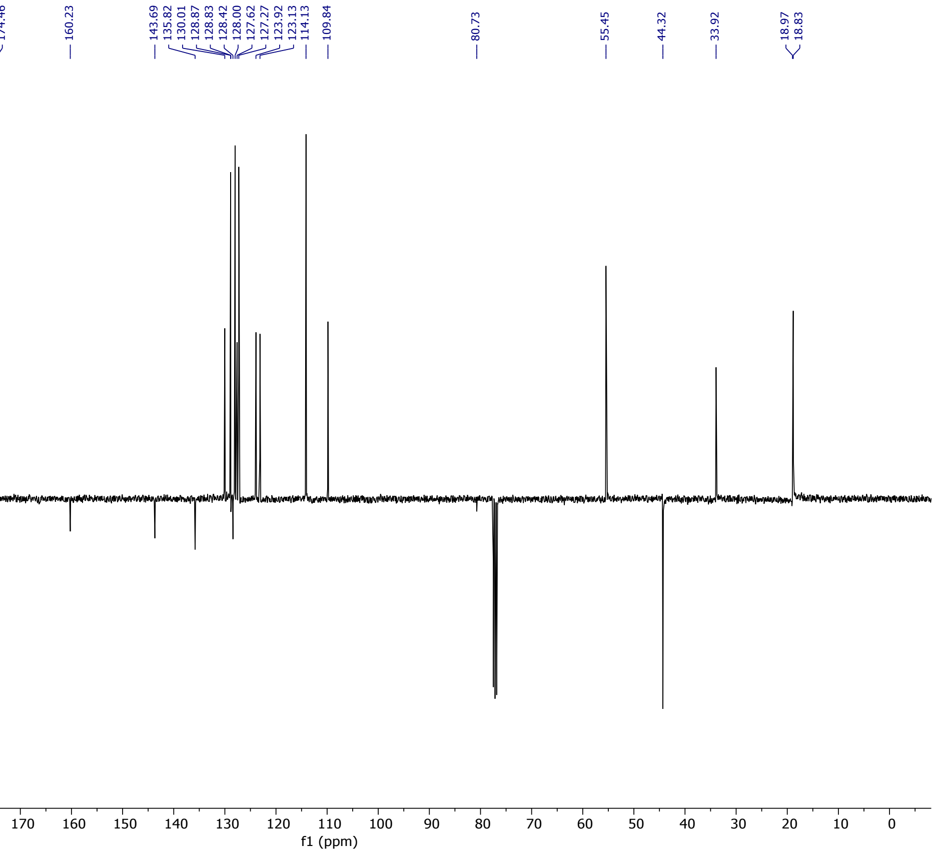

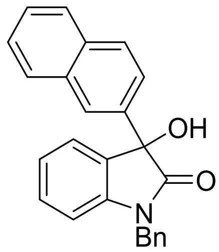

12

^{1}H, $CDCl_3$, 400 MHz

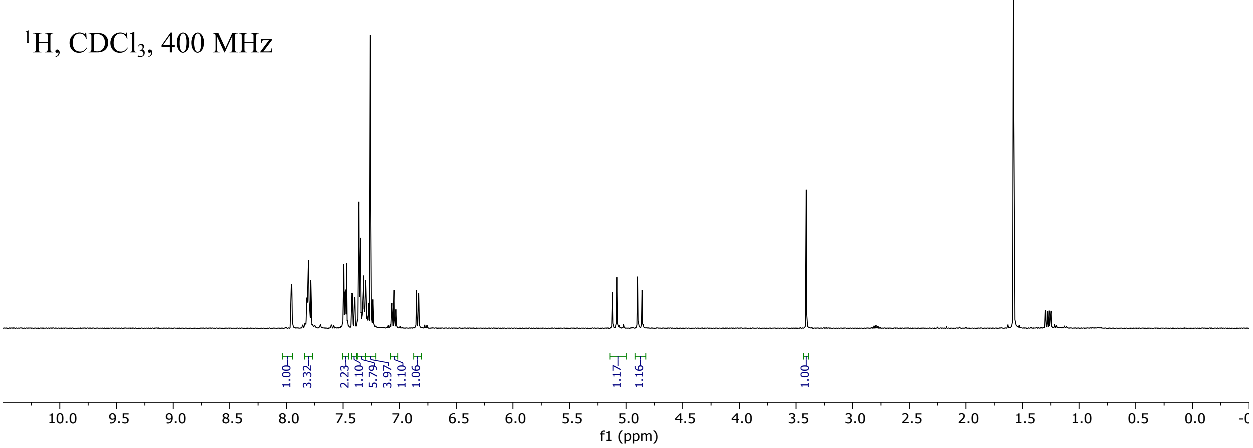

S10

^1H, $CDCl_3$, 400 MHz

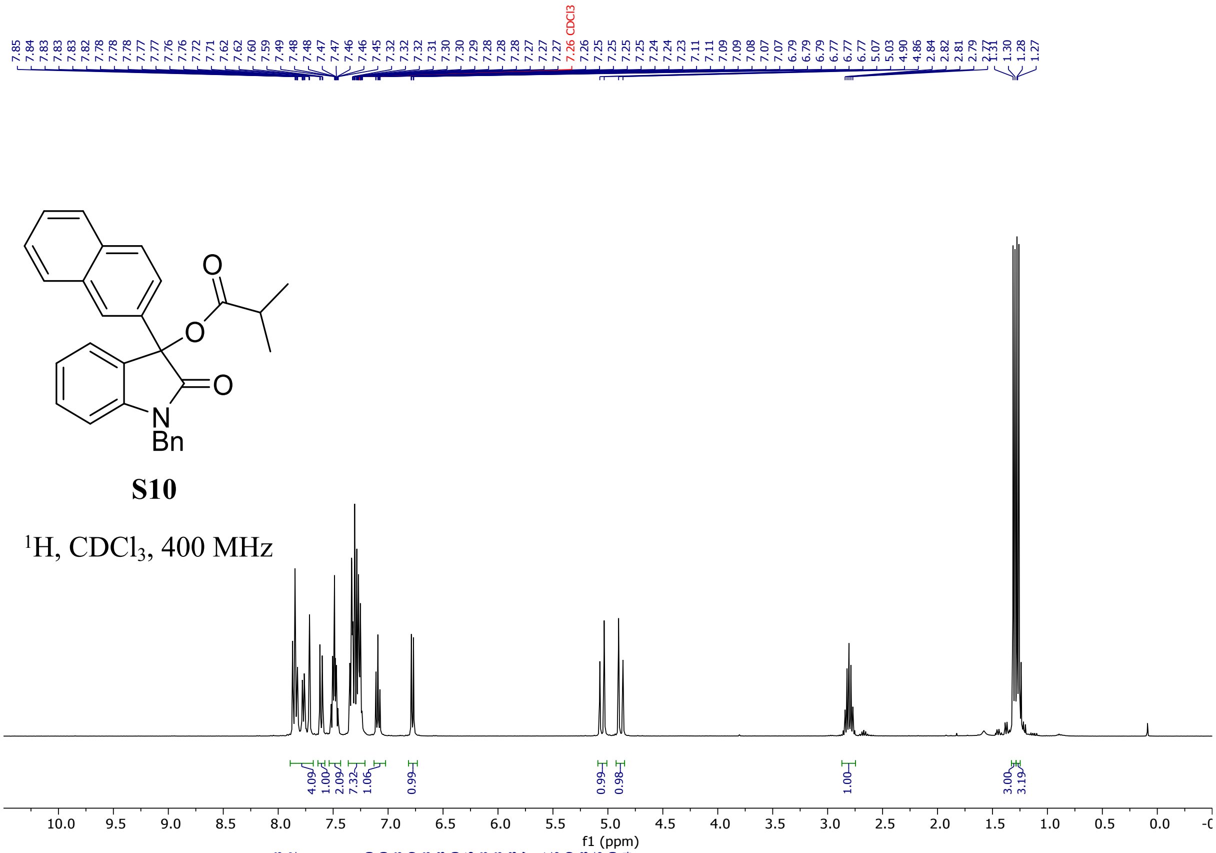

S10

^{13}C, $CDCl_3$, 101 MHz

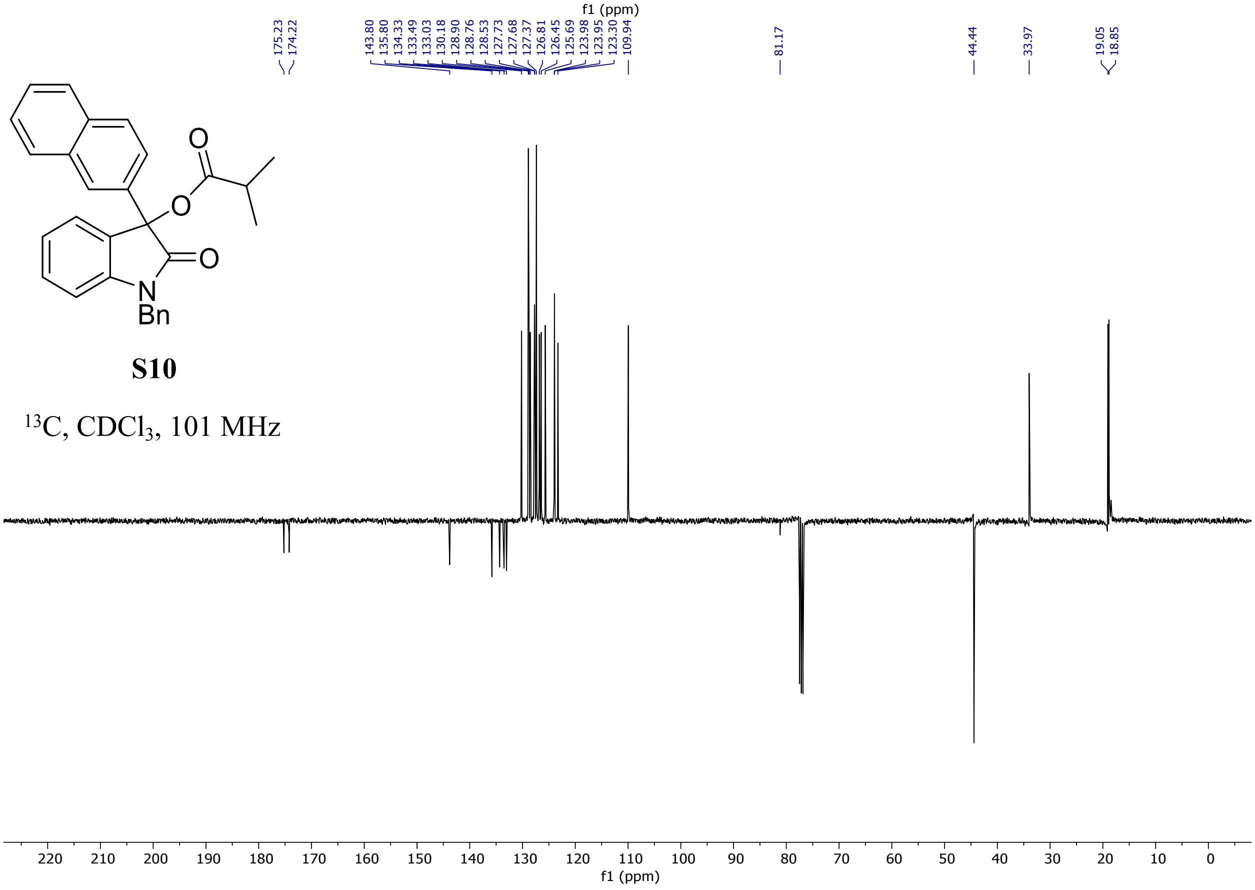

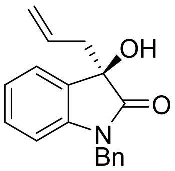

13

^1H, $CDCl_3$, 500 MHz

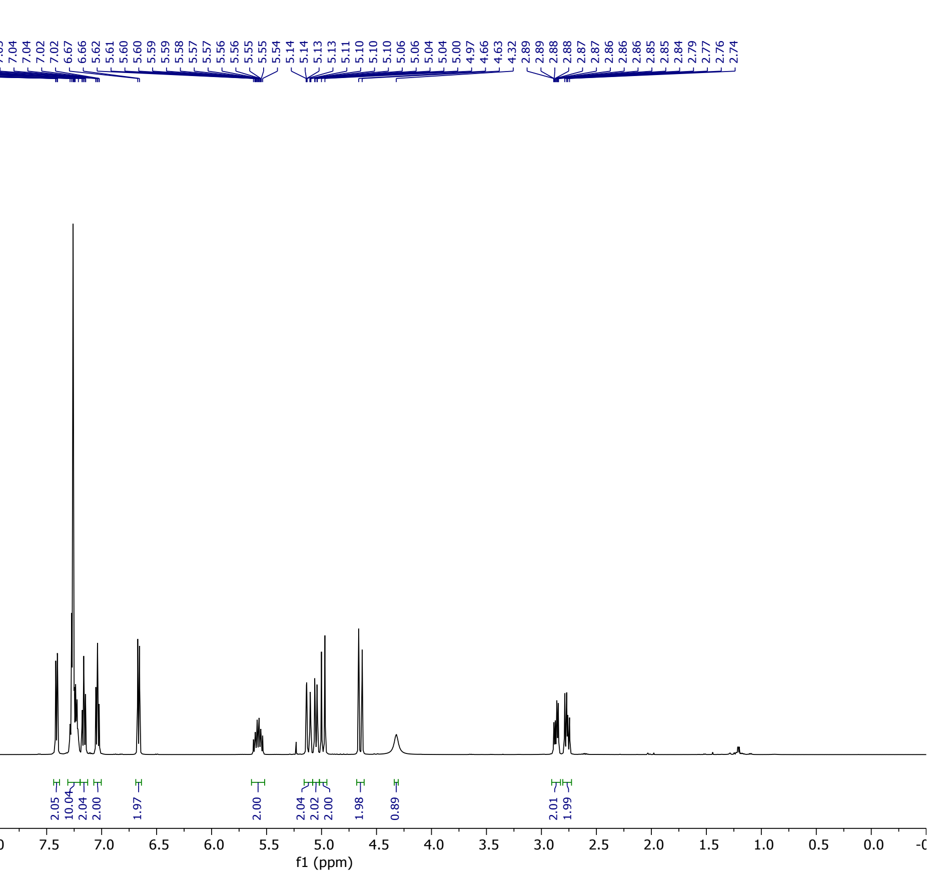

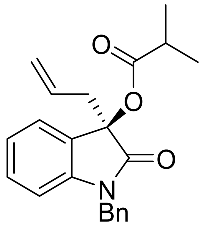

S11

^1H, $CDCl_3$, 500 MHz

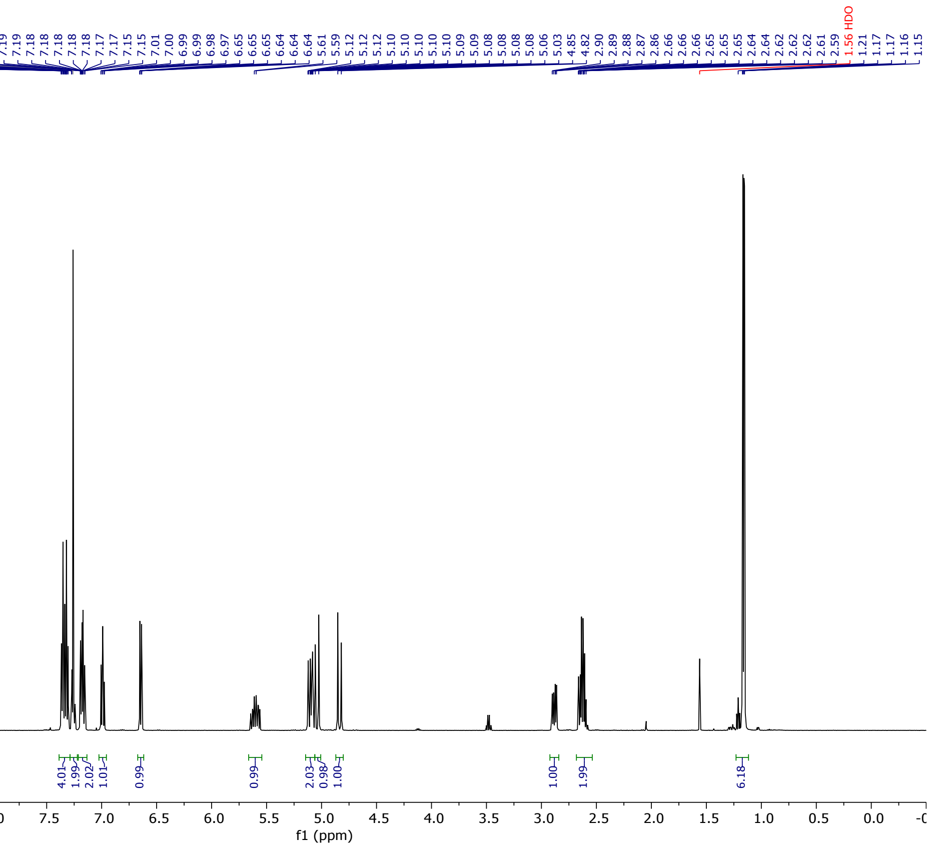

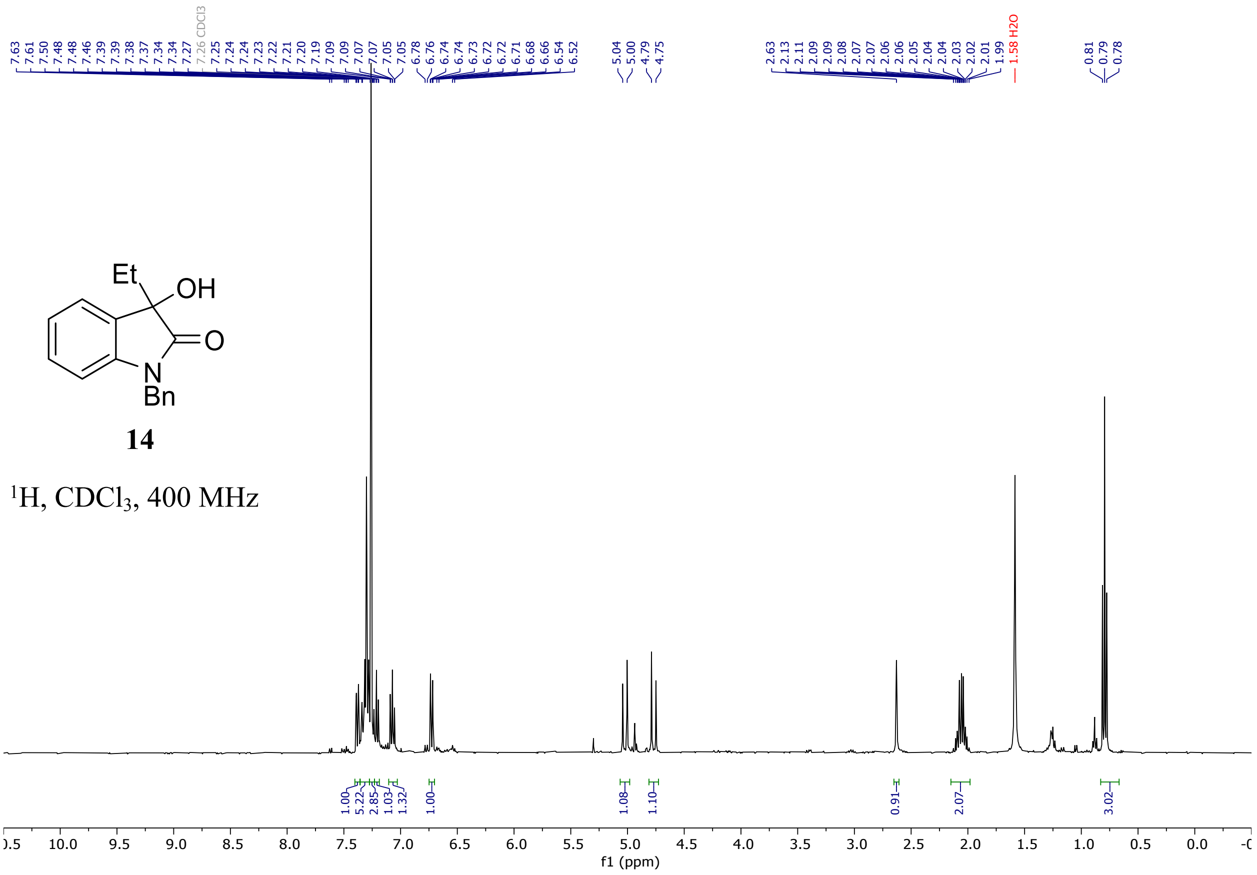

Et
OH
O
N
Bn
14
¹H, CDCl₃, 400 MHz
7.63
7.61
7.50
7.48
7.48
7.46
7.39
7.39
7.38
7.37
7.34
7.34
7.27
7.26 CDCl3
7.25
7.24
7.24
7.23
7.22
7.21
7.20
7.19
7.09
7.09
7.07
7.07
7.05
7.05
6.78
6.76
6.74
6.74
6.73
6.72
6.72
6.71
6.68
6.66
6.54
6.52
5.04
5.00
4.79
4.75
2.63
2.13
2.11
2.09
2.09
2.08
2.07
2.07
2.06
2.06
2.05
2.04
2.04
2.03
2.02
2.01
1.99
1.58 H2O
0.81
0.79
0.78
1.00
5.22
2.85
1.03
1.32
1.00
1.08
1.10
0.91
2.07
3.02
f1 (ppm)

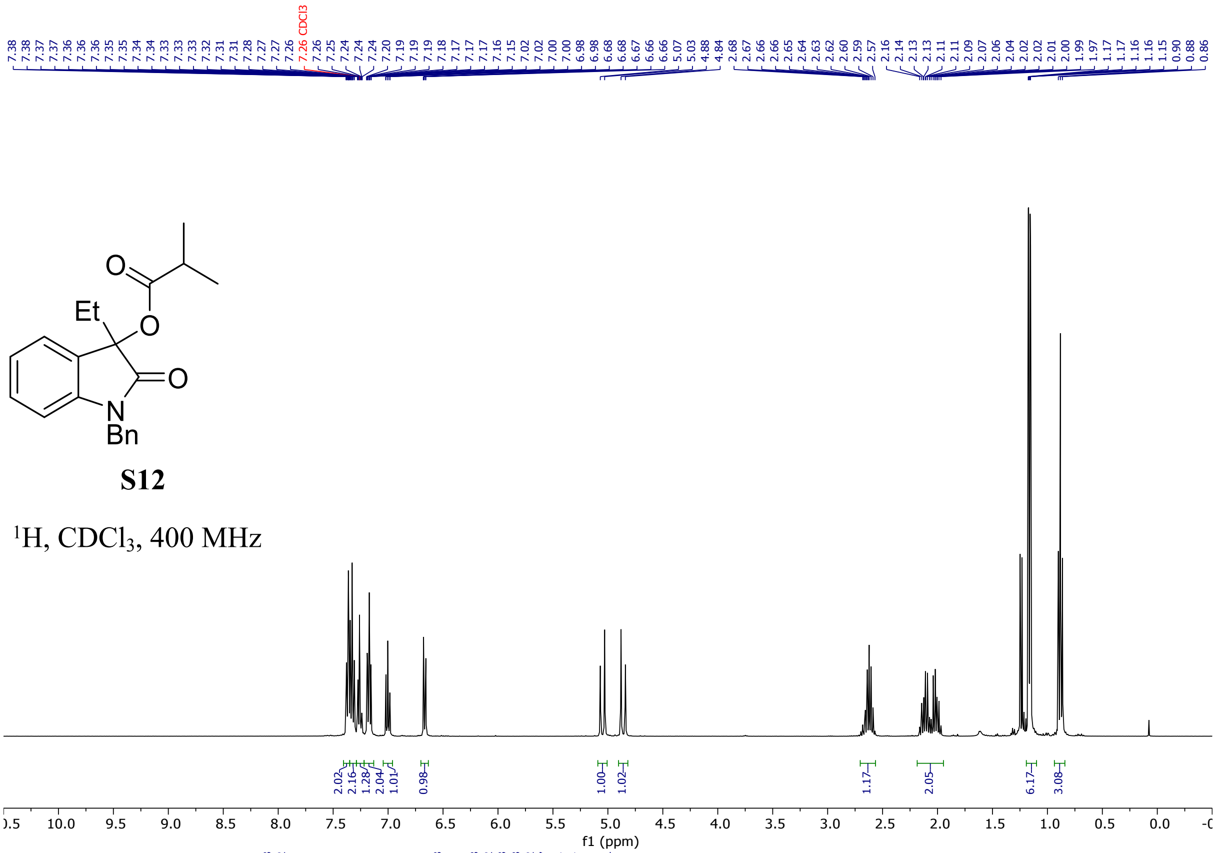
7.38 7.38 7.37 7.36 7.36 7.35 7.35 7.34 7.34 7.33 7.33 7.33 7.32 7.31 7.31 7.28 7.27 7.27 7.26 7.26 CDCl3 7.26 7.25 7.24 7.24 7.24 7.20 7.19 7.19 7.18 7.17 7.17 7.17 7.16 7.15 7.02 7.02 7.00 7.00 6.98 6.98 6.68 6.68 6.67 6.66 6.66 5.07 5.03 4.88 4.84 2.68 2.67 2.66 2.66 2.65 2.64 2.63 2.62 2.60 2.59 2.57 2.16 2.14 2.13 2.13 2.11 2.11 2.09 2.07 2.06 2.04 2.02 2.02 2.01 2.00 1.99 1.97 1.17 1.17 1.16 1.16 1.15 0.90 0.88 0.86
Et
O
O
O
N
Bn
S12
^{1}H, $CDCl_3$, 400 MHz
2.02 2.16 1.28 2.04 1.01 0.98 1.00 1.02 1.17 2.05 6.17 3.08
f1 (ppm)

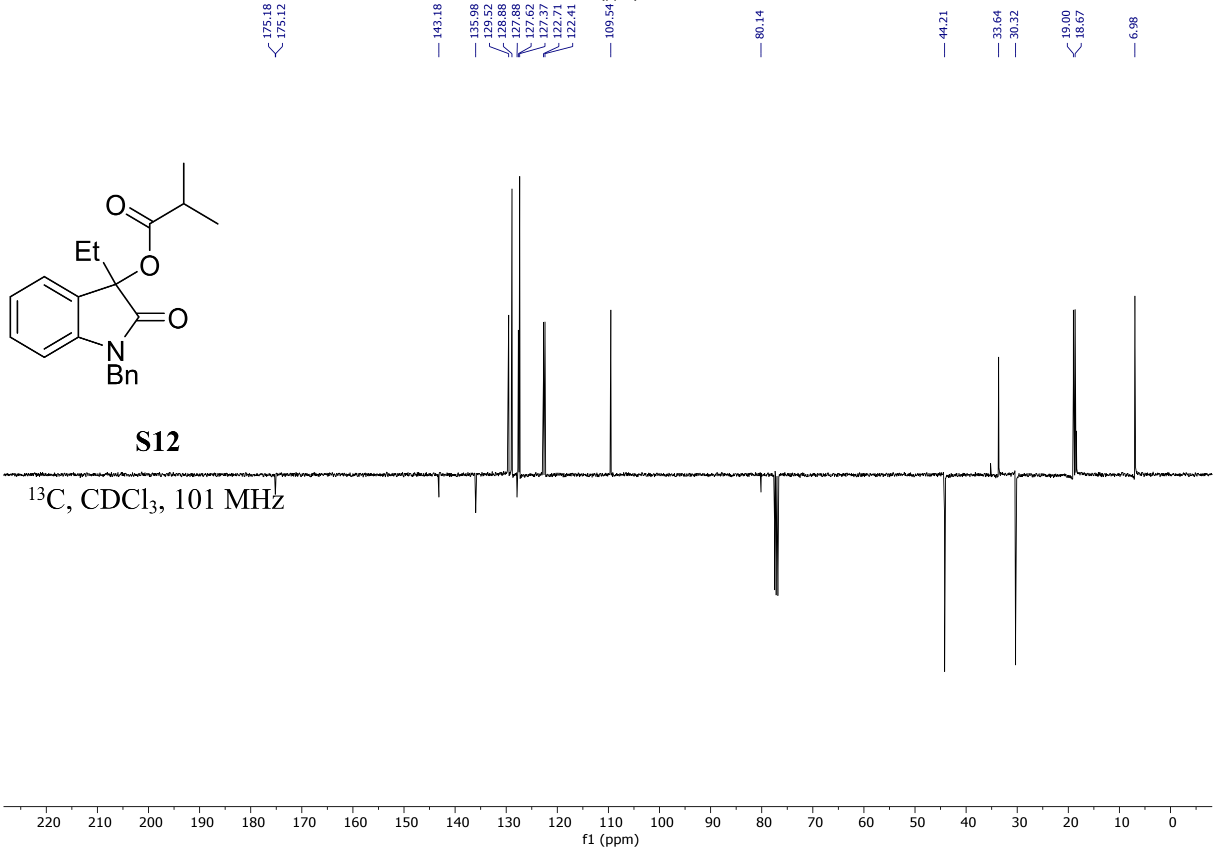
175.18 175.12 143.18 135.98 129.52 128.88 127.88 127.62 127.37 122.71 122.41 109.54 80.14 44.21 33.64 30.32 19.00 18.67 6.98
Et
O
O
O
N
Bn
S12
^{13}C, $CDCl_3$, 101 MHz
f1 (ppm)

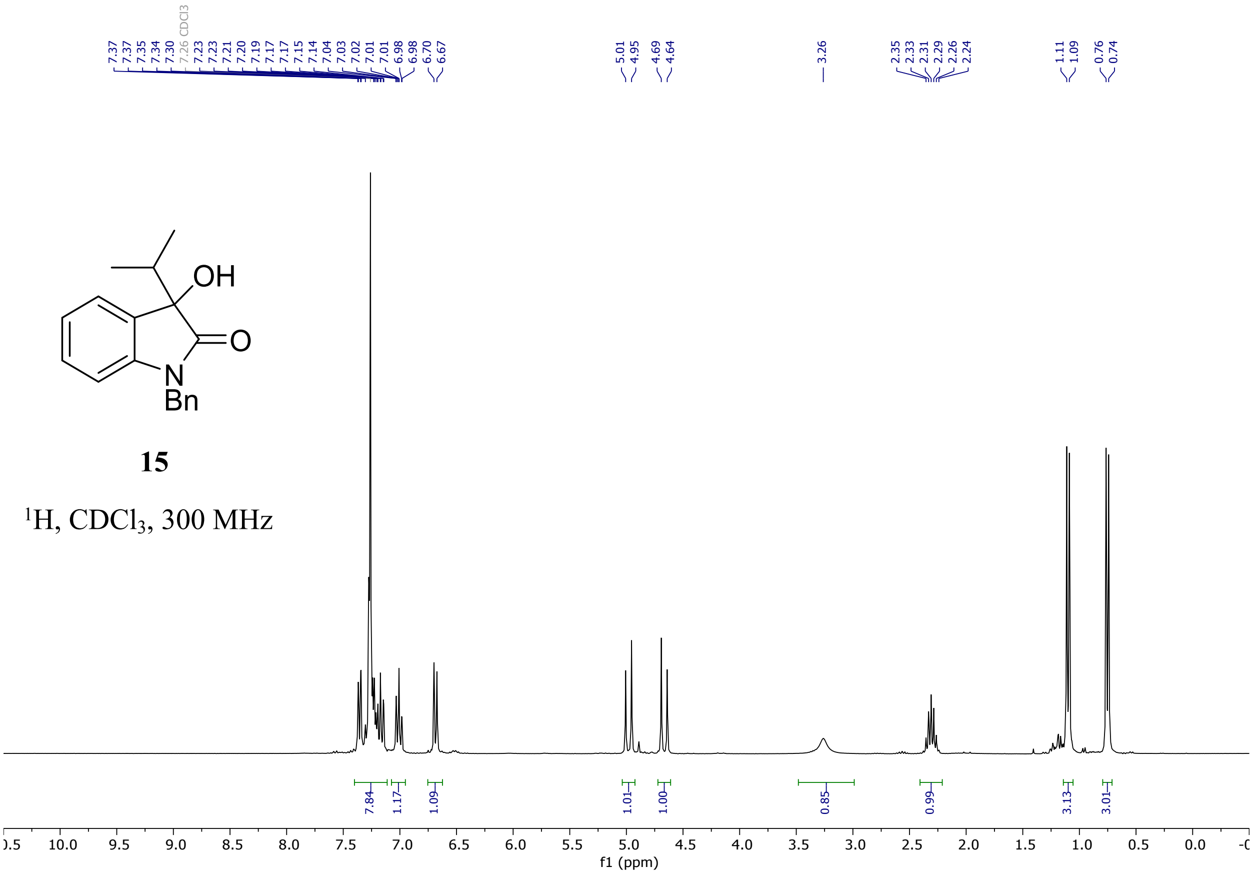
7.37 7.37 7.35 7.34 7.30 7.26 CDCl3 7.23 7.23 7.21 7.20 7.19 7.17 7.17 7.15 7.14 7.04 7.03 7.02 7.01 7.01 6.98 6.98 6.70 6.67
5.01 4.95 4.69 4.64
3.26
2.35 2.33 2.31 2.29 2.26 2.24
1.11 1.09 0.76 0.74
OH
O
N
Bn
15
¹H, CDCl₃, 300 MHz
7.84 1.17 1.09 1.01 1.00 0.85 0.99 3.13 3.01
f1 (ppm)

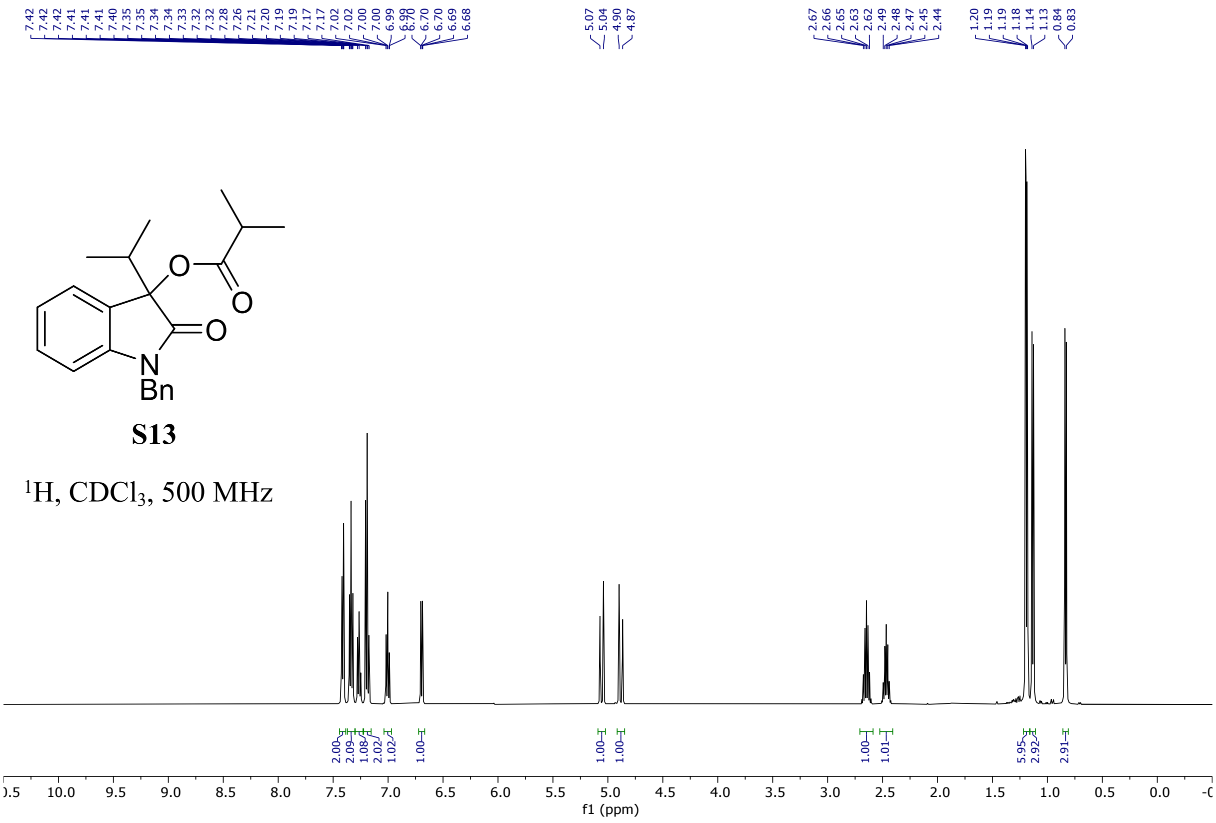
7.42 7.42 7.42 7.41 7.41 7.40 7.35 7.35 7.34 7.34 7.33 7.32 7.32 7.28 7.26 7.21 7.20 7.19 7.19 7.17 7.17 7.02 7.02 7.00 6.99 6.98 6.70 6.70 6.69 6.68
5.07 5.04 4.90 4.87
2.67 2.66 2.65 2.63 2.62 2.49 2.48 2.47 2.45 2.44
1.20 1.19 1.19 1.18 1.14 1.13 0.84 0.83
O
O
O
N
Bn
S13
¹H, CDCl₃, 500 MHz
2.00 2.09 1.08 2.02 1.02 1.00 1.00 1.00 1.00 1.01 5.95 2.92 2.91
f1 (ppm)

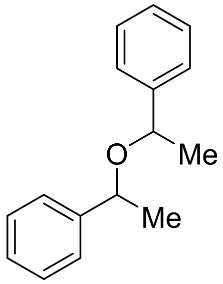

28

^{1}H, $CDCl_3$, 500 MHz

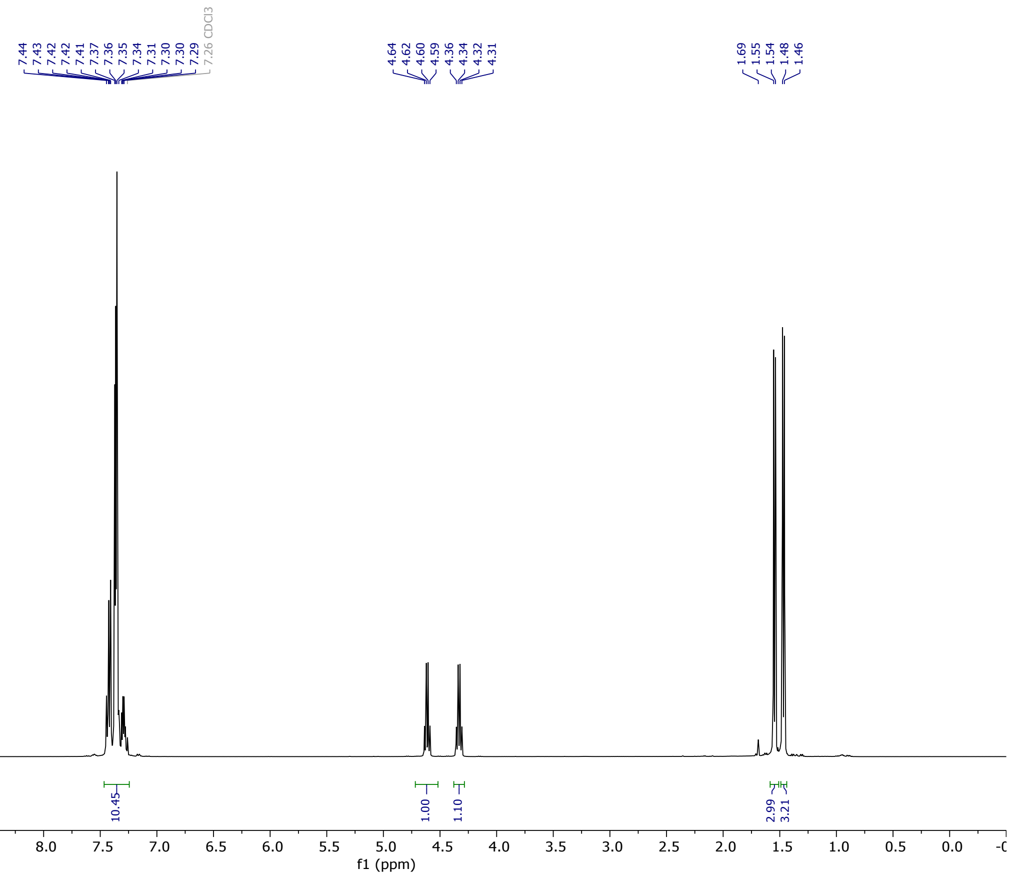

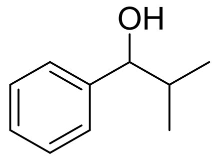

17

^{1}H, $CDCl_3$, 500 MHz

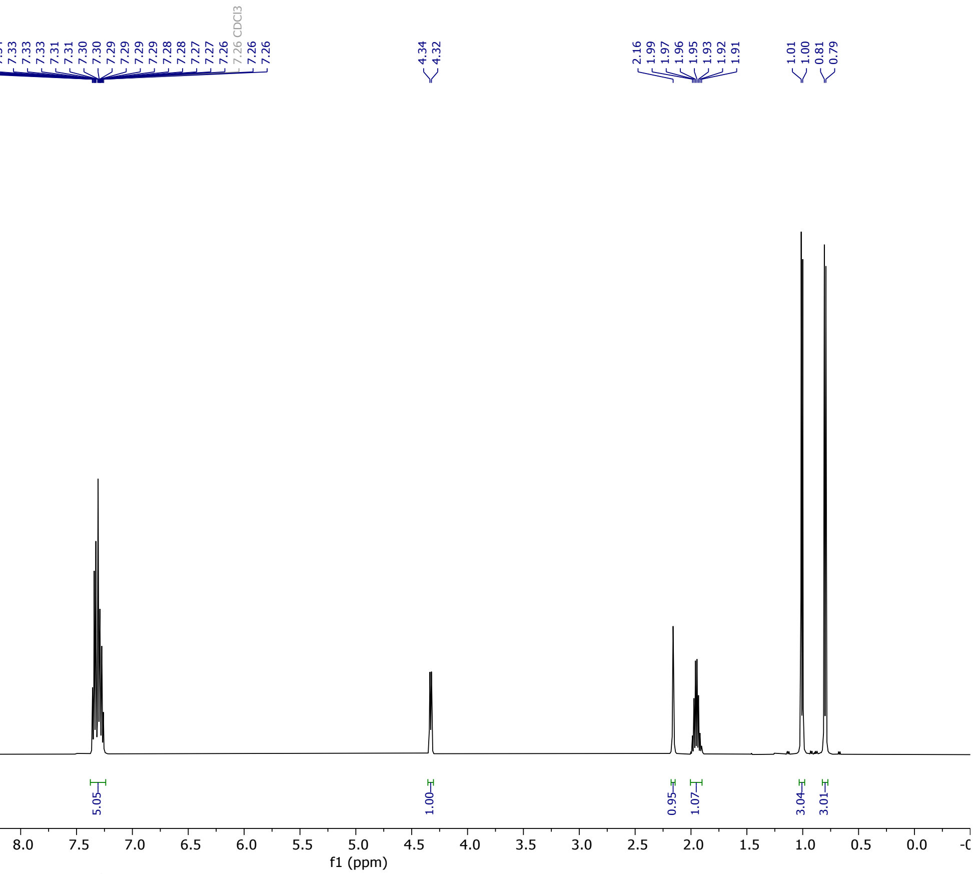

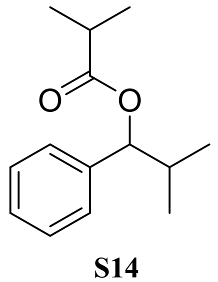

S14

^{1}H, $CDCl_3$, 500 MHz

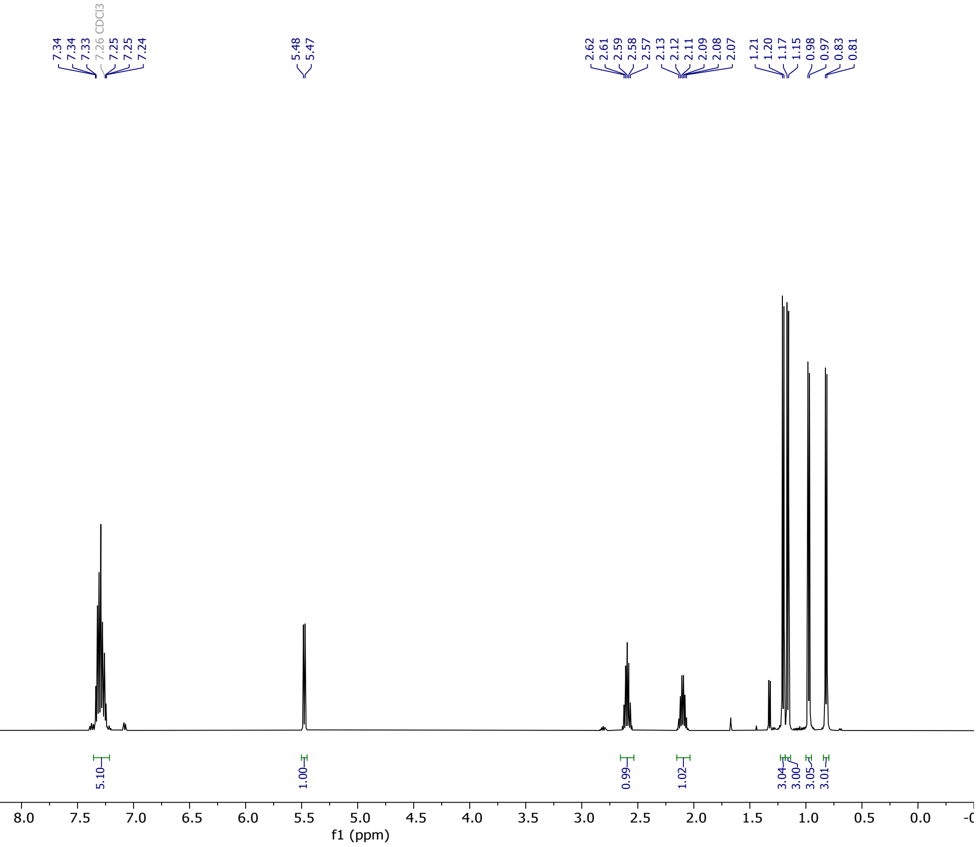

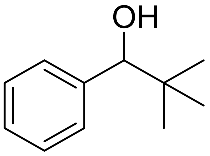

18

^{1}H, $CDCl_3$, 400 MHz

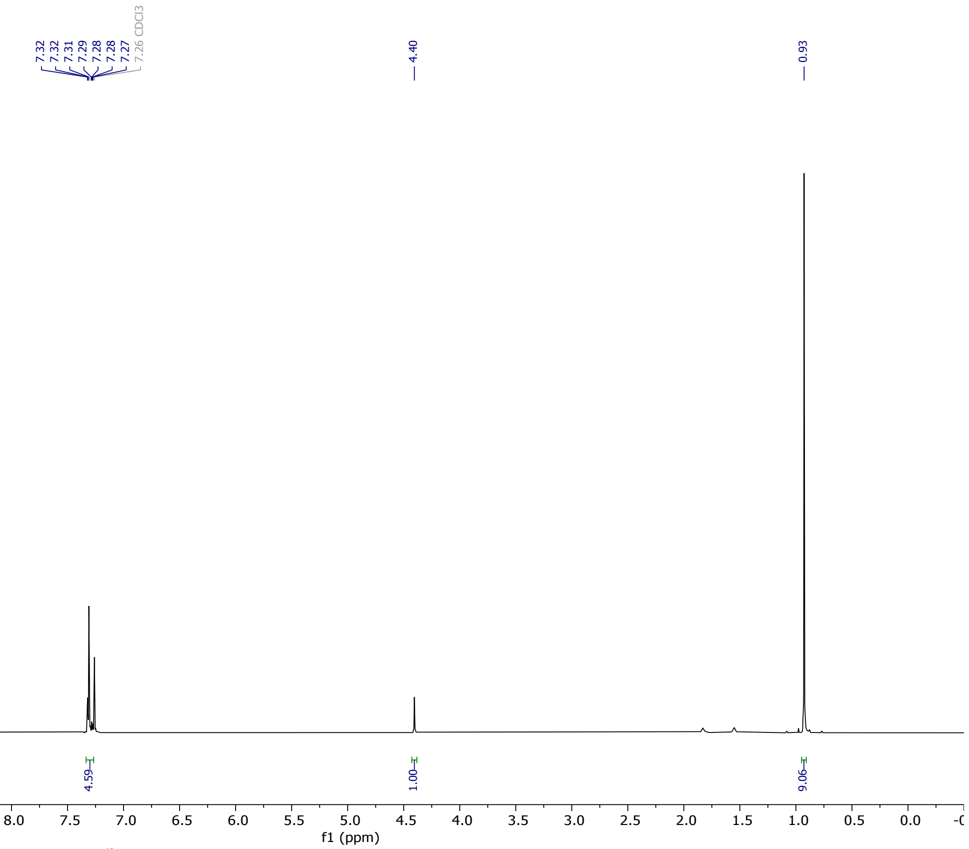

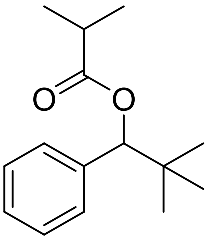

S15

^{1}H, $CDCl_3$, 500 MHz

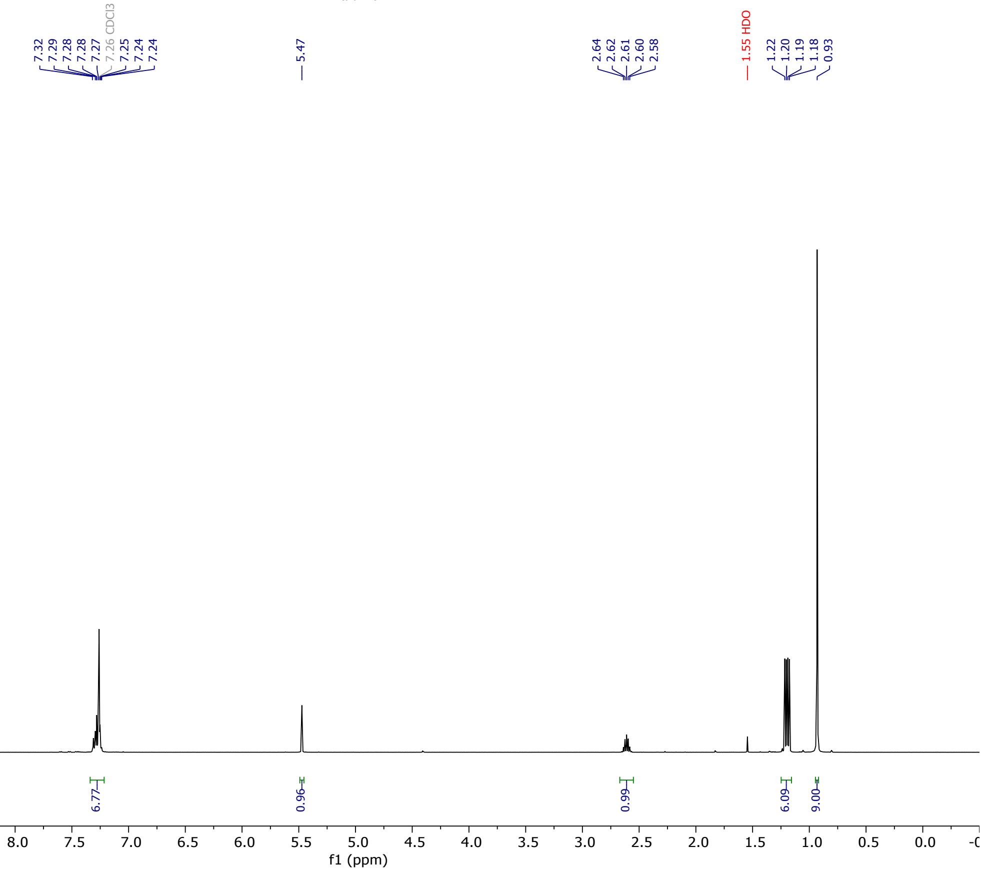

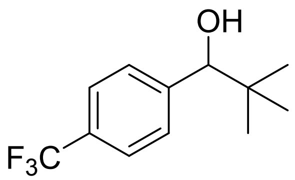

19

^{1}H, $CDCl_3$, 500 MHz

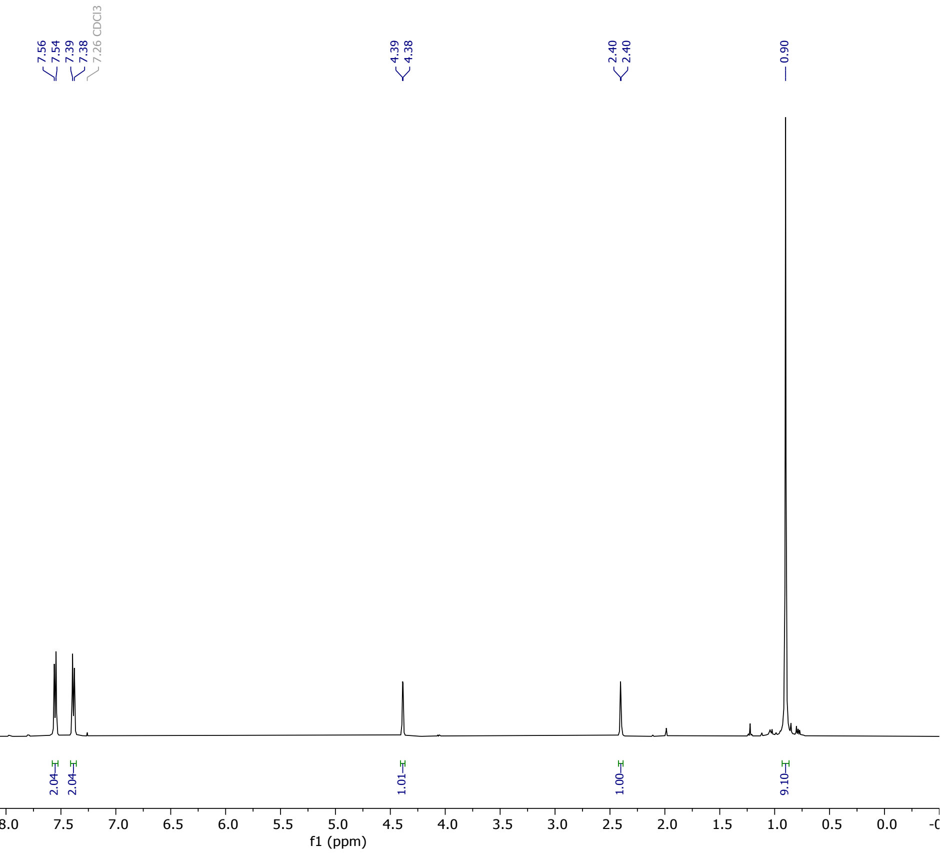

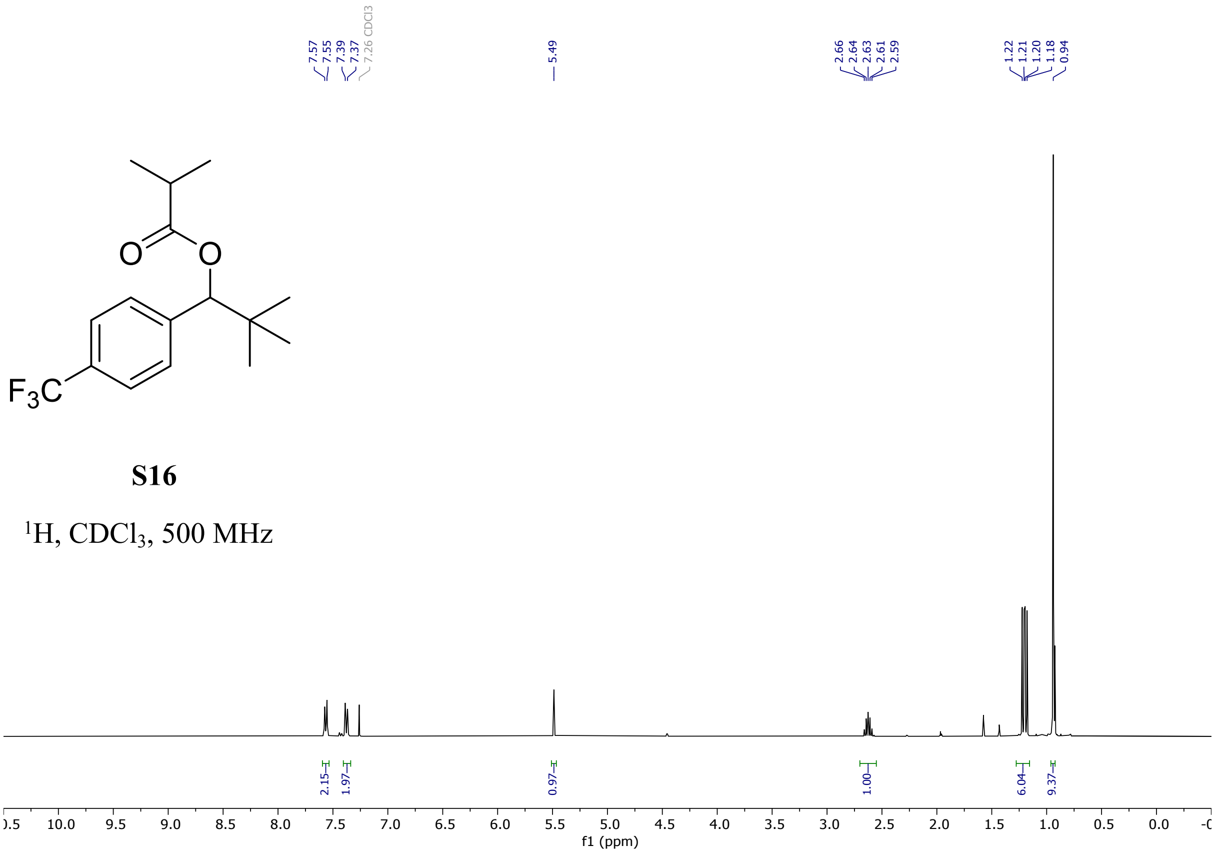

S16

^{1}H, $CDCl_3$, 500 MHz

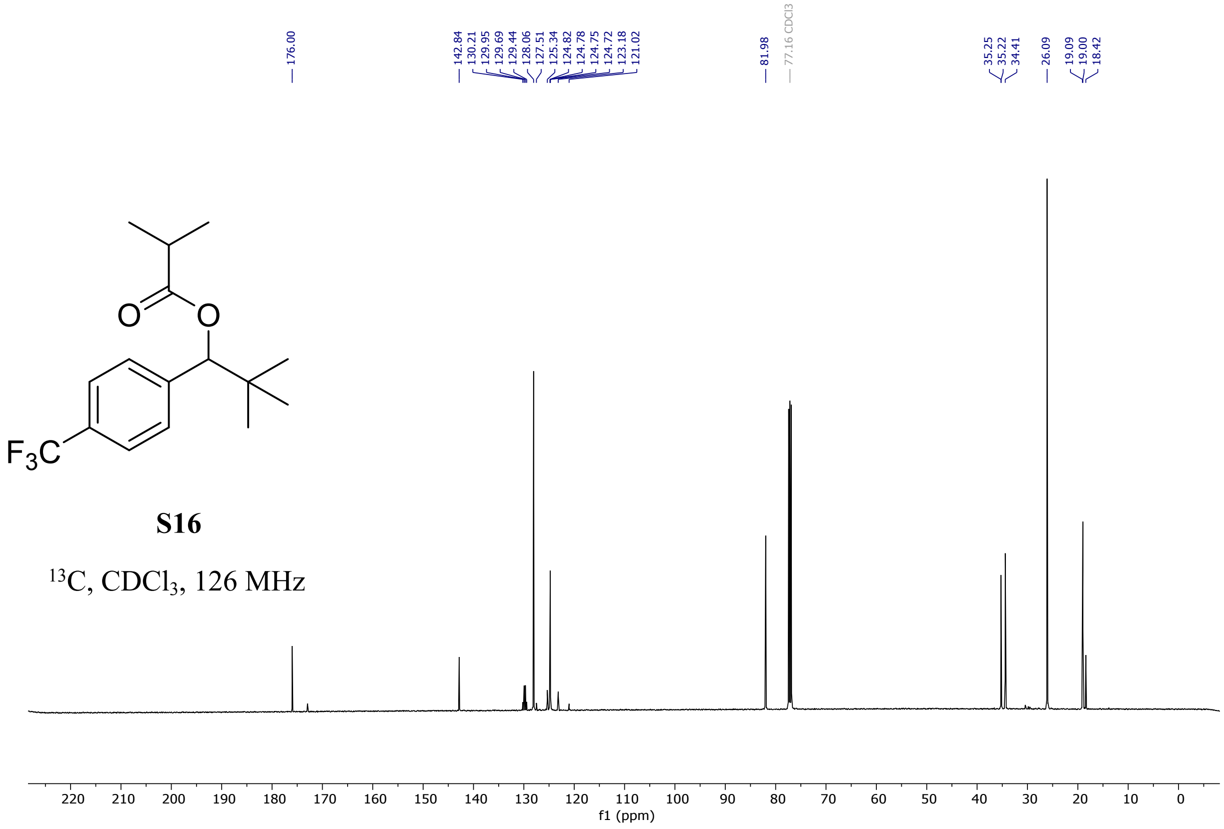

S16

^{13}C, $CDCl_3$, 126 MHz

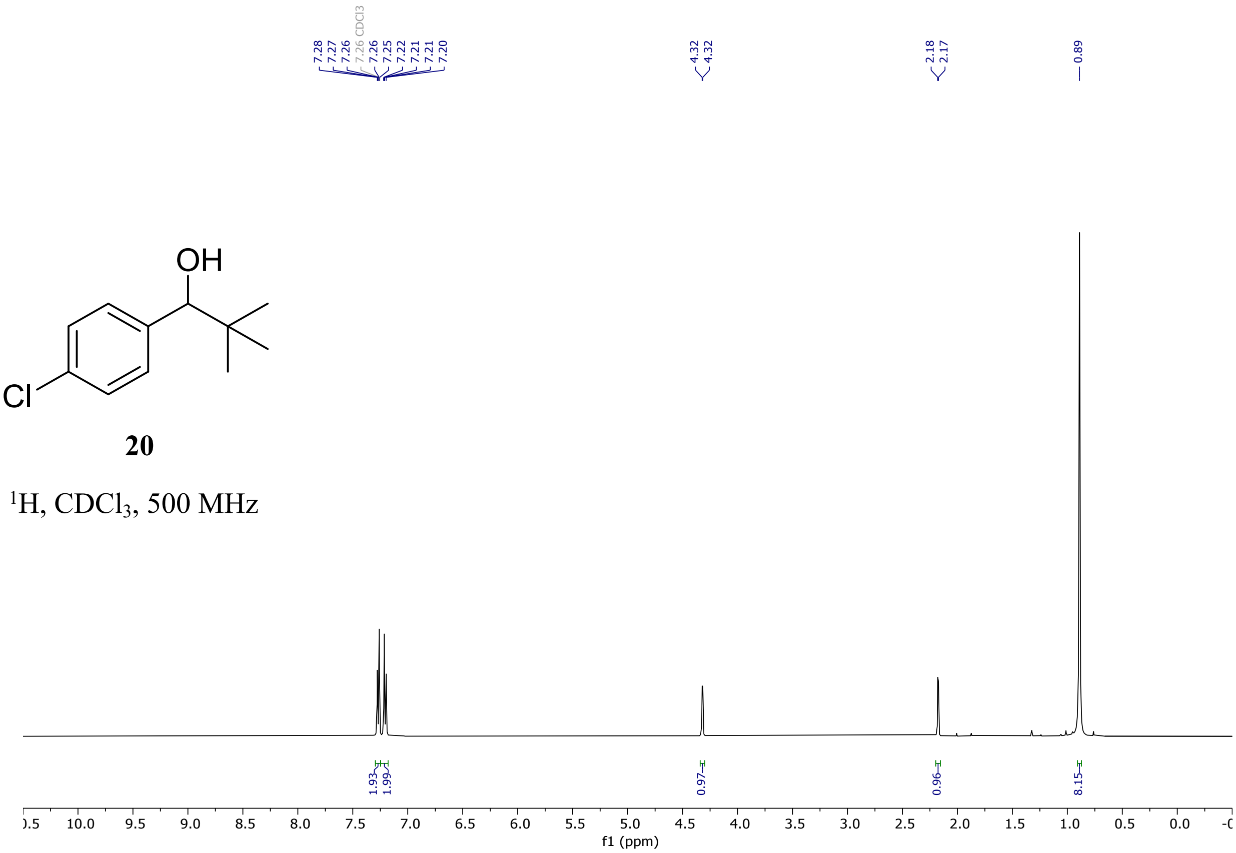

20

^{1}H, $CDCl_3$, 500 MHz

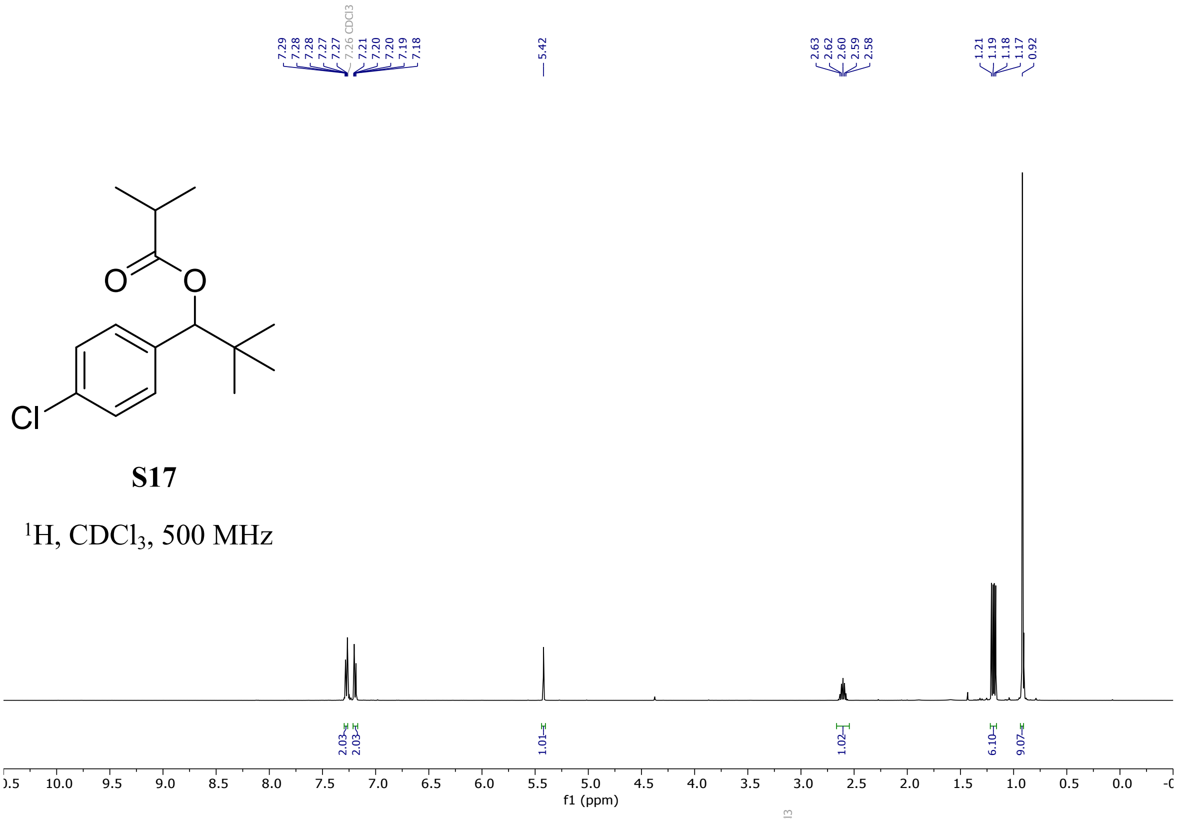

S17

^{1}H, $CDCl_3$, 500 MHz

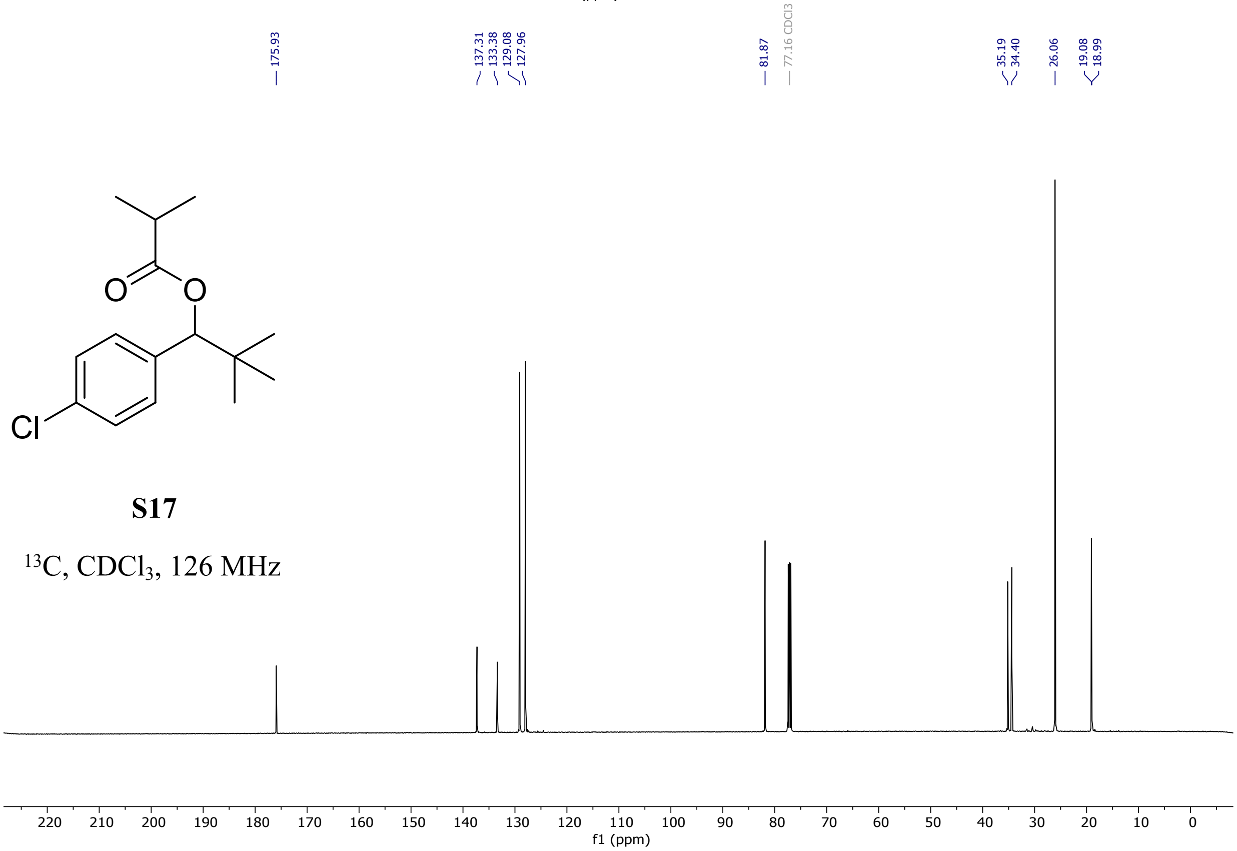

S17

^{13}C, $CDCl_3$, 126 MHz

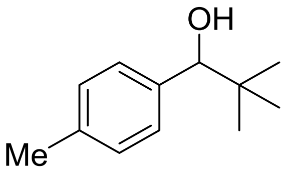

21

^{1}H, $CDCl_3$, 500 MHz

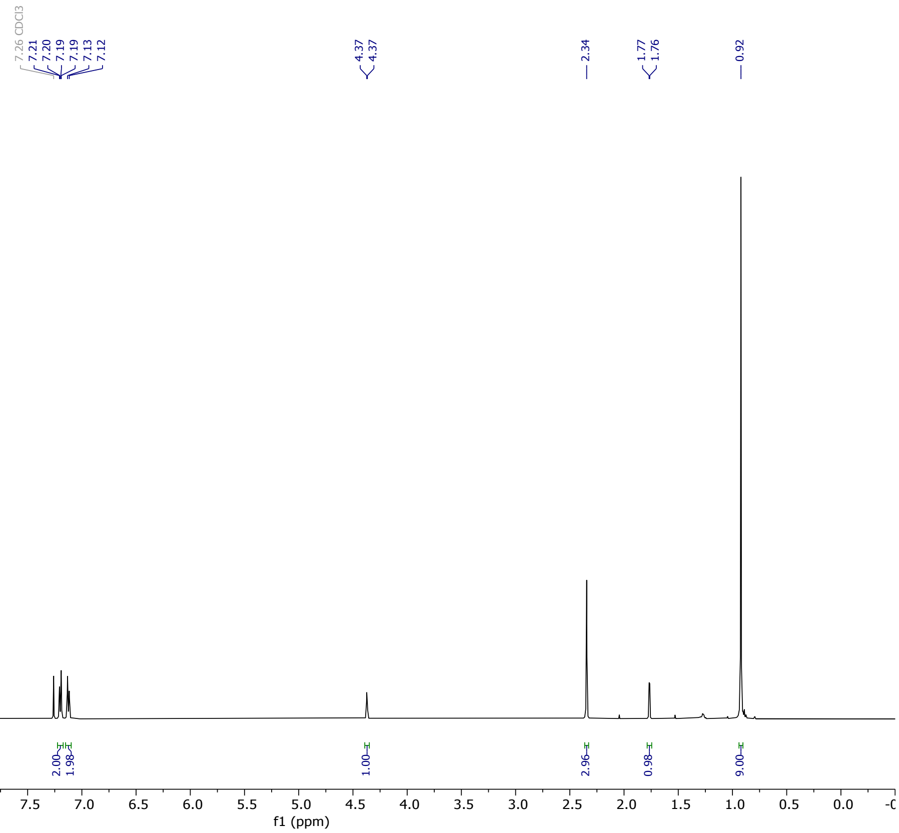

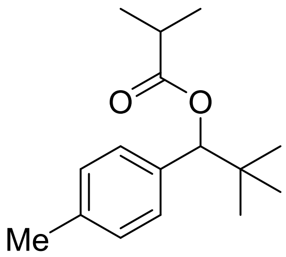

S18

^{1}H, $CDCl_3$, 500 MHz

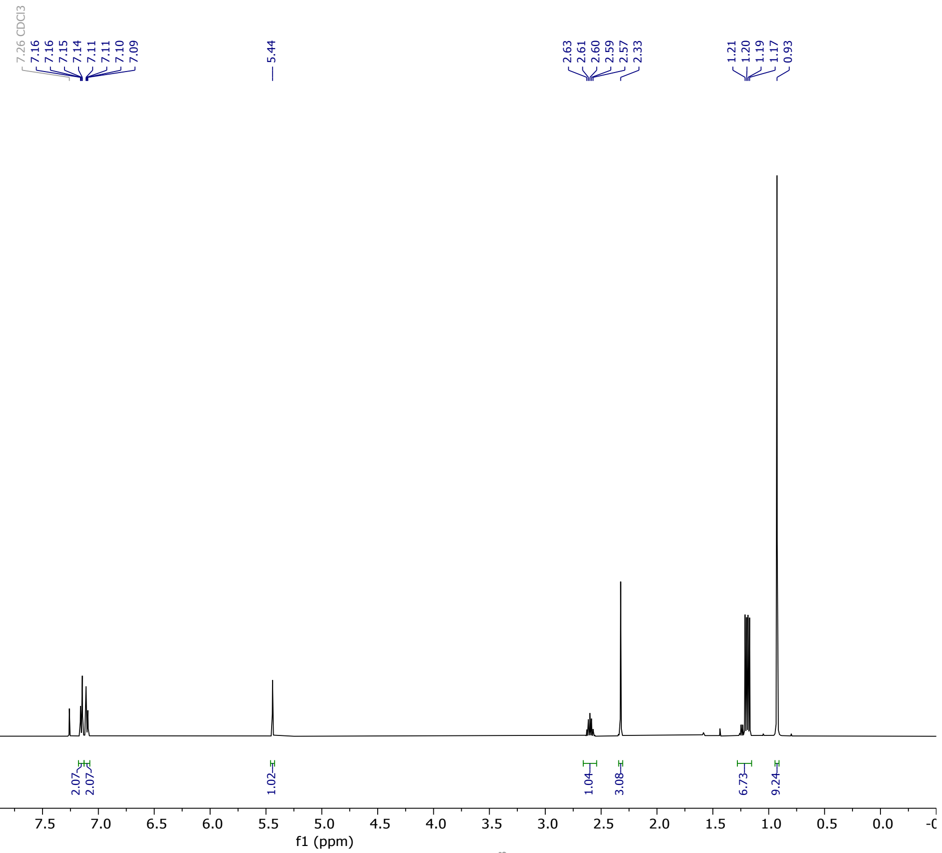

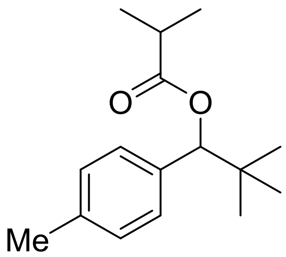

S18

^{13}C, $CDCl_3$, 126 MHz

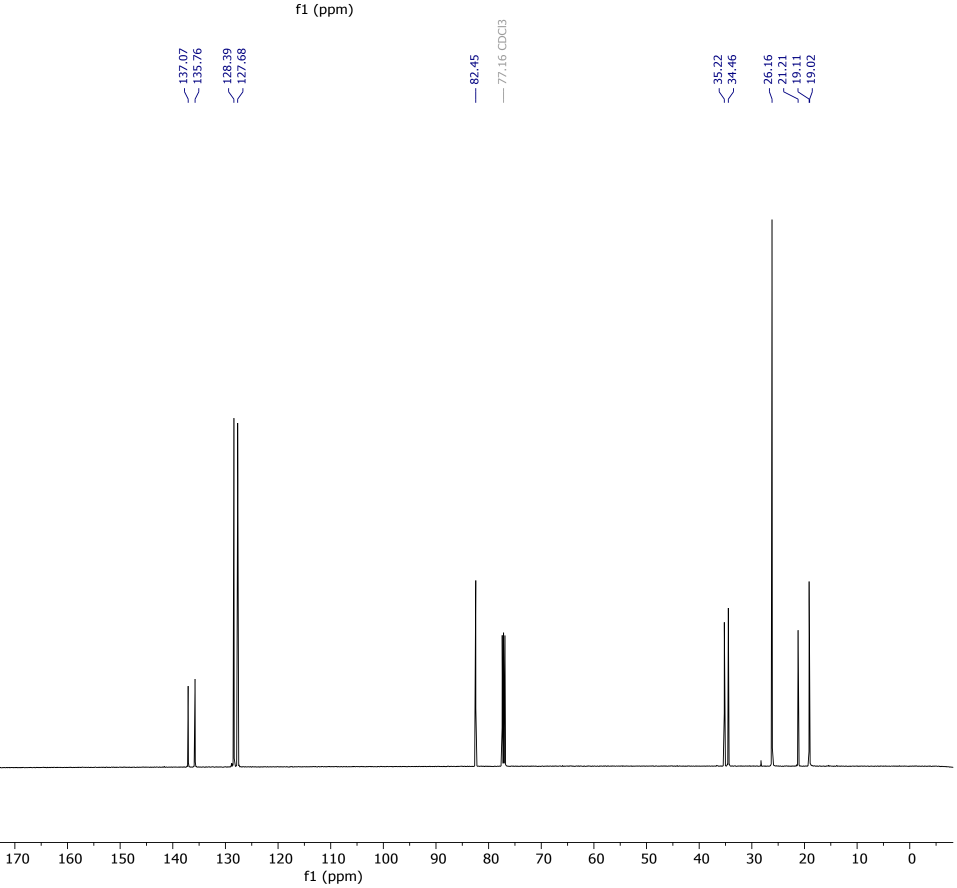

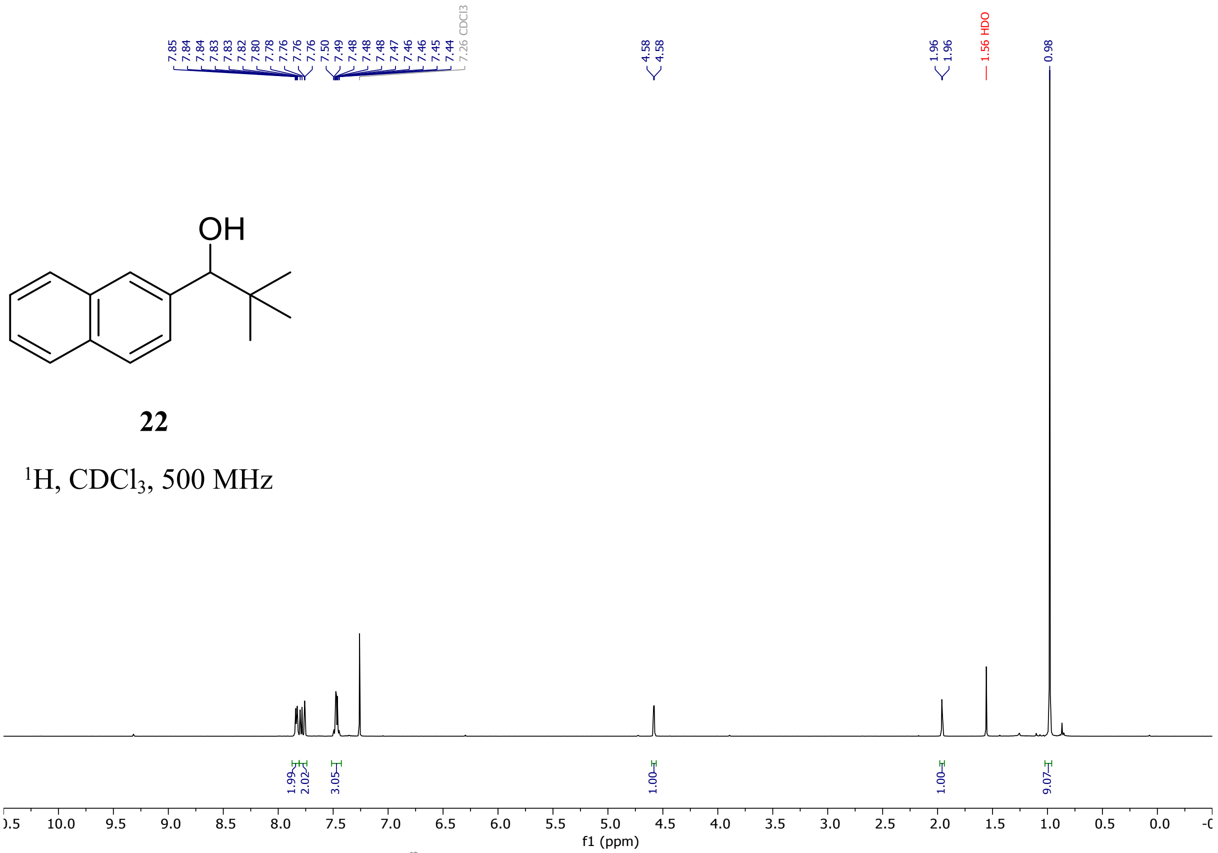

22

^{1}H, $CDCl_3$, 500 MHz

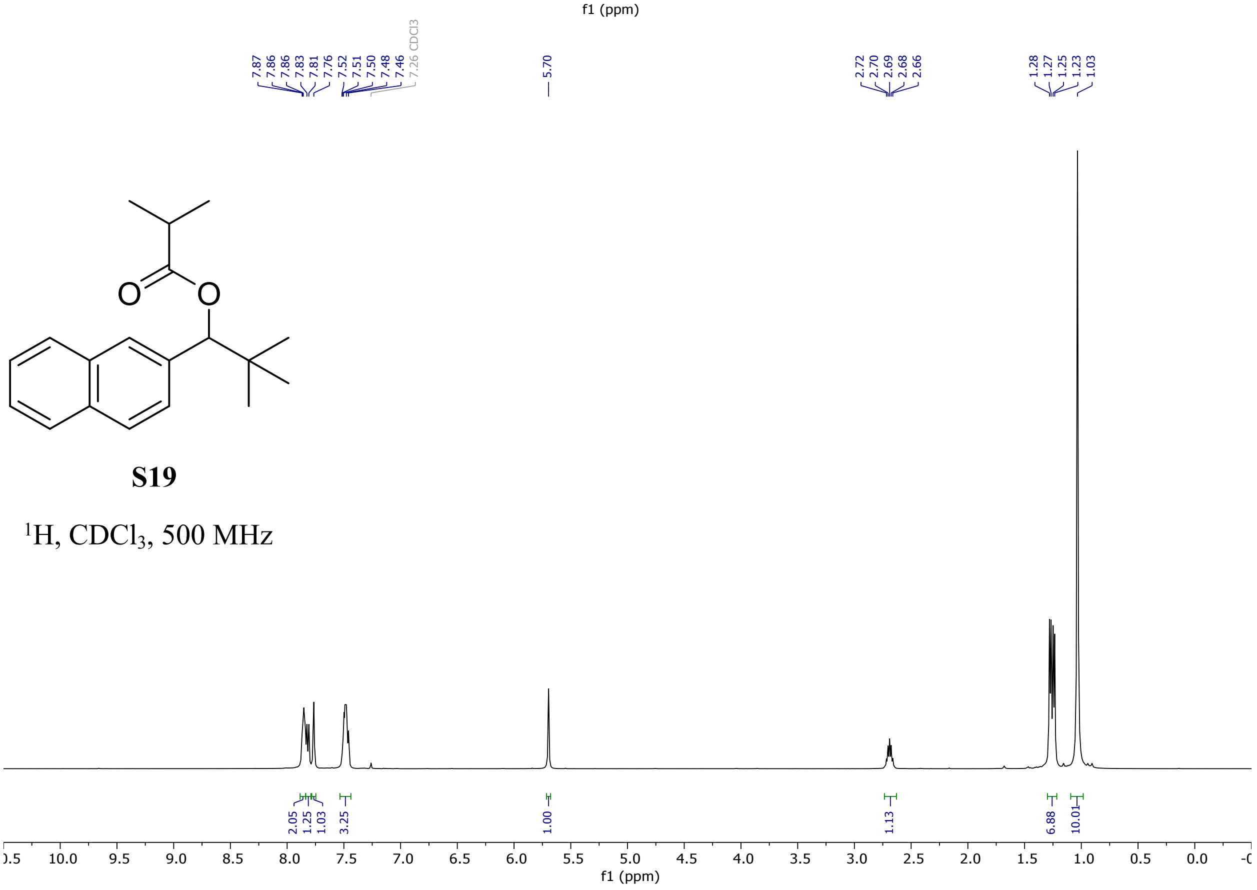

S19

^{1}H, $CDCl_3$, 500 MHz

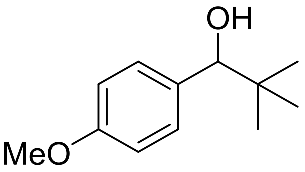

23

^{1}H, $CDCl_3$, 500 MHz

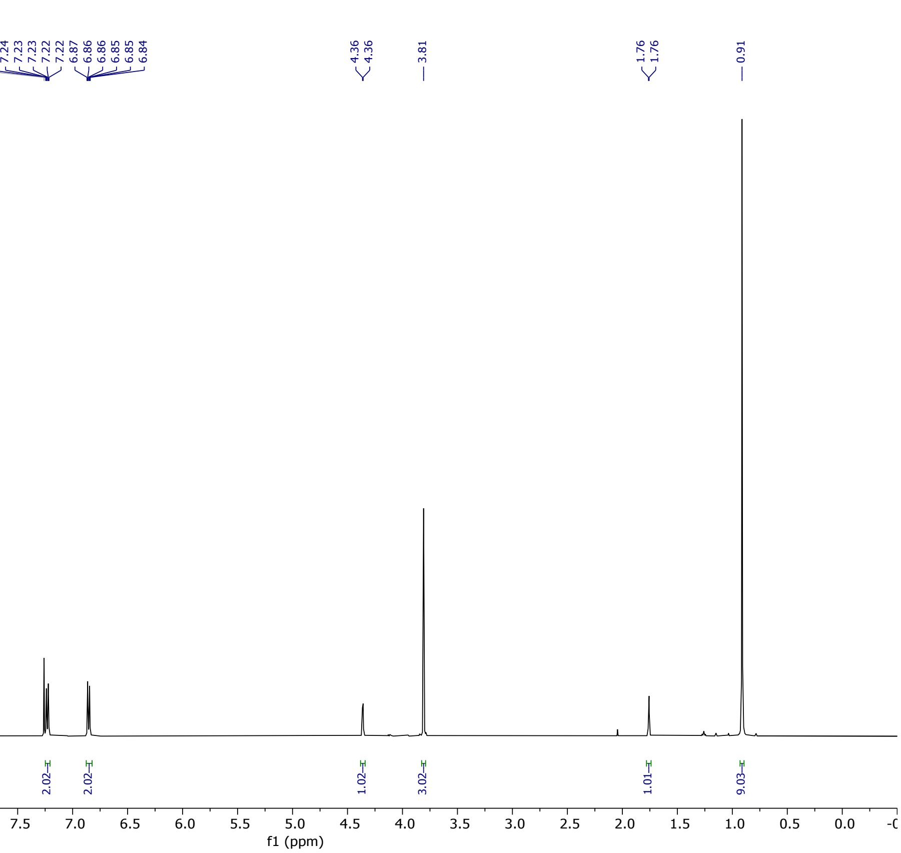

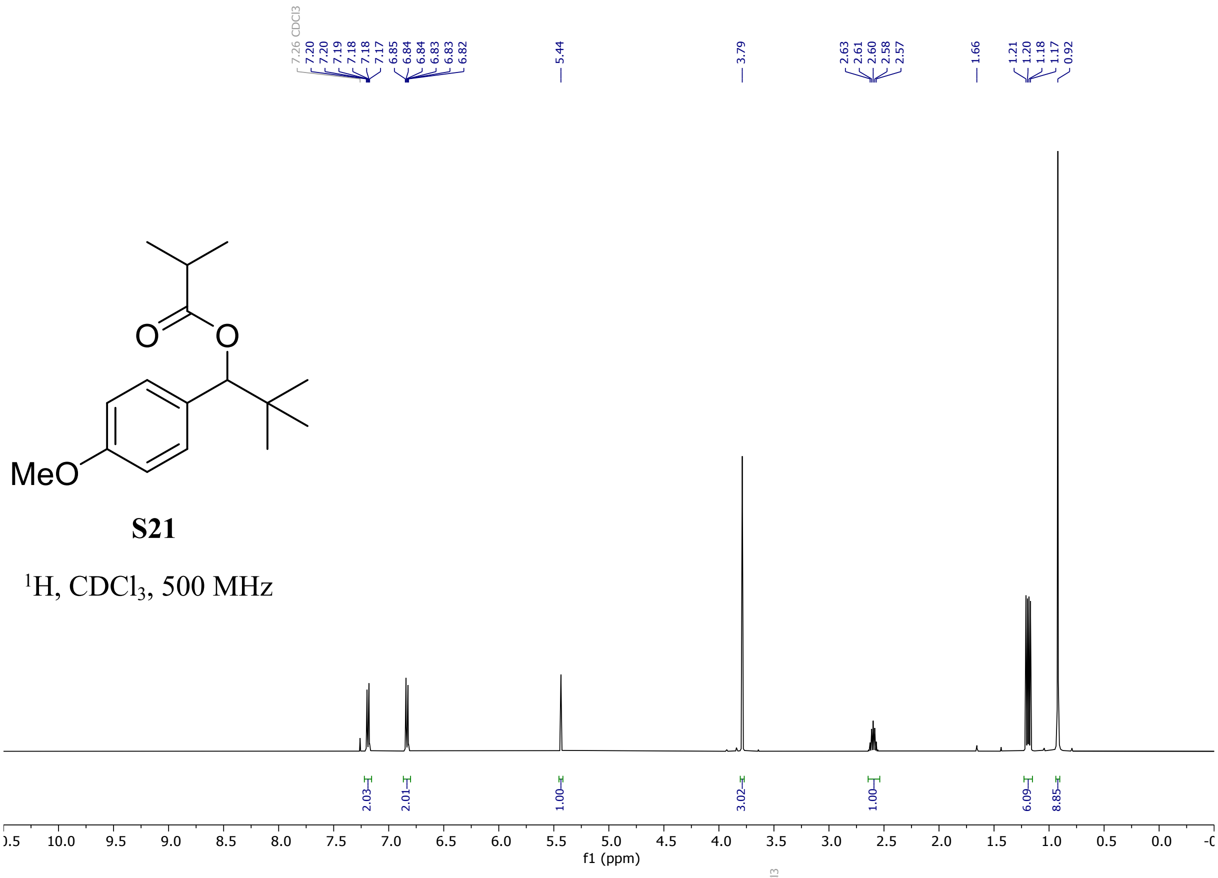

S21

^{1}H, $CDCl_3$, 500 MHz

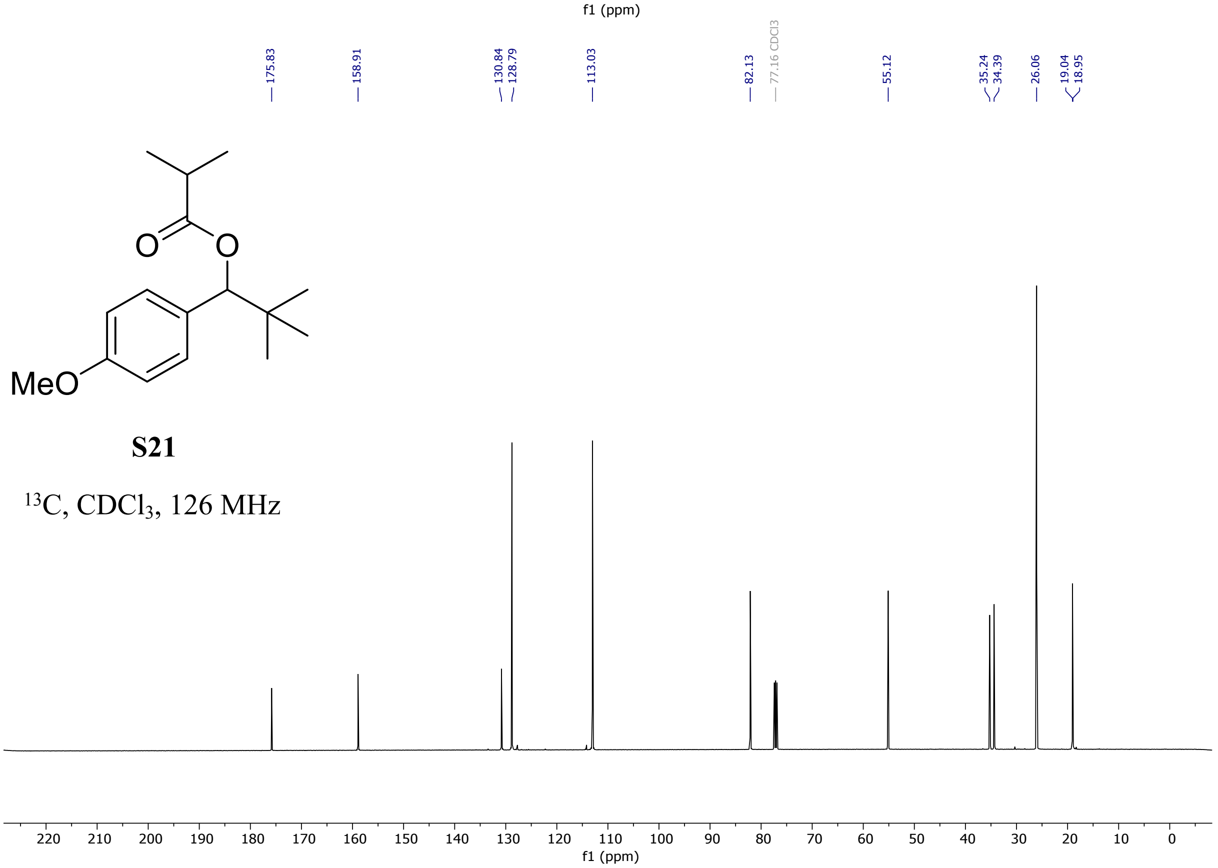

S21

^{13}C, $CDCl_3$, 126 MHz

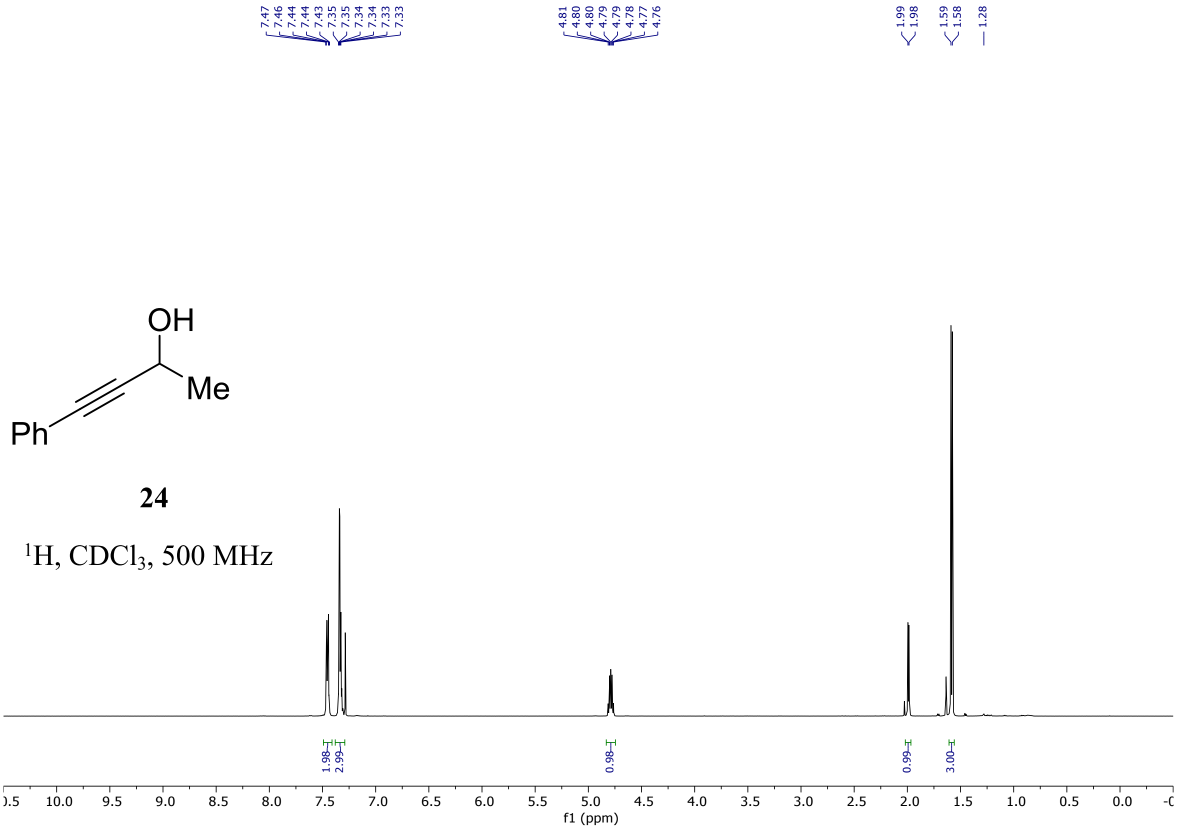
7.47 7.46 7.44 7.44 7.43 7.35 7.35 7.34 7.34 7.33 7.33
4.81 4.80 4.80 4.79 4.79 4.78 4.77 4.76
1.99 1.98 1.59 1.58 1.28
OH
Me
Ph
24
^{1}H, CDCl$_3$, 500 MHz
1.98 2.99 0.98 0.99 3.00
f1 (ppm)

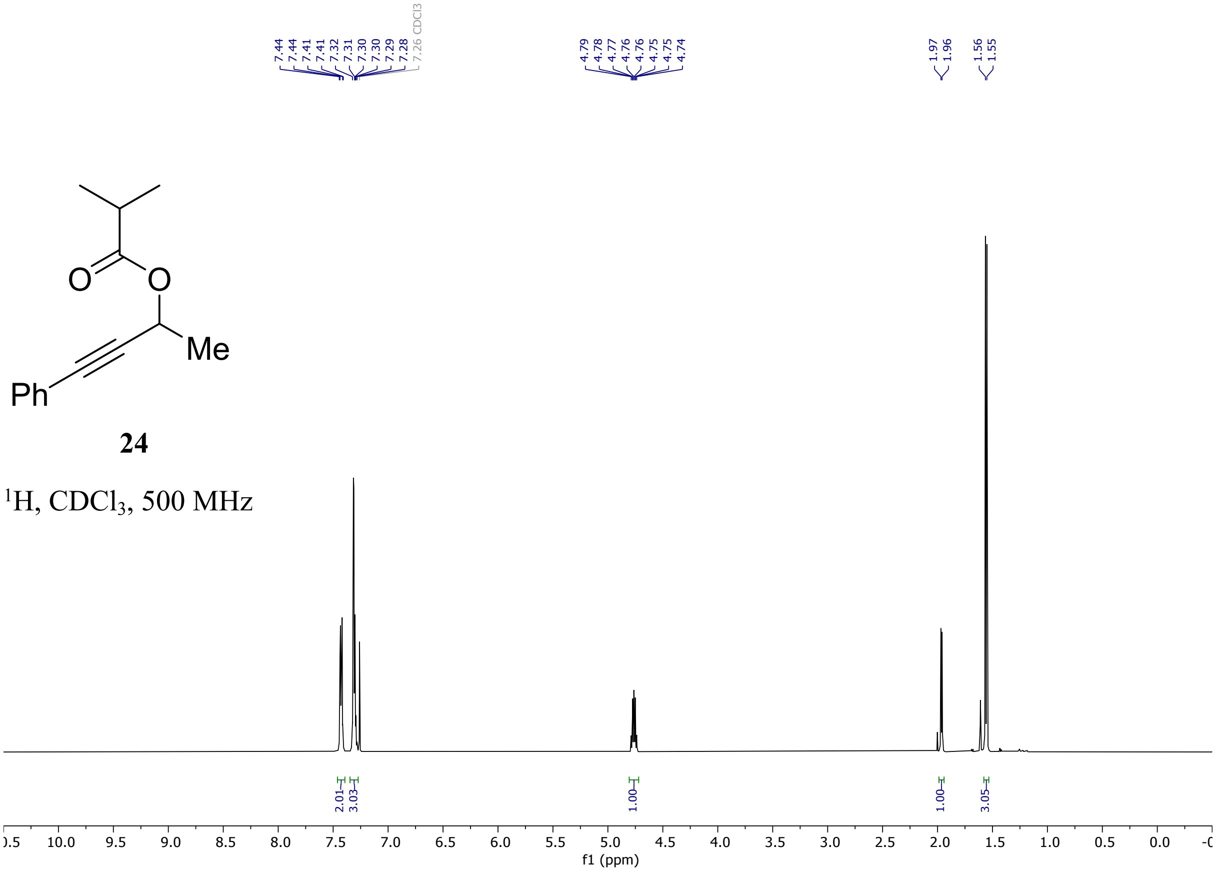
7.44 7.44 7.41 7.41 7.32 7.31 7.30 7.30 7.29 7.28 7.26 CDCl3
4.79 4.78 4.77 4.76 4.76 4.75 4.75 4.74
1.97 1.96 1.56 1.55
O
O
Me
Ph
24
^{1}H, CDCl$_3$, 500 MHz
2.01 3.03 1.00 1.00 3.05
f1 (ppm)

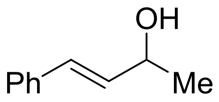

25

^{1}H, $CDCl_3$, 500 MHz

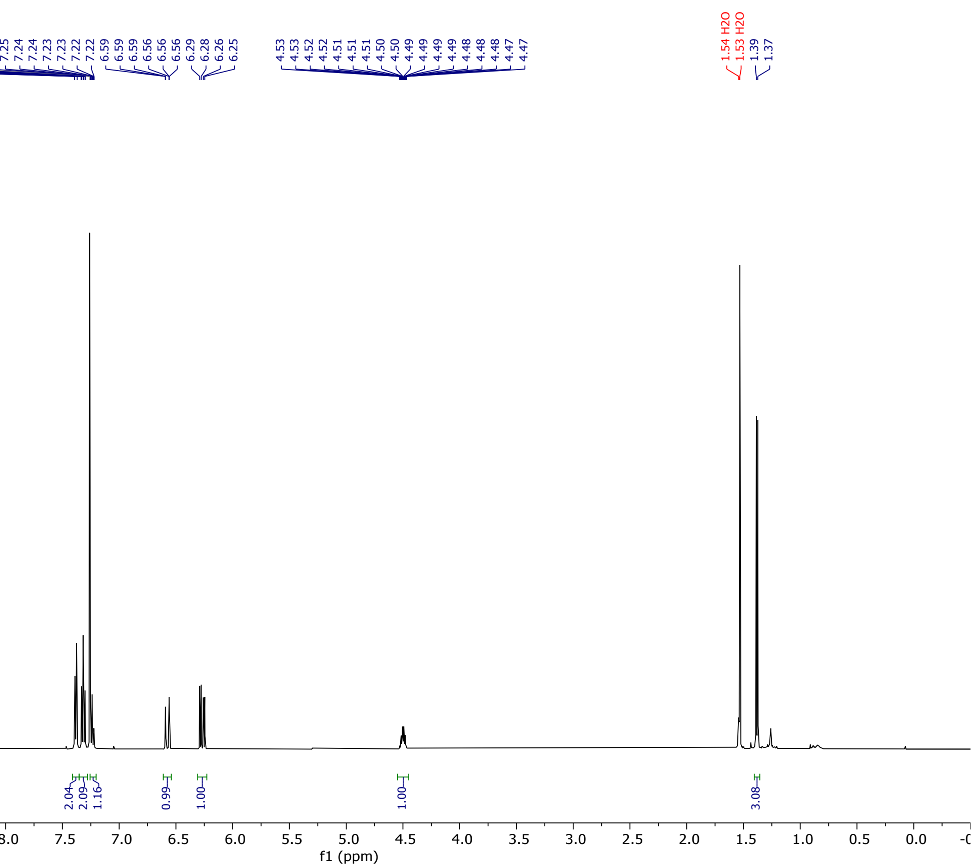

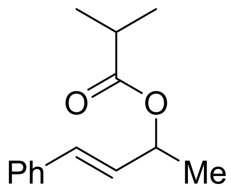

S27

^{1}H, $CDCl_3$, 500 MHz

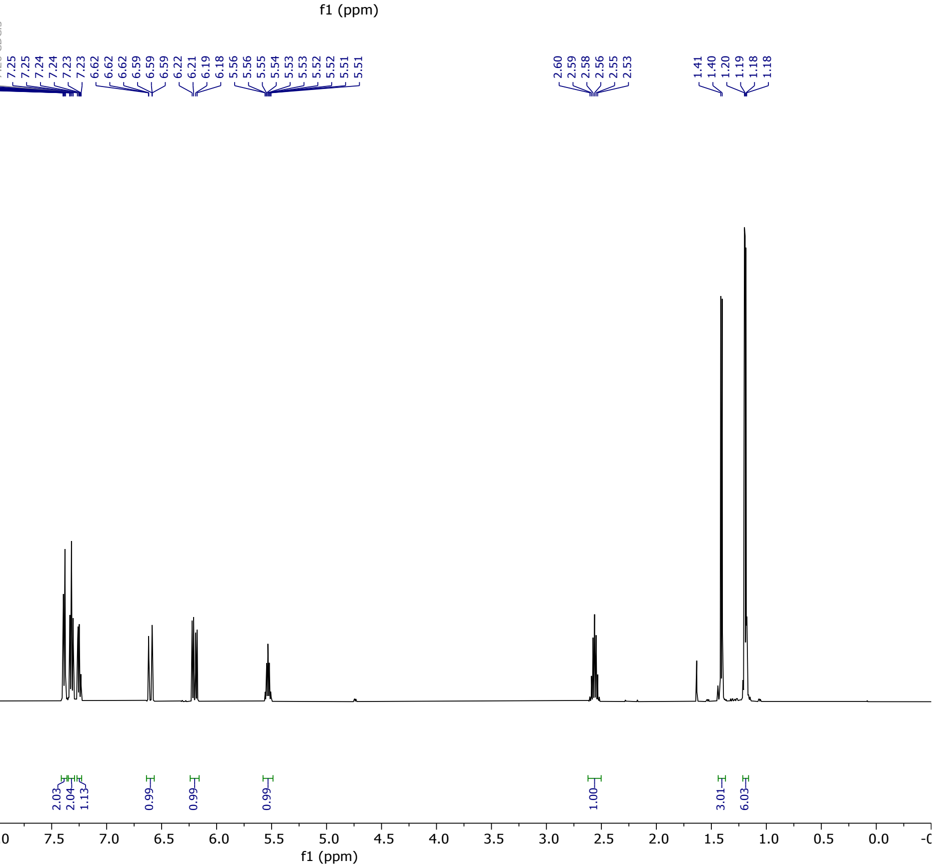

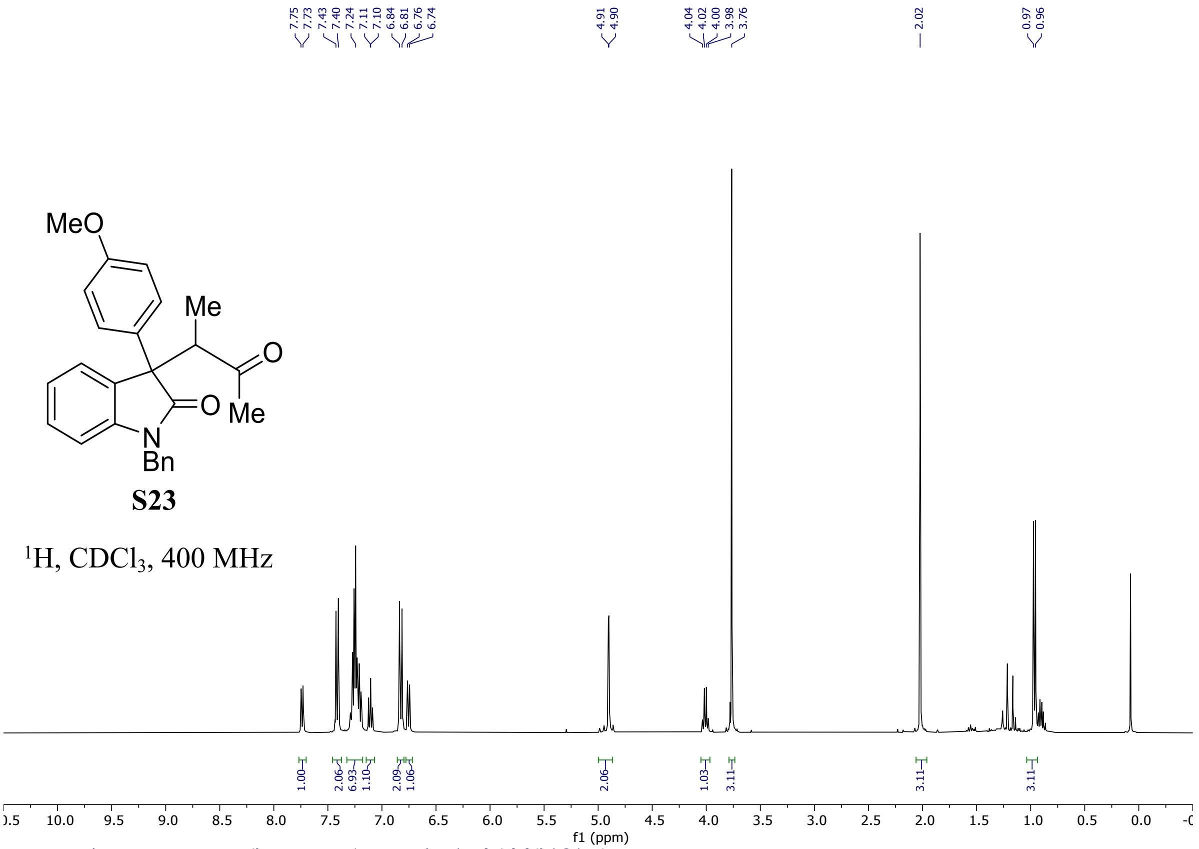
7.75
7.73
7.43
7.40
7.24
7.11
7.10
6.84
6.81
6.76
6.74
4.91
4.90
4.04
4.02
4.00
3.98
3.76
2.02
0.97
0.96
MeO
Me
O
O
Me
N
Bn
S23
^{1}H, $CDCl_3$, 400 MHz
1.00
2.06
6.93
1.10
2.09
1.06
2.06
1.03
3.11
3.11
3.11
f1 (ppm)

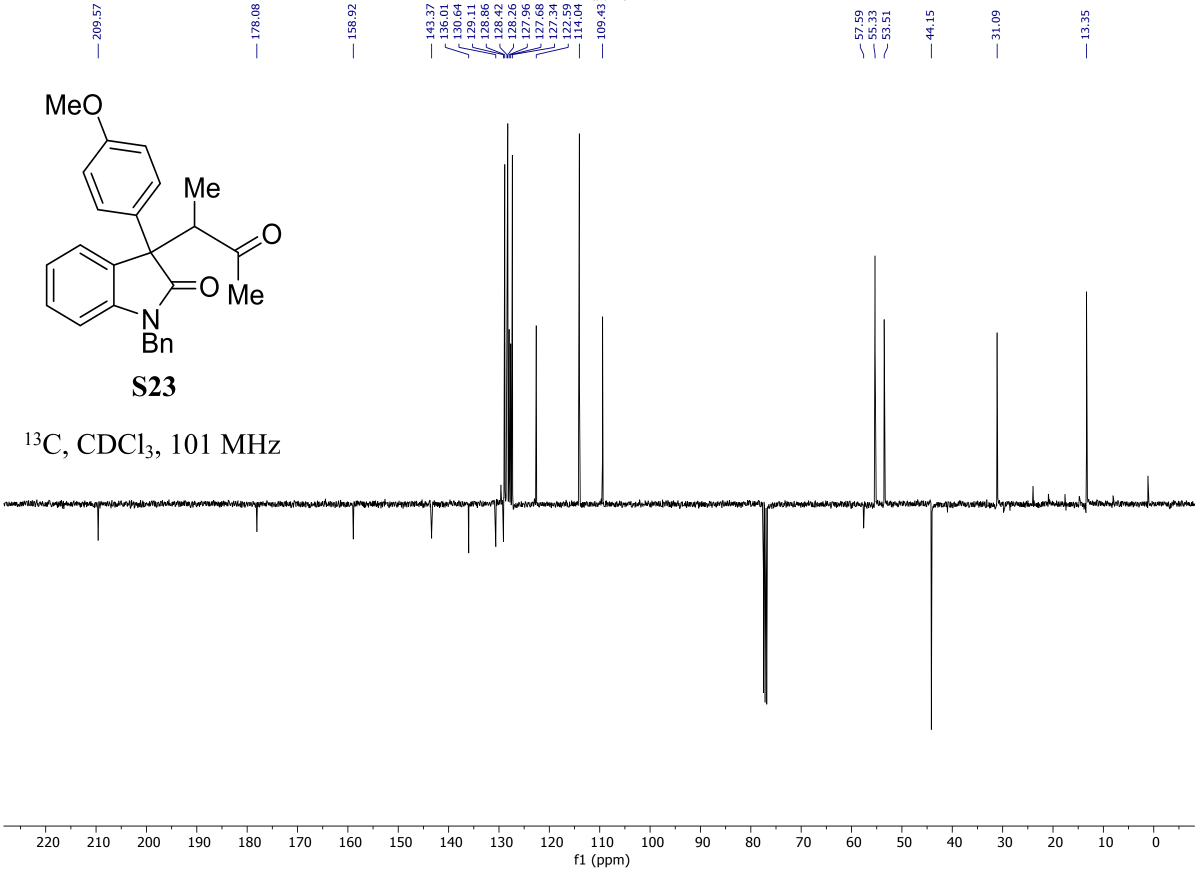
209.57
178.08
158.92
143.37
136.01
130.64
129.11
128.86
128.42
128.26
127.96
127.68
127.34
122.59
114.04
109.43
57.59
55.33
53.51
44.15
31.09
13.35
MeO
Me
O
O
Me
N
Bn
S23
^{13}C, $CDCl_3$, 101 MHz
f1 (ppm)

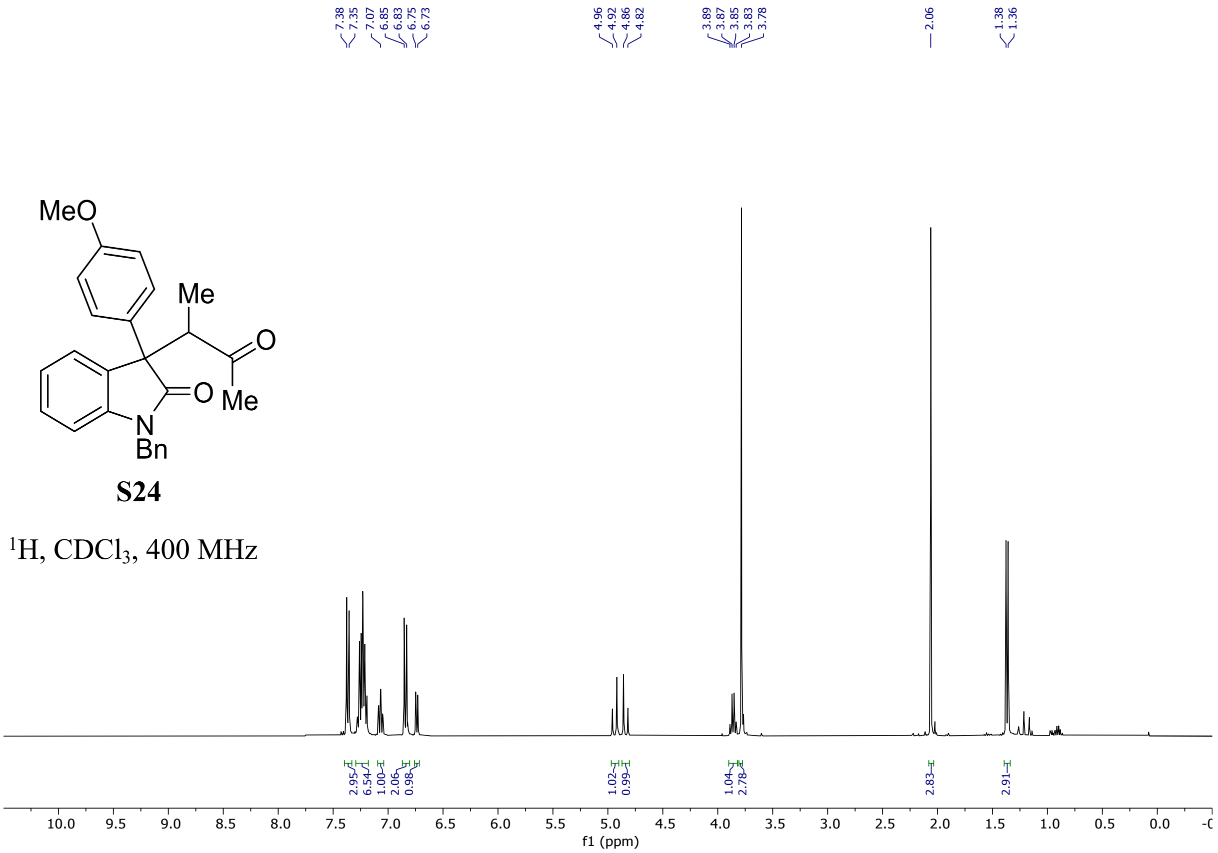
7.38
7.35
7.07
6.85
6.83
6.75
6.73
4.96
4.92
4.86
4.82
3.89
3.87
3.85
3.83
3.78
2.06
1.38
1.36
MeO
Me
O
O
Me
N
Bn
S24
^{1}H, CDCl$_3$, 400 MHz
2.95
6.54
1.00
2.06
0.98
1.02
0.99
1.04
2.78
2.83
2.91
f1 (ppm)

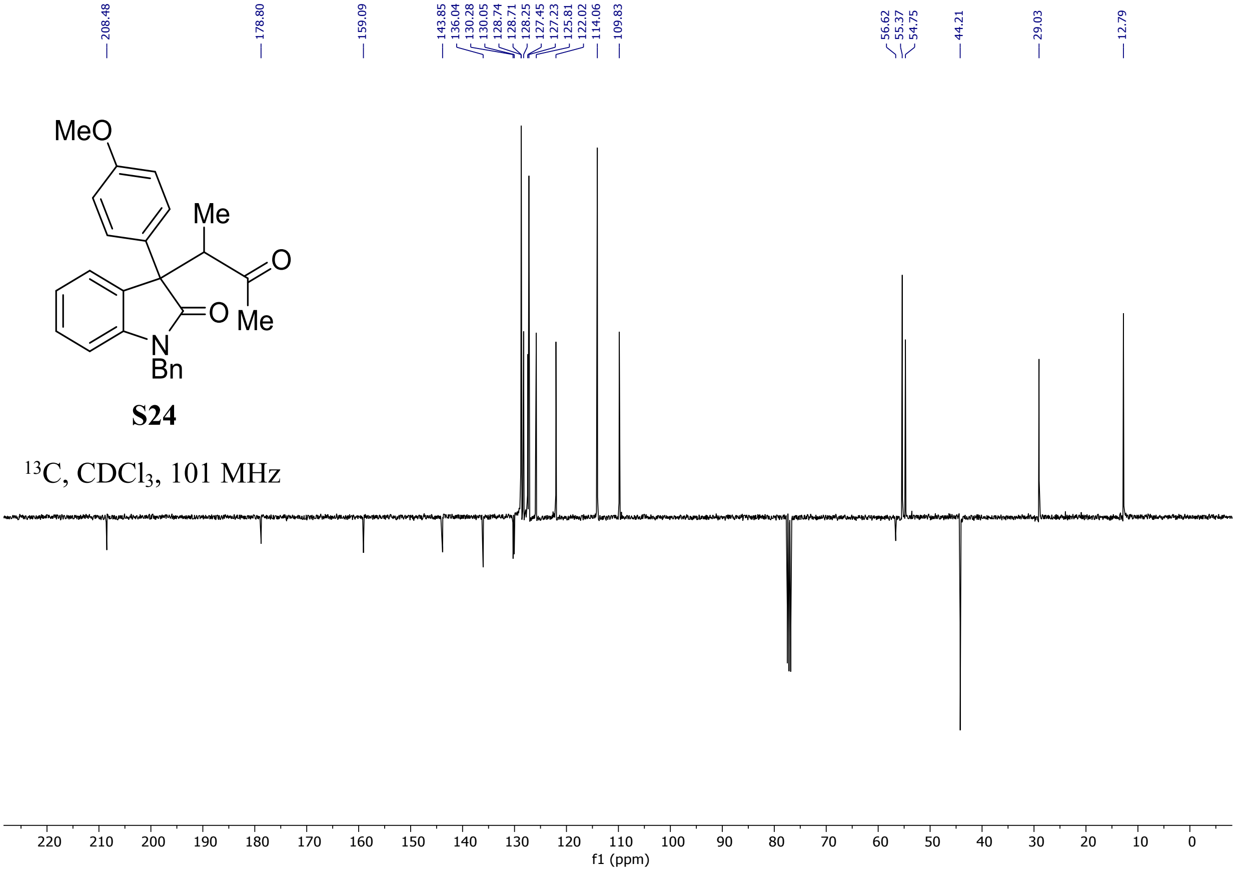
208.48
178.80
159.09
143.85
136.04
130.28
130.05
128.74
128.71
128.25
127.45
127.23
125.81
122.02
114.06
109.83
56.62
55.37
54.75
44.21
29.03
12.79
MeO
Me
O
O
Me
N
Bn
S24
^{13}C, CDCl$_3$, 101 MHz
f1 (ppm)

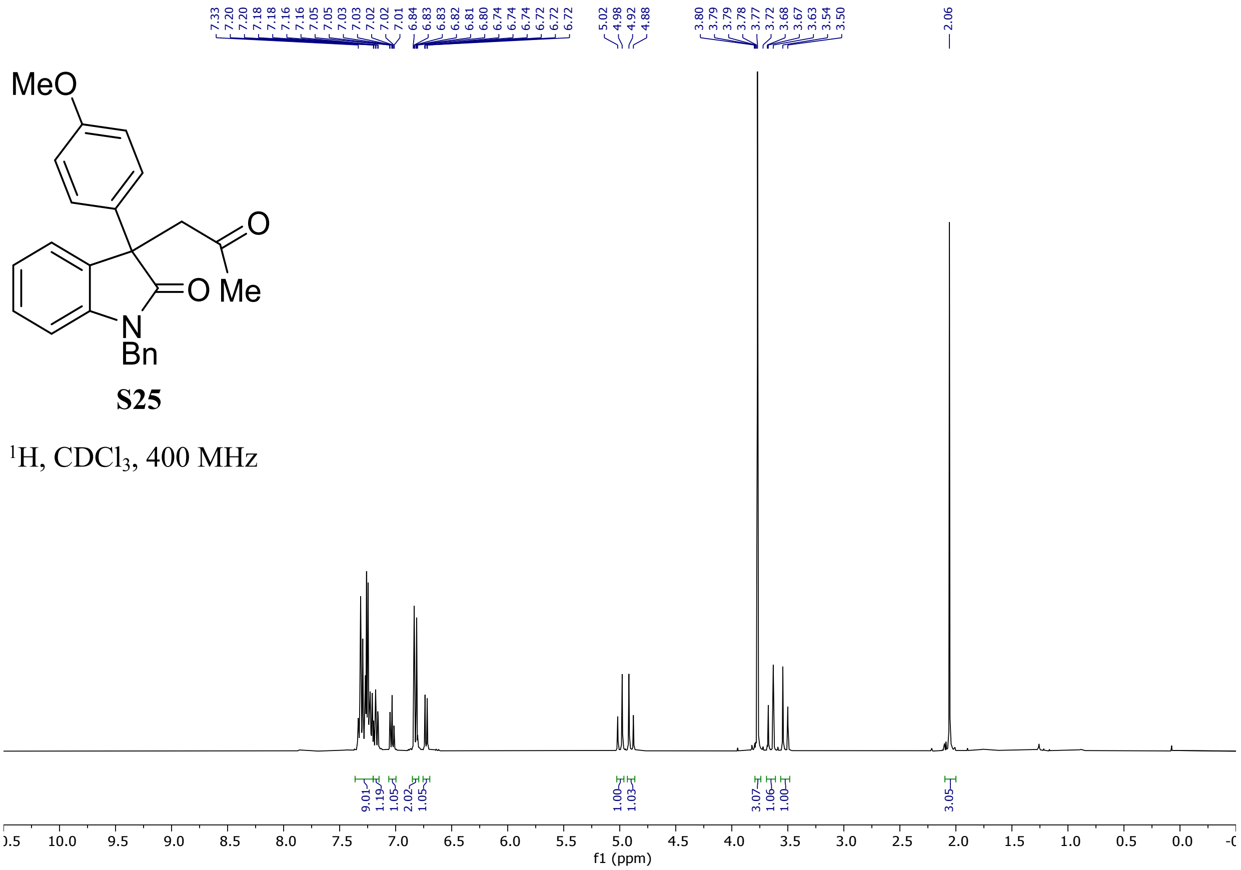
7.33 7.20 7.20 7.18 7.18 7.16 7.16 7.05 7.05 7.03 7.03 7.02 7.02 7.01 6.84 6.83 6.83 6.82 6.81 6.80 6.74 6.74 6.74 6.72 6.72 6.72
5.02 4.98 4.92 4.88
3.80 3.79 3.79 3.78 3.77 3.72 3.68 3.67 3.63 3.54 3.50
2.06
MeO
O
O Me
N
Bn
S25
1H, CDCl3, 400 MHz
9.01 1.19 1.05 2.02 1.05
1.00 1.03
3.07 1.06 1.00
3.05
10.0 9.5 9.0 8.5 8.0 7.5 7.0 6.5 6.0 5.5 5.0 4.5 4.0 3.5 3.0 2.5 2.0 1.5 1.0 0.5 0.0
f1 (ppm)

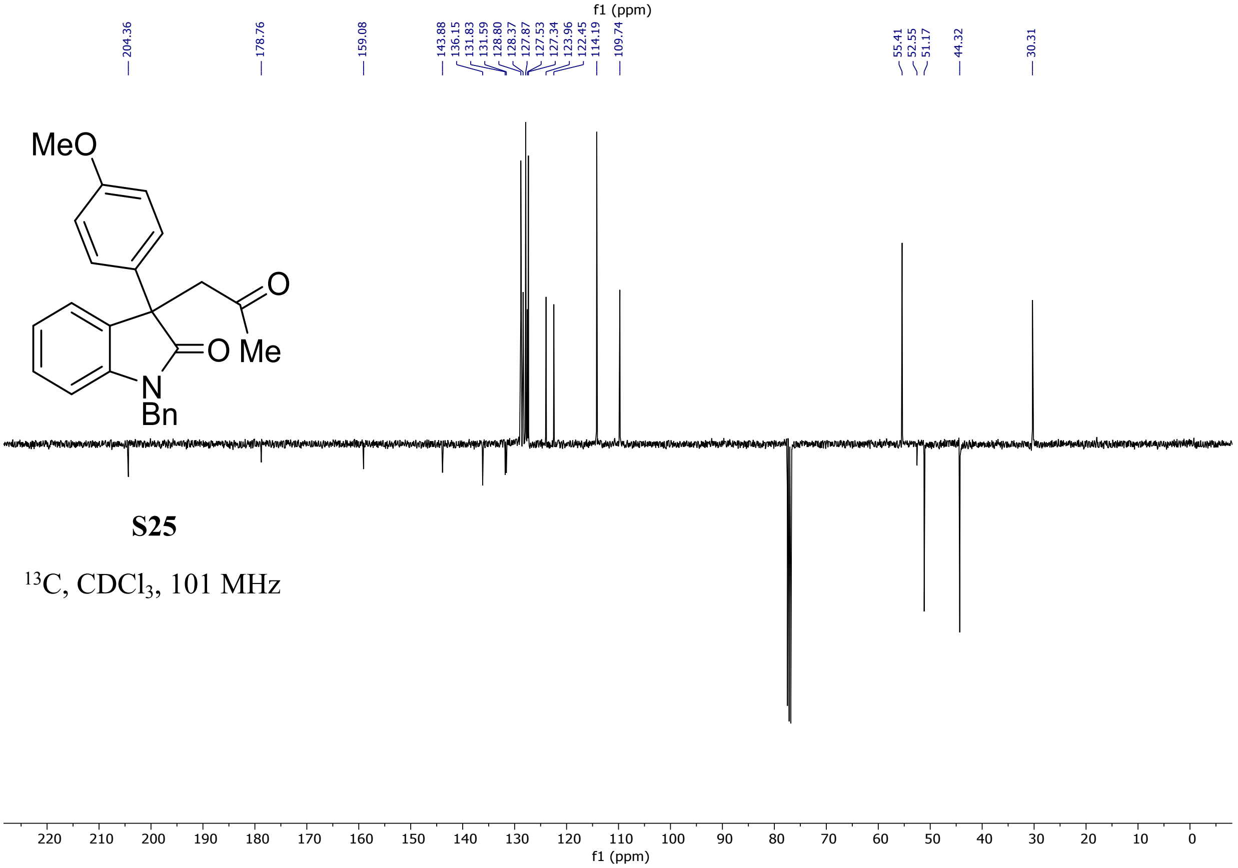
204.36
178.76
159.08
143.88 136.15 131.83 131.59 128.80 128.37 127.87 127.53 127.34 123.96 122.45 114.19 109.74
55.41 52.55 51.17 44.32 30.31
MeO
O
O Me
N
Bn
S25
13C, CDCl3, 101 MHz
220 210 200 190 180 170 160 150 140 130 120 110 100 90 80 70 60 50 40 30 20 10 0
f1 (ppm)

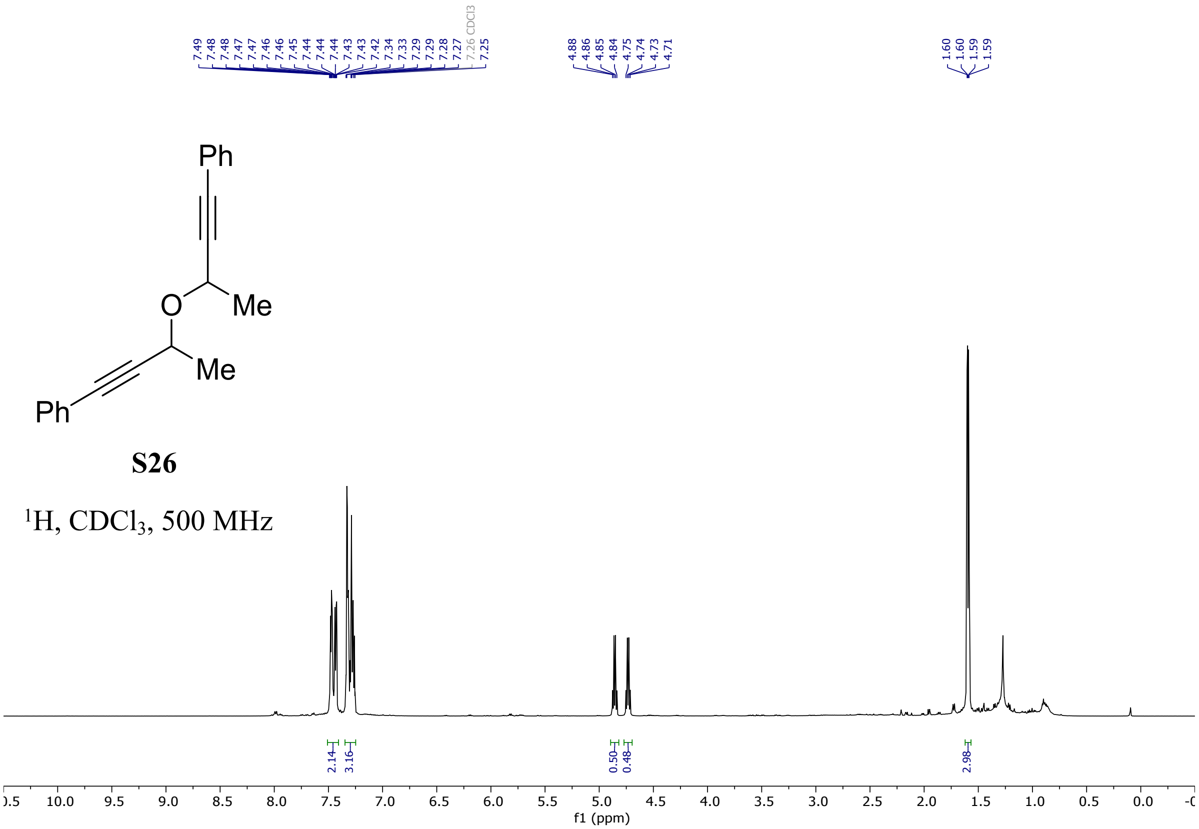

S26

^{1}H, $CDCl_3$, 500 MHz

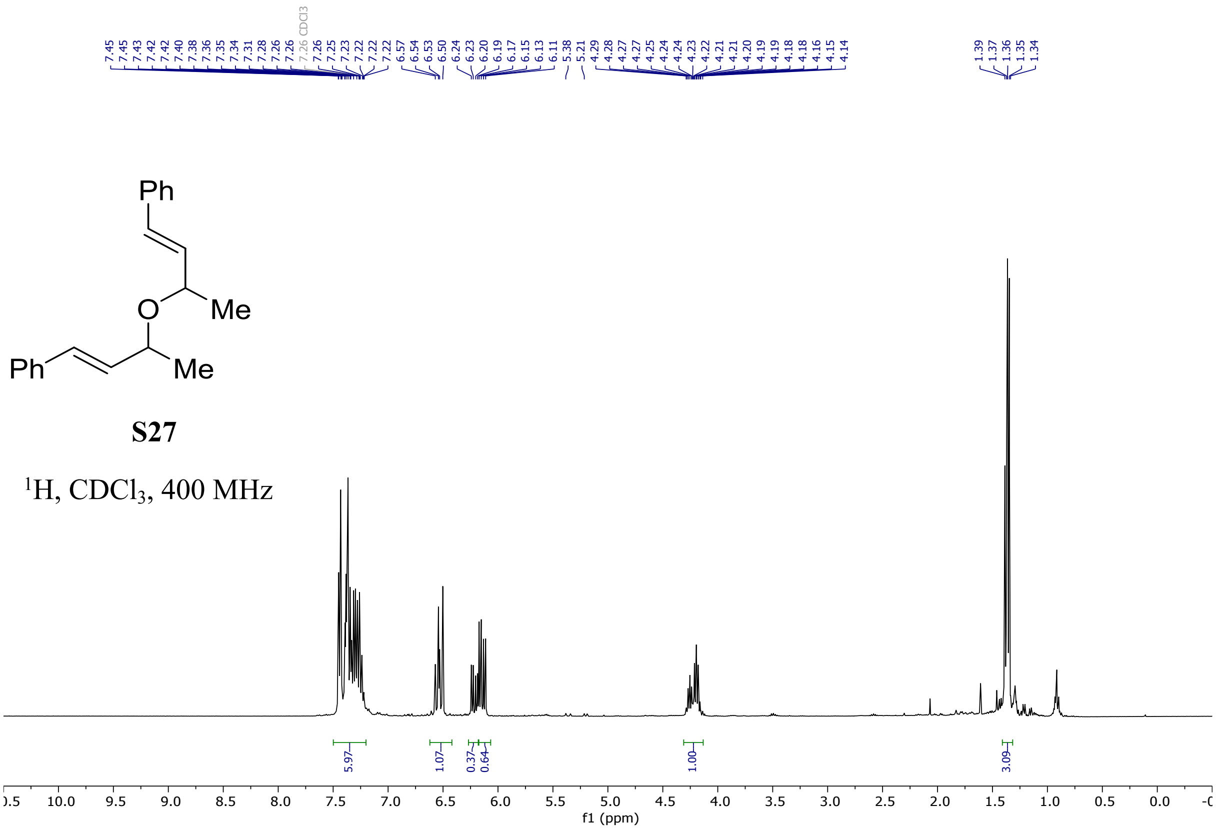

S27

^{1}H, $CDCl_3$, 400 MHz

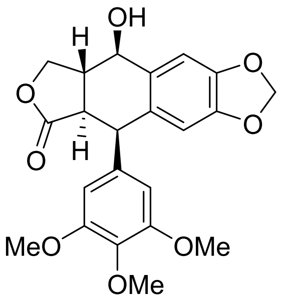

26

^{1}H, CDCl$_3$, 500 MHz

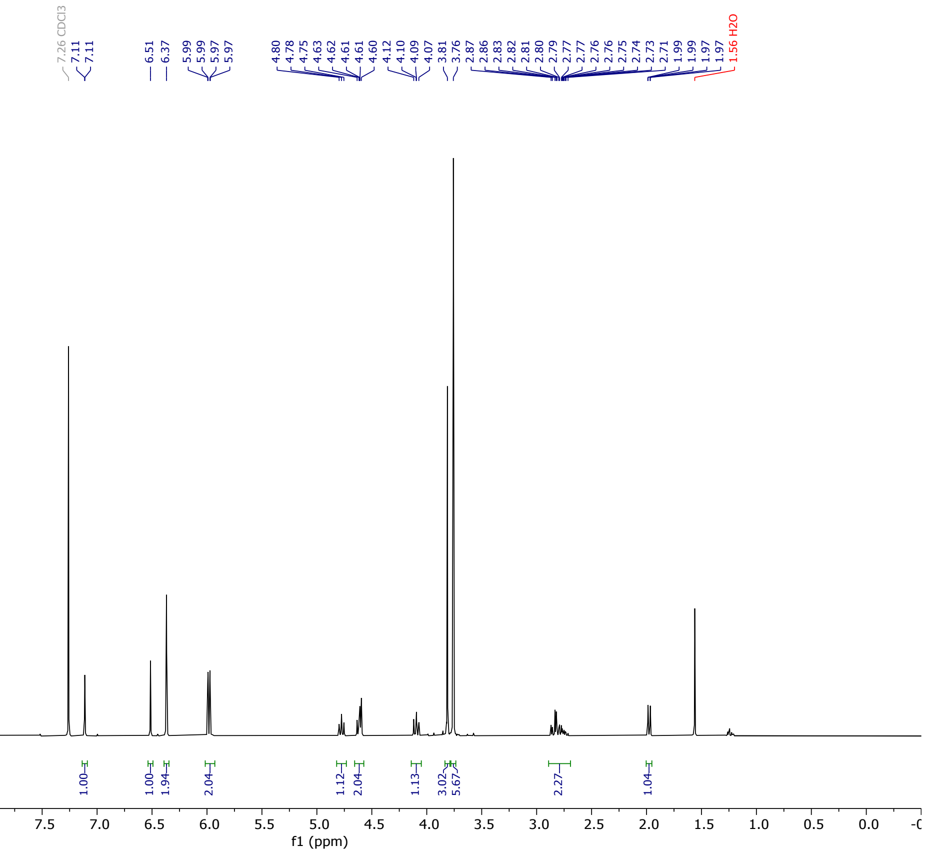

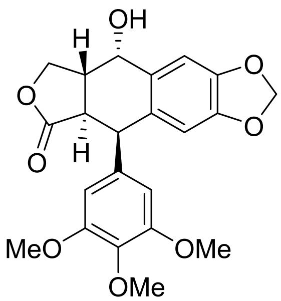

27

^{1}H, CDCl$_3$, 500 MHz

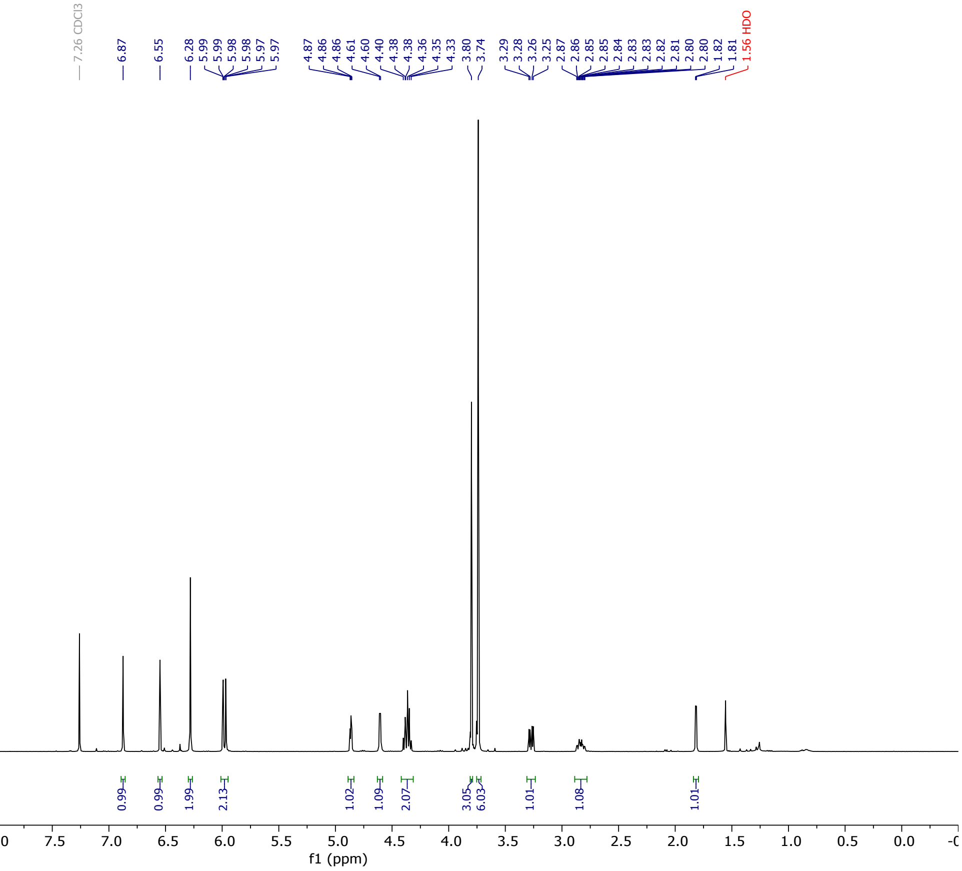

9. Appendix 2: HPLC Spectra

For Racemization: *First trace is the authentic racemic sample, the second trace is the enantioenriched alcohol from the kinetic resolution, and the third trace is the experimental sample following treatment with the boronic acid / oxalic acid under the conditions for (attempted) racemization.*

HPLC Data for **1-Benzyl-3-hydroxy-3-phenylindolin-2-one 3.3**: Chiralpak AD-H (90:10 hexane:IPA, flow rate 1.25 mL.min^{-1}, 211 nm, 40 °C) t_R (*R*): 16.9 min, t_R (*S*): 21.1 min, >99:1 (*R*:*S*) er.

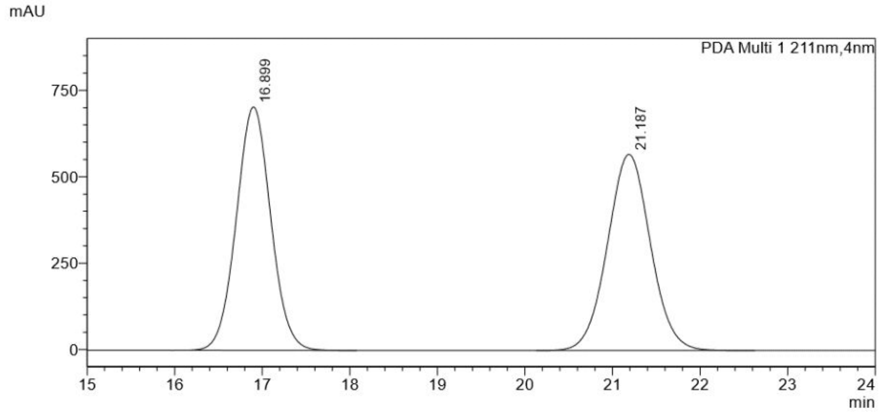

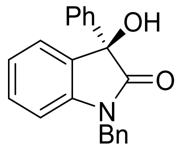

1

<Peak Table>

PDA Ch1 211nm

Peak#	Ret. Time	Area%
1	16.899	49.881
2	21.187	50.119
Total		100.000

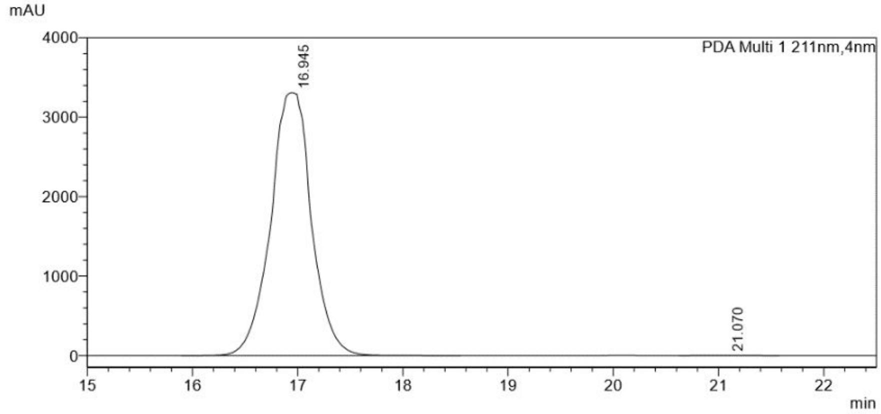

<Peak Table>

PDA Ch1 211nm

Peak#	Ret. Time	Area%
1	16.945	99.982
2	21.070	0.018
Total		100.000

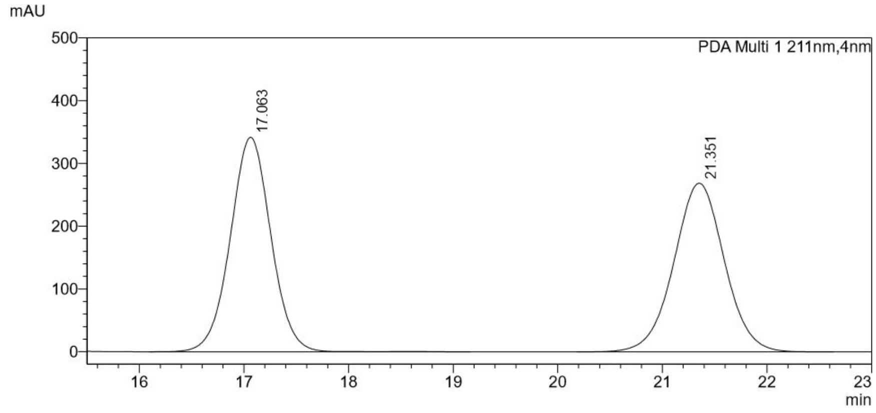

<Peak Table>

PDA Ch1 211nm

Peak#	Ret. Time	Area%
1	17.063	50.609
2	21.351	49.391
Total		100.000

HPLC data for **1-Methyl-3-hydroxy-3-phenylindolin-2-one 8**: Chiralpak AD-H (95:5 hexane:IPA, flow rate 1.0 mLmin^{-1}, 211 nm, 30 °C) t_R (*R*): 27.9 min, t_R (*S*): 31.0 min, >99:1 (*R*:*S*) er.

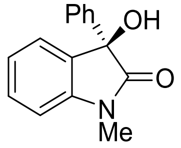

8

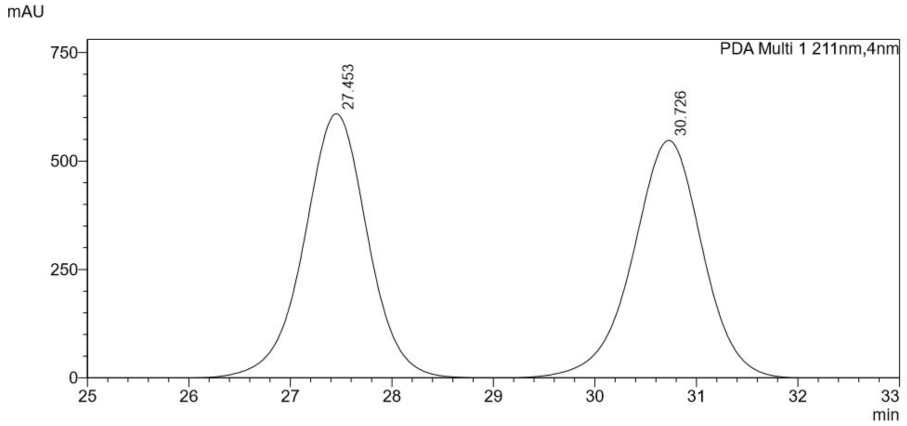

<Peak Table>

PDA Ch1 211nm

Peak#	Ret. Time	Area%
1	27.453	50.394
2	30.726	49.606
Total		100.000

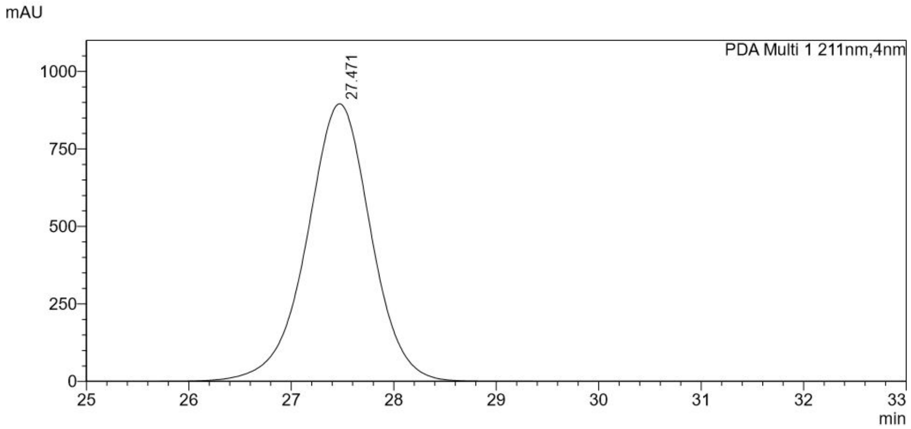

<Peak Table>

PDA Ch1 211nm

Peak#	Ret. Time	Area%
1	27.471	100.000
Total		100.000

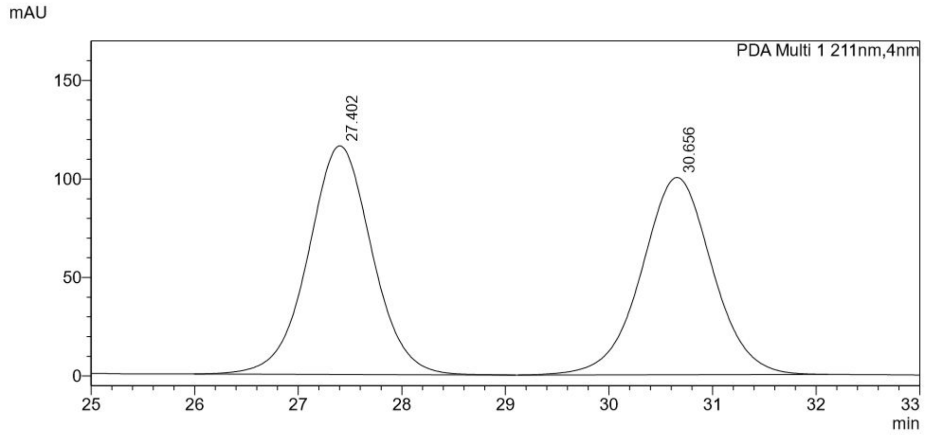

<Peak Table>

PDA Ch1 211nm

Peak#	Ret. Time	Area%
1	27.402	51.105
2	30.656	48.895
Total		100.000

HPLC data for **1-Allyl-3-hydroxy-3-phenylindolin-2-one 9**: Chiralpak OD-H (95:5 hexane:IPA, flow rate 1.0 mLmin^{-1}, 211 nm, 30 °C) t_R (*S*): 13.8 min, t_R (*R*): 15.7 min, 97:3 (*R*:*S*) er.

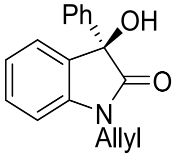

9

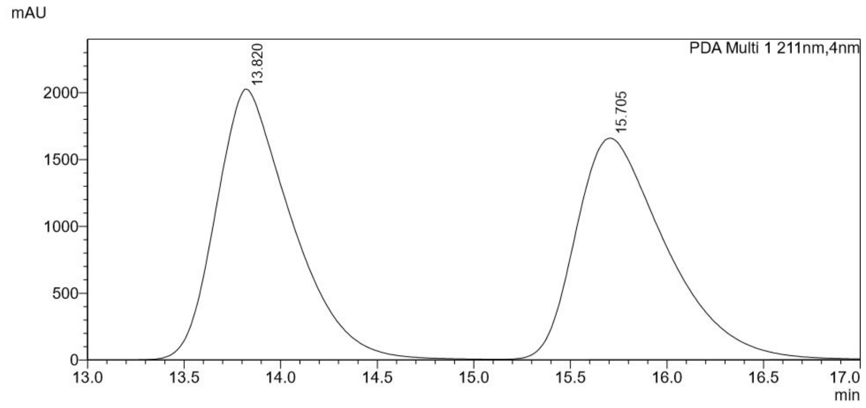

<Peak Table>

PDA Ch1 211nm

Peak#	Ret. Time	Area%
1	13.820	50.536
2	15.705	49.464
Total		100.000

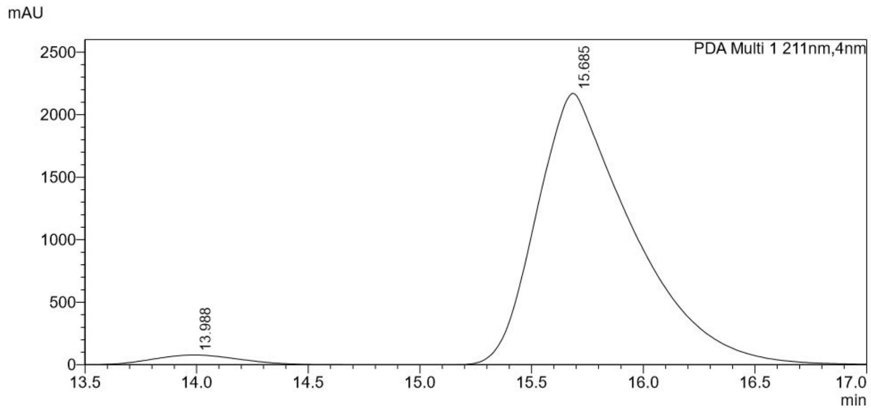

<Peak Table>

PDA Ch1 211nm

Peak#	Ret. Time	Area%
1	13.988	3.443
2	15.685	96.557
Total		100.000

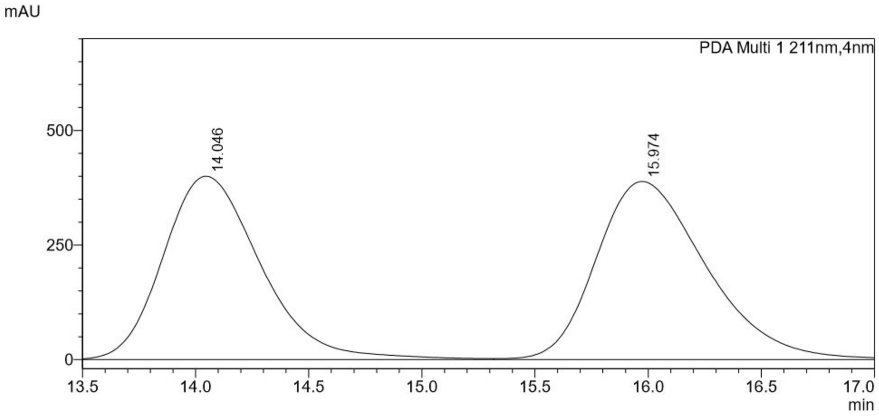

<Peak Table>

PDA Ch1 211nm

Peak#	Ret. Time	Area%
1	14.046	47.676
2	15.974	52.324
Total		100.000

HPLC data for **1-Benzyl-3-hydroxy-3-phenylindolin-2-one 10**: Chiralpak AD-H (90:10 hexane:IPA, flow rate 1.5 mLmin^{-1}, 211 nm, 40 °C) t_R (*R*): 12.3 min, t_R (*S*): 15.8 min, >99:1 (*R*:*S*) er. [32]

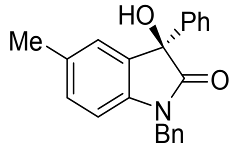

10

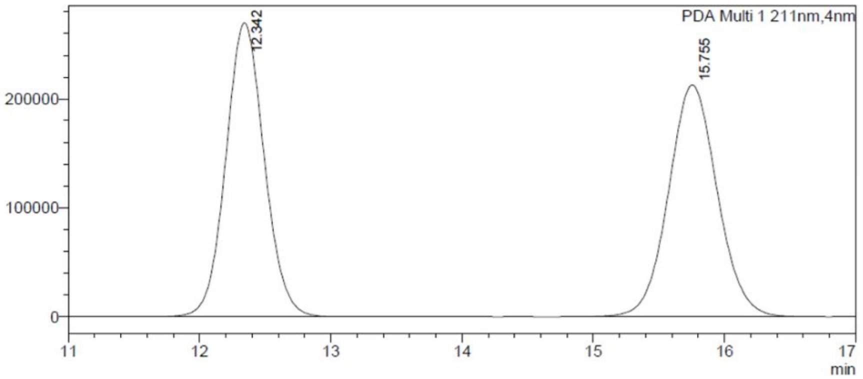

PDA Ch1 211nm

Peak#	Ret. Time	Area%
1	12.342	49.961
2	15.755	50.039
Total		100.000

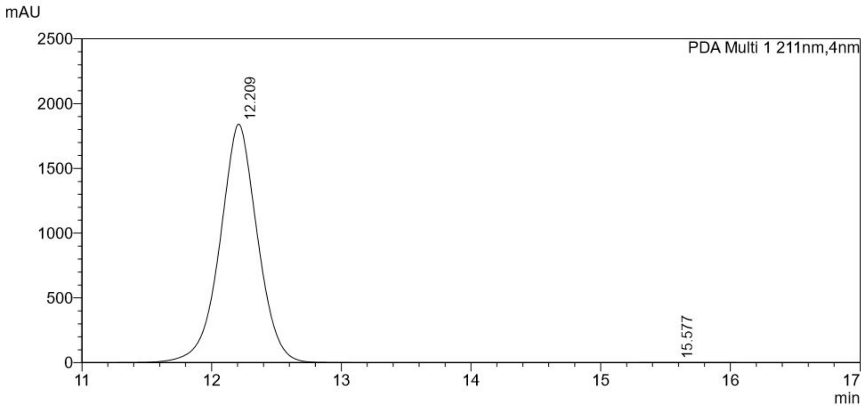

<Peak Table>

PDA Ch1 211nm

Peak#	Ret. Time	Area%
1	12.209	99.959
2	15.577	0.041
Total		100.000

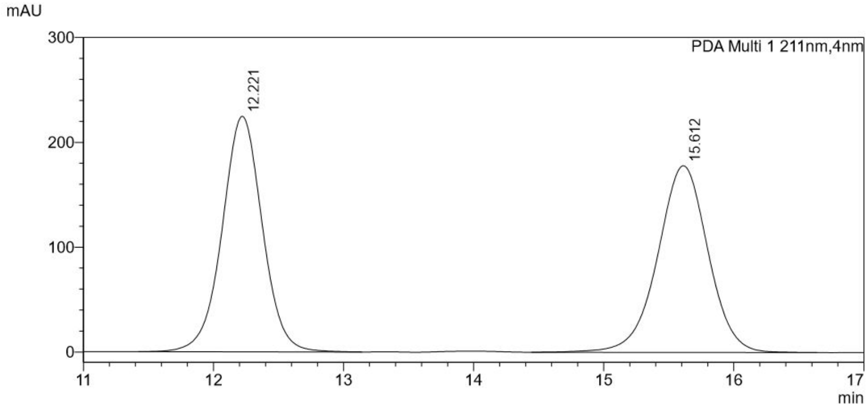

<Peak Table>

PDA Ch1 211nm

Peak#	Ret. Time	Area%
1	12.221	50.138
2	15.612	49.862
Total		100.000

HPLC data for **1-Benzyl-3-hydroxy-3-(4-methoyphenyl)indolin-2-one 11**: Chiralpak IC (80:20 hexane:IPA, flow rate 1.0 mLmin[-1], 211 nm, 30 °C) t_R (*R*): 15.9 min, t_R (*S*): 21.3 min, >99:1 (*R*:*S*) er.

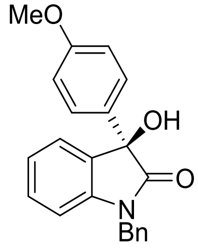

11

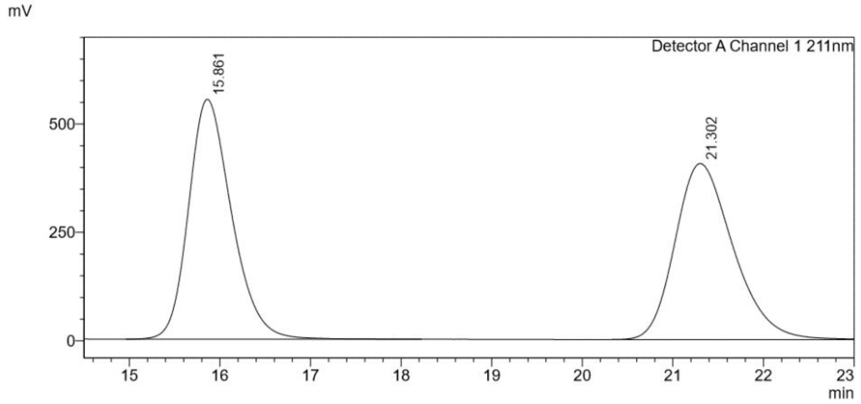

<Peak Table>

Detector A Channel 1 211nm

Peak#	Ret. Time	Area%
1	15.861	50.264
2	21.302	49.736
Total		100.000

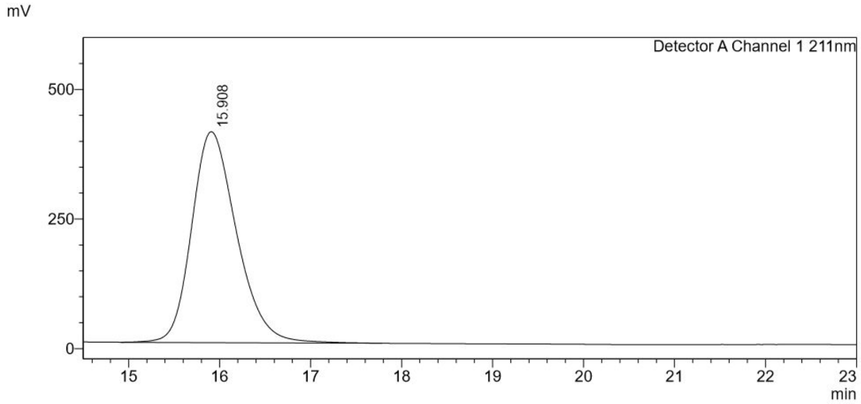

<Peak Table>

Detector A Channel 1 211nm

Peak#	Ret. Time	Area%
1	15.908	100.000
Total		100.000

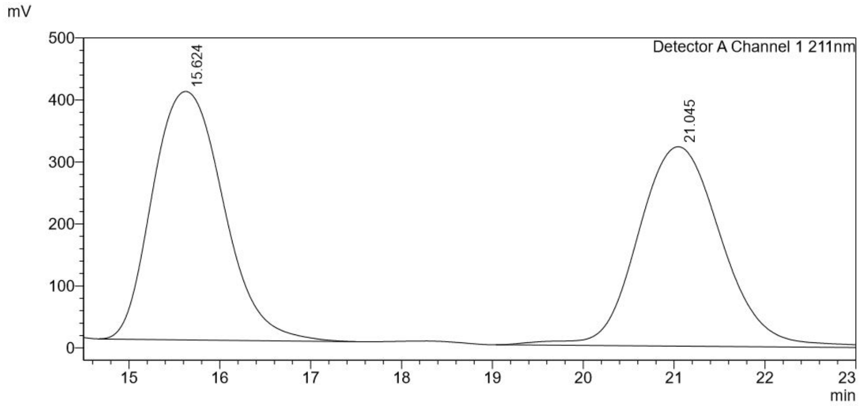

<Peak Table>

Detector A Channel 1 211nm

Peak#	Ret. Time	Area%
1	15.624	51.151
2	21.045	48.849
Total		100.000

HPLC data for **1-Benzyl-3-hydroxy-3-(naphthalen-2-yl)indolin-2-one 12**: Chiralpak IA (70:30 hexane:IPA, flow rate 0.5 mLmin^{-1}, 211 nm, 30 °C) t_R (*R*): 24.4 min, t_R (*S*): 30.3 min, >99:1 (*R*:*S*) er.

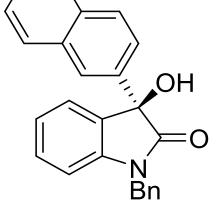

12

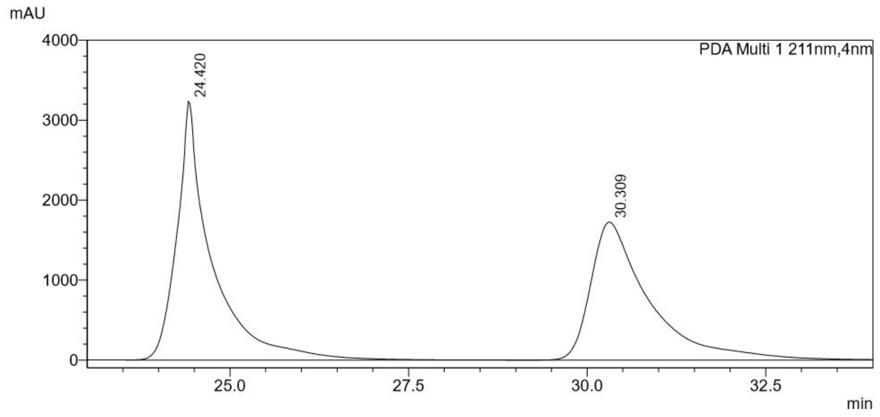

<Peak Table>

PDA Ch1 211nm

Peak#	Ret. Time	Area%
1	24.420	52.620
2	30.309	47.380
Total		100.000

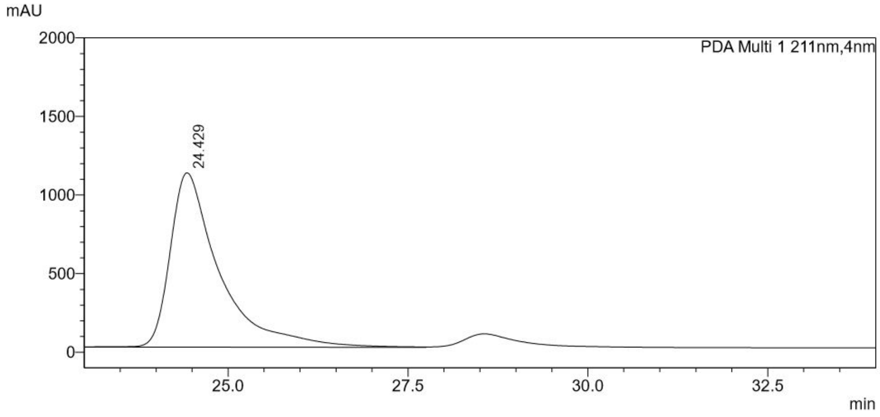

<Peak Table>

PDA Ch1 211nm

Peak#	Ret. Time	Area%
1	24.429	100.000
Total		100.000

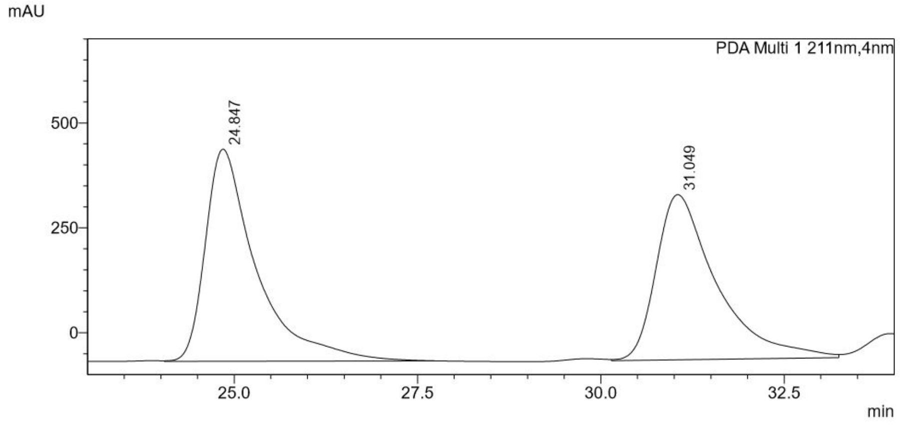

<Peak Table>

PDA Ch1 211nm

Peak#	Ret. Time	Area%
1	24.847	52.058
2	31.049	47.942
Total		100.000

HPLC data for **3-Allyl-1-benzyl-3-hydroxyindolin-2-one 13**: Chiralpak OD-H (98:2 hexane:IPA, flow rate 1.0 mLmin^{-1}, 254 nm, 30 °C) t_R (*R*): 32.1 min, t_R (*S*): 38.4 min, >99:1 (*R*:*S*) er.

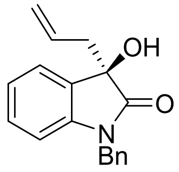

13

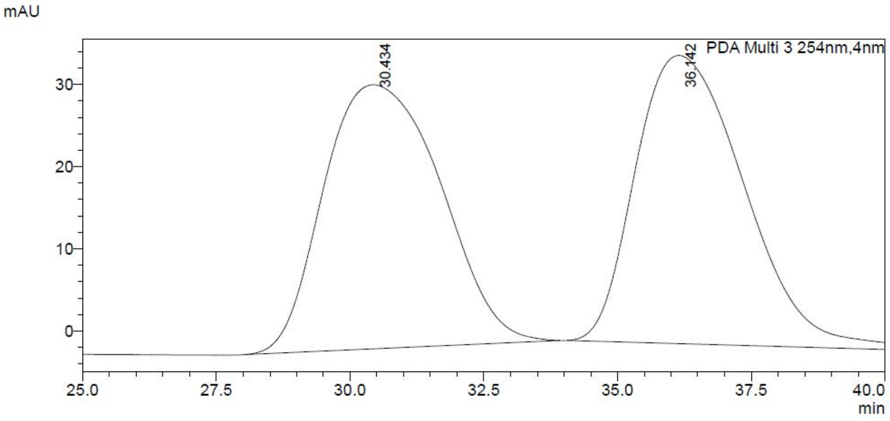

<Peak Table>

PDA Ch3 254nm

Peak#	Ret. Time	Area%
1	30.434	49.682
2	36.142	50.318
Total		100.000

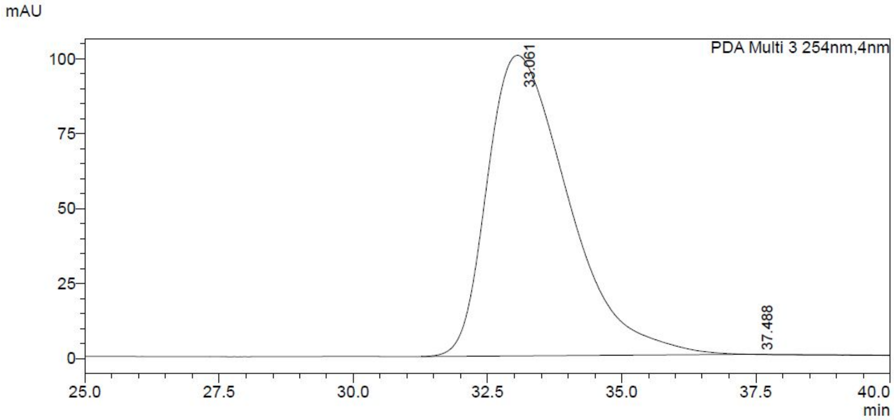

<Peak Table>

PDA Ch3 254nm

Peak#	Ret. Time	Area%
1	33.061	100.143
2	37.488	-0.143
Total		100.000

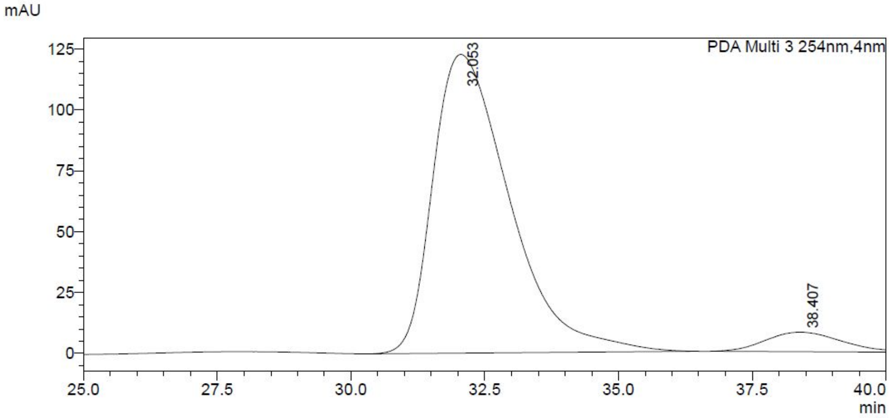

<Peak Table>

PDA Ch3 254nm

Peak#	Ret. Time	Area%
1	32.053	93.879
2	38.407	6.121
Total		100.000

HPLC data for **1-Benzyl-3-ethyl-3-hydroxylindolin-2-one 14**: Chiralpak IC (80:20 hexane:IPA, flow rate 1.0 mLmin^{-1}, 211 nm, 30 °C) t_R (*R*): 7.7 min, t_R (*S*): 12.6 min, >99:1 (*R*:*S*) er.

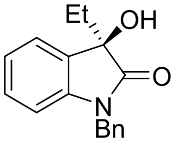

14

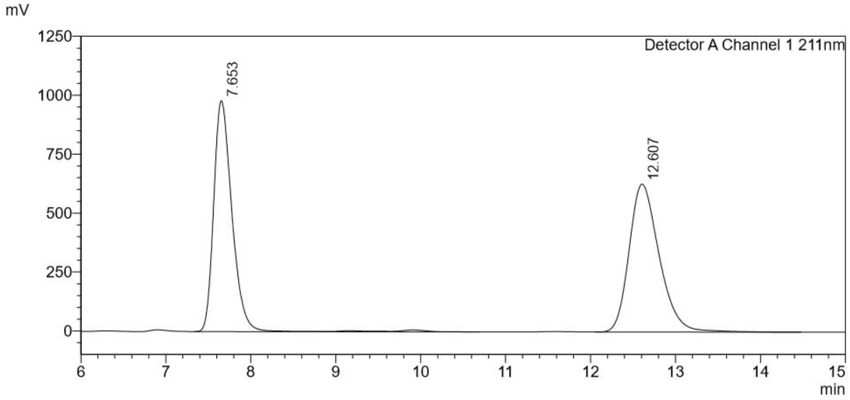

<Peak Table>

Detector A Channel 1 211nm

Peak#	Ret. Time	Area%
1	7.653	48.596
2	12.607	51.404
Total		100.000

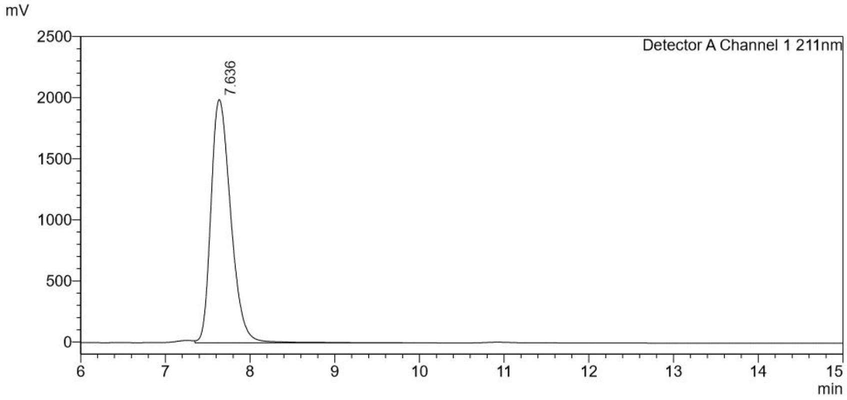

<Peak Table>

Detector A Channel 1 211nm

Peak#	Ret. Time	Area%
1	7.636	100.000
Total		100.000

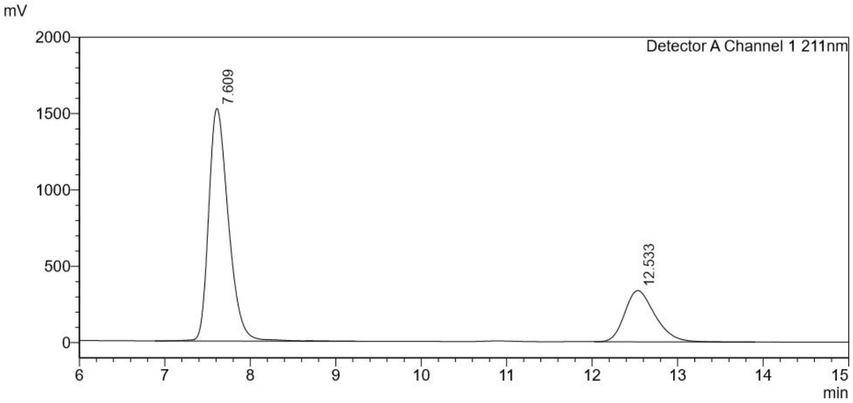

<Peak Table>

Detector A Channel 1 211nm

Peak#	Ret. Time	Area%
1	7.609	74.563
2	12.533	25.437
Total		100.000

HPLC data for **1-Benzyl-3-hydroxy-isopropylindolin-2-one 15**: Chiralpak AD-H (95:5 hexane:IPA, flow rate 1.0 mLmin^{-1}, 211 nm, 30 °C) t_R (*R*): 23.4 min, t_R (*S*): 28.5 min, >99:1 (*R*:*S*) er.

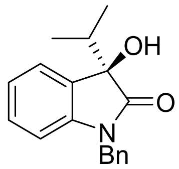

15

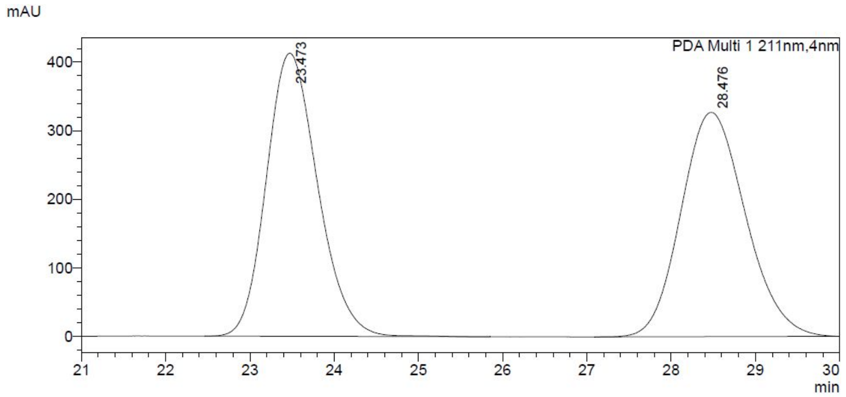

<Peak Table>

PDA Ch1 211nm

Peak#	Ret. Time	Area%
1	23.473	50.141
2	28.476	49.859
Total		100.000

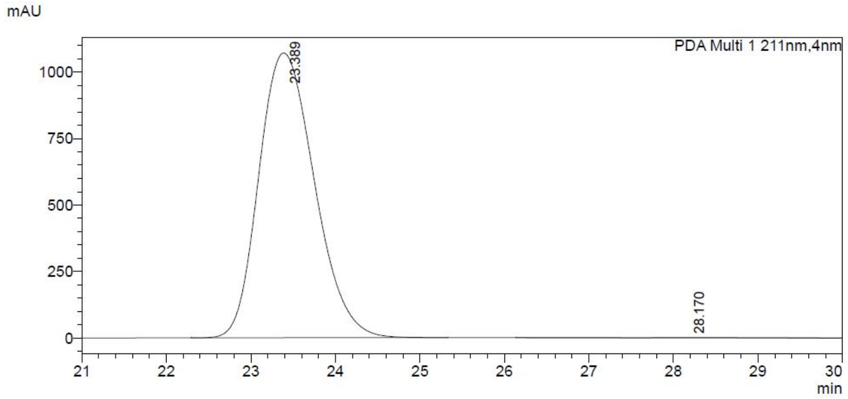

<Peak Table>

PDA Ch1 211nm

Peak#	Ret. Time	Area%
1	23.389	99.961
2	28.170	0.039
Total		100.000

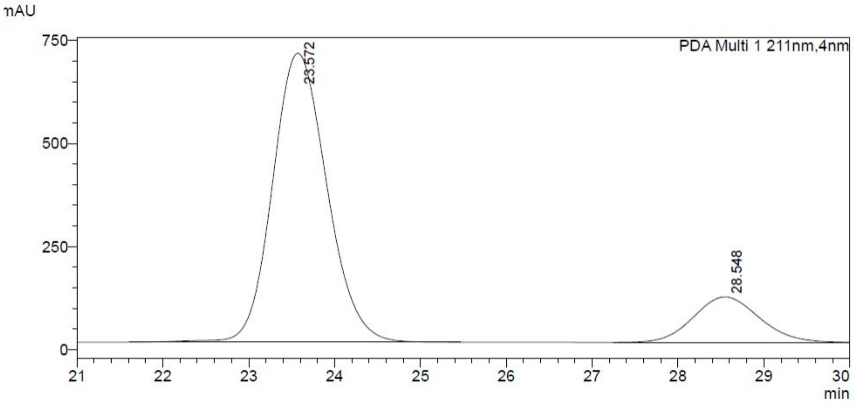

<Peak Table>

PDA Ch1 211nm

Peak#	Ret. Time	Area%
1	23.572	83.975
2	28.548	16.025
Total		100.000

HPLC data for **1-Phenylethanol 16**: Chiralpak OD-H (95:5 hexane:IPA, flow rate 1.0 mLmin^{-1}, 220 nm, 30 °C) t_R (*R*) : 8.2 min, t_R (*S*): 9.9 min, >99:1 (*S*:*R*) er.

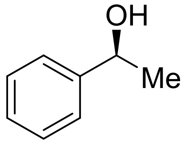

16

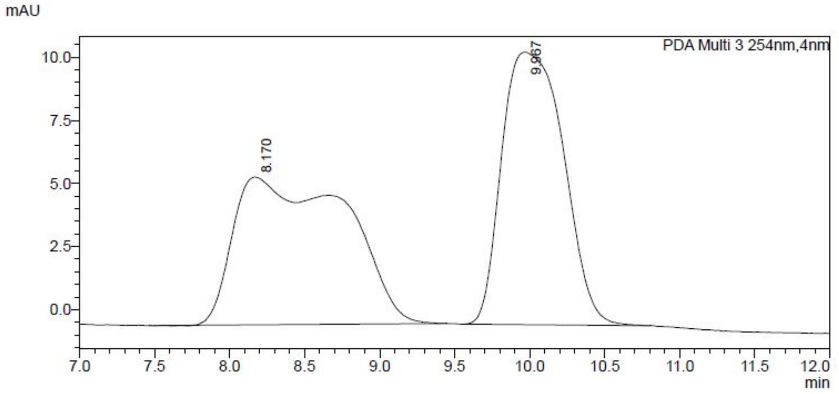

<Peak Table>

PDA Ch3 254nm

Peak#	Ret. Time	Area%
1	8.170	49.873
2	9.967	50.127
Total		100.000

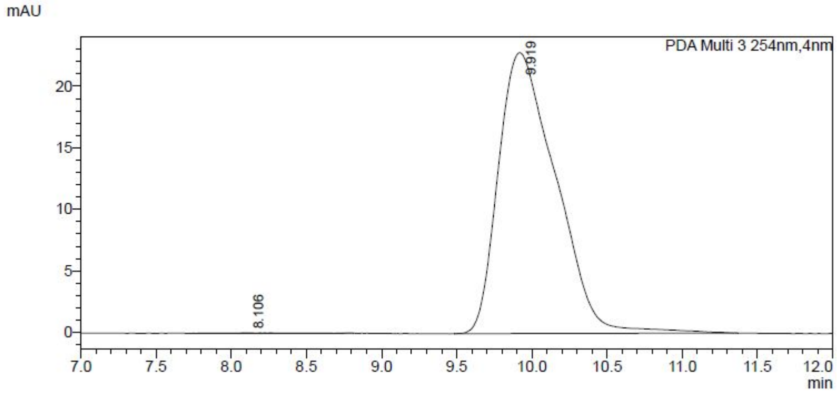

<Peak Table>

PDA Ch3 254nm

Peak#	Ret. Time	Area%
1	8.106	0.165
2	9.919	99.835
Total		100.000

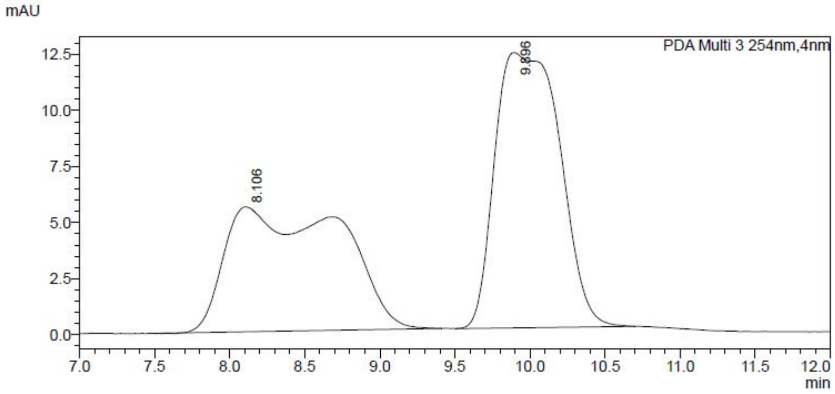

<Peak Table>

PDA Ch3 254nm

Peak#	Ret. Time	Area%
1	8.106	44.737
2	9.896	55.263
Total		100.000

HPLC data for **2-Methyl-1-phenylpropanol 17**: Chiralpak AD-H (99.5:0.5 hexane:IPA, flow rate 1.0 mLmin^{-1}, 220 nm, 30 °C) t_R (*R*) : 18.5 min, t_R (*S*): 20.2 min, >99:1 (*S*:*R*) er.

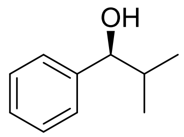

17

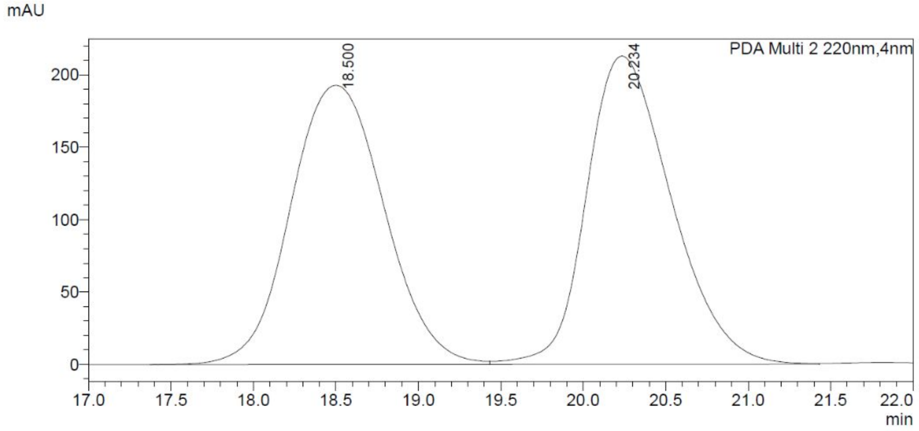

<Peak Table>

PDA Ch2 220nm

Peak#	Ret. Time	Area%
1	18.500	50.083
2	20.234	49.917
Total		100.000

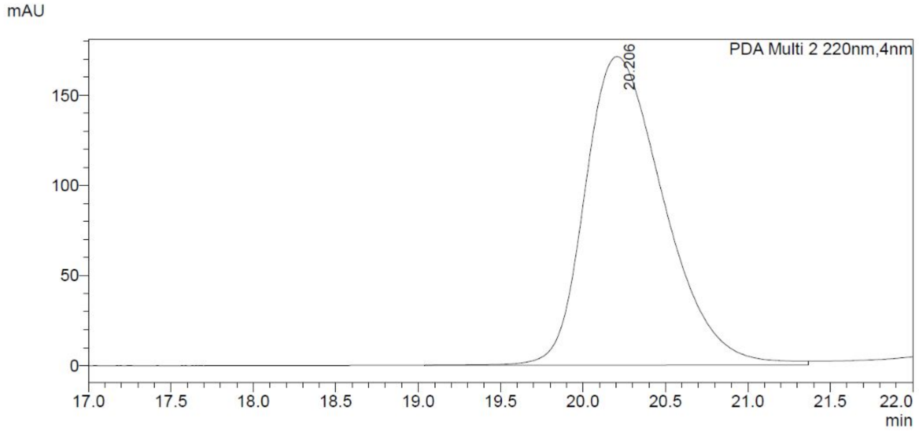

<Peak Table>

PDA Ch2 220nm

Peak#	Ret. Time	Area%
1	20.206	100.000
Total		100.000

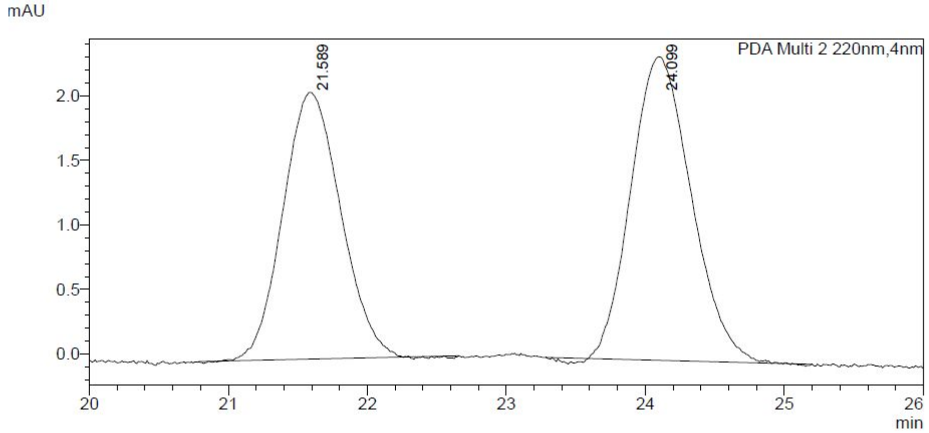

<Peak Table>

PDA Ch2 220nm

Peak#	Ret. Time	Area%
1	21.589	45.768
2	24.099	54.232
Total		100.000

HPLC data for **2,2-Dimethyl-1-phenylpropanol 18**: Chiralpak OD-H (95:5 hexane:IPA, flow rate 1.0 mLmin^{-1}, 211 nm, 30 °C) t_R (*S*): 6.4 min, t_R (*R*): 9.0 min, >99:1 (*S*:*R*) er.

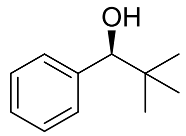

18

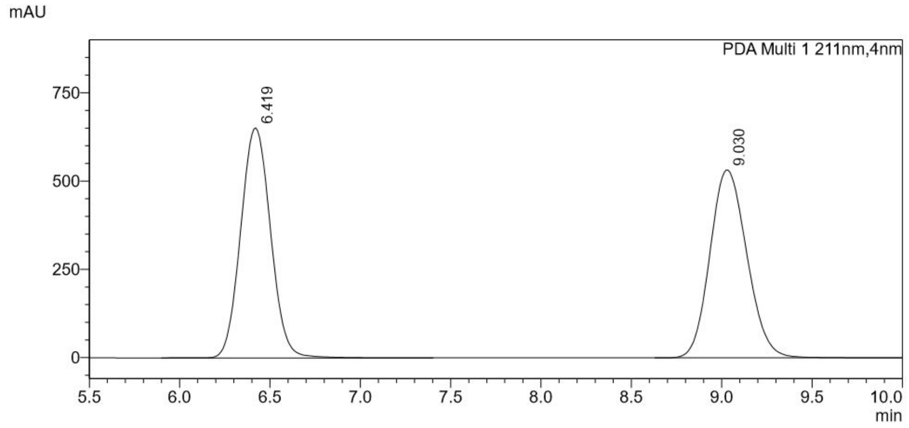

<Peak Table>

PDA Ch1 211nm

Peak#	Ret. Time	Area%
1	6.419	49.699
2	9.030	50.301
Total		100.000

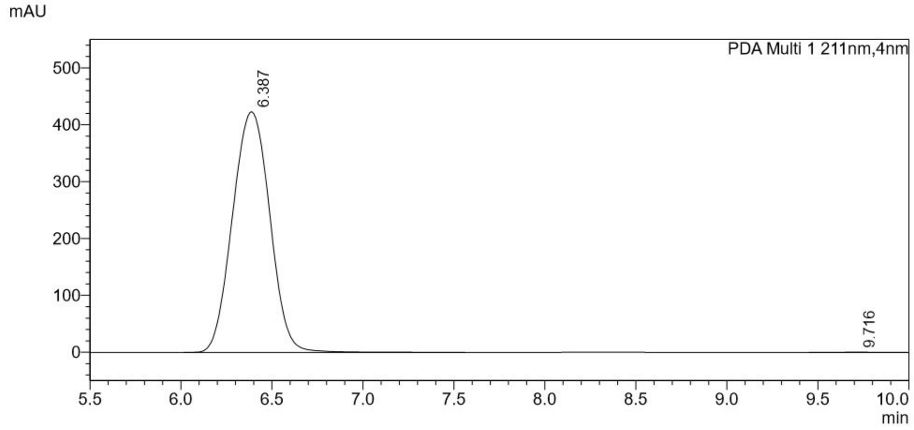

<Peak Table>

PDA Ch1 211nm

Peak#	Ret. Time	Area%
1	6.387	99.931
2	9.716	0.069
Total		100.000

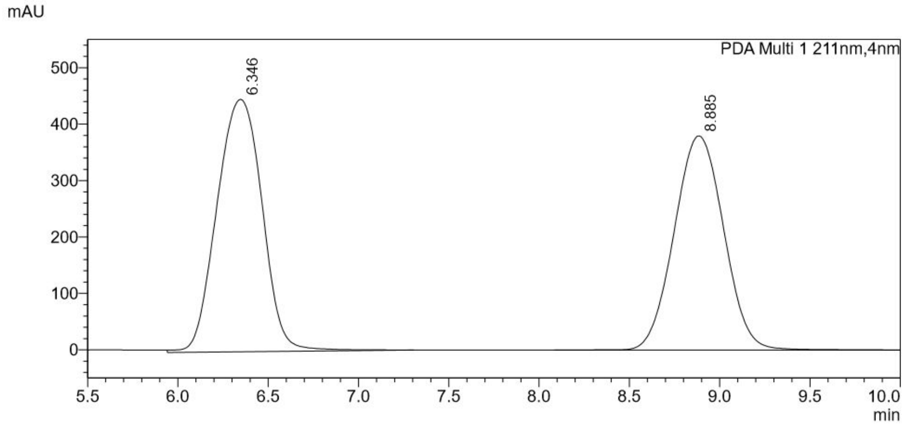

<Peak Table>

PDA Ch1 211nm

Peak#	Ret. Time	Area%
1	6.346	51.927
2	8.885	48.073
Total		100.000

HPLC data for **2,2-Dimethyl-1-(4-(trifluoromethyl)phenyl)propan-1-ol 19**: Chiralpak OJ-H (99:1 hexane:IPA, flow rate 1.0 mLmin^{-1}, 220 nm, 30 °C) t_R (*S*): 9.4 min, t_R (*R*): 10.3 min, 97:3 (*S*:*R*) er.

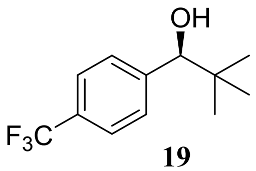

19

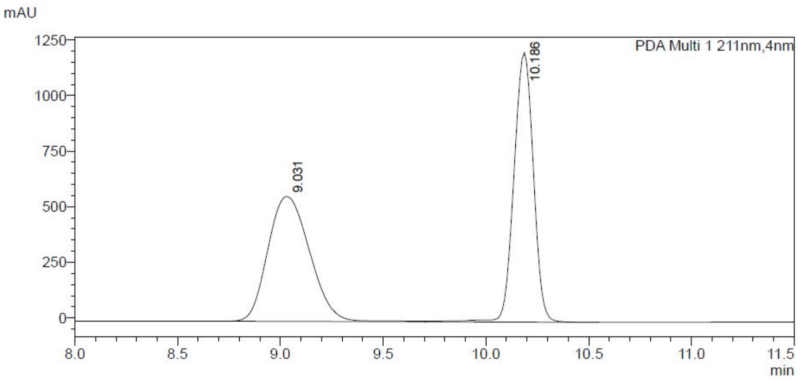

<Peak Table>

PDA Ch1 211nm

Peak#	Ret. Time	Area%
1	9.031	49.866
2	10.186	50.134
Total		100.000

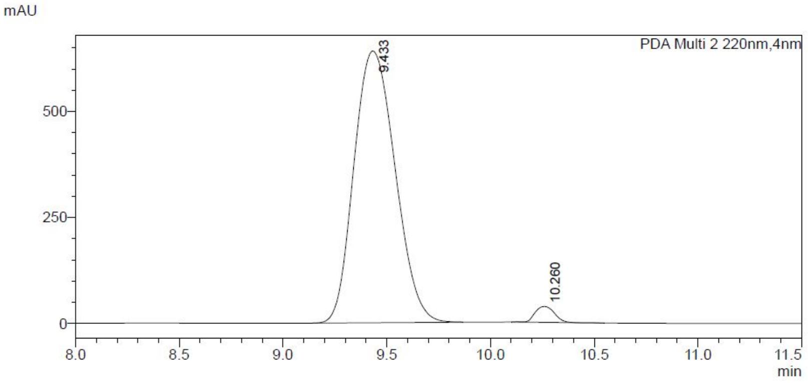

<Peak Table>

PDA Ch2 220nm

Peak#	Ret. Time	Area%
1	9.433	97.365
2	10.260	2.635
Total		100.000

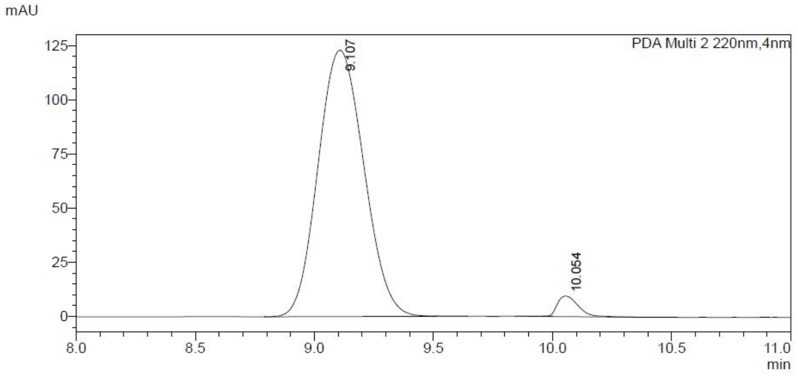

<Peak Table>

PDA Ch2 220nm

Peak#	Ret. Time	Area%
1	9.107	96.522
2	10.054	3.478
Total		100.000

HPLC data for **1-(4-Chlorophenyl)-2,2-dimethylpropan-1-ol 20**: Chiralpak IC (99.8:0.2 hexane:IPA, flow rate 1.0 mLmin^{-1}, 211 nm, 30 °C) t_R (*S*): 7.7 min, t_R (*R*): 7.2 min, 98:2 (*S*:*R*) er.

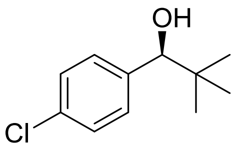

20

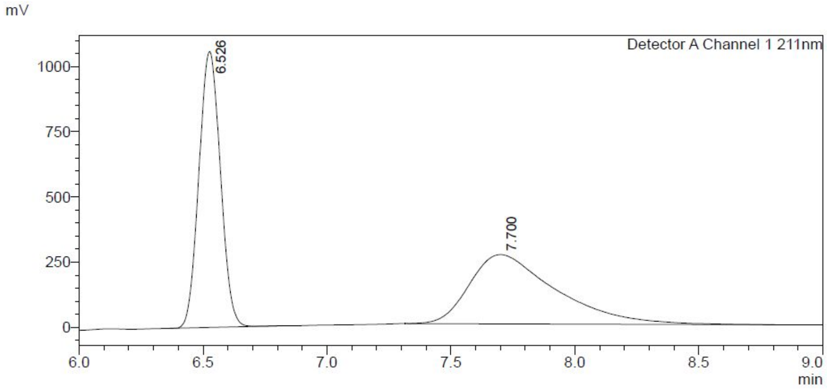

Peak#	Ret. Time	Area%
1	6.526	49.848
2	7.700	50.152
Total		100.000

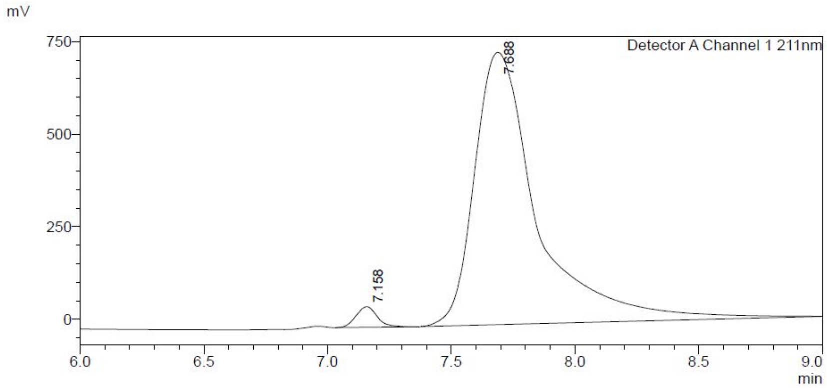

Peak#	Ret. Time	Area%
1	7.158	2.472
2	7.688	97.528
Total		100.000

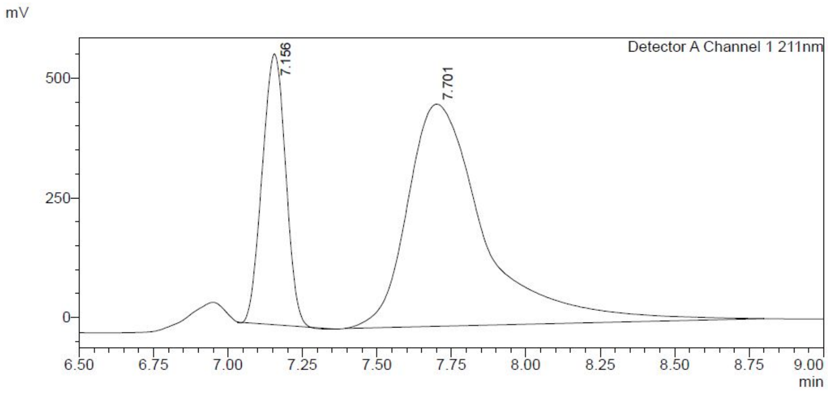

Peak#	Ret. Time	Area%
1	7.156	26.885
2	7.701	73.115
Total		100.000

HPLC data for **2,2-Dimethyl-1-(*p*-tolyl)propan-1-ol**: Chiralpak OJ-H (98:2 hexane:IPA, flow rate 1.0 mLmin^{-1}, 220 nm, 30 °C) t_R (*S*): 5.7 min, t_R (*R*): 6.2 min, >99:1 (*S*:*R*) er.

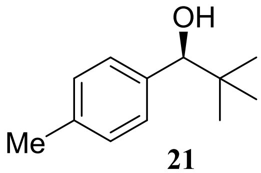

21

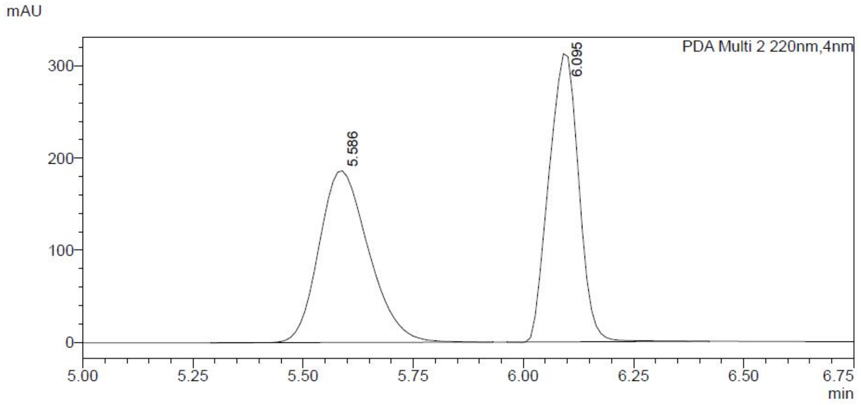

<Peak Table>

PDA Ch2 220nm

Peak#	Ret. Time	Area%
1	5.586	49.832
2	6.095	50.168
Total		100.000

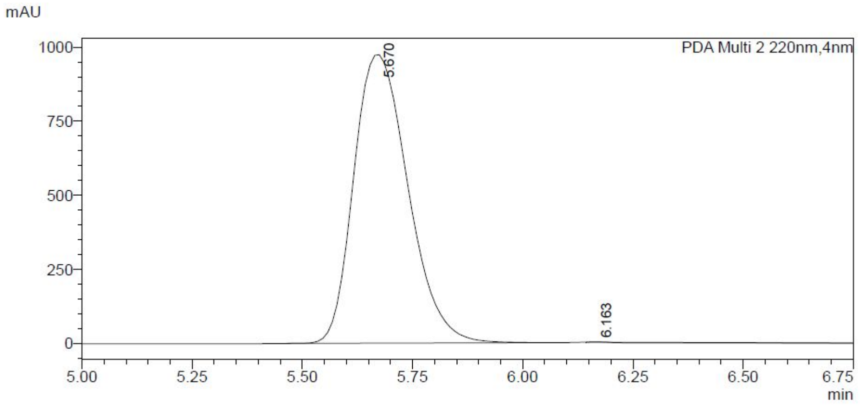

<Peak Table>

PDA Ch2 220nm

Peak#	Ret. Time	Area%
1	5.670	100.484
2	6.163	-0.484
Total		100.000

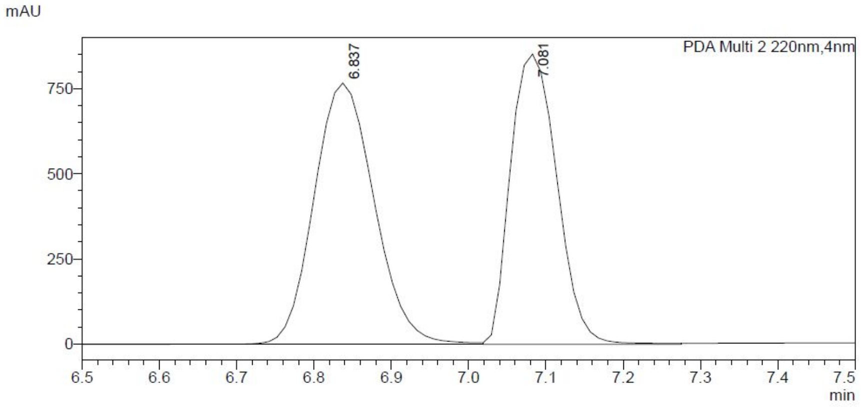

<Peak Table>

PDA Ch2 220nm

Peak#	Ret. Time	Area%
1	6.837	53.915
2	7.081	46.085
Total		100.000

HPLC data for **2,2-Dimethyl-1-(naphthalen-2-yl)propan-1-ol 22**: Chiralpak OJ-H (99:1 hexane:IPA, flow rate 1.0 mLmin^{-1}, 220 nm, 30 °C) t_R (*S*): 22.3 min, t_R (*R*): 28.3 min, 97:3 (*S*:*R*) er.

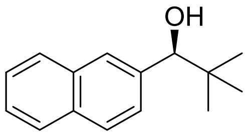

22

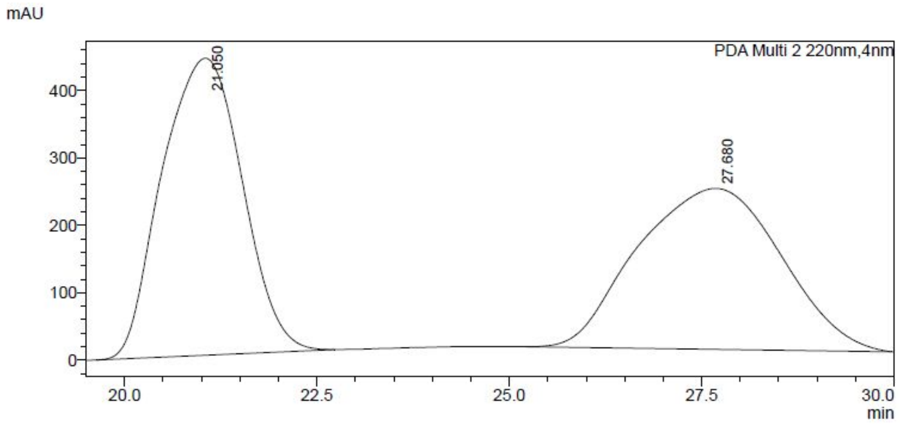

<Peak Table>

PDA Ch2 220nm

Peak#	Ret. Time	Area%
1	21.050	50.843
2	27.680	49.157
Total		100.000

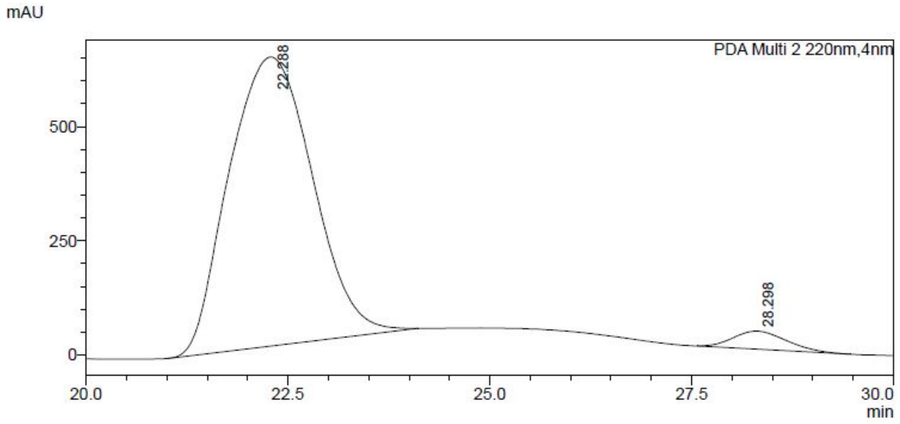

<Peak Table>

PDA Ch2 220nm

Peak#	Ret. Time	Area%
1	22.288	96.189
2	28.298	3.811
Total		100.000

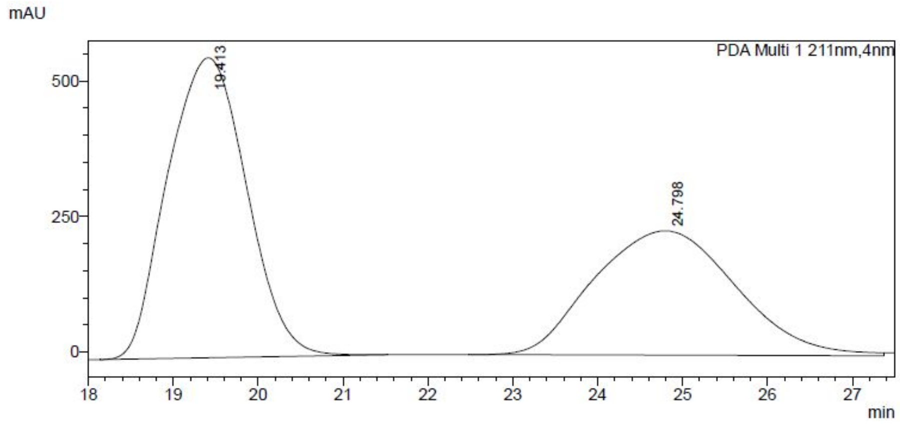

<Peak Table>

PDA Ch2 220nm

Peak#	Ret. Time	Area%
1	19.414	55.719
2	24.796	44.281
Total		100.000

HPLC data for **1-(4-Methoxyphenyl)-2,2,-dimethylpropan-1-ol 23**: Chiralpak AD-H (99:1 hexane:IPA, flow rate 1.0 mLmin^{-1}, 220 nm, 30 °C) t_R (*R*): 21.1 min, t_R (*S*): 23.3 min, >99:1 (*S*:*R*) er.

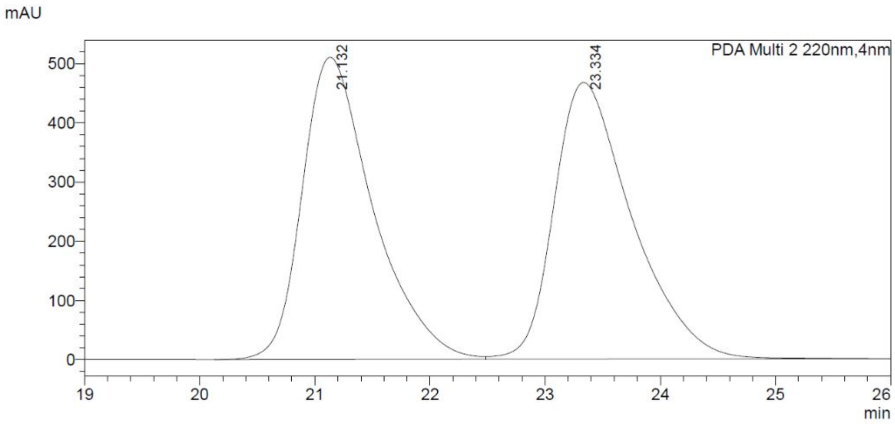

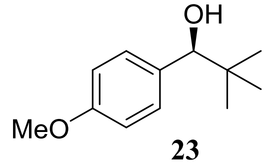

23

<Peak Table>

PDA Ch2 220nm

Peak#	Ret. Time	Area%
1	21.132	49.787
2	23.334	50.213
Total		100.000

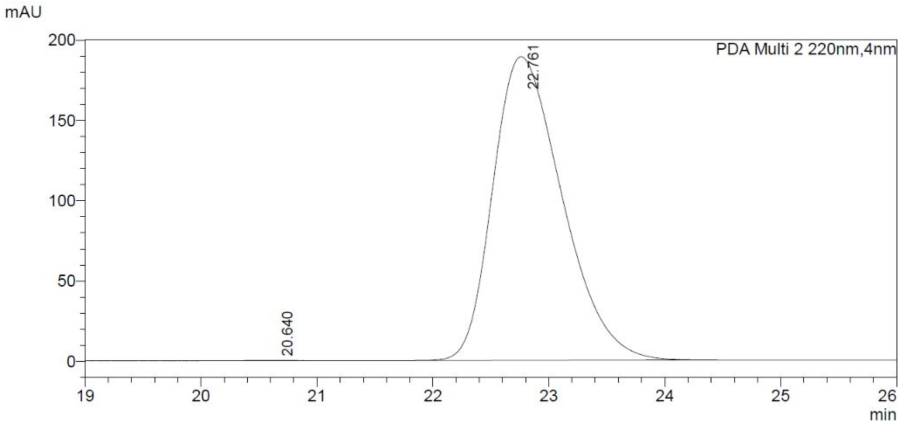

<Peak Table>

PDA Ch2 220nm

Peak#	Ret. Time	Area%
1	20.640	-0.000
2	22.761	100.000
Total		100.000

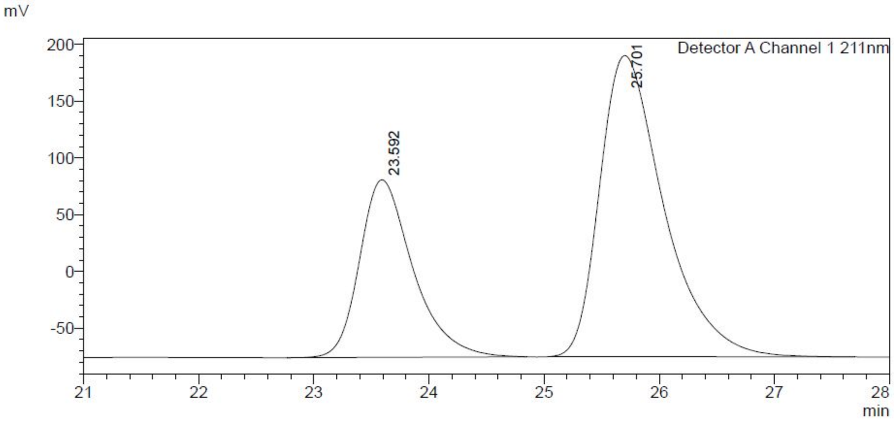

Peak#	Ret. Time	Area%
1	23.592	32.916
2	25.701	67.084
Total		100.000

HPLC data for **4-Phenylbut-3-yn-2-ol 24**: Chiralpak OD-H (95:5 hexane:IPA, flow rate 1.0 mLmin^{-1}, 254 nm, 30 °C) t_R (*S*): 28.1 min, t_R (*R*): 10.8 min, >99:1 (*S*:*R*) er.

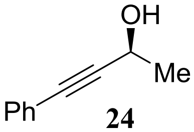

24

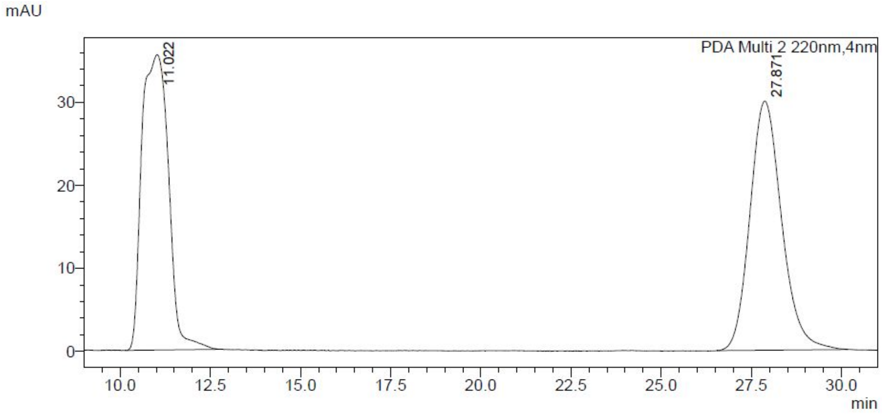

<Peak Table>

PDA Ch2 220nm

Peak#	Ret. Time	Area%
1	11.022	50.000
2	27.871	50.000
Total		100.000

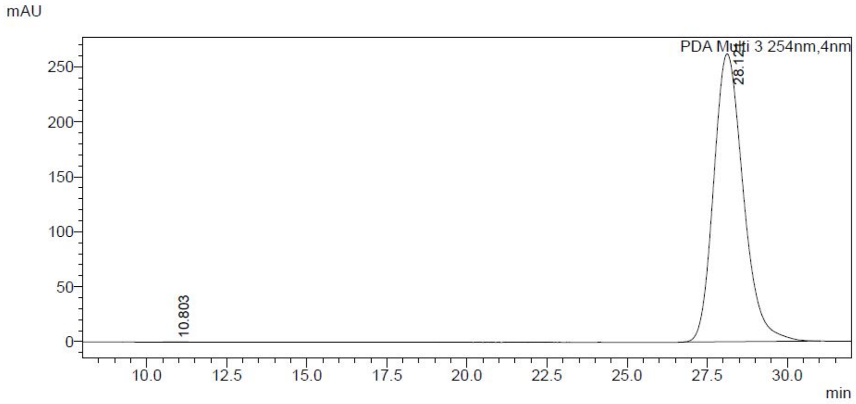

<Peak Table>

PDA Ch3 254nm

Peak#	Ret. Time	Area%
1	10.803	0.015
2	28.121	99.985
Total		100.000

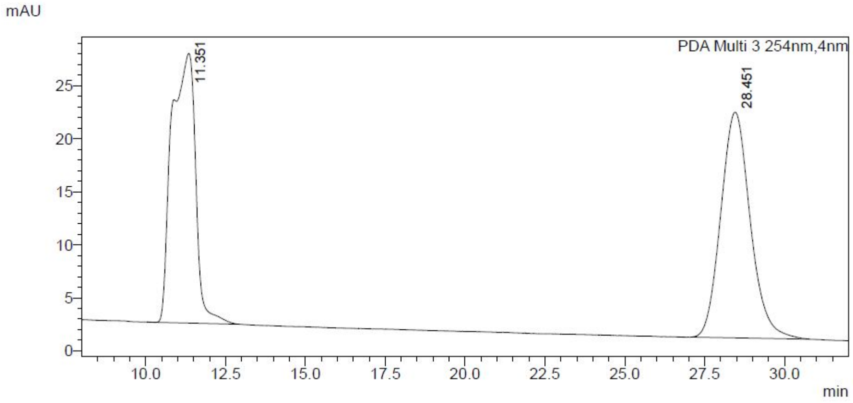

<Peak Table>

PDA Ch3 254nm

Peak#	Ret. Time	Area%
1	11.351	49.206
2	28.451	50.794
Total		100.000

HPLC data for **(*E*)-4-Phenylbut-3-en-2-ol 25**: Chiralpak OD-H (95:5 hexane:IPA, flow rate 1.0 mLmin^{-1}, 220 nm, 30 °C) t_R (*S*): 25.0 min, t_R (*R*): 15.4 min, 96:4 (*S*:*R*) er.

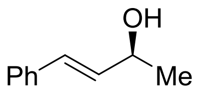

25

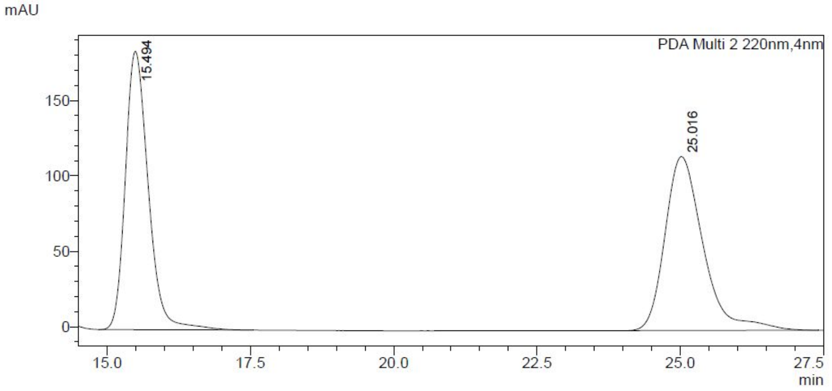

<Peak Table>

PDA Ch2 220nm

Peak#	Ret. Time	Area%
1	15.494	49.104
2	25.016	50.896
Total		100.000

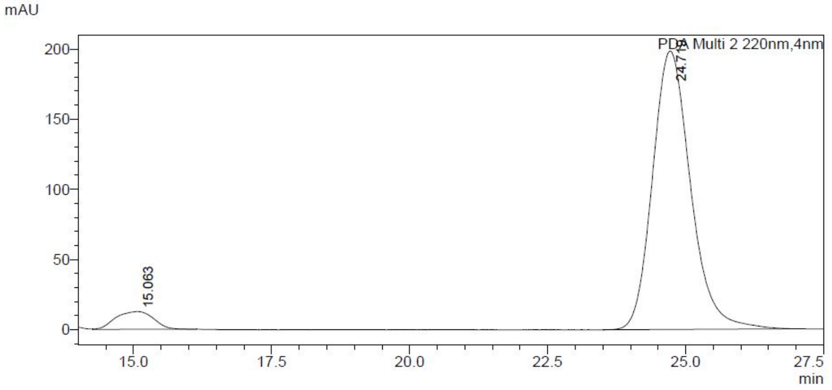

<Peak Table>

PDA Ch2 220nm

Peak#	Ret. Time	Area%
1	15.063	5.965
2	24.719	94.035
Total		100.000

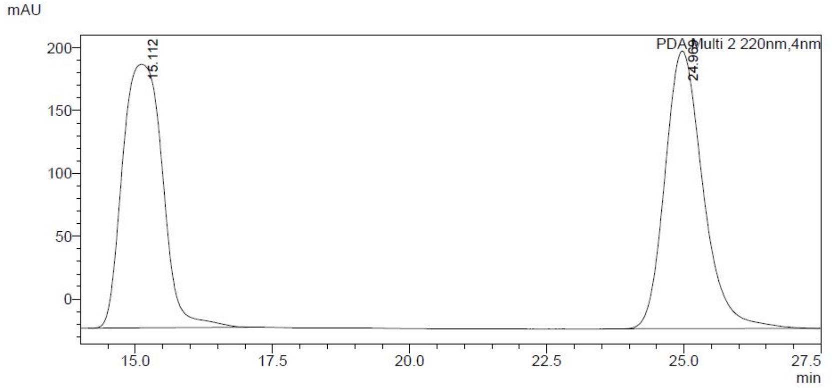

<Peak Table>

PDA Ch2 220nm

Peak#	Ret. Time	Area%
1	15.112	49.995
2	24.969	50.005
Total		100.000

HPLC data for esters from the kinetic resolution.

HPLC data for **1-Benzyl-2-oxo-3-phenylindolin-3-yl isobutyrate S5**: Chiralpak AD-H (90:10 hexane:IPA, flow rate 1.25 mLmin^{-1}, 211 nm, 40 °C) t_R (*R*): 10.0 min, t_R (*S*): 20.0 min, 77:23 (*S*:*R*) er. [9]

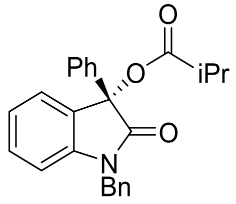

S5

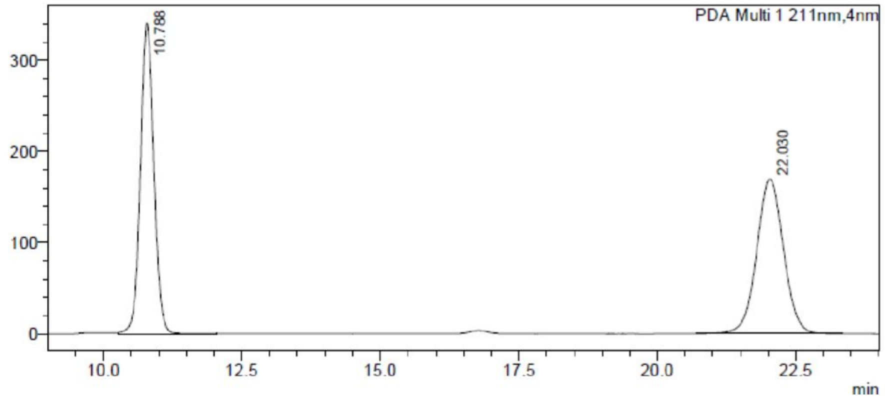

PDA Ch1 211nm

Peak#	Ret. Time	Area%
1	10.788	50.122
2	22.030	49.878
Total		100.000

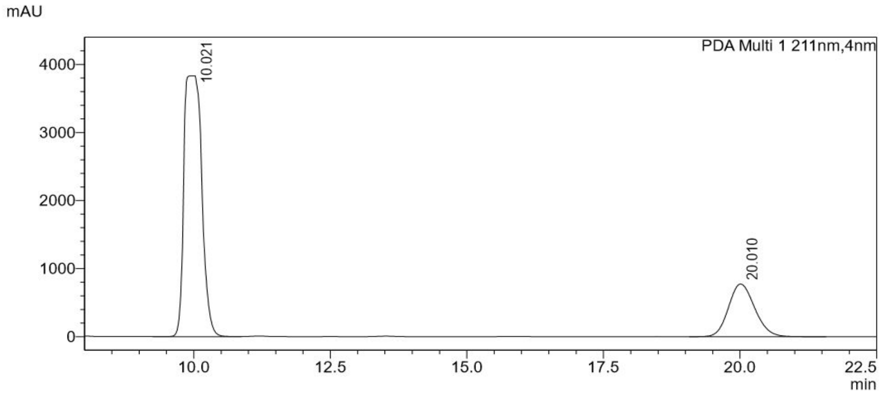

<Peak Table>

PDA Ch1 211nm

Peak#	Ret. Time	Area%
1	10.021	77.027
2	20.010	22.973
Total		100.000

HPLC data for **1-Methyl-2-oxo-3-phenylindolin-3-yl isobutyrate S6**: Chiralpak AD-H (95:5 hexane:IPA, flow rate 1.0 mLmin^{-1}, 211 nm, 30 °C) t_R (*S*): 8.8 min, t_R (*R*): 12.0 min, 89:11 (*S*:*R*) er. [32]

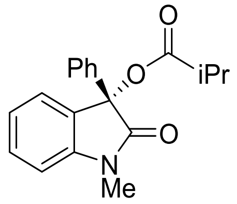

S6

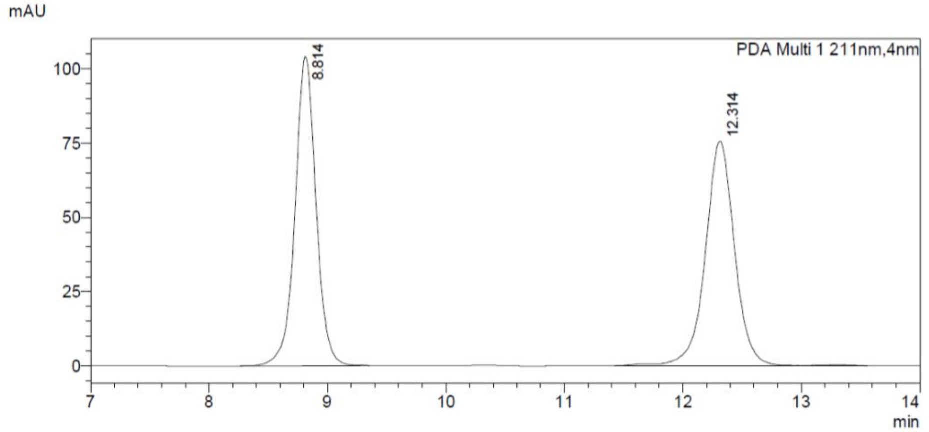

<Peak Table>

PDA Ch1 211nm

Peak#	Ret. Time	Area%
1	8.814	49.600
2	12.314	50.400
Total		100.000

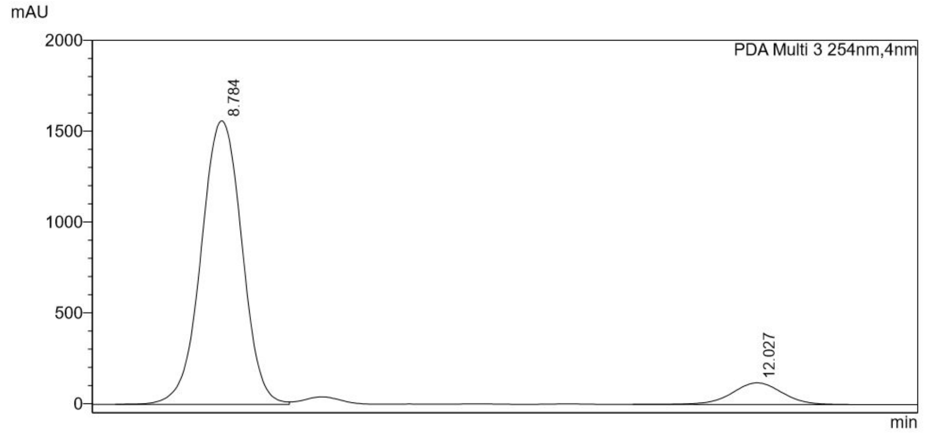

<Peak Table>

PDA Ch3 254nm

Peak#	Ret. Time	Area%
1	8.784	91.371
2	12.027	8.629
Total		100.000

HPLC data for **1-Allyl-2-oxo-3-phenylindolin-3-yl isobutyrate S7**: Chiralpak OD-H (99:1 hexane:IPA, flow rate 1.0 mLmin^{-1}, 211 nm, 30 °C) t_R (*R*): 11.3 min, t_R (*S*): 12.4 min, 98:2 (*S*:*R*) er. [32]

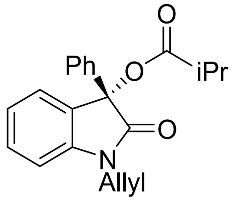

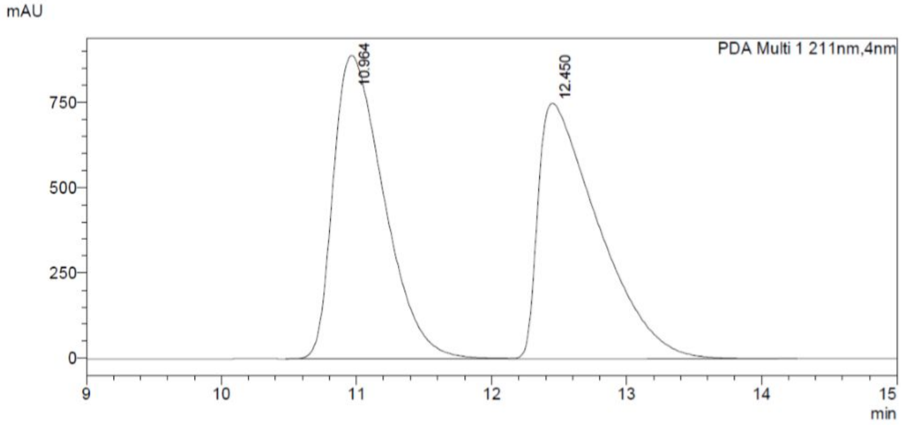

<Peak Table>

PDA Ch1 211nm

Peak#	Ret. Time	Area%
1	10.964	49.832
2	12.450	50.168
Total		100.000

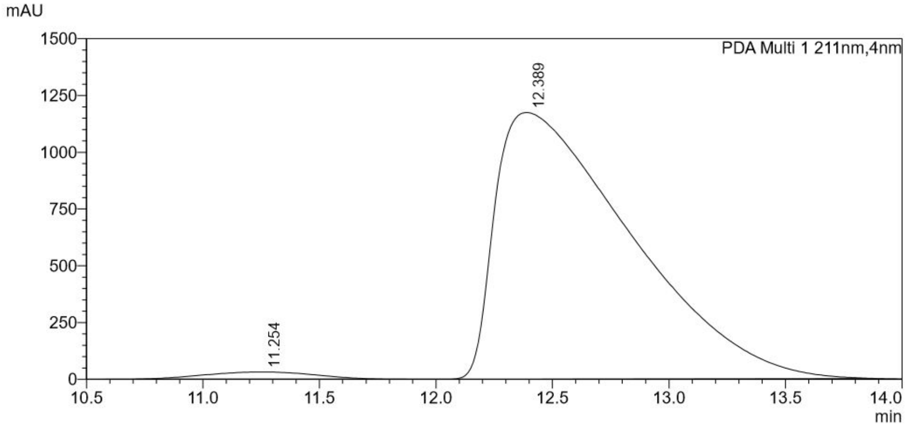

<Peak Table>

PDA Ch1 211nm

Peak#	Ret. Time	Area%
1	11.254	2.247
2	12.389	97.753
Total		100.000

HPLC data for **1-Benzyl-2-oxo-3-phenylindolin-3-yl isobutyrate S8**: Chiralpak AD-H (95:5 hexane:IPA, flow rate 1.5 mLmin^{-1}, 211 nm, 40 °C) t_R (*S*): 11.2 min, t_R (*R*): 25.0 min, 90:10 (*S*:*R*) er. [32]

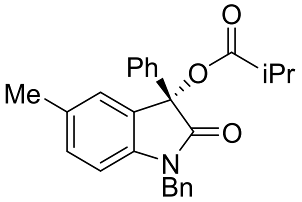

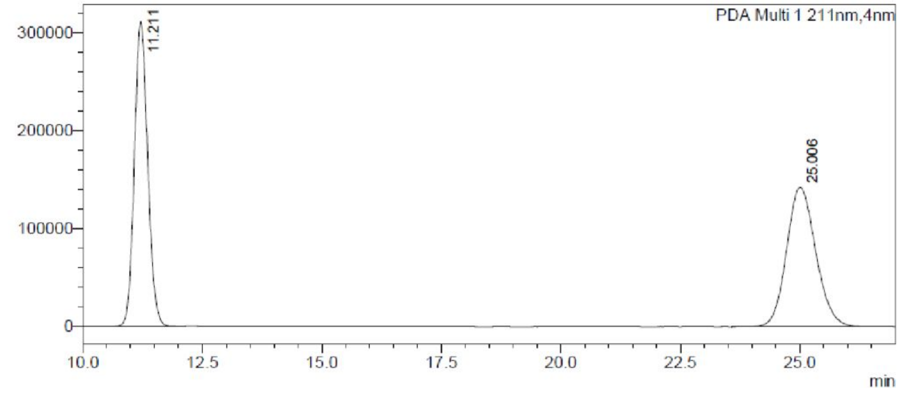

PDA Ch1 211nm

Peak#	Ret. Time	Area%
1	11.211	49.997
2	25.006	50.003
Total		100.000

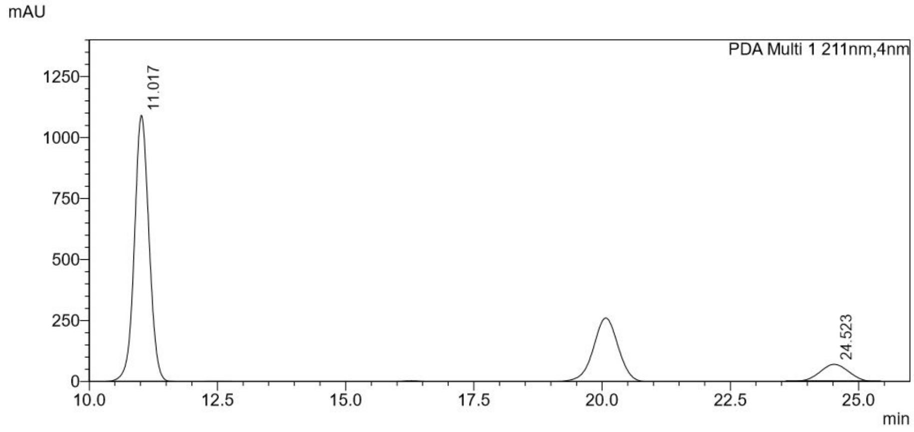

<Peak Table>

PDA Ch1 211nm

Peak#	Ret. Time	Area%
1	11.017	89.908
2	24.523	10.092
Total		100.000

HPLC data for **1-Benzyl-2-oxo-3-(4-methoxyphenyl)indolin-3-yl isobutyrate S9**: Chiralpak IA (80:20 hexane:IPA, flow rate 1.0 mLmin^{-1}, 211 nm, 30 °C) t_R (*S*): 15.3 min, t_R (*R*): 19.7 min, 77:23 (*S*:*R*) er.

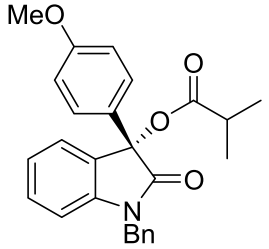

S9

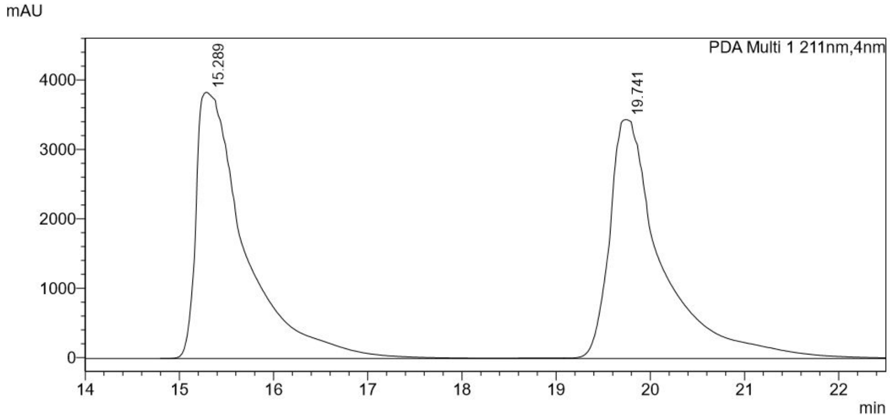

<Peak Table>

PDA Ch1 211nm

Peak#	Ret. Time	Area%
1	15.289	50.747
2	19.741	49.253
Total		100.000

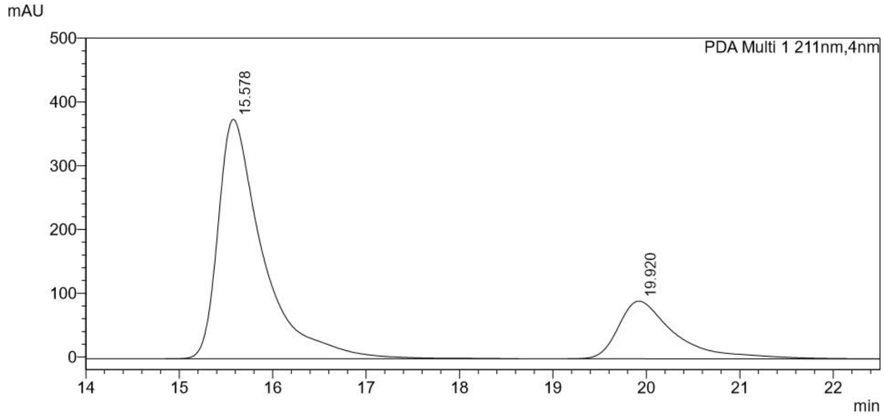

<Peak Table>

PDA Ch1 211nm

Peak#	Ret. Time	Area%
1	15.578	76.648
2	19.920	23.352
Total		100.000

HPLC data for **1-Benzyl-2-oxo-3-(naphthalen-2-yl)indolin-3-yl isobutyrate S10**: Chiralpak IA (70:30 hexane:IPA, flow rate 1.0 mLmin^{-1}, 211 nm, 30 °C) t_R (*S*): 14.2 min, t_R (*R*): 31.2 min, 84:17 (*S*:*R*) er.

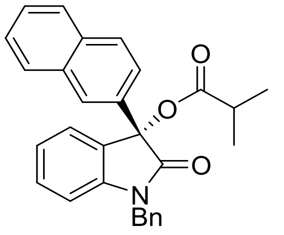

S10

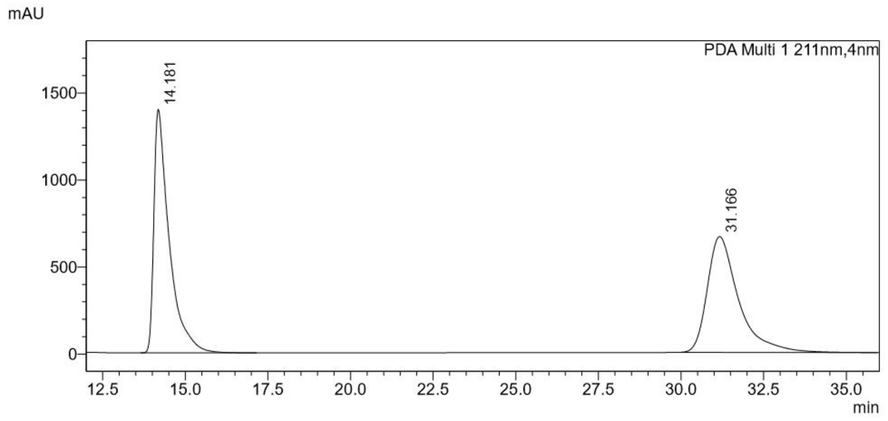

<Peak Table>

PDA Ch1 211nm

Peak#	Ret. Time	Area%
1	14.181	50.574
2	31.166	49.426
Total		100.000

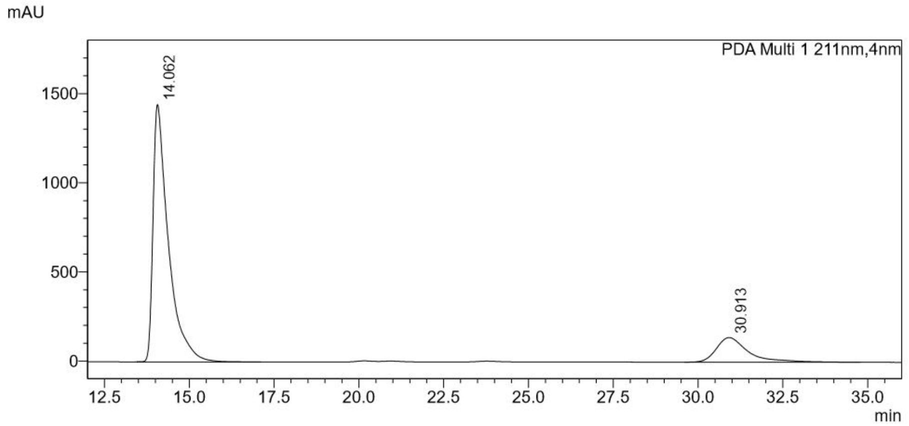

<Peak Table>

PDA Ch1 211nm

Peak#	Ret. Time	Area%
1	14.062	83.473
2	30.913	16.527
Total		100.000

HPLC data for ***(S)*-3-Allyl-1-benzyl-2-oxoindolin-3-yl isobutyrate S11** where the conditions were previously described by our group[2]: Chiralpak AD-H, (98:2 hexane:IPA, flow rate 1.0 mLmin^{-1}, 211 nm, 30 °C) t_R (*S*): 14.8 min, t_R (*R*): 16.7 min, 87:13 (*S*:*R*) er.

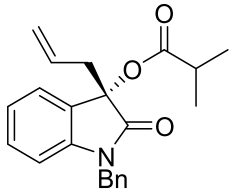

S11

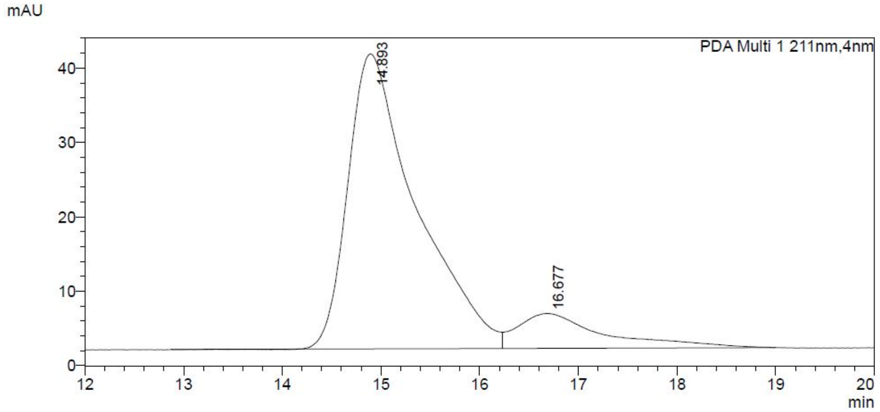

<Peak Table>

PDA Ch1 211nm

Peak#	Ret. Time	Area%
1	14.893	87.027
2	16.677	12.973
Total		100.000

HPLC data for **1-Benzyl-3-ethyl-2-oxoindolin-3-yl isobutyrate S12**: Chiralpak AD-H (98:2 hexane:IPA, flow rate 1.0 mLmin^{-1}, 211 nm, 30 °C) t_R (*S*): 14.6 min, t_R (*R*): 17.1 min, 71:29 (*S*:*R*) er.

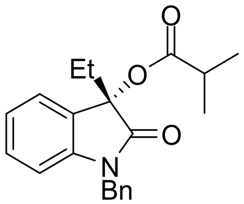

S12

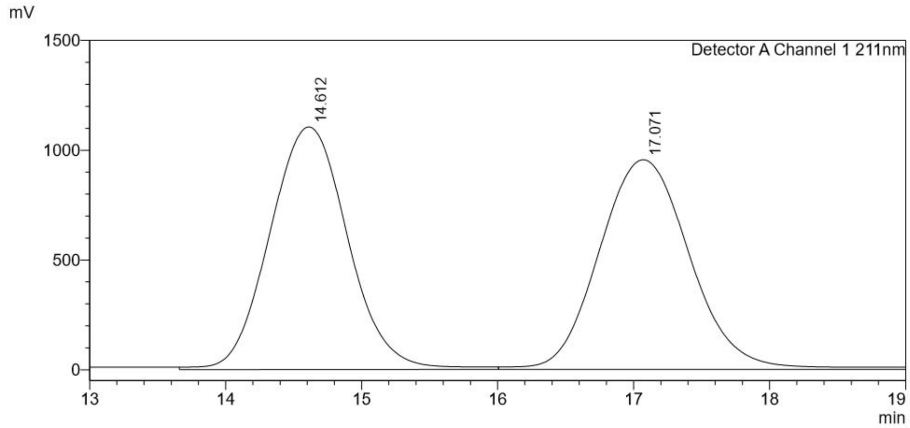

<Peak Table>

Detector A Channel 1 211nm

Peak#	Ret. Time	Area%
1	14.612	48.459
2	17.071	51.541
Total		100.000

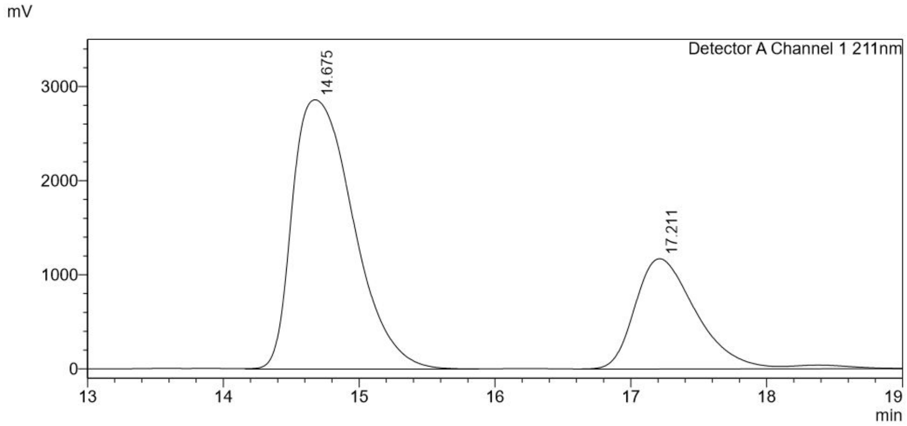

<Peak Table>

Detector A Channel 1 211nm

Peak#	Ret. Time	Area%
1	14.675	70.687
2	17.211	29.313
Total		100.000

HPLC data for ***(S)*****-3-Allyl-1-benzyl-2-oxoindolin-3-yl isobutyrate S13** where the conditions were previously described by our group[2]: Chiralpak AD-H, (98:2 hexane:IPA, flow rate 1.5 mLmin^{-1}, 211 nm, 40 °C) t_R (*S*): 6.8 min, t_R (*R*): 8.1 min, 66:34 (*S*:*R*) er.

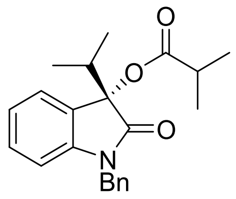

S13

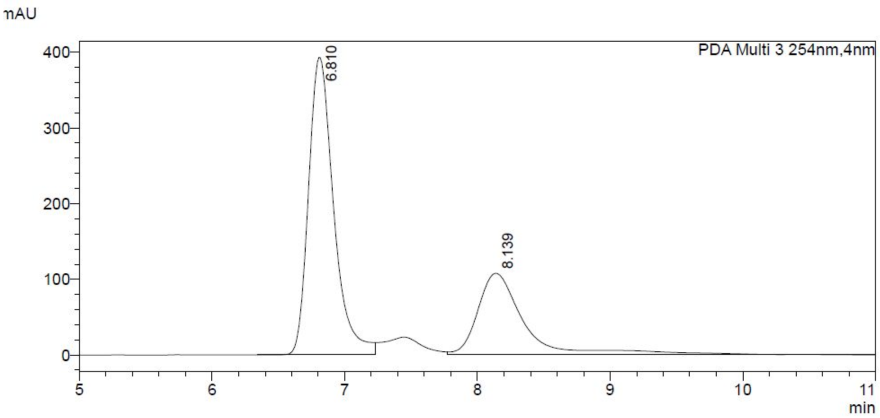

<Peak Table>

PDA Ch3 254nm

Peak#	Ret. Time	Area%
1	6.810	65.749
2	8.139	34.251
Total		100.000

HPLC data for **2-Methyl-1-phenylpropyl isobutyrate S14**: Chiralpak OJ-H (99.5:0.5 hexane:IPA, flow rate 1.0 mLmin^{-1}, 220 nm, 30 °C) t_R (*R*): 4.5 min, t_R (*S*): 5.8 min, 93:7 (*R*:*S*) er.

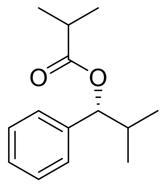

S14

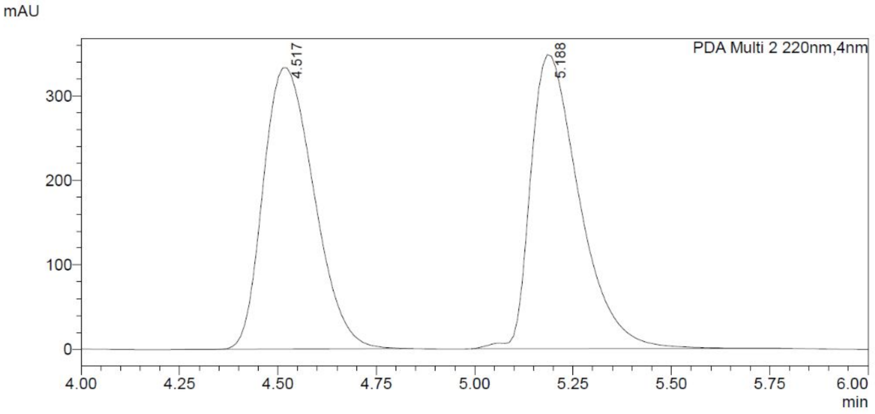

PDA Ch2 220nm

Peak#	Ret. Time	Area%
1	4.517	49.985
2	5.188	50.015
Total		100.000

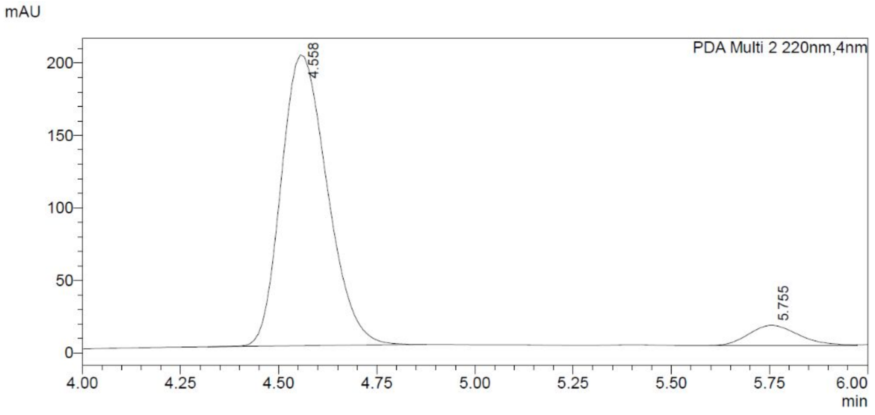

<Peak Table>

PDA Ch2 220nm

Peak#	Ret. Time	Area%
1	4.558	92.820
2	5.755	7.180
Total		100.000

HPLC data for **2,2-Dimethyl-1-phenylpropyl isobutyrate S15**: Chiralpak AD-H (99.8:0.2 hexane:IPA, flow rate 1.0 mLmin^{-1}, 270 nm, 30 °C) t_R (*R*): 5.0 min, t_R (*S*): 6.5 min, 94:6 (*R*:*S*) er.

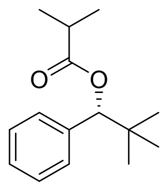

S15

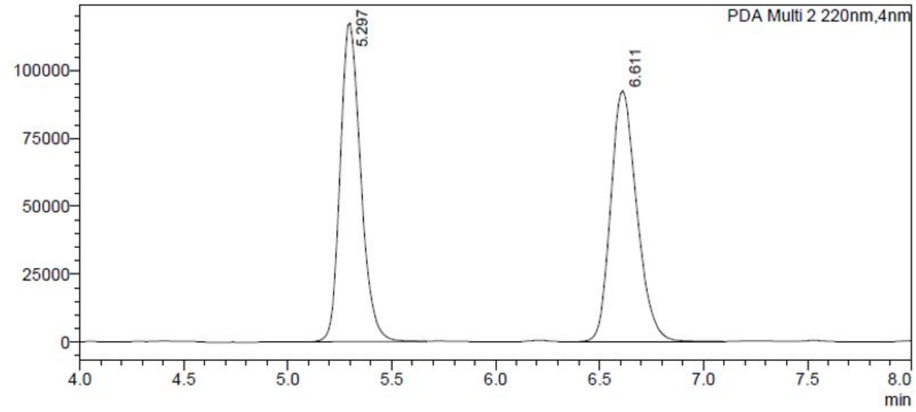

PDA Ch2 220nm

Peak#	Ret. Time	Area%
1	5.297	49.734
2	6.611	50.266
Total		100.000

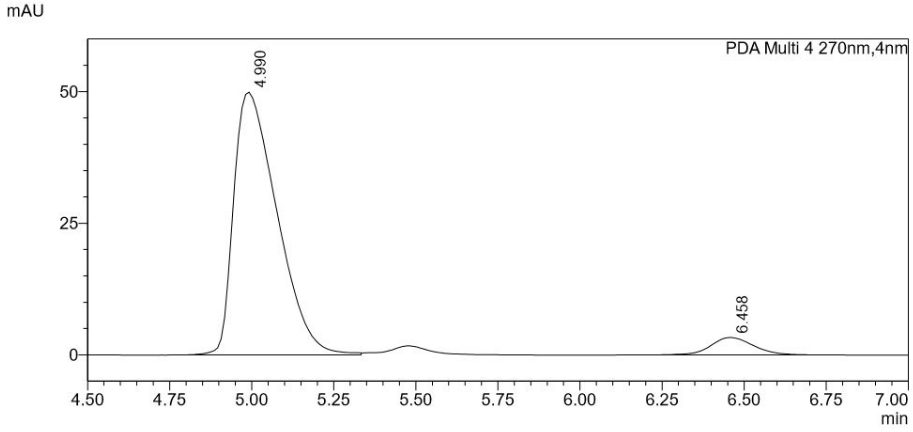

<Peak Table>

PDA Ch4 270nm

Peak#	Ret. Time	Area%
1	4.990	93.786
2	6.458	6.214
Total		100.000

HPLC data for **2,2-Dimethyl-1-(4-(trifluoromethyl)phenyl)propyl isobutyrate S16**: Chiralpak AD-H (99.8:0.2 hexane:IPA, flow rate 1.0 mLmin^{-1}, 211 nm, 30 °C) t_R (*R*): 5.1 min, t_R (*S*): 5.7 min, 87:13 (*R*:*S*) er.

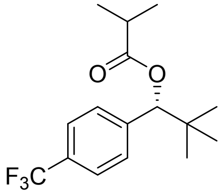

S16

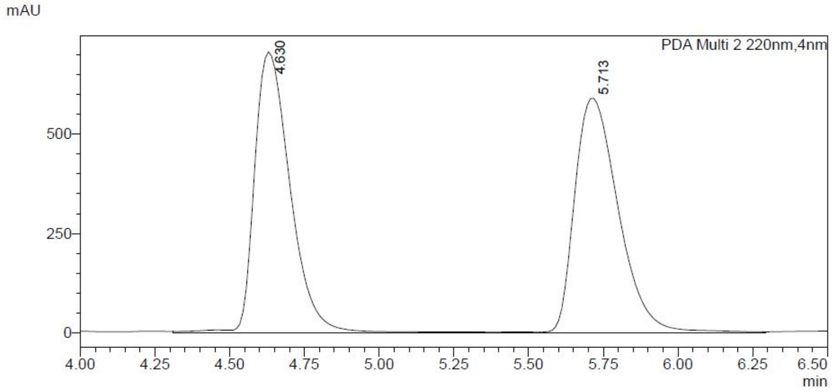

<Peak Table>

PDA Ch2 220nm

Peak#	Ret. Time	Area%
1	4.630	49.591
2	5.713	50.409
Total		100.000

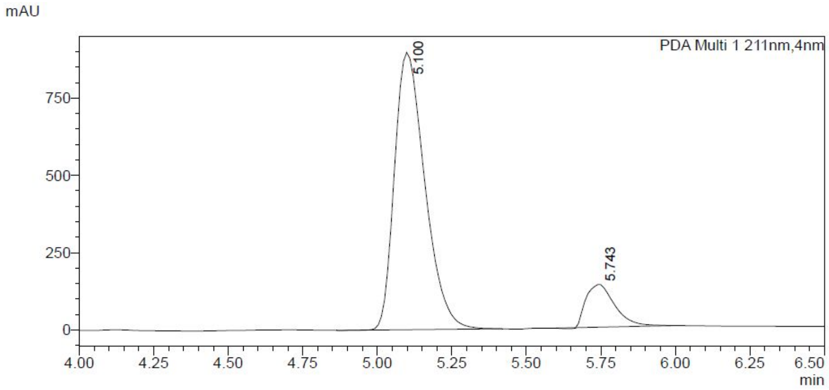

<Peak Table>

PDA Ch1 211nm

Peak#	Ret. Time	Area%
1	5.100	87.225
2	5.743	12.775
Total		100.000

HPLC data for **1-(4-Chlorophenyl)-2,2-dimethylpropyl isobutyrate S17**: Chiralpak AD-H (99.5:0.5 hexane:IPA, flow rate 1.0 mLmin^{-1}, 211 nm, 30 °C) t_R (*S*): 4.3 min, t_R (*R*): 4.6 min, >99:1 (*R*:*S*) er.

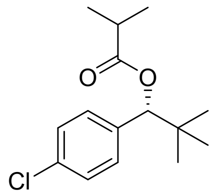

S17

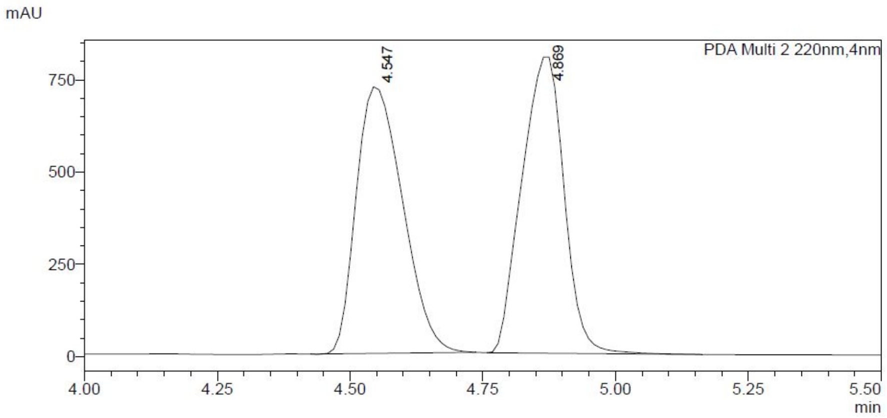

<Peak Table>

PDA Ch2 220nm

Peak#	Ret. Time	Area%
1	4.547	49.873
2	4.869	50.127
Total		100.000

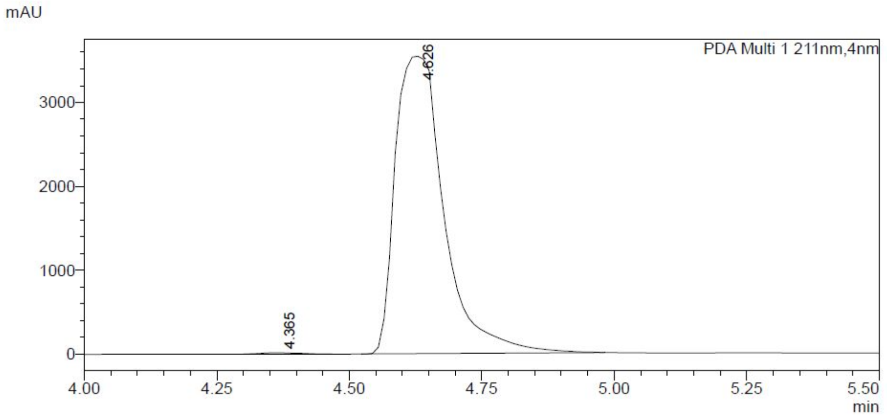

<Peak Table>

PDA Ch1 211nm

Peak#	Ret. Time	Area%
1	4.365	0.521
2	4.626	99.479
Total		100.000

HPLC data for **2,2-Dimethyl-1-(*p*-tolyl)propyl isobutyrate S18**: Chiralpak AD-H (99.5:0.5 hexane:IPA, flow rate 1.0 mLmin^{-1}, 211 nm, 30 °C) t_R (*R*): 4.4 min, t_R (*S*): 5.0 min, 79:21 (*R*:*S*) er.

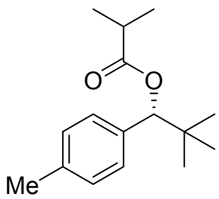

S18

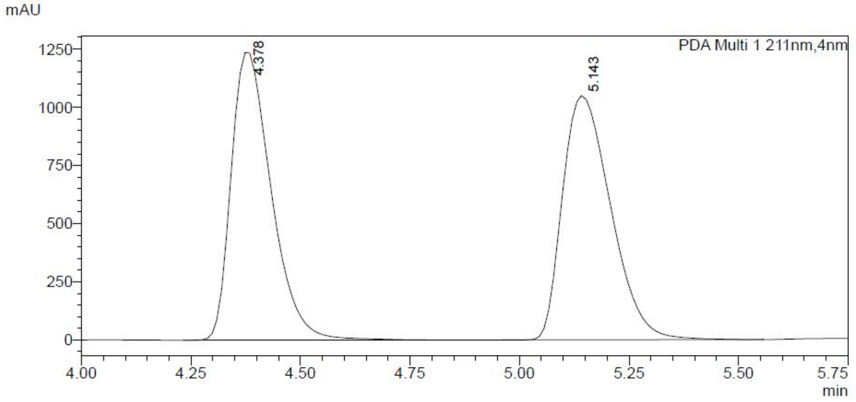

<Peak Table>

PDA Ch1 211nm

Peak#	Ret. Time	Area%
1	4.378	49.458
2	5.143	50.542
Total		100.000

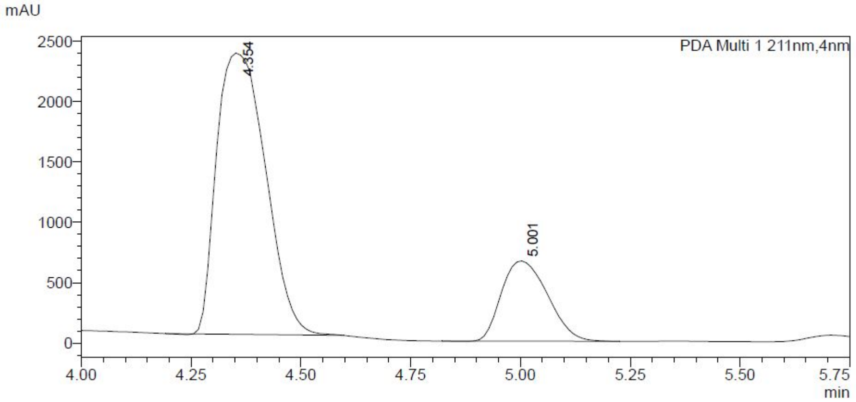

<Peak Table>

PDA Ch1 211nm

Peak#	Ret. Time	Area%
1	4.354	78.841
2	5.001	21.159
Total		100.000

HPLC data for **2,2-Dimethyl-1-(naphthalen-2-yl)propyl isobutyrate S19**: Chiralpak AD-H (99.5:0.5 hexane:IPA, flow rate 1.0 mLmin^{-1}, 220 nm, 30 °C) t_R (*S*): 5.7 min, t_R (*R*): 4.3 min, 84:16 (*R*:*S*) er.

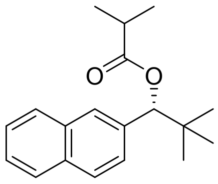

S19

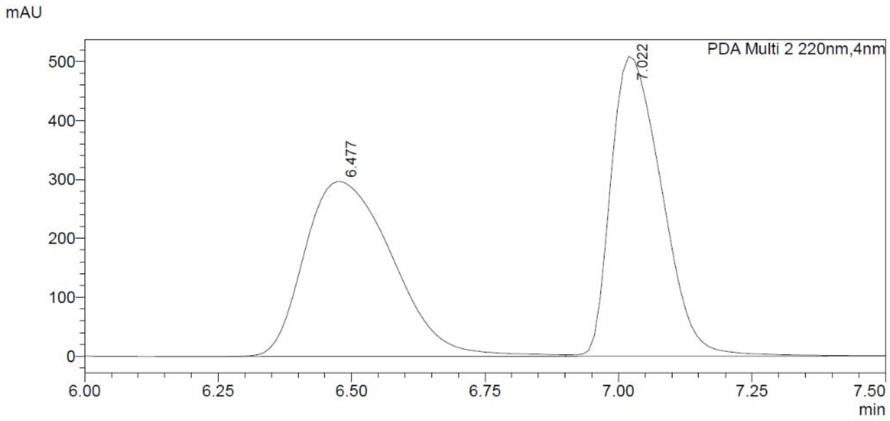

<Peak Table>

PDA Ch2 220nm

Peak#	Ret. Time	Area%
1	6.477	50.183
2	7.022	49.817
Total		100.000

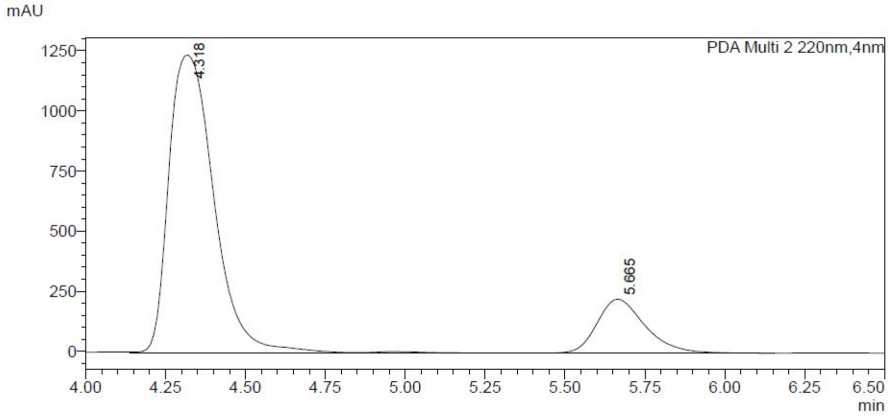

<Peak Table>

PDA Ch2 220nm

Peak#	Ret. Time	Area%
1	4.318	84.024
2	5.665	15.976
Total		100.000

HPLC data for **1-(4-Methoxyphenyl)-2,2-dimethylpropyl isobutyrate S20**: Chiralpak AD-H (99.5:0.5 hexane:IPA, flow rate 1.0 mLmin^{-1}, 220 nm, 30 °C) t_R (*R*): 4.4 min, t_R (*S*): 5.8 min, 94:6 (*R*:*S*) er.

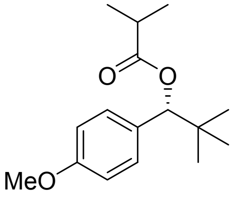

S20

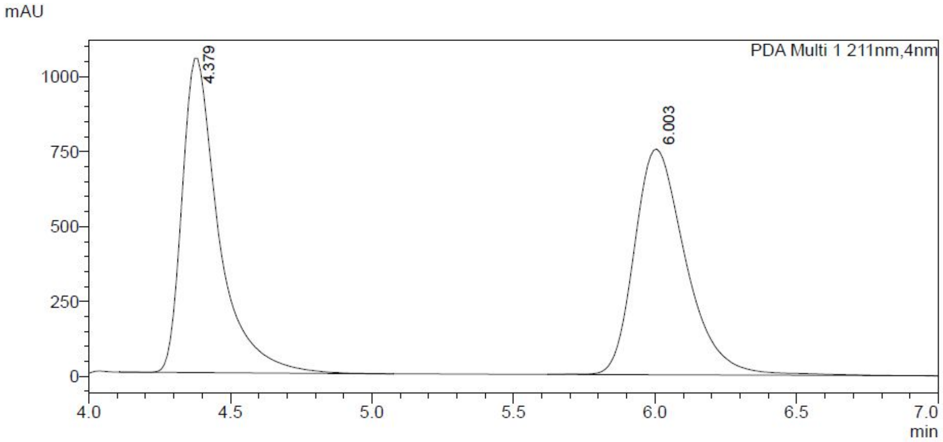

<Peak Table>

PDA Ch1 211nm

Peak#	Ret. Time	Area%
1	4.379	49.761
2	6.003	50.239
Total		100.000

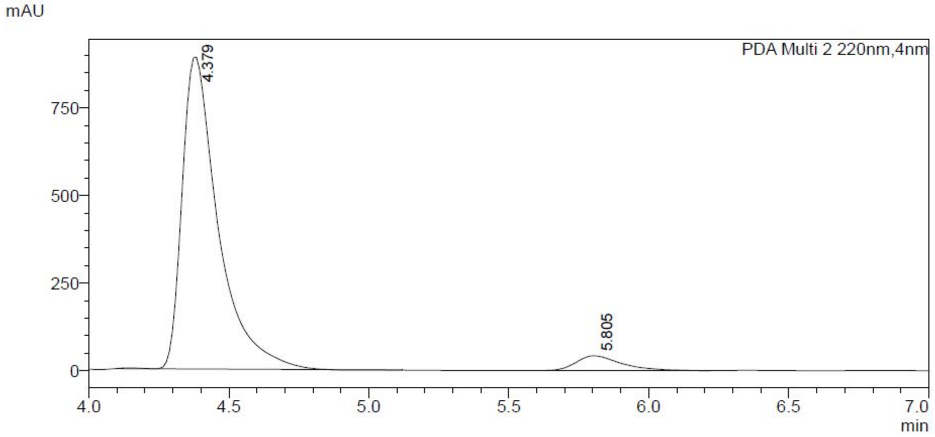

<Peak Table>

PDA Ch2 220nm

Peak#	Ret. Time	Area%
1	4.379	94.456
2	5.805	5.544
Total		100.000

HPLC data for **4-Phenylbut-3-yn-2-yl isobutyrate S21**: Chiralpak AS-H (99.8:0.2 hexane:IPA, flow rate 1.0 mLmin^{-1}, 254 nm, 30 °C) t_R (*R*): 4.3 min, t_R (*S*): 4.2 min, 73:27 (*R*:*S*) er.

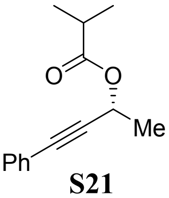

S21

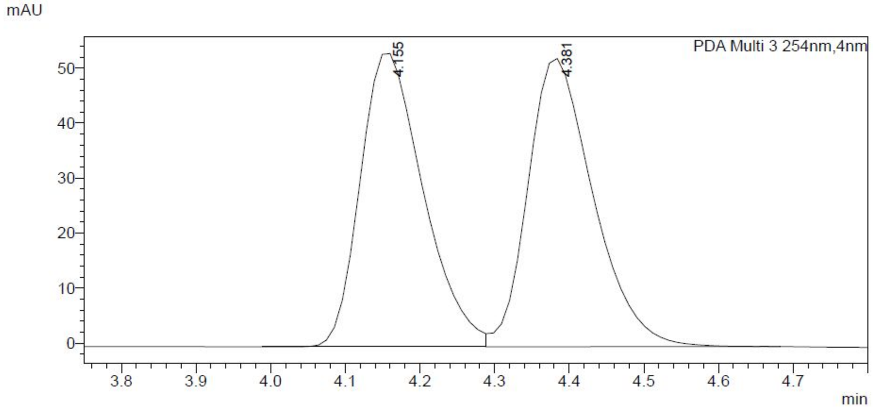

<Peak Table>

PDA Ch3 254nm

Peak#	Ret. Time	Area%
1	4.155	49.471
2	4.381	50.271
3	8.830	0.258
Total		100.000

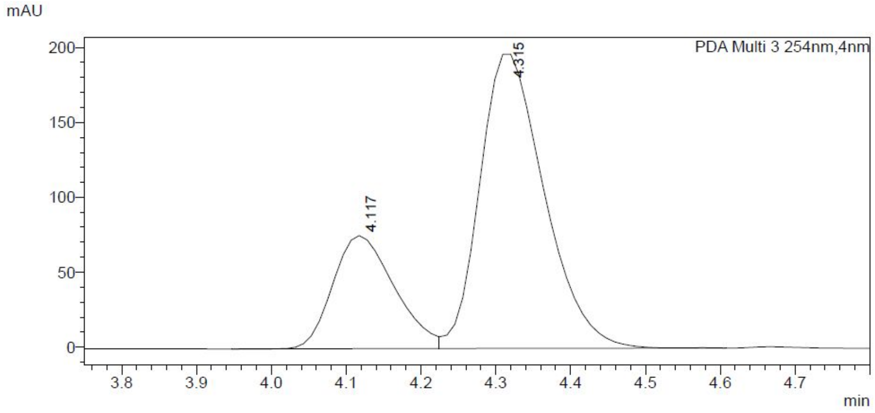

<Peak Table>

PDA Ch3 254nm

Peak#	Ret. Time	Area%
1	4.117	26.879
2	4.315	73.121
Total		100.000

F

HPLC data for **(*E*)-4-Phenylbut-3-en-2-yl isobutyrate S22**: Chiralpak AD-H (99.8:0.2 hexane:IPA, flow rate 1.0 mLmin^{-1}, 220 nm, 30 °C) t_R (*R*): 6.2 min, t_R (*S*): 6.8 min, 87:13 (*R*:*S*) er.

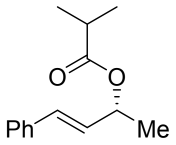

S22

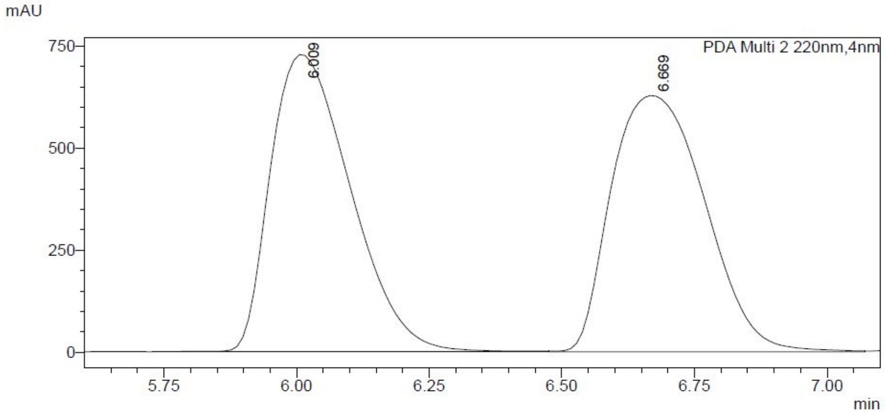

<Peak Table>

PDA Ch2 220nm

Peak#	Ret. Time	Area%
1	6.009	49.785
2	6.669	50.215
Total		100.000

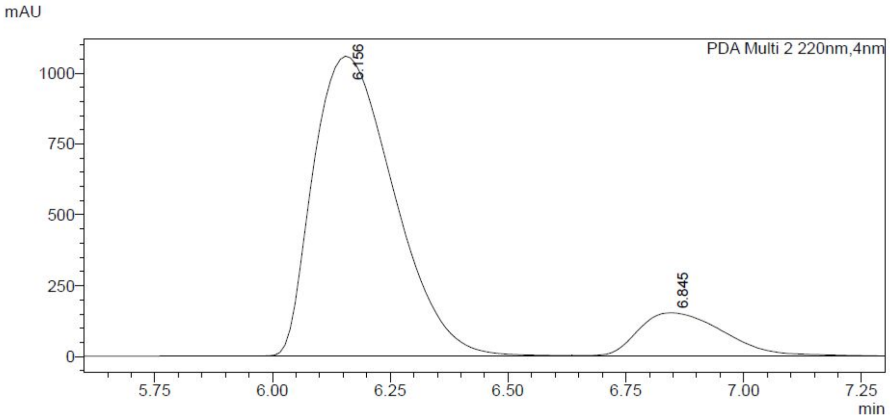

<Peak Table>

PDA Ch2 220nm

Peak#	Ret. Time	Area%
1	6.156	86.506
2	6.845	13.494
Total		100.000
